# Response of marine microbes to iron contained in colloids of glacial origin: a Kerguelen Island case study

**DOI:** 10.1093/ismeco/ycaf093

**Published:** 2025-06-03

**Authors:** Rhea Thoppil, Stéphane Blain, Rui Zhang, Audrey Guéneuguès, Olivier Crispi, Philippe Catala, Barbara Marie, Ingrid Obernosterer

**Affiliations:** Sorbonne Université, CNRS, Laboratoire d’Océanographie Microbienne (LOMIC), 66650 Banyuls-sur-Mer, France; Sorbonne Université, CNRS, Laboratoire d’Océanographie Microbienne (LOMIC), 66650 Banyuls-sur-Mer, France; Sorbonne Université, CNRS, Laboratoire d’Océanographie Microbienne (LOMIC), 66650 Banyuls-sur-Mer, France; School of Life Sciences and Medicine, Shandong University of Technology, 255000 Zibo, Shandong, China; Sorbonne Université, CNRS, Laboratoire d’Océanographie Microbienne (LOMIC), 66650 Banyuls-sur-Mer, France; Sorbonne Université, CNRS, Laboratoire d’Océanographie Microbienne (LOMIC), 66650 Banyuls-sur-Mer, France; Sorbonne Université, CNRS, Laboratoire d’Océanographie Microbienne (LOMIC), 66650 Banyuls-sur-Mer, France; Sorbonne Université, CNRS, Laboratoire d’Océanographie Microbienne (LOMIC), 66650 Banyuls-sur-Mer, France; Sorbonne Université, CNRS, Laboratoire d’Océanographie Microbienne (LOMIC), 66650 Banyuls-sur-Mer, France

**Keywords:** glacial colloids, iron, marine prokaryotes, microbial community composition, metagenomics, Southern Ocean

## Abstract

The trace element iron (Fe) is a major constraint for microbially mediated processes in the Southern Ocean. The accelerated melting of glaciers could present a novel source of Fe, but whether glacial Fe is bioavailable to marine microbes is not known. We investigated the response of marine heterotrophic prokaryotes to Fe contained in colloids (20–200 nm) of glacial origin collected on Kerguelen Island (Southern Ocean). We followed prokaryotic growth in incubation experiments amended with colloids of either glacial or nonglacial origin and determined community composition and the abundance of genes involved in Fe-related processes in metagenomes and metagenome-assembled genomes (MAGs) at the final time point. Prokaryotic taxa belonging to *Vibrionaceae* and *Pseudomonadaceae* accounted together for 32% to 67% of the relative abundances in the glacial colloid–amended treatments, while *Rhodobacteraceae*, *Flavobacteriaceae*, and *Alteromonadaceae* were the dominant contributors to the communities in the incubations amended with nonglacial colloids. Metagenomic analysis revealed a higher abundance of genes involved in the biosynthesis of the siderophores pyoverdine and vibrioferrin as well as their respective transporters in the presence of glacial colloids compared to nonglacial colloids. Genes for the transport of both siderophores were present in diverse MAGs, while biosynthesis genes were detected in fewer MAGs. Our results suggest that the utilization of siderophores facilitates access to Fe from glacial colloids and points to the key role of specific prokaryotes in rendering this source of Fe available to Southern Ocean microbial communities.

## Introduction

The meridional circulation in the Southern Ocean (SO) is characterized by an intense circumpolar upwelling [1] that supplies the surface ocean with high concentrations of major nutrients. However, primary production of phytoplankton remains low due to the chronic limitation of the micronutrient iron (Fe). The availability of Fe constrains the drawdown of nutrients and CO_2_, and Fe further regulates the production of phytoplankton-derived dissolved organic carbon (DOC), with consequences that affect the microbial food web. Contrasting with this global view, localised phytoplankton blooms have been recorded and different sources of Fe such as islands [2] and hydrothermal activity [3] have been identified. The naturally Fe-fertilized Kerguelen bloom is one of the most documented examples of the effects of Fe with respect to Fe inputs and seasonal cycling [4–8]. Incubation experiments have revealed the complex interplay between Fe and other potential limiting factors like silicon and light for diatoms [9] or DOC for heterotrophic prokaryotes [10, 11]. Despite extensive research, not all sources of Fe sustaining the phytoplankton blooms in the region of Kerguelen have been identified.

The melting of ice sheets, glaciers, and icebergs has been considered as a source of Fe in polar oceans [8, 12]. Glacial Fe, as a result of glacial erosion, exists in different chemical forms which are not equally bioavailable, which leads to consequences affecting microbial and higher trophic level productivity [13]. Particulate minerals with low solubility make up a large fraction of glacial Fe [14]. The formation of authigenic Fe particles occurs in oxic aqueous microenvironments beneath the ice, where Fe(II)-bearing rocks are oxidized [15]. Portions of these minerals are colloids, found in the nanometer-sized fraction (20–200 nm), where Fe is present as ferrihydrite, an (oxy)hydroxide form [16]. In contrast to rapidly sinking particles, colloids can be transported off the coast, thus rendering this form of Fe potentially available to microbial communities in the open ocean. Colloids have recently received attention due to their roles in the regulation of the Fe supply from sediments [17] and as a vector for Fe cycling through the colloidal shunt, which acts as an intermediate between the dissolved and particulate phases of Fe [18].

Microbially mediated transformations may be required to access Fe contained within colloids. One pathway of interest is the microbial production of siderophores, which are low–molecular weight compounds (500–1500 Da) that can strongly chelate to Fe in seawater [19]. These organic ligands are thereby capable of rendering chemically complex sources of Fe bioavailable. The role of siderophores for lithogenic Fe sources has been acknowledged in mineral weathering in the soil [20]. Insights from experimental studies using bacterial strains showed that solubilisation of Fe and silica was accomplished by siderophores like pyoverdine and pyochelin that are produced by *Pseudomonadaceae* from smectite, a mineral produced from the weathering of rocks [21], as well as other materials like kaolinite [22], ferrihydrite [23], and olivine [24]. Petrobactin, a siderophore produced by the bacterial strain *Alteromonas* was shown to render colloidal Fe bioavailable [25], and diverse siderophores, including vibrioferrin and petrobactin, are crucial for enabling marine microbial communities to access Fe contained in atmospheric dust [26, 27]. The objective of this study was to determine the taxonomic and functional responses of marine prokaryotic communities to colloidal Fe of glacial origin.

## Material and methods

**Figure 1:**
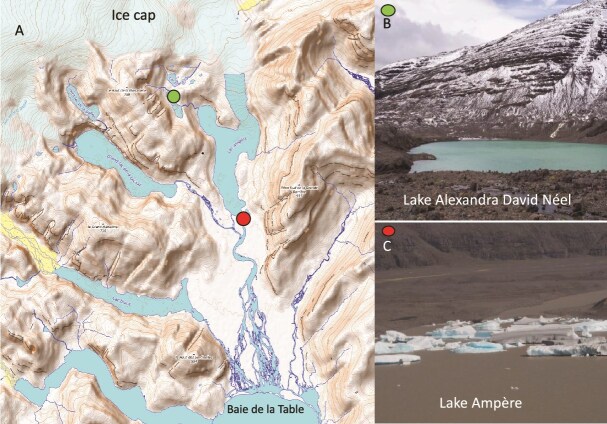
Illustrative representation of the sampling sites: (A) Overview of the sampling locations on Kerguelen Island (B) Lake Alexandra David Néel and (C) Lake Ampére.

Our study was carried out on Kerguelen Island (Indian Sector of the SO, 49°S, 70°E) during the Project Bioavailability of Iron contained in Nanoparticles of Glacial Origin (BINGO) from December 2019 to January 2020. Samples were collected in the region around the Glacier Ampère, a glacier that is one of the main outlets of the Cook Ice Cap (CIC) and has been experiencing dramatic wastage since 1960 [28]. For the collection of colloids, we sampled two lakes that are geographically close (0.6 km). Both of these lakes have a mineral-type catchment area, dominated by basalt, but they differ with respect to the influence of the CIC (Fig. 1). Lake Ampère is directly connected with one of the glaciers (Glacier Ampère) of the CIC and therefore receives icebergs and meltwater influenced by glacial erosion. The outflow of Lake Ampère enters the coastal site *Baie de la Table*. By contrast, Lake Alexandra David Néel is located at an altitude of 200 m, with no connection to the CIC, and this lake has therefore no contemporary input of glacial meltwater. For clarity, we will refer in the following to Lake Ampère as a glacial lake and to Lake Alexandra David Néel as a nonglacial lake.

Water samples were collected using a 6-L Niskin bottle that was deployed horizontally about 5 m from the coast. The Niskin bottle was closed from distance and pulled back to the shore, and then turned vertically, and samples were then drawn from the spigot. For the environmental characterization of these two freshwater systems, samples were taken for the major inorganic nutrients nitrate (NO_3_^−^), phosphate (PO_4_^3−^), silicic acid (Si(OH)_4_), iron (Fe), and DOC, as well as prokaryotic cell abundances.

### Microbial incubation experiments

To determine the response of marine prokaryotic communities to colloids originating from the two different lakes, we performed incubation experiments as described in detail below. Briefly, we set up batch cultures with marine microbial communities collected at a coastal site (*Baie de la Table*, Fig. 1) which were amended with colloids from either the glacial or nonglacial lake.

#### Preparation of colloids by ultrafiltration

Colloids were obtained from both lakes by ultrafiltration. Roughly 6 L of water was sequentially filtered through 0.8 µm and 0.2 μm pore size polycarbonate (PC) filters, and the < 0.2 μm filtrate was then passed through an ultrafiltration system (Pellicon, Millipore) equipped with 100-kDa cassette filters. The retentate (RUF) contained colloids >100 kDa, corresponding to ~20 nm. To obtain the RUF, we used a similar concentration factor for water from both lakes (22.8 and 22 for the glacial and nonglacial lake, respectively), and the colloids present in these retentates were transferred to the incubations (see below) using a dilution factor of 22.

#### Sampling of marine microbial communities for the incubation experiments

Microbial communities were collected at a coastal site (*Baie de la Table*, 85 m overall depth) located in the fjord near the outflow of the river originating in the glacial lake (Fig. 1). Seawater was collected by hand from a dinghy using an acid-cleaned PC bottle. Seawater was filtered through 0.8 μm PC filters, and the < 0.8 μm filtrate was used as inoculum.

#### Experimental setup

The microbial communities contained in the < 0.8 μm filtrate were mixed with 0.2 μm filtered seawater in a 1:10 ratio. The following treatments were set up, each in biological triplicates: one treatment amended with colloids from the glacial lake (diluted 1:22 from the concentrates described above) and one treatment with colloids from the nonglacial lake (diluted 1:22). Unamended incubations served as control. Aged seawater (stored in the dark for >10 years) from the Mediterranean Sea was used to prepare the 0.2 μm filtered seawater for the incubations. This approach was chosen to assure a low Fe and organic carbon growth medium for the microbial incubations. The 0.2 μm filtration of the aged seawater was carried out under trace-metal clean conditions in the home laboratory. The filtrates were prepared in two volumes, 30 mL (to follow cell abundance) and 500 mL (for diversity and metagenomic analyses) in acid-washed PC bottles, and the lids were covered with parafilm to avoid any contamination during the transport. In the laboratory at the base on Kerguelen Island (Port aux Francais, PAF), the PC bottles were opened and the respective volumes of the < 0.8 μm filtrates and colloids were added. The preparation of the cultures and subsampling were carried out under a laminar flow hood (ADS Laminaire). The final volumes of the batch cultures were 34.5 and 575 mL, respectively, and the cultures were incubated at 6°C in the dark in a cooled incubator (Panasonic). All flasks were sealed with parafilm during the incubation period. The 34.5 mL cultures (biological triplicates per treatment and time point, 36 bottles in total) were used for subsampling of prokaryotic cell abundance during the incubation period (10 days), and the 575 mL cultures (biological triplicates per treatment for the final time point, 9 bottles in total) were used for the collection of samples for prokaryotic diversity and metagenomic analyses at the final time point. We chose to follow prokaryotic cell abundances in small incubation bottles (30 mL) to avoid potential contamination of the cultures during subsampling. Therefore, triplicate bottles for each treatment were sacrificed at each time point. Analyses of inorganic nutrients and DOC, and the enumeration of nonphototrophic prokaryotes, was done using standard methods that are described in the Supplementary Material.

#### Sample collection for microbial community composition and metagenomics

For the description of the prokaryotic community composition and metagenomics, 500 mL batch culture was concentrated on 0.22 μm cartridges (Sterivex™ Millipore, EMD, Billerica, MA) at the end of the microbial incubations. The cartridges were sealed at both ends using parafilm and stored at −80°C until DNA extraction. Extractions were performed using the AllPrep Kit (Qiagen, Hilden, Germany) as described in [29] with minor modifications. The filter units were handled according to standard methods as described in the Supplementary Material. DNA concentration was measured using the QuantiFluor® Double-stranded DNA (dsDNA) system. The DNA extracted from 2 replicates of the control was lost during transport to the sequencing company. We have therefore focused in the following on the comparison between the two treatments (glacial and nonglacial colloids).

#### Microbial community composition

The PCR amplification of DNA extracts was performed using the primers 515F–Y (5′-GTGYCAGCMGCCGCGG TAA) and 926R (5′-CCGYCAATTYMTTTRAGTTT) that encompass the V4 and V5 hypervariable regions of the 16S rRNA gene [30]. The 16S rRNA gene amplicons were sequenced via next-generation sequencing (Illumina MiSeq 2 × 250-bp chemistry on one flow-cell) at the platform LGBT (Berlin, Germany). Mock community DNA (LGC standards, UK) was used as a standard for subsequent analyses and considered as a DNA sample for all treatments. Raw sequences (Table S1) were demultiplexed and quality filtered using the Illumina *bcl2fastq* v.2.20 at the platform Biosearch Technologies (Berlin, Germany), which can be accessed in the European Nucleotide Archive (ENA) repository at https://www.ebi.ac.uk/ena under the project ID PRJEB74701. Taxonomy was assigned to the amplicon sequence variants (ASVs) based on the *GTDB* v.202 reference database [31,32] using *DADA2* v1.24 [33] in *R* v4.2.1 [34]. The number of reads per sample varied between 10 062 and 282 039 for the 8 samples (including the only control and initial community used as inoculum). To achieve random subsampling, the samples were rarefied to 10 062 reads using *phyloseq* v1.40 [35] in R. The resulting ASVs were subjected to non-metric dimensional scaling (nMDS) ordinations based on Bray–Curtis dissimilarity [36], similarity percentage analysis (SIMPER) [37], and analysis of similarity (ANOSIM) using *phyloseq* v1.40 and *vegan* v2.6 [38] as described in the Supplementary Material.

#### Metagenomic gene sequencing

The metagenomics samples were sequenced using an Illumina NovaSeq 6000 system with 2 × 150 bp chemistry at Fasteris SA, Inc. (Switzerland). This sequencing yielded 186–272 million pairs of metagenomic reads per sample, totaling 261.8 GB (Table S2). The datasets can be accessed in the ENA repository under the project ID PRJEB74700. The raw reads were checked with FastQC and processed using *Trimmomatic* (v 0.32) [39]. Since the same microbial community (coastal site *Baie de la Table*) was used as inoculum for both treatments a co-assembly metagenome approach (Fig. S1) was considered.

The short reads from the six samples that passed the quality control were co-assembled by using *megahit* v1.2.9 [40] with a minimum length of 1000 bp. For comparison, *spades* v3.15.5 [41] was also used for assembly of the short reads with the parameter*—meta*. Based on the comparison between both assemblers using *quast* v.5.0.2 [42], *megahit* was used for further analyses. To develop a nonredundant database, the contig file was reformatted with *anvi’o* [43] with the parameter *—min-len 2000*. *Prodigal* v2.6.3 was used to predict the open reading frames (ORFs) [44] with the parameters *-p meta*. *Salmon* v.1.10.2 [45] was later used for quantifying each gene in each sample and for assigning the short reads to the nonredundant gene sequences in the database with parameters set at *—meta*. The quantified occurrences of each gene in each sample were normalized as a metric of genes per kilobase million (GPM) for the metagenome to eliminate the library size effect and ensure comparability between the samples, as explained in the Supplementary Material [46]. The tool *FeGenie* [47] was used for detection of genes with Fe-related pathways based on separate contig files for subsequent alignment and annotation of each gene detected. This tool uses Hidden Markov Models (HMMs) along with *BLAST* to annotate Fe-related genes. In the present dataset, the genes that were identified passed the predetermined bit score cutoffs as given in the *FeGenie* [48]. Although not all subunits of each gene family in each sample were identified (summarized in Table S1 in [48], it must be noted that the HMMs developed in *FeGenie* are sensitive to the presence of the entire gene family. To retrieve the GPM for each identified gene at 100% similarity, the gene corresponding to the respective Fe-related pathway in each sample was merged with gene coverage data obtained using *anvi’o* [43] with parameter *anvi-profile-blitz* combined with *salmon* [45] gene count data. This final dataset was used to compute statistical comparisons between the glacial and nonglacial amended samples using two-sample Students *t* tests and a statistically significant result considered as *P* <0.05. Gene families of interest were taxonomically screened using *BLAST+* [49].

#### Genome-resolved metagenomics

For effective binning, we used the *anvi’o* pipeline [50]. Short reads from the co-assembled metagenomes were mapped to the scaffolds using *bowtie2* v2.5.1 [51] and stored as BAM files with *samtools* v.1.15.1 [52] with a minimum identity of 95%. Using the *anvi-init-bam* option from *anvi’o*, the alignment files of each sample were indexed and later used to create metagenome-assembled genomes (MAGs) for an enhanced depiction of the microbial-associated functions. Since the metagenomes were co-assembled, we expected the highest completion of MAGs. Using *anvi’o*, each BAM file was profiled to obtain the coverage and detection statistics of each scaffold to create combined mapping profiles which were then merged into a profile database. Contigs were manually binned using *MetaBAT* v.2.15 [53], *MaxBin* v.2.2.7 [54], and *CONCOCT* v.1.10 [55] to ensure comparability between each binning tool. To retrieve a nonredundant set of bins, *DAS_Tool* v1.1.3 [56] was used to dereplicate, aggregate and score each MAG. The resulting MAGs were imported into *anvi’o* for refinement based on their GC-content and sequence composition. Later, taxonomic identification of the MAGs was performed internally with *anvi’o* and *GTDB-Tk* [57]. The relative abundance of each MAG was determined by assessing the percentage of reads recruited from each MAG across all samples. *CheckM* v1.2.2 [58] and *anvi’o* were used to reassess the completion and redundancy of each MAG. As an additional screening method due to the limitations of *FeGenie*, *antiSMASH* [59] was used to detect secondary metabolite biosynthetic gene clusters (BGCs) focused on siderophore biosynthesis and siderophore transport. It must be noted that the *antiSMASH* does not show specific gene transporters for siderophores but rather common transporters for secondary metabolite biosynthesis gene clusters which may include transport-related genes for siderophores [60]. Results of these were combined with results of *FeGenie* analyses that were also conducted on the final MAGs to obtain siderophore-related gene information for each MAG.

Visualisation of all the above-mentioned data was performed using the *ggplot2* package, and heatmaps were produced with the *pheatmap* package on *R* v4.2.1 using Euclidean distance measure for distance [34].

## Results

### Environmental context

Concentrations of phosphate (PO_4_^3−^, < 0.1 μM) and nitrate (NO_3_^−^, < 1.3 μM) were substantially lower in the two lakes compared to the coastal marine site (1.45 μM for PO_4_^3−^, 17.8 μM for NO_3_^−^) (Table 1). By contrast, both lakes were enriched in silicic acid (Si (OH)_4_, > 33.5 μM) compared to the *Baie de la Table* (4.5 μM). The same pattern was observed for dissolved iron (DFe), with roughly 2 orders of magnitude higher concentrations in the freshwater systems than at the coastal site. Despite their geographical proximity, marked differences between lakes were observed for DFe. Concentrations in the glacial lake (324 nM) were 3-fold higher compared to the nonglacial lake (98 nM). DOC concentrations were low in both lakes (< 21 μM) and concentrations typical for SO surface water were determined for the *Baie de la Table* (55 μM). Prokaryotic abundances were similar between lakes and about 20-fold lower compared to the coastal site. The temperature was 3°C in the two lakes and 6°C in surface water at the coastal site.

**Table 1:** Brief description of the study sites.

	Lake Ampère (glacial)	Lake Alexandra David Néel (nonglacial)	*Baie de la Table* (coastal marine)
PO_4_^3−^ (μM)	0.12	0.05	1.45
NO_3_^−^ (μM)	1.30	0.28	17.8
Si (OH)_4_ (μM)	33.5	42.2	4.5
DFe (nM)	324	98	< 1
DOC (μM)	14	21	55
Prokaryotic abundance (x10^5^ cells mL^−1^)	0.78	1.12	23.96

DFe, dissolved iron; DOC, dissolved organic carbon; NO_3_^−^, nitrate; PO_4_^3^, phosphate; Si (OH)_4_, silicic acid

#### Nutrient conditions in the incubation experiments

The concentration of Fe in the glacial RUF was 1800 nM and in the nonglacial RUF was 450 nM, resulting in 79 nM and 20 nM colloidal Fe in the respective incubations. DOC concentrations in the glacial and nonglacial RUF were 50 and 34 μM, respectively, yielding 1.5 and 2.3 μM colloidal DOC in the respective incubations. Concentrations of DOC in the seawater medium were 62 μM, PO_4_^3−^ was 0.02 μM, and NO_3_^−^ was 1.13 μM.

#### Microbial growth and community composition in the incubation experiments

**Figure 2:**
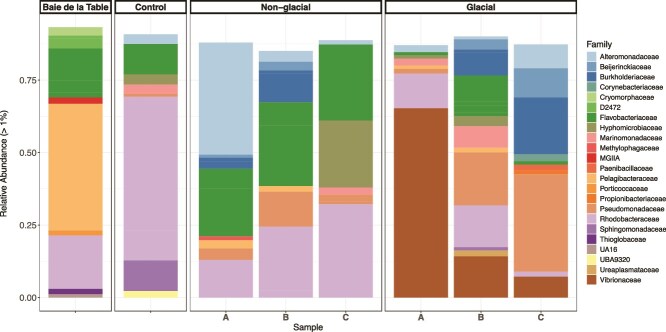
Composition of the microbial communities in the inoculum (*Baie de la Table*) and the incubations at the final time point (10 days). Relative abundance of ASVs (>1% in at least one sample) grouped at the family level in the control and each biological replicate (A, B, C) of the incubations amended with nonglacial and glacial colloids are shown.

Prokaryotic cell abundances in the batch cultures increased by up to 4-fold after 10 days of incubation (Fig. S2). Prokaryotic cell abundances were not different between treatments over the first 4 days of incubation. At the final time point, cell abundances were significantly higher (by 1.3-fold) in the treatments amended with glacial colloids (8.73 ± 0.9 × 10^5^ cells mL^−1^) compared to the control (Students *t*-test, *p* =0.029), but this was not the case for the treatments amended with nonglacial colloids (7.32 ± 1.12 × 10^5^ cells mL^−1^; *p* = 0.396 ). Prokaryotic cell abundances were statistically not different between the two treatments (Students *t*-test, *P* = 0.166) after 10 days of incubation. The observed differences in the composition of the prokaryotic communities between treatments (*ANOSIM p* = 0.023, Fig. S3) were mainly driven by the relative abundance patterns of *Vibrionaceae* and *Pseudomonadaceae* in the glacial colloid amended treatments (Fig. 2, Table S3). Despite the considerable variability among replicates, the relative abundances of *Vibrionaceae* (14%–64%) and *Pseudomonadaceae* (in 2 out of 3 replicates; 18%–33%) were overall higher in the incubations with glacial colloids compared to the incubations with nonglacial colloids (0.01%–1% for *Vibrionaceae and* 3%–11% for *Pseudomonadaceae). Vibrionaceae* were assigned to two ASVs, both belonging to the genus *Vibrio,* and *Pseudomonadaceae* were assigned to a single ASV (genus *Pseudomonas*) (Table S3). SIMPER analysis revealed that ASV5 assigned to *Vibrionaceae* contributed 18% (*p* = 0.00138) to the dissimilarity between the treatments (Table S4). *Rhodobacteraceae, Flavobacteriaceae*, and *Alteromonadaceae* were the dominant contributors to the communities in the incubations amended with non-glacial colloids (Fig. S4). ASV1 and ASV6 (*Rhodobacteraceae)* and ASV26 (*Flavobacteriaceae*) contributed between 1% and 7% (*p* = 0.00138) to the dissimilarity between treatments (Table S4). These families both also had high relative abundances in the initial community used as inoculum and in the only control available (Fig. 2).

#### Functions related to the access of Fe on the community level and in MAGs

**Figure 3:**
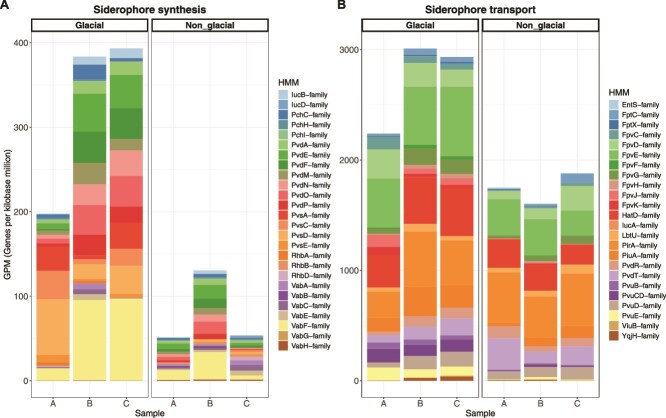
Stacked bar plots showing the normalized gene abundances (GPM) in incubations with glacial and nonglacial colloids. (A) Subunits of gene families associated with siderophore synthesis and (B) subunits of gene families associated with siderophore transport.

**Figure 4:**
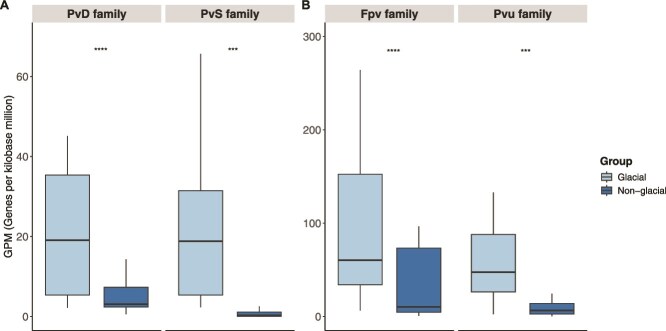
Box plots of normalized gene abundances (GPM) in incubations with glacial and nonglacial colloids. (A) Siderophore synthesis gene families *pvd* (pyoverdine) and *pvs* (vibrioferrin) and (B) siderophore transport gene families *fpv* (pyoverdine) and *pvu* (vibrioferrin). ^*^p-significance: ^***^p < 0.001; ^****^p < 0.0001.

To investigate the potential of prokaryotes in accessing Fe, we searched for Fe-related genes in the metagenomes obtained at the final time point of the incubation experiments. Using the FeGenie database, we focused our search on genes related to Fe^3+^ and Fe^2+^ transport, heme transport, and siderophore biosynthesis and transport. Normalized gene abundances (genes per kilobase million, GPM) for these gene categories were not significantly different between treatments (Student *t*-test, *p* > 0.05) (Fig. S5), except for siderophore synthesis and transport genes (Fig. 3). The siderophore synthesis gene families *pvd* (pyoverdine) and *pvs* (vibrioferrin) and the respective siderophore transport gene families *fpv* (pyoverdine) and *pvu* (vibrioferrin) were significantly higher in the incubations amended with glacial compared to nonglacial colloids (Fig. 4). The siderophore synthesis *pvd* genes were predominantly affiliated with *Pseudomonadaceae* and those of *pvs* with *Vibrionaceae* (Fig. S6). The transporter genes were taxonomically affiliated with a large range of prokaryotic groups, including *Pseudomonadaceae* and *Vibrionaceae*.

**Figure 5:**
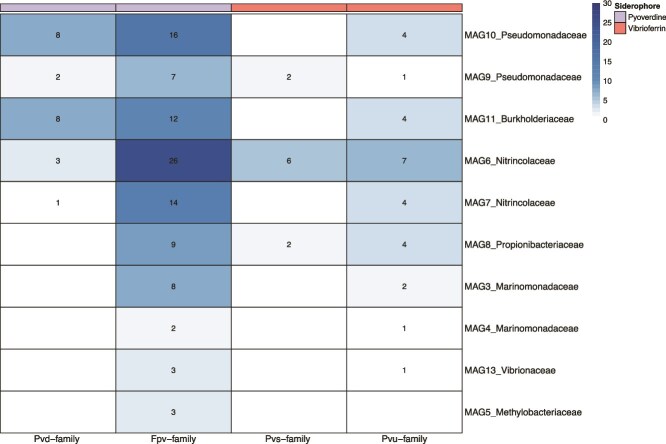
Heatmap displaying the gene counts per MAG (normalized for sequencing depth) involved in the synthesis and transport of the siderophores pyoverdine (*pvd* and *fpv*, respectively) and vibrioferrin (*pvs* and *pvu*, respectively).

We further screened 84 good-quality MAGs (Table S5) for the presence of siderophore transport and synthesis genes of pyoverdine and vibrioferrin using FeGenie. We detected one or more of these gene families in 10 MAGs (Fig. 5). For pyoverdine transport, the *fpv* gene family was detected in all 10 MAGs and one or more genes involved in the pyoverdine synthesis (*pvd)* were detected in 5 MAGs. For vibrioferrin transport, the *pvu* gene family was recorded in all MAGs, except for *Methylobacteriaceae* MAG5, and one or more genes involved in the *pvs* gene family (vibrioferrin synthesis) were detected in 3 MAGs. Gene counts for pyoverdine transport (*fpv*) were substantially higher than those for vibrioferrin transport (*pvu*), and MAGs belonging to *Nitrincolaceae*, *Pseudomonadaceae*, and *Burkholderiaceae* were dominant contributors. The 10 MAGs containing genes involved in the transport and synthesis of pyoverdine and vibrioferrin had a higher cumulative coverage in the incubations amended with glacial compared to nonglacial colloids (Fig. S7).

An additional screening using *antiSMASH* resulted in the detection of BGCs in all of the 10 MAGs illustrated in Fig. 5. Investigation of these BGCs revealed the presence of siderophore biosynthesis protein *pvs*D in *Nitrincolaceae* MAG6 and *lucA*/*lucC* siderophore biosynthesis family proteins in *Nitrincolaceae* MAG6, *Pseudomonadaceae* MAG10, and *Vibrionaceae* MAG13 (Table S6). When exploring BGCs for siderophore transport, TonB-dependent transporters (TBDTs) were identified in MAGs like *Nitrincolaceae* MAG6 and *Pseudomonadaceae* MAG10. Furthermore, the ATP-Binding Casette (ABC) transporter required for transport of siderophores were detected in 6 MAGs including *Pseudomonadaceae* MAG10 and MFS (Major Facilitator Superfamily) transporter genes were identified in 4 MAGs including *Pseudomonadaceae* MAG10.

## Discussion

Here we report the experimental testing of the taxonomic and functional response of SO prokaryotic communities to colloids of glacial origin. The colloids used in the present study were enriched in Fe, an element that can be co-limiting with DOC for prokaryotic growth in the SO [11,61]. We observed an increased contribution of taxa belonging to *Pseudomonadaceae* and *Vibrionaceae* and a higher abundance of genes involved in the synthesis and transport of the siderophores pyoverdine and vibrioferrin in incubations amended with glacial compared to nonglacial colloids. By contrast, no differences were observed for other siderophores or Fe-related genes. These results suggest that these two siderophores could play a specific role in the access to Fe bound to glacial colloids, while a large repertoire of Fe-uptake mechanisms could be involved in the utilization of colloidal Fe independent of its origin.

Pyoverdine has been reported to be mostly produced by *Pseudomonadaceae* [62], and this metabolic trait, also observed in the present study through the taxonomic affiliation of the respective genes (Fig. S6), could explain the comparatively higher contribution of ASV9 (Fig. S4, Table S3) to the microbial community in our incubations amended with glacial colloids. Pyoverdine is a mixed-type fluorescent siderophore (Fig. S8) with strong chelating properties [63]. Ferripyoverdine is transported through TBDTs into the periplasm, where Fe^3+^ is reduced to Fe^2+^; the latter is then transported to the cytoplasm by an ABC transporter. The siderophore is not imported into the cytoplasm, but recycled [64]. This appears to be an efficient mechanism and overcomes the need for continuous synthesis of siderophores, an energy-demanding pathway. Several subunits of the *pvd* family required for pyoverdine synthesis (Fig. S8) were detected in MAGs not affiliated with *Pseudomonadaceae*. This was the case for *Burkholderiaceae* MAG11 and *Nitrincolaceae* MAG6 and MAG7, where the presence of certain subunits of the pyoverdine synthesis operon alone is insufficient to enable pyoverdine biosynthesis [65]. Vibrioferrin is a carboxylate siderophore (Fig. S8) with weak chelating strength [66]. This siderophore is highly photosensitive and following photolysis, it can form a product incapable of binding Fe [66,67]. *Vibrionaceae*, known producers of vibrioferrin [68], were highly abundant in the glacial amended treatments and the siderophore synthesis genes *pvs* were exclusively affiliated with *Vibrionaceae* (Fig. S6), supporting the idea that the capacity to synthesize and transport this siderophore could have led to a preferential growth of taxa belonging to this family.

*Nitrincolaceae* MAG6 revealed the highest counts for the transporter genes of both siderophores. In our incubations, however, the relative abundances of ASVs belonging to *Nitrincolaceae* remained low in all treatments (< 1%), likely due to unfavourable growth conditions, most importantly the lack of readily available substrates for this group, which has previously been shown to rapidly respond to phytoplankton blooms in the SO [29,46]. Furthermore, the low gene counts for siderophore synthesis in this MAG suggests an opportunistic shift where energy could be redirected from synthesis to the transport of siderophores like pyoverdine and vibrioferrin [69, 70]. This finding can support the idea that *Nitrincolaceae* are potential “cheaters” for siderophore uptake, paving the scenario for public-good interactions where microbes that lack biosynthesis genes can transport Fe bound to siderophores [71–73].

Our observations provide for the first time insights into how Fe-rich colloids of glacial origin could influence compositional and functional aspects of marine microbial communities in the SO. Our experimental approach did not allow us to account for the differences in the concentrations of colloidal Fe in the two lakes for their respective additions to the incubations. While a possible influence of colloidal Fe concentrations cannot be excluded, it is unlikely that the higher amount of Fe added in the form of glacial (79 nM) compared to nonglacial colloids (20 nM) was the main driver of our results. Using a prokaryotic Fe:C ratio of 10 to 140 μmol mol^−1^ [74] and a prokaryotic C-content of 12 fg cell^−1^ [75] we estimated a prokaryotic consumption ranging between 6 and 84 pmol Fe L^−1^ over the course of the experiment, which represents 0.006% to 0.4% of the added concentration of colloidal Fe. This suggests that both types of Fe-colloids were added in excess and a dose effect is likely to be small. Prokaryotic cell abundances were not different between the treatments amended with glacial and nonglacial colloids, further suggesting that prokaryotes could not take maximum advantage of the potentially available Fe source, most likely due to the lack of bioavailable organic carbon in our growth medium. Concentrations of abiotic variables were identical among incubations, supporting the idea that the source of colloids is likely the predominant shaping factor of the microbial response on the functional level, while a possible top-down effect by heterotrophic nanoflagellates could have resulted in the observed variability in the community composition.

From a biogeochemical perspective, the higher abundances of two dissimilar types of siderophores, a strong and weak chelator, suggests a range of chemical speciation of Fe in glacial colloids. *In situ* concentrations of siderophores are in the picomolar range [76–78] and they account for up to 10% of the DFe pool. A variety of siderophores has been quantified in the marine environment among which ferrioxamines and amphibactins are the most commonly observed [79]. Although, to the best of our knowledge, the specific siderophores pyoverdine and vibrioferrin have not been quantified in the marine environment, the presence and expression of the respective biosynthesis and transport genes by marine microbes supports the idea of their role in the ocean [27,80–82]. Moreover, the expression of catecholate siderophore biosynthetic pathways has been observed in marine particles in the SO [83]. The results from the present study extend these previous observations to Fe contained in glacial colloids. Understanding the role of specific prokaryotes in rendering Fe available to the larger microbial community will help in predicting its potential impacts on phytoplankton productivity in the SO exposed to rapid environmental forcings.

## Supplementary Material

UPDATED_SOURCEThoppil_ISMECom_Suppl_Revised_v1_ycaf093

Table_S3_ycaf093

Table_S5_ycaf093

## Dataset availability

The datasets generated during and/or analysed during the current study are available in the European Nucleotide Archive (ENA) repository under the project IDs PRJEB74700 and PRJEB74701 for the metagenome dataset and 16 s rRNA dataset respectively. (https://www.ebi.ac.uk/ena). The associated R scripts used in this study are uploaded to GitHub (https://github.com/rthoppil14/BINGO1.git).
