# Supplementary material for: Response of marine microbes to iron contained in colloids of glacial origin: a Kerguelen Island case study
Source: ISME Commun. 2025 Jun 3;5(1):ycaf093. doi: 10.1093/ismeco/ycaf093 (PMC12445661; doi:10.1093/ismeco/ycaf093)
Supplement: UPDATED_SOURCEThoppil_ISMECom_Suppl_Revised_v1_ycaf093 [file updated_sourcethoppil_ismecom_suppl_revised_v1_ycaf093.zip › Table_S3.pdf]

Table S3. List of all detected amplicon sequence variants (ASVs) with taxonomic assignments and respective relative abundances based on the type of treatments: inoculum, control, glacial and non-glacial amended incubations

OTU	Group	Sample	Abundance	Kingdom	Phylum	Class	Order	Family	Genus
ASV1	Control	Ctr-tf-IIb	0,460793545	Bacteria	Proteobacteria	Alphaproteobacteria	Rhodobacterales	Rhodobacteraceae	Sulfitobacter
ASV1	Non-glacial	LacADNRUFtfb	0,218566913	Bacteria	Proteobacteria	Alphaproteobacteria	Rhodobacterales	Rhodobacteraceae	Sulfitobacter
ASV1	Non-glacial	LacADNRUFtfc	0,212512124	Bacteria	Proteobacteria	Alphaproteobacteria	Rhodobacterales	Rhodobacteraceae	Sulfitobacter
ASV1	Baie de la Table	BdT0-2	0,184501038	Bacteria	Proteobacteria	Alphaproteobacteria	Rhodobacterales	Rhodobacteraceae	Sulfitobacter
ASV1	Non-glacial	LacADNRUFtfa	0,119358862	Bacteria	Proteobacteria	Alphaproteobacteria	Rhodobacterales	Rhodobacteraceae	Sulfitobacter
ASV1	Glacial	LacAmpRUFtf-b	0,103660512	Bacteria	Proteobacteria	Alphaproteobacteria	Rhodobacterales	Rhodobacteraceae	Sulfitobacter
ASV1	Glacial	LacAmpRUFtf-a	0,090700852	Bacteria	Proteobacteria	Alphaproteobacteria	Rhodobacterales	Rhodobacteraceae	Sulfitobacter
ASV1	Glacial	LacAmpRUFtf-c	0,016460905	Bacteria	Proteobacteria	Alphaproteobacteria	Rhodobacterales	Rhodobacteraceae	Sulfitobacter
ASV10	Glacial	LacAmpRUFtf-b	0,088975273	Bacteria	Bacteroidota	Bacteroidia	Flavobacteriales	Flavobacteriaceae	Tenacibaculum
ASV10	Control	Ctr-tf-IIb	0,06375281	Bacteria	Bacteroidota	Bacteroidia	Flavobacteriales	Flavobacteriaceae	Tenacibaculum
ASV10	Non-glacial	LacADNRUFtfb	0,061390938	Bacteria	Bacteroidota	Bacteroidia	Flavobacteriales	Flavobacteriaceae	Tenacibaculum
ASV10	Non-glacial	LacADNRUFtfa	0,045683403	Bacteria	Bacteroidota	Bacteroidia	Flavobacteriales	Flavobacteriaceae	Tenacibaculum
ASV10	Baie de la Table	BdT0-2	0,028964914	Bacteria	Bacteroidota	Bacteroidia	Flavobacteriales	Flavobacteriaceae	Tenacibaculum
ASV10	Non-glacial	LacADNRUFtfc	0,013457808	Bacteria	Bacteroidota	Bacteroidia	Flavobacteriales	Flavobacteriaceae	Tenacibaculum
ASV10	Glacial	LacAmpRUFtf-c	0,01181312	Bacteria	Bacteroidota	Bacteroidia	Flavobacteriales	Flavobacteriaceae	Tenacibaculum
ASV10	Glacial	LacAmpRUFtf-a	0,010408294	Bacteria	Bacteroidota	Bacteroidia	Flavobacteriales	Flavobacteriaceae	Tenacibaculum
ASV1001	Glacial	LacAmpRUFtf-b	0,001727675	Bacteria	Firmicutes	Bacilli	Bacillales	Bacillaceae_D	Bacillus_M
ASV1001	Baie de la Table	BdT0-2	0	Bacteria	Firmicutes	Bacilli	Bacillales	Bacillaceae_D	Bacillus_M
ASV1001	Control	Ctr-tf-IIb	0	Bacteria	Firmicutes	Bacilli	Bacillales	Bacillaceae_D	Bacillus_M
ASV1001	Non-glacial	LacADNRUFtfa	0	Bacteria	Firmicutes	Bacilli	Bacillales	Bacillaceae_D	Bacillus_M
ASV1001	Non-glacial	LacADNRUFtfb	0	Bacteria	Firmicutes	Bacilli	Bacillales	Bacillaceae_D	Bacillus_M
ASV1001	Non-glacial	LacADNRUFtfc	0	Bacteria	Firmicutes	Bacilli	Bacillales	Bacillaceae_D	Bacillus_M
ASV1001	Glacial	LacAmpRUFtf-a	0	Bacteria	Firmicutes	Bacilli	Bacillales	Bacillaceae_D	Bacillus_M
ASV1001	Glacial	LacAmpRUFtf-c	0	Bacteria	Firmicutes	Bacilli	Bacillales	Bacillaceae_D	Bacillus_M
ASV1007	Non-glacial	LacADNRUFtfb	0,000189673	Bacteria	Proteobacteria	Alphaproteobacteria	Reyranellales	Reyranellaceae	Reyranella
ASV1007	Baie de la Table	BdT0-2	0	Bacteria	Proteobacteria	Alphaproteobacteria	Reyranellales	Reyranellaceae	Reyranella
ASV1007	Control	Ctr-tf-IIb	0	Bacteria	Proteobacteria	Alphaproteobacteria	Reyranellales	Reyranellaceae	Reyranella
ASV1007	Non-glacial	LacADNRUFtfa	0	Bacteria	Proteobacteria	Alphaproteobacteria	Reyranellales	Reyranellaceae	Reyranella
ASV1007	Non-glacial	LacADNRUFtfc	0	Bacteria	Proteobacteria	Alphaproteobacteria	Reyranellales	Reyranellaceae	Reyranella
ASV1007	Glacial	LacAmpRUFtf-a	0	Bacteria	Proteobacteria	Alphaproteobacteria	Reyranellales	Reyranellaceae	Reyranella

ASV1007	Glacial	LacAmpRUFtf-b	0	Bacteria	Proteobacteria	Alphaproteobacteria	Reyranellales	Reyranellaceae	Reyranella
ASV1007	Glacial	LacAmpRUFtf-c	0	Bacteria	Proteobacteria	Alphaproteobacteria	Reyranellales	Reyranellaceae	Reyranella
ASV1008	Baie de la Table	BdT0-2	0	Bacteria	Cyanobacteriota	Cyanobacteriia	Leptolyngbyales	Leptolyngbyaceae	Phormidesmis_A
ASV1008	Control	Ctr-tf-IIb	0	Bacteria	Cyanobacteriota	Cyanobacteriia	Leptolyngbyales	Leptolyngbyaceae	Phormidesmis_A
ASV1008	Non-glacial	LacADNRUFtfa	0	Bacteria	Cyanobacteriota	Cyanobacteriia	Leptolyngbyales	Leptolyngbyaceae	Phormidesmis_A
ASV1008	Non-glacial	LacADNRUFtfb	0	Bacteria	Cyanobacteriota	Cyanobacteriia	Leptolyngbyales	Leptolyngbyaceae	Phormidesmis_A
ASV1008	Non-glacial	LacADNRUFtfc	0	Bacteria	Cyanobacteriota	Cyanobacteriia	Leptolyngbyales	Leptolyngbyaceae	Phormidesmis_A
ASV1008	Glacial	LacAmpRUFtf-a	0	Bacteria	Cyanobacteriota	Cyanobacteriia	Leptolyngbyales	Leptolyngbyaceae	Phormidesmis_A
ASV1008	Glacial	LacAmpRUFtf-b	0	Bacteria	Cyanobacteriota	Cyanobacteriia	Leptolyngbyales	Leptolyngbyaceae	Phormidesmis_A
ASV1008	Glacial	LacAmpRUFtf-c	0	Bacteria	Cyanobacteriota	Cyanobacteriia	Leptolyngbyales	Leptolyngbyaceae	Phormidesmis_A
ASV1010	Baie de la Table	BdT0-2	0	Bacteria	Firmicutes	Bacilli_A	Brevibacillales	Brevibacillaceae	Brevibacillus
ASV1010	Control	Ctr-tf-IIb	0	Bacteria	Firmicutes	Bacilli_A	Brevibacillales	Brevibacillaceae	Brevibacillus
ASV1010	Non-glacial	LacADNRUFtfa	0	Bacteria	Firmicutes	Bacilli_A	Brevibacillales	Brevibacillaceae	Brevibacillus
ASV1010	Non-glacial	LacADNRUFtfb	0	Bacteria	Firmicutes	Bacilli_A	Brevibacillales	Brevibacillaceae	Brevibacillus
ASV1010	Non-glacial	LacADNRUFtfc	0	Bacteria	Firmicutes	Bacilli_A	Brevibacillales	Brevibacillaceae	Brevibacillus
ASV1010	Glacial	LacAmpRUFtf-a	0	Bacteria	Firmicutes	Bacilli_A	Brevibacillales	Brevibacillaceae	Brevibacillus
ASV1010	Glacial	LacAmpRUFtf-b	0	Bacteria	Firmicutes	Bacilli_A	Brevibacillales	Brevibacillaceae	Brevibacillus
ASV1010	Glacial	LacAmpRUFtf-c	0	Bacteria	Firmicutes	Bacilli_A	Brevibacillales	Brevibacillaceae	Brevibacillus
ASV1014	Baie de la Table	BdT0-2	0	Bacteria	Dadabacteria	UBA1144	UBA1144	UBA1144	TMED58
ASV1014	Control	Ctr-tf-IIb	0	Bacteria	Dadabacteria	UBA1144	UBA1144	UBA1144	TMED58
ASV1014	Non-glacial	LacADNRUFtfa	0	Bacteria	Dadabacteria	UBA1144	UBA1144	UBA1144	TMED58
ASV1014	Non-glacial	LacADNRUFtfb	0	Bacteria	Dadabacteria	UBA1144	UBA1144	UBA1144	TMED58
ASV1014	Non-glacial	LacADNRUFtfc	0	Bacteria	Dadabacteria	UBA1144	UBA1144	UBA1144	TMED58
ASV1014	Glacial	LacAmpRUFtf-a	0	Bacteria	Dadabacteria	UBA1144	UBA1144	UBA1144	TMED58
ASV1014	Glacial	LacAmpRUFtf-b	0	Bacteria	Dadabacteria	UBA1144	UBA1144	UBA1144	TMED58
ASV1014	Glacial	LacAmpRUFtf-c	0	Bacteria	Dadabacteria	UBA1144	UBA1144	UBA1144	TMED58
ASV1019	Baie de la Table	BdT0-2	0	Bacteria	Proteobacteria	Gammaproteobacteria	Betaproteobacteriales	Neisseriaceae	Morococcus
ASV1019	Control	Ctr-tf-IIb	0	Bacteria	Proteobacteria	Gammaproteobacteria	Betaproteobacteriales	Neisseriaceae	Morococcus
ASV1019	Non-glacial	LacADNRUFtfa	0	Bacteria	Proteobacteria	Gammaproteobacteria	Betaproteobacteriales	Neisseriaceae	Morococcus
ASV1019	Non-glacial	LacADNRUFtfb	0	Bacteria	Proteobacteria	Gammaproteobacteria	Betaproteobacteriales	Neisseriaceae	Morococcus
ASV1019	Non-glacial	LacADNRUFtfc	0	Bacteria	Proteobacteria	Gammaproteobacteria	Betaproteobacteriales	Neisseriaceae	Morococcus
ASV1019	Glacial	LacAmpRUFtf-a	0	Bacteria	Proteobacteria	Gammaproteobacteria	Betaproteobacteriales	Neisseriaceae	Morococcus
ASV1019	Glacial	LacAmpRUFtf-b	0	Bacteria	Proteobacteria	Gammaproteobacteria	Betaproteobacteriales	Neisseriaceae	Morococcus
ASV1019	Glacial	LacAmpRUFtf-c	0	Bacteria	Proteobacteria	Gammaproteobacteria	Betaproteobacteriales	Neisseriaceae	Morococcus

ASV1022	Baie de la Table	BdT0-2	0	Bacteria	Proteobacteria	Gammaproteobacteria	Pseudomonadales	Spongiibacteraceae	Spongiibacter
ASV1022	Control	Ctr-tf-IIb	0	Bacteria	Proteobacteria	Gammaproteobacteria	Pseudomonadales	Spongiibacteraceae	Spongiibacter
ASV1022	Non-glacial	LacADNRUFtfa	0	Bacteria	Proteobacteria	Gammaproteobacteria	Pseudomonadales	Spongiibacteraceae	Spongiibacter
ASV1022	Non-glacial	LacADNRUFtfb	0	Bacteria	Proteobacteria	Gammaproteobacteria	Pseudomonadales	Spongiibacteraceae	Spongiibacter
ASV1022	Non-glacial	LacADNRUFtfc	0	Bacteria	Proteobacteria	Gammaproteobacteria	Pseudomonadales	Spongiibacteraceae	Spongiibacter
ASV1022	Glacial	LacAmpRUFtf-a	0	Bacteria	Proteobacteria	Gammaproteobacteria	Pseudomonadales	Spongiibacteraceae	Spongiibacter
ASV1022	Glacial	LacAmpRUFtf-b	0	Bacteria	Proteobacteria	Gammaproteobacteria	Pseudomonadales	Spongiibacteraceae	Spongiibacter
ASV1022	Glacial	LacAmpRUFtf-c	0	Bacteria	Proteobacteria	Gammaproteobacteria	Pseudomonadales	Spongiibacteraceae	Spongiibacter
ASV1023	Glacial	LacAmpRUFtf-a	0,000321492	Bacteria	Proteobacteria	Alphaproteobacteria	Parvibaculales	RS24	IMCC14465
ASV1023	Baie de la Table	BdT0-2	0	Bacteria	Proteobacteria	Alphaproteobacteria	Parvibaculales	RS24	IMCC14465
ASV1023	Control	Ctr-tf-IIb	0	Bacteria	Proteobacteria	Alphaproteobacteria	Parvibaculales	RS24	IMCC14465
ASV1023	Non-glacial	LacADNRUFtfa	0	Bacteria	Proteobacteria	Alphaproteobacteria	Parvibaculales	RS24	IMCC14465
ASV1023	Non-glacial	LacADNRUFtfb	0	Bacteria	Proteobacteria	Alphaproteobacteria	Parvibaculales	RS24	IMCC14465
ASV1023	Non-glacial	LacADNRUFtfc	0	Bacteria	Proteobacteria	Alphaproteobacteria	Parvibaculales	RS24	IMCC14465
ASV1023	Glacial	LacAmpRUFtf-b	0	Bacteria	Proteobacteria	Alphaproteobacteria	Parvibaculales	RS24	IMCC14465
ASV1023	Glacial	LacAmpRUFtf-c	0	Bacteria	Proteobacteria	Alphaproteobacteria	Parvibaculales	RS24	IMCC14465
ASV1025	Baie de la Table	BdT0-2	0	Bacteria	Proteobacteria	Gammaproteobacteria	Betaproteobacteriales	Burkholderiaceae	Duganella
ASV1025	Control	Ctr-tf-IIb	0	Bacteria	Proteobacteria	Gammaproteobacteria	Betaproteobacteriales	Burkholderiaceae	Duganella
ASV1025	Non-glacial	LacADNRUFtfa	0	Bacteria	Proteobacteria	Gammaproteobacteria	Betaproteobacteriales	Burkholderiaceae	Duganella
ASV1025	Non-glacial	LacADNRUFtfb	0	Bacteria	Proteobacteria	Gammaproteobacteria	Betaproteobacteriales	Burkholderiaceae	Duganella
ASV1025	Non-glacial	LacADNRUFtfc	0	Bacteria	Proteobacteria	Gammaproteobacteria	Betaproteobacteriales	Burkholderiaceae	Duganella
ASV1025	Glacial	LacAmpRUFtf-a	0	Bacteria	Proteobacteria	Gammaproteobacteria	Betaproteobacteriales	Burkholderiaceae	Duganella
ASV1025	Glacial	LacAmpRUFtf-b	0	Bacteria	Proteobacteria	Gammaproteobacteria	Betaproteobacteriales	Burkholderiaceae	Duganella
ASV1025	Glacial	LacAmpRUFtf-c	0	Bacteria	Proteobacteria	Gammaproteobacteria	Betaproteobacteriales	Burkholderiaceae	Duganella
ASV1028	Glacial	LacAmpRUFtf-c	0,000242072	Bacteria	Proteobacteria	Alphaproteobacteria	Rhodobacterales	Rhodobacteraceae	Oceanicella
ASV1028	Baie de la Table	BdT0-2	0	Bacteria	Proteobacteria	Alphaproteobacteria	Rhodobacterales	Rhodobacteraceae	Oceanicella
ASV1028	Control	Ctr-tf-IIb	0	Bacteria	Proteobacteria	Alphaproteobacteria	Rhodobacterales	Rhodobacteraceae	Oceanicella
ASV1028	Non-glacial	LacADNRUFtfa	0	Bacteria	Proteobacteria	Alphaproteobacteria	Rhodobacterales	Rhodobacteraceae	Oceanicella
ASV1028	Non-glacial	LacADNRUFtfb	0	Bacteria	Proteobacteria	Alphaproteobacteria	Rhodobacterales	Rhodobacteraceae	Oceanicella
ASV1028	Non-glacial	LacADNRUFtfc	0	Bacteria	Proteobacteria	Alphaproteobacteria	Rhodobacterales	Rhodobacteraceae	Oceanicella
ASV1028	Glacial	LacAmpRUFtf-a	0	Bacteria	Proteobacteria	Alphaproteobacteria	Rhodobacterales	Rhodobacteraceae	Oceanicella
ASV1028	Glacial	LacAmpRUFtf-b	0	Bacteria	Proteobacteria	Alphaproteobacteria	Rhodobacterales	Rhodobacteraceae	Oceanicella
ASV1041	Baie de la Table	BdT0-2	0	Bacteria	Bacteroidota	Bacteroidia	Flavobacteriales	Flavobacteriaceae	Aquimarina
ASV1041	Control	Ctr-tf-IIb	0	Bacteria	Bacteroidota	Bacteroidia	Flavobacteriales	Flavobacteriaceae	Aquimarina

ASV1041	Non-glacial	LacADNRUFtfa	0	Bacteria	Bacteroidota	Bacteroidia	Flavobacteriales	Flavobacteriaceae	Aquimarina
ASV1041	Non-glacial	LacADNRUFtfb	0	Bacteria	Bacteroidota	Bacteroidia	Flavobacteriales	Flavobacteriaceae	Aquimarina
ASV1041	Non-glacial	LacADNRUFtfc	0	Bacteria	Bacteroidota	Bacteroidia	Flavobacteriales	Flavobacteriaceae	Aquimarina
ASV1041	Glacial	LacAmpRUFtf-a	0	Bacteria	Bacteroidota	Bacteroidia	Flavobacteriales	Flavobacteriaceae	Aquimarina
ASV1041	Glacial	LacAmpRUFtf-b	0	Bacteria	Bacteroidota	Bacteroidia	Flavobacteriales	Flavobacteriaceae	Aquimarina
ASV1041	Glacial	LacAmpRUFtf-c	0	Bacteria	Bacteroidota	Bacteroidia	Flavobacteriales	Flavobacteriaceae	Aquimarina
ASV1043	Baie de la Table	BdT0-2	0	Archaea	Crenarchaeota	Nitrososphaeria	Nitrososphaerales	Nitrosopumilaceae	Nitrosopumilus
ASV1043	Control	Ctr-tf-IIb	0	Archaea	Crenarchaeota	Nitrososphaeria	Nitrososphaerales	Nitrosopumilaceae	Nitrosopumilus
ASV1043	Non-glacial	LacADNRUFtfa	0	Archaea	Crenarchaeota	Nitrososphaeria	Nitrososphaerales	Nitrosopumilaceae	Nitrosopumilus
ASV1043	Non-glacial	LacADNRUFtfb	0	Archaea	Crenarchaeota	Nitrososphaeria	Nitrososphaerales	Nitrosopumilaceae	Nitrosopumilus
ASV1043	Non-glacial	LacADNRUFtfc	0	Archaea	Crenarchaeota	Nitrososphaeria	Nitrososphaerales	Nitrosopumilaceae	Nitrosopumilus
ASV1043	Glacial	LacAmpRUFtf-a	0	Archaea	Crenarchaeota	Nitrososphaeria	Nitrososphaerales	Nitrosopumilaceae	Nitrosopumilus
ASV1043	Glacial	LacAmpRUFtf-b	0	Archaea	Crenarchaeota	Nitrososphaeria	Nitrososphaerales	Nitrosopumilaceae	Nitrosopumilus
ASV1043	Glacial	LacAmpRUFtf-c	0	Archaea	Crenarchaeota	Nitrososphaeria	Nitrososphaerales	Nitrosopumilaceae	Nitrosopumilus
ASV1049	Baie de la Table	BdT0-2	0	Bacteria	Cyanobacteriota	Cyanobacteriia	Synechococcales_A	Cyanobiaceae	Prochlorococcus_A
ASV1049	Control	Ctr-tf-IIb	0	Bacteria	Cyanobacteriota	Cyanobacteriia	Synechococcales_A	Cyanobiaceae	Prochlorococcus_A
ASV1049	Non-glacial	LacADNRUFtfa	0	Bacteria	Cyanobacteriota	Cyanobacteriia	Synechococcales_A	Cyanobiaceae	Prochlorococcus_A
ASV1049	Non-glacial	LacADNRUFtfb	0	Bacteria	Cyanobacteriota	Cyanobacteriia	Synechococcales_A	Cyanobiaceae	Prochlorococcus_A
ASV1049	Non-glacial	LacADNRUFtfc	0	Bacteria	Cyanobacteriota	Cyanobacteriia	Synechococcales_A	Cyanobiaceae	Prochlorococcus_A
ASV1049	Glacial	LacAmpRUFtf-a	0	Bacteria	Cyanobacteriota	Cyanobacteriia	Synechococcales_A	Cyanobiaceae	Prochlorococcus_A
ASV1049	Glacial	LacAmpRUFtf-b	0	Bacteria	Cyanobacteriota	Cyanobacteriia	Synechococcales_A	Cyanobiaceae	Prochlorococcus_A
ASV1049	Glacial	LacAmpRUFtf-c	0	Bacteria	Cyanobacteriota	Cyanobacteriia	Synechococcales_A	Cyanobiaceae	Prochlorococcus_A
ASV1052	Baie de la Table	BdT0-2	0	Bacteria	Proteobacteria	Alphaproteobacteria	Rhizobiales	Hyphomicrobiaceae	Hyphomicrobium_A
ASV1052	Control	Ctr-tf-IIb	0	Bacteria	Proteobacteria	Alphaproteobacteria	Rhizobiales	Hyphomicrobiaceae	Hyphomicrobium_A
ASV1052	Non-glacial	LacADNRUFtfa	0	Bacteria	Proteobacteria	Alphaproteobacteria	Rhizobiales	Hyphomicrobiaceae	Hyphomicrobium_A
ASV1052	Non-glacial	LacADNRUFtfb	0	Bacteria	Proteobacteria	Alphaproteobacteria	Rhizobiales	Hyphomicrobiaceae	Hyphomicrobium_A
ASV1052	Non-glacial	LacADNRUFtfc	0	Bacteria	Proteobacteria	Alphaproteobacteria	Rhizobiales	Hyphomicrobiaceae	Hyphomicrobium_A
ASV1052	Glacial	LacAmpRUFtf-a	0	Bacteria	Proteobacteria	Alphaproteobacteria	Rhizobiales	Hyphomicrobiaceae	Hyphomicrobium_A
ASV1052	Glacial	LacAmpRUFtf-b	0	Bacteria	Proteobacteria	Alphaproteobacteria	Rhizobiales	Hyphomicrobiaceae	Hyphomicrobium_A
ASV1052	Glacial	LacAmpRUFtf-c	0	Bacteria	Proteobacteria	Alphaproteobacteria	Rhizobiales	Hyphomicrobiaceae	Hyphomicrobium_A
ASV1054	Glacial	LacAmpRUFtf-c	0,000484144	Bacteria	Proteobacteria	Alphaproteobacteria	Rhizobiales	Rhizobiaceae	Neorhizobium
ASV1054	Non-glacial	LacADNRUFtfb	8,43E-05	Bacteria	Proteobacteria	Alphaproteobacteria	Rhizobiales	Rhizobiaceae	Neorhizobium
ASV1054	Baie de la Table	BdT0-2	0	Bacteria	Proteobacteria	Alphaproteobacteria	Rhizobiales	Rhizobiaceae	Neorhizobium
ASV1054	Control	Ctr-tf-IIb	0	Bacteria	Proteobacteria	Alphaproteobacteria	Rhizobiales	Rhizobiaceae	Neorhizobium

ASV1054	Non-glacial	LacADNRUFtfa	0	Bacteria	Proteobacteria	Alphaproteobacteria	Rhizobiales	Rhizobiaceae	Neorhizobium
ASV1054	Non-glacial	LacADNRUFtfc	0	Bacteria	Proteobacteria	Alphaproteobacteria	Rhizobiales	Rhizobiaceae	Neorhizobium
ASV1054	Glacial	LacAmpRUFtf-a	0	Bacteria	Proteobacteria	Alphaproteobacteria	Rhizobiales	Rhizobiaceae	Neorhizobium
ASV1054	Glacial	LacAmpRUFtf-b	0	Bacteria	Proteobacteria	Alphaproteobacteria	Rhizobiales	Rhizobiaceae	Neorhizobium
ASV1059	Glacial	LacAmpRUFtf-a	0,000241119	Bacteria	Proteobacteria	Alphaproteobacteria	Rhodobacterales	Rhodobacteraceae	Pseudooceanicola_B
ASV1059	Baie de la Table	BdT0-2	0	Bacteria	Proteobacteria	Alphaproteobacteria	Rhodobacterales	Rhodobacteraceae	Pseudooceanicola_B
ASV1059	Control	Ctr-tf-IIb	0	Bacteria	Proteobacteria	Alphaproteobacteria	Rhodobacterales	Rhodobacteraceae	Pseudooceanicola_B
ASV1059	Non-glacial	LacADNRUFtfa	0	Bacteria	Proteobacteria	Alphaproteobacteria	Rhodobacterales	Rhodobacteraceae	Pseudooceanicola_B
ASV1059	Non-glacial	LacADNRUFtfb	0	Bacteria	Proteobacteria	Alphaproteobacteria	Rhodobacterales	Rhodobacteraceae	Pseudooceanicola_B
ASV1059	Non-glacial	LacADNRUFtfc	0	Bacteria	Proteobacteria	Alphaproteobacteria	Rhodobacterales	Rhodobacteraceae	Pseudooceanicola_B
ASV1059	Glacial	LacAmpRUFtf-b	0	Bacteria	Proteobacteria	Alphaproteobacteria	Rhodobacterales	Rhodobacteraceae	Pseudooceanicola_B
ASV1059	Glacial	LacAmpRUFtf-c	0	Bacteria	Proteobacteria	Alphaproteobacteria	Rhodobacterales	Rhodobacteraceae	Pseudooceanicola_B
ASV106	Baie de la Table	BdT0-2	0,002568587	Bacteria	Proteobacteria	Alphaproteobacteria	Rhizobiales	TMED25	MED-G09
ASV106	Non-glacial	LacADNRUFtfb	0,00061117	Bacteria	Proteobacteria	Alphaproteobacteria	Rhizobiales	TMED25	MED-G09
ASV106	Non-glacial	LacADNRUFtfa	0,00060049	Bacteria	Proteobacteria	Alphaproteobacteria	Rhizobiales	TMED25	MED-G09
ASV106	Control	Ctr-tf-IIb	0,00032854	Bacteria	Proteobacteria	Alphaproteobacteria	Rhizobiales	TMED25	MED-G09
ASV106	Glacial	LacAmpRUFtf-a	0,000200932	Bacteria	Proteobacteria	Alphaproteobacteria	Rhizobiales	TMED25	MED-G09
ASV106	Non-glacial	LacADNRUFtfc	0	Bacteria	Proteobacteria	Alphaproteobacteria	Rhizobiales	TMED25	MED-G09
ASV106	Glacial	LacAmpRUFtf-b	0	Bacteria	Proteobacteria	Alphaproteobacteria	Rhizobiales	TMED25	MED-G09
ASV106	Glacial	LacAmpRUFtf-c	0	Bacteria	Proteobacteria	Alphaproteobacteria	Rhizobiales	TMED25	MED-G09
ASV1060	Baie de la Table	BdT0-2	0	Bacteria	Bacteroidota	Bacteroidia	AKYH767-A	OLB10	OLB10
ASV1060	Control	Ctr-tf-IIb	0	Bacteria	Bacteroidota	Bacteroidia	AKYH767-A	OLB10	OLB10
ASV1060	Non-glacial	LacADNRUFtfa	0	Bacteria	Bacteroidota	Bacteroidia	AKYH767-A	OLB10	OLB10
ASV1060	Non-glacial	LacADNRUFtfb	0	Bacteria	Bacteroidota	Bacteroidia	AKYH767-A	OLB10	OLB10
ASV1060	Non-glacial	LacADNRUFtfc	0	Bacteria	Bacteroidota	Bacteroidia	AKYH767-A	OLB10	OLB10
ASV1060	Glacial	LacAmpRUFtf-a	0	Bacteria	Bacteroidota	Bacteroidia	AKYH767-A	OLB10	OLB10
ASV1060	Glacial	LacAmpRUFtf-b	0	Bacteria	Bacteroidota	Bacteroidia	AKYH767-A	OLB10	OLB10
ASV1060	Glacial	LacAmpRUFtf-c	0	Bacteria	Bacteroidota	Bacteroidia	AKYH767-A	OLB10	OLB10
ASV1063	Baie de la Table	BdT0-2	0	Bacteria	Proteobacteria	Gammaproteobacteria	Betaproteobacteriales	Gallionellaceae	Gallionella
ASV1063	Control	Ctr-tf-IIb	0	Bacteria	Proteobacteria	Gammaproteobacteria	Betaproteobacteriales	Gallionellaceae	Gallionella
ASV1063	Non-glacial	LacADNRUFtfa	0	Bacteria	Proteobacteria	Gammaproteobacteria	Betaproteobacteriales	Gallionellaceae	Gallionella
ASV1063	Non-glacial	LacADNRUFtfb	0	Bacteria	Proteobacteria	Gammaproteobacteria	Betaproteobacteriales	Gallionellaceae	Gallionella
ASV1063	Non-glacial	LacADNRUFtfc	0	Bacteria	Proteobacteria	Gammaproteobacteria	Betaproteobacteriales	Gallionellaceae	Gallionella
ASV1063	Glacial	LacAmpRUFtf-a	0	Bacteria	Proteobacteria	Gammaproteobacteria	Betaproteobacteriales	Gallionellaceae	Gallionella

ASV1063	Glacial	LacAmpRUFtf-b	0	Bacteria	Proteobacteria	Gammaproteobacteria	Betaproteobacteriales	Gallionellaceae	Gallionella
ASV1063	Glacial	LacAmpRUFtf-c	0	Bacteria	Proteobacteria	Gammaproteobacteria	Betaproteobacteriales	Gallionellaceae	Gallionella
ASV1064	Glacial	LacAmpRUFtf-a	0,000120559	Bacteria	Proteobacteria	Gammaproteobacteria	Betaproteobacteriales	Burkholderiaceae	Limnobacter
ASV1064	Baie de la Table	BdT0-2	0	Bacteria	Proteobacteria	Gammaproteobacteria	Betaproteobacteriales	Burkholderiaceae	Limnobacter
ASV1064	Control	Ctr-tf-IIb	0	Bacteria	Proteobacteria	Gammaproteobacteria	Betaproteobacteriales	Burkholderiaceae	Limnobacter
ASV1064	Non-glacial	LacADNRUFtfa	0	Bacteria	Proteobacteria	Gammaproteobacteria	Betaproteobacteriales	Burkholderiaceae	Limnobacter
ASV1064	Non-glacial	LacADNRUFtfb	0	Bacteria	Proteobacteria	Gammaproteobacteria	Betaproteobacteriales	Burkholderiaceae	Limnobacter
ASV1064	Non-glacial	LacADNRUFtfc	0	Bacteria	Proteobacteria	Gammaproteobacteria	Betaproteobacteriales	Burkholderiaceae	Limnobacter
ASV1064	Glacial	LacAmpRUFtf-b	0	Bacteria	Proteobacteria	Gammaproteobacteria	Betaproteobacteriales	Burkholderiaceae	Limnobacter
ASV1064	Glacial	LacAmpRUFtf-c	0	Bacteria	Proteobacteria	Gammaproteobacteria	Betaproteobacteriales	Burkholderiaceae	Limnobacter
ASV1065	Glacial	LacAmpRUFtf-b	0,001511716	Bacteria	Bacteroidota	Bacteroidia	Bacteroidales	UBA932	RC9
ASV1065	Baie de la Table	BdT0-2	0	Bacteria	Bacteroidota	Bacteroidia	Bacteroidales	UBA932	RC9
ASV1065	Control	Ctr-tf-IIb	0	Bacteria	Bacteroidota	Bacteroidia	Bacteroidales	UBA932	RC9
ASV1065	Non-glacial	LacADNRUFtfa	0	Bacteria	Bacteroidota	Bacteroidia	Bacteroidales	UBA932	RC9
ASV1065	Non-glacial	LacADNRUFtfb	0	Bacteria	Bacteroidota	Bacteroidia	Bacteroidales	UBA932	RC9
ASV1065	Non-glacial	LacADNRUFtfc	0	Bacteria	Bacteroidota	Bacteroidia	Bacteroidales	UBA932	RC9
ASV1065	Glacial	LacAmpRUFtf-a	0	Bacteria	Bacteroidota	Bacteroidia	Bacteroidales	UBA932	RC9
ASV1065	Glacial	LacAmpRUFtf-c	0	Bacteria	Bacteroidota	Bacteroidia	Bacteroidales	UBA932	RC9
ASV1066	Glacial	LacAmpRUFtf-c	0,000677802	Bacteria	Firmicutes	Bacilli	Bacillales	Bacillaceae_A	Bacillus_Y
ASV1066	Baie de la Table	BdT0-2	0	Bacteria	Firmicutes	Bacilli	Bacillales	Bacillaceae_A	Bacillus_Y
ASV1066	Control	Ctr-tf-IIb	0	Bacteria	Firmicutes	Bacilli	Bacillales	Bacillaceae_A	Bacillus_Y
ASV1066	Non-glacial	LacADNRUFtfa	0	Bacteria	Firmicutes	Bacilli	Bacillales	Bacillaceae_A	Bacillus_Y
ASV1066	Non-glacial	LacADNRUFtfb	0	Bacteria	Firmicutes	Bacilli	Bacillales	Bacillaceae_A	Bacillus_Y
ASV1066	Non-glacial	LacADNRUFtfc	0	Bacteria	Firmicutes	Bacilli	Bacillales	Bacillaceae_A	Bacillus_Y
ASV1066	Glacial	LacAmpRUFtf-a	0	Bacteria	Firmicutes	Bacilli	Bacillales	Bacillaceae_A	Bacillus_Y
ASV1066	Glacial	LacAmpRUFtf-b	0	Bacteria	Firmicutes	Bacilli	Bacillales	Bacillaceae_A	Bacillus_Y
ASV1067	Glacial	LacAmpRUFtf-c	0,000677802	Bacteria	Firmicutes	Bacilli	Bacillales	Bacillaceae_A	Bacillus_AC
ASV1067	Baie de la Table	BdT0-2	0	Bacteria	Firmicutes	Bacilli	Bacillales	Bacillaceae_A	Bacillus_AC
ASV1067	Control	Ctr-tf-IIb	0	Bacteria	Firmicutes	Bacilli	Bacillales	Bacillaceae_A	Bacillus_AC
ASV1067	Non-glacial	LacADNRUFtfa	0	Bacteria	Firmicutes	Bacilli	Bacillales	Bacillaceae_A	Bacillus_AC
ASV1067	Non-glacial	LacADNRUFtfb	0	Bacteria	Firmicutes	Bacilli	Bacillales	Bacillaceae_A	Bacillus_AC
ASV1067	Non-glacial	LacADNRUFtfc	0	Bacteria	Firmicutes	Bacilli	Bacillales	Bacillaceae_A	Bacillus_AC
ASV1067	Glacial	LacAmpRUFtf-a	0	Bacteria	Firmicutes	Bacilli	Bacillales	Bacillaceae_A	Bacillus_AC
ASV1067	Glacial	LacAmpRUFtf-b	0	Bacteria	Firmicutes	Bacilli	Bacillales	Bacillaceae_A	Bacillus_AC

ASV1068	Glacial	LacAmpRUFtf-b	0,001403736	Bacteria	Bacteroidota	Bacteroidia	Flavobacteriales	Weeksellaceae	Chryseobacterium
ASV1068	Glacial	LacAmpRUFtf-c	0,000677802	Bacteria	Bacteroidota	Bacteroidia	Flavobacteriales	Weeksellaceae	Chryseobacterium
ASV1068	Baie de la Table	BdT0-2	0	Bacteria	Bacteroidota	Bacteroidia	Flavobacteriales	Weeksellaceae	Chryseobacterium
ASV1068	Control	Ctr-tf-IIb	0	Bacteria	Bacteroidota	Bacteroidia	Flavobacteriales	Weeksellaceae	Chryseobacterium
ASV1068	Non-glacial	LacADNRUFtfa	0	Bacteria	Bacteroidota	Bacteroidia	Flavobacteriales	Weeksellaceae	Chryseobacterium
ASV1068	Non-glacial	LacADNRUFtfb	0	Bacteria	Bacteroidota	Bacteroidia	Flavobacteriales	Weeksellaceae	Chryseobacterium
ASV1068	Non-glacial	LacADNRUFtfc	0	Bacteria	Bacteroidota	Bacteroidia	Flavobacteriales	Weeksellaceae	Chryseobacterium
ASV1068	Glacial	LacAmpRUFtf-a	0	Bacteria	Bacteroidota	Bacteroidia	Flavobacteriales	Weeksellaceae	Chryseobacterium
ASV1070	Baie de la Table	BdT0-2	0	Bacteria	Proteobacteria	Gammaproteobacteria	UBA4486	UBA4486	UBA7359
ASV1070	Control	Ctr-tf-IIb	0	Bacteria	Proteobacteria	Gammaproteobacteria	UBA4486	UBA4486	UBA7359
ASV1070	Non-glacial	LacADNRUFtfa	0	Bacteria	Proteobacteria	Gammaproteobacteria	UBA4486	UBA4486	UBA7359
ASV1070	Non-glacial	LacADNRUFtfb	0	Bacteria	Proteobacteria	Gammaproteobacteria	UBA4486	UBA4486	UBA7359
ASV1070	Non-glacial	LacADNRUFtfc	0	Bacteria	Proteobacteria	Gammaproteobacteria	UBA4486	UBA4486	UBA7359
ASV1070	Glacial	LacAmpRUFtf-a	0	Bacteria	Proteobacteria	Gammaproteobacteria	UBA4486	UBA4486	UBA7359
ASV1070	Glacial	LacAmpRUFtf-b	0	Bacteria	Proteobacteria	Gammaproteobacteria	UBA4486	UBA4486	UBA7359
ASV1070	Glacial	LacAmpRUFtf-c	0	Bacteria	Proteobacteria	Gammaproteobacteria	UBA4486	UBA4486	UBA7359
ASV1078	Baie de la Table	BdT0-2	0	Bacteria	Myxococcota	UBA4248	UBA7976	UBA1532	UBA1532
ASV1078	Control	Ctr-tf-IIb	0	Bacteria	Myxococcota	UBA4248	UBA7976	UBA1532	UBA1532
ASV1078	Non-glacial	LacADNRUFtfa	0	Bacteria	Myxococcota	UBA4248	UBA7976	UBA1532	UBA1532
ASV1078	Non-glacial	LacADNRUFtfb	0	Bacteria	Myxococcota	UBA4248	UBA7976	UBA1532	UBA1532
ASV1078	Non-glacial	LacADNRUFtfc	0	Bacteria	Myxococcota	UBA4248	UBA7976	UBA1532	UBA1532
ASV1078	Glacial	LacAmpRUFtf-a	0	Bacteria	Myxococcota	UBA4248	UBA7976	UBA1532	UBA1532
ASV1078	Glacial	LacAmpRUFtf-b	0	Bacteria	Myxococcota	UBA4248	UBA7976	UBA1532	UBA1532
ASV1078	Glacial	LacAmpRUFtf-c	0	Bacteria	Myxococcota	UBA4248	UBA7976	UBA1532	UBA1532
ASV1080	Baie de la Table	BdT0-2	0	Bacteria	Proteobacteria	Alphaproteobacteria	Rhizobiales	Rhizobiaceae	Pararhizobium
ASV1080	Control	Ctr-tf-IIb	0	Bacteria	Proteobacteria	Alphaproteobacteria	Rhizobiales	Rhizobiaceae	Pararhizobium
ASV1080	Non-glacial	LacADNRUFtfa	0	Bacteria	Proteobacteria	Alphaproteobacteria	Rhizobiales	Rhizobiaceae	Pararhizobium
ASV1080	Non-glacial	LacADNRUFtfb	0	Bacteria	Proteobacteria	Alphaproteobacteria	Rhizobiales	Rhizobiaceae	Pararhizobium
ASV1080	Non-glacial	LacADNRUFtfc	0	Bacteria	Proteobacteria	Alphaproteobacteria	Rhizobiales	Rhizobiaceae	Pararhizobium
ASV1080	Glacial	LacAmpRUFtf-a	0	Bacteria	Proteobacteria	Alphaproteobacteria	Rhizobiales	Rhizobiaceae	Pararhizobium
ASV1080	Glacial	LacAmpRUFtf-b	0	Bacteria	Proteobacteria	Alphaproteobacteria	Rhizobiales	Rhizobiaceae	Pararhizobium
ASV1080	Glacial	LacAmpRUFtf-c	0	Bacteria	Proteobacteria	Alphaproteobacteria	Rhizobiales	Rhizobiaceae	Pararhizobium
ASV1081	Baie de la Table	BdT0-2	0	Bacteria	Proteobacteria	Alphaproteobacteria	Sphingomonadales	Sphingomonadaceae	Blastomonas
ASV1081	Control	Ctr-tf-IIb	0	Bacteria	Proteobacteria	Alphaproteobacteria	Sphingomonadales	Sphingomonadaceae	Blastomonas

ASV1081	Non-glacial	LacADNRUFtfa	0	Bacteria	Proteobacteria	Alphaproteobacteria	Sphingomonadales	Sphingomonadaceae	Blastomonas
ASV1081	Non-glacial	LacADNRUFtfb	0	Bacteria	Proteobacteria	Alphaproteobacteria	Sphingomonadales	Sphingomonadaceae	Blastomonas
ASV1081	Non-glacial	LacADNRUFtfc	0	Bacteria	Proteobacteria	Alphaproteobacteria	Sphingomonadales	Sphingomonadaceae	Blastomonas
ASV1081	Glacial	LacAmpRUFtf-a	0	Bacteria	Proteobacteria	Alphaproteobacteria	Sphingomonadales	Sphingomonadaceae	Blastomonas
ASV1081	Glacial	LacAmpRUFtf-b	0	Bacteria	Proteobacteria	Alphaproteobacteria	Sphingomonadales	Sphingomonadaceae	Blastomonas
ASV1081	Glacial	LacAmpRUFtf-c	0	Bacteria	Proteobacteria	Alphaproteobacteria	Sphingomonadales	Sphingomonadaceae	Blastomonas
ASV1087	Baie de la Table	BdT0-2	0	Bacteria	Proteobacteria	Alphaproteobacteria	Rhizobiales	Xanthobacteraceae	Rhodoplanes
ASV1087	Control	Ctr-tf-IIb	0	Bacteria	Proteobacteria	Alphaproteobacteria	Rhizobiales	Xanthobacteraceae	Rhodoplanes
ASV1087	Non-glacial	LacADNRUFtfa	0	Bacteria	Proteobacteria	Alphaproteobacteria	Rhizobiales	Xanthobacteraceae	Rhodoplanes
ASV1087	Non-glacial	LacADNRUFtfb	0	Bacteria	Proteobacteria	Alphaproteobacteria	Rhizobiales	Xanthobacteraceae	Rhodoplanes
ASV1087	Non-glacial	LacADNRUFtfc	0	Bacteria	Proteobacteria	Alphaproteobacteria	Rhizobiales	Xanthobacteraceae	Rhodoplanes
ASV1087	Glacial	LacAmpRUFtf-a	0	Bacteria	Proteobacteria	Alphaproteobacteria	Rhizobiales	Xanthobacteraceae	Rhodoplanes
ASV1087	Glacial	LacAmpRUFtf-b	0	Bacteria	Proteobacteria	Alphaproteobacteria	Rhizobiales	Xanthobacteraceae	Rhodoplanes
ASV1087	Glacial	LacAmpRUFtf-c	0	Bacteria	Proteobacteria	Alphaproteobacteria	Rhizobiales	Xanthobacteraceae	Rhodoplanes
ASV1089	Non-glacial	LacADNRUFtfb	0,000273973	Bacteria	Actinobacteriota	Actinobacteria	Actinomycetales	Micrococcaceae	Kocuria
ASV1089	Glacial	LacAmpRUFtf-c	0,000145243	Bacteria	Actinobacteriota	Actinobacteria	Actinomycetales	Micrococcaceae	Kocuria
ASV1089	Non-glacial	LacADNRUFtfc	7,27E-05	Bacteria	Actinobacteriota	Actinobacteria	Actinomycetales	Micrococcaceae	Kocuria
ASV1089	Baie de la Table	BdT0-2	0	Bacteria	Actinobacteriota	Actinobacteria	Actinomycetales	Micrococcaceae	Kocuria
ASV1089	Control	Ctr-tf-IIb	0	Bacteria	Actinobacteriota	Actinobacteria	Actinomycetales	Micrococcaceae	Kocuria
ASV1089	Non-glacial	LacADNRUFtfa	0	Bacteria	Actinobacteriota	Actinobacteria	Actinomycetales	Micrococcaceae	Kocuria
ASV1089	Glacial	LacAmpRUFtf-a	0	Bacteria	Actinobacteriota	Actinobacteria	Actinomycetales	Micrococcaceae	Kocuria
ASV1089	Glacial	LacAmpRUFtf-b	0	Bacteria	Actinobacteriota	Actinobacteria	Actinomycetales	Micrococcaceae	Kocuria
ASV1092	Baie de la Table	BdT0-2	0	Bacteria	Proteobacteria	Gammaproteobacteria	Enterobacterales	Alteromonadaceae	Thalassotalea
ASV1092	Control	Ctr-tf-IIb	0	Bacteria	Proteobacteria	Gammaproteobacteria	Enterobacterales	Alteromonadaceae	Thalassotalea
ASV1092	Non-glacial	LacADNRUFtfa	0	Bacteria	Proteobacteria	Gammaproteobacteria	Enterobacterales	Alteromonadaceae	Thalassotalea
ASV1092	Non-glacial	LacADNRUFtfb	0	Bacteria	Proteobacteria	Gammaproteobacteria	Enterobacterales	Alteromonadaceae	Thalassotalea
ASV1092	Non-glacial	LacADNRUFtfc	0	Bacteria	Proteobacteria	Gammaproteobacteria	Enterobacterales	Alteromonadaceae	Thalassotalea
ASV1092	Glacial	LacAmpRUFtf-a	0	Bacteria	Proteobacteria	Gammaproteobacteria	Enterobacterales	Alteromonadaceae	Thalassotalea
ASV1092	Glacial	LacAmpRUFtf-b	0	Bacteria	Proteobacteria	Gammaproteobacteria	Enterobacterales	Alteromonadaceae	Thalassotalea
ASV1092	Glacial	LacAmpRUFtf-c	0	Bacteria	Proteobacteria	Gammaproteobacteria	Enterobacterales	Alteromonadaceae	Thalassotalea
ASV1093	Glacial	LacAmpRUFtf-a	0,000200932	Bacteria	Proteobacteria	Gammaproteobacteria	Enterobacterales	Vibrionaceae	Grimontia
ASV1093	Baie de la Table	BdT0-2	0	Bacteria	Proteobacteria	Gammaproteobacteria	Enterobacterales	Vibrionaceae	Grimontia
ASV1093	Control	Ctr-tf-IIb	0	Bacteria	Proteobacteria	Gammaproteobacteria	Enterobacterales	Vibrionaceae	Grimontia
ASV1093	Non-glacial	LacADNRUFtfa	0	Bacteria	Proteobacteria	Gammaproteobacteria	Enterobacterales	Vibrionaceae	Grimontia

ASV1093	Non-glacial	LacADNRUFtfb	0	Bacteria	Proteobacteria	Gammaproteobacteria	Enterobacterales	Vibrionaceae	Grimontia
ASV1093	Non-glacial	LacADNRUFtfc	0	Bacteria	Proteobacteria	Gammaproteobacteria	Enterobacterales	Vibrionaceae	Grimontia
ASV1093	Glacial	LacAmpRUFtf-b	0	Bacteria	Proteobacteria	Gammaproteobacteria	Enterobacterales	Vibrionaceae	Grimontia
ASV1093	Glacial	LacAmpRUFtf-c	0	Bacteria	Proteobacteria	Gammaproteobacteria	Enterobacterales	Vibrionaceae	Grimontia
ASV1095	Glacial	LacAmpRUFtf-a	0,000522424	Bacteria	Proteobacteria	Gammaproteobacteria	Thiotrichales	Thiotrichaceae	Leucothrix
ASV1095	Non-glacial	LacADNRUFtfc	0,000121242	Bacteria	Proteobacteria	Gammaproteobacteria	Thiotrichales	Thiotrichaceae	Leucothrix
ASV1095	Baie de la Table	BdT0-2	0	Bacteria	Proteobacteria	Gammaproteobacteria	Thiotrichales	Thiotrichaceae	Leucothrix
ASV1095	Control	Ctr-tf-IIb	0	Bacteria	Proteobacteria	Gammaproteobacteria	Thiotrichales	Thiotrichaceae	Leucothrix
ASV1095	Non-glacial	LacADNRUFtfa	0	Bacteria	Proteobacteria	Gammaproteobacteria	Thiotrichales	Thiotrichaceae	Leucothrix
ASV1095	Non-glacial	LacADNRUFtfb	0	Bacteria	Proteobacteria	Gammaproteobacteria	Thiotrichales	Thiotrichaceae	Leucothrix
ASV1095	Glacial	LacAmpRUFtf-b	0	Bacteria	Proteobacteria	Gammaproteobacteria	Thiotrichales	Thiotrichaceae	Leucothrix
ASV1095	Glacial	LacAmpRUFtf-c	0	Bacteria	Proteobacteria	Gammaproteobacteria	Thiotrichales	Thiotrichaceae	Leucothrix
ASV1098	Glacial	LacAmpRUFtf-c	0,000629388	Bacteria	Proteobacteria	Gammaproteobacteria	Enterobacterales	Enterobacteriaceae	Enterobacter
ASV1098	Non-glacial	LacADNRUFtfc	0,000193986	Bacteria	Proteobacteria	Gammaproteobacteria	Enterobacterales	Enterobacteriaceae	Enterobacter
ASV1098	Baie de la Table	BdT0-2	0	Bacteria	Proteobacteria	Gammaproteobacteria	Enterobacterales	Enterobacteriaceae	Enterobacter
ASV1098	Control	Ctr-tf-IIb	0	Bacteria	Proteobacteria	Gammaproteobacteria	Enterobacterales	Enterobacteriaceae	Enterobacter
ASV1098	Non-glacial	LacADNRUFtfa	0	Bacteria	Proteobacteria	Gammaproteobacteria	Enterobacterales	Enterobacteriaceae	Enterobacter
ASV1098	Non-glacial	LacADNRUFtfb	0	Bacteria	Proteobacteria	Gammaproteobacteria	Enterobacterales	Enterobacteriaceae	Enterobacter
ASV1098	Glacial	LacAmpRUFtf-a	0	Bacteria	Proteobacteria	Gammaproteobacteria	Enterobacterales	Enterobacteriaceae	Enterobacter
ASV1098	Glacial	LacAmpRUFtf-b	0	Bacteria	Proteobacteria	Gammaproteobacteria	Enterobacterales	Enterobacteriaceae	Enterobacter
ASV1105	Baie de la Table	BdT0-2	0	Bacteria	Verrucomicrobio	Verrucomicrobiae	Pedosphaerales	AAA164-E04	UBA7971
ASV1105	Control	Ctr-tf-IIb	0	Bacteria	Verrucomicrobio	Verrucomicrobiae	Pedosphaerales	AAA164-E04	UBA7971
ASV1105	Non-glacial	LacADNRUFtfa	0	Bacteria	Verrucomicrobio	Verrucomicrobiae	Pedosphaerales	AAA164-E04	UBA7971
ASV1105	Non-glacial	LacADNRUFtfb	0	Bacteria	Verrucomicrobio	Verrucomicrobiae	Pedosphaerales	AAA164-E04	UBA7971
ASV1105	Non-glacial	LacADNRUFtfc	0	Bacteria	Verrucomicrobio	Verrucomicrobiae	Pedosphaerales	AAA164-E04	UBA7971
ASV1105	Glacial	LacAmpRUFtf-a	0	Bacteria	Verrucomicrobio	Verrucomicrobiae	Pedosphaerales	AAA164-E04	UBA7971
ASV1105	Glacial	LacAmpRUFtf-b	0	Bacteria	Verrucomicrobio	Verrucomicrobiae	Pedosphaerales	AAA164-E04	UBA7971
ASV1105	Glacial	LacAmpRUFtf-c	0	Bacteria	Verrucomicrobio	Verrucomicrobiae	Pedosphaerales	AAA164-E04	UBA7971
ASV111	Baie de la Table	BdT0-2	0,002295333	Bacteria	Bacteroidota	Bacteroidia	Flavobacteriales	UBA7430	GCA-2692065
ASV111	Non-glacial	LacADNRUFtfa	0,001431937	Bacteria	Bacteroidota	Bacteroidia	Flavobacteriales	UBA7430	GCA-2692065
ASV111	Non-glacial	LacADNRUFtfb	0,000969442	Bacteria	Bacteroidota	Bacteroidia	Flavobacteriales	UBA7430	GCA-2692065
ASV111	Glacial	LacAmpRUFtf-b	0,000539898	Bacteria	Bacteroidota	Bacteroidia	Flavobacteriales	UBA7430	GCA-2692065
ASV111	Glacial	LacAmpRUFtf-c	0,000484144	Bacteria	Bacteroidota	Bacteroidia	Flavobacteriales	UBA7430	GCA-2692065
ASV111	Control	Ctr-tf-IIb	0,000348573	Bacteria	Bacteroidota	Bacteroidia	Flavobacteriales	UBA7430	GCA-2692065

ASV111	Non-glacial	LacADNRUFtfc	0,000218235	Bacteria	Bacteroidota	Bacteroidia	Flavobacteriales	UBA7430	GCA-2692065
ASV111	Glacial	LacAmpRUFtf-a	0	Bacteria	Bacteroidota	Bacteroidia	Flavobacteriales	UBA7430	GCA-2692065
ASV1113	Glacial	LacAmpRUFtf-a	0,000361678	Bacteria	Proteobacteria	Gammaproteobacteria	Pseudomonadales	Nitrincolaceae	Amphritea
ASV1113	Baie de la Table	BdT0-2	0	Bacteria	Proteobacteria	Gammaproteobacteria	Pseudomonadales	Nitrincolaceae	Amphritea
ASV1113	Control	Ctr-tf-IIb	0	Bacteria	Proteobacteria	Gammaproteobacteria	Pseudomonadales	Nitrincolaceae	Amphritea
ASV1113	Non-glacial	LacADNRUFtfa	0	Bacteria	Proteobacteria	Gammaproteobacteria	Pseudomonadales	Nitrincolaceae	Amphritea
ASV1113	Non-glacial	LacADNRUFtfb	0	Bacteria	Proteobacteria	Gammaproteobacteria	Pseudomonadales	Nitrincolaceae	Amphritea
ASV1113	Non-glacial	LacADNRUFtfc	0	Bacteria	Proteobacteria	Gammaproteobacteria	Pseudomonadales	Nitrincolaceae	Amphritea
ASV1113	Glacial	LacAmpRUFtf-b	0	Bacteria	Proteobacteria	Gammaproteobacteria	Pseudomonadales	Nitrincolaceae	Amphritea
ASV1113	Glacial	LacAmpRUFtf-c	0	Bacteria	Proteobacteria	Gammaproteobacteria	Pseudomonadales	Nitrincolaceae	Amphritea
ASV112	Glacial	LacAmpRUFtf-c	0,001452433	Bacteria	Proteobacteria	Gammaproteobacteria	Enterobacterales	Enterobacteriaceae	Escherichia
ASV112	Baie de la Table	BdT0-2	0	Bacteria	Proteobacteria	Gammaproteobacteria	Enterobacterales	Enterobacteriaceae	Escherichia
ASV112	Control	Ctr-tf-IIb	0	Bacteria	Proteobacteria	Gammaproteobacteria	Enterobacterales	Enterobacteriaceae	Escherichia
ASV112	Non-glacial	LacADNRUFtfa	0	Bacteria	Proteobacteria	Gammaproteobacteria	Enterobacterales	Enterobacteriaceae	Escherichia
ASV112	Non-glacial	LacADNRUFtfb	0	Bacteria	Proteobacteria	Gammaproteobacteria	Enterobacterales	Enterobacteriaceae	Escherichia
ASV112	Non-glacial	LacADNRUFtfc	0	Bacteria	Proteobacteria	Gammaproteobacteria	Enterobacterales	Enterobacteriaceae	Escherichia
ASV112	Glacial	LacAmpRUFtf-a	0	Bacteria	Proteobacteria	Gammaproteobacteria	Enterobacterales	Enterobacteriaceae	Escherichia
ASV112	Glacial	LacAmpRUFtf-b	0	Bacteria	Proteobacteria	Gammaproteobacteria	Enterobacterales	Enterobacteriaceae	Escherichia
ASV1120	Baie de la Table	BdT0-2	0	Bacteria	Cyanobacteriota	Cyanobacteriia	Eurycoccales	PCC-7336	PCC-7336
ASV1120	Control	Ctr-tf-IIb	0	Bacteria	Cyanobacteriota	Cyanobacteriia	Eurycoccales	PCC-7336	PCC-7336
ASV1120	Non-glacial	LacADNRUFtfa	0	Bacteria	Cyanobacteriota	Cyanobacteriia	Eurycoccales	PCC-7336	PCC-7336
ASV1120	Non-glacial	LacADNRUFtfb	0	Bacteria	Cyanobacteriota	Cyanobacteriia	Eurycoccales	PCC-7336	PCC-7336
ASV1120	Non-glacial	LacADNRUFtfc	0	Bacteria	Cyanobacteriota	Cyanobacteriia	Eurycoccales	PCC-7336	PCC-7336
ASV1120	Glacial	LacAmpRUFtf-a	0	Bacteria	Cyanobacteriota	Cyanobacteriia	Eurycoccales	PCC-7336	PCC-7336
ASV1120	Glacial	LacAmpRUFtf-b	0	Bacteria	Cyanobacteriota	Cyanobacteriia	Eurycoccales	PCC-7336	PCC-7336
ASV1120	Glacial	LacAmpRUFtf-c	0	Bacteria	Cyanobacteriota	Cyanobacteriia	Eurycoccales	PCC-7336	PCC-7336
ASV1121	Non-glacial	LacADNRUFtfb	0,000252898	Bacteria	Firmicutes	Bacilli	Lactobacillales	Carnobacteriaceae	Alloiococcus
ASV1121	Baie de la Table	BdT0-2	0	Bacteria	Firmicutes	Bacilli	Lactobacillales	Carnobacteriaceae	Alloiococcus
ASV1121	Control	Ctr-tf-IIb	0	Bacteria	Firmicutes	Bacilli	Lactobacillales	Carnobacteriaceae	Alloiococcus
ASV1121	Non-glacial	LacADNRUFtfa	0	Bacteria	Firmicutes	Bacilli	Lactobacillales	Carnobacteriaceae	Alloiococcus
ASV1121	Non-glacial	LacADNRUFtfc	0	Bacteria	Firmicutes	Bacilli	Lactobacillales	Carnobacteriaceae	Alloiococcus
ASV1121	Glacial	LacAmpRUFtf-a	0	Bacteria	Firmicutes	Bacilli	Lactobacillales	Carnobacteriaceae	Alloiococcus
ASV1121	Glacial	LacAmpRUFtf-b	0	Bacteria	Firmicutes	Bacilli	Lactobacillales	Carnobacteriaceae	Alloiococcus
ASV1121	Glacial	LacAmpRUFtf-c	0	Bacteria	Firmicutes	Bacilli	Lactobacillales	Carnobacteriaceae	Alloiococcus

ASV1129	Baie de la Table	BdT0-2	0	Bacteria	Proteobacteria	Alphaproteobacteria	Rhodobacterales	Rhodobacteraceae	Pseudorhodobacter_A
ASV1129	Control	Ctr-tf-IIb	0	Bacteria	Proteobacteria	Alphaproteobacteria	Rhodobacterales	Rhodobacteraceae	Pseudorhodobacter_A
ASV1129	Non-glacial	LacADNRUFtfa	0	Bacteria	Proteobacteria	Alphaproteobacteria	Rhodobacterales	Rhodobacteraceae	Pseudorhodobacter_A
ASV1129	Non-glacial	LacADNRUFtfb	0	Bacteria	Proteobacteria	Alphaproteobacteria	Rhodobacterales	Rhodobacteraceae	Pseudorhodobacter_A
ASV1129	Non-glacial	LacADNRUFtfc	0	Bacteria	Proteobacteria	Alphaproteobacteria	Rhodobacterales	Rhodobacteraceae	Pseudorhodobacter_A
ASV1129	Glacial	LacAmpRUFtf-a	0	Bacteria	Proteobacteria	Alphaproteobacteria	Rhodobacterales	Rhodobacteraceae	Pseudorhodobacter_A
ASV1129	Glacial	LacAmpRUFtf-b	0	Bacteria	Proteobacteria	Alphaproteobacteria	Rhodobacterales	Rhodobacteraceae	Pseudorhodobacter_A
ASV1129	Glacial	LacAmpRUFtf-c	0	Bacteria	Proteobacteria	Alphaproteobacteria	Rhodobacterales	Rhodobacteraceae	Pseudorhodobacter_A
ASV1133	Glacial	LacAmpRUFtf-c	0,000580973	Bacteria	Firmicutes_A	Clostridia	Oscillospirales	Oscillospiraceae	Oscillibacter
ASV1133	Baie de la Table	BdT0-2	0	Bacteria	Firmicutes_A	Clostridia	Oscillospirales	Oscillospiraceae	Oscillibacter
ASV1133	Control	Ctr-tf-IIb	0	Bacteria	Firmicutes_A	Clostridia	Oscillospirales	Oscillospiraceae	Oscillibacter
ASV1133	Non-glacial	LacADNRUFtfa	0	Bacteria	Firmicutes_A	Clostridia	Oscillospirales	Oscillospiraceae	Oscillibacter
ASV1133	Non-glacial	LacADNRUFtfb	0	Bacteria	Firmicutes_A	Clostridia	Oscillospirales	Oscillospiraceae	Oscillibacter
ASV1133	Non-glacial	LacADNRUFtfc	0	Bacteria	Firmicutes_A	Clostridia	Oscillospirales	Oscillospiraceae	Oscillibacter
ASV1133	Glacial	LacAmpRUFtf-a	0	Bacteria	Firmicutes_A	Clostridia	Oscillospirales	Oscillospiraceae	Oscillibacter
ASV1133	Glacial	LacAmpRUFtf-b	0	Bacteria	Firmicutes_A	Clostridia	Oscillospirales	Oscillospiraceae	Oscillibacter
ASV1134	Glacial	LacAmpRUFtf-c	0,000580973	Bacteria	Bacteroidota	Bacteroidia	Flavobacteriales	Flavobacteriaceae	Gillisia
ASV1134	Baie de la Table	BdT0-2	0	Bacteria	Bacteroidota	Bacteroidia	Flavobacteriales	Flavobacteriaceae	Gillisia
ASV1134	Control	Ctr-tf-IIb	0	Bacteria	Bacteroidota	Bacteroidia	Flavobacteriales	Flavobacteriaceae	Gillisia
ASV1134	Non-glacial	LacADNRUFtfa	0	Bacteria	Bacteroidota	Bacteroidia	Flavobacteriales	Flavobacteriaceae	Gillisia
ASV1134	Non-glacial	LacADNRUFtfb	0	Bacteria	Bacteroidota	Bacteroidia	Flavobacteriales	Flavobacteriaceae	Gillisia
ASV1134	Non-glacial	LacADNRUFtfc	0	Bacteria	Bacteroidota	Bacteroidia	Flavobacteriales	Flavobacteriaceae	Gillisia
ASV1134	Glacial	LacAmpRUFtf-a	0	Bacteria	Bacteroidota	Bacteroidia	Flavobacteriales	Flavobacteriaceae	Gillisia
ASV1134	Glacial	LacAmpRUFtf-b	0	Bacteria	Bacteroidota	Bacteroidia	Flavobacteriales	Flavobacteriaceae	Gillisia
ASV1135	Glacial	LacAmpRUFtf-c	0,000580973	Bacteria	Firmicutes_A	Clostridia	Lachnospirales	Lachnospiraceae	Coprococcus
ASV1135	Baie de la Table	BdT0-2	0	Bacteria	Firmicutes_A	Clostridia	Lachnospirales	Lachnospiraceae	Coprococcus
ASV1135	Control	Ctr-tf-IIb	0	Bacteria	Firmicutes_A	Clostridia	Lachnospirales	Lachnospiraceae	Coprococcus
ASV1135	Non-glacial	LacADNRUFtfa	0	Bacteria	Firmicutes_A	Clostridia	Lachnospirales	Lachnospiraceae	Coprococcus
ASV1135	Non-glacial	LacADNRUFtfb	0	Bacteria	Firmicutes_A	Clostridia	Lachnospirales	Lachnospiraceae	Coprococcus
ASV1135	Non-glacial	LacADNRUFtfc	0	Bacteria	Firmicutes_A	Clostridia	Lachnospirales	Lachnospiraceae	Coprococcus
ASV1135	Glacial	LacAmpRUFtf-a	0	Bacteria	Firmicutes_A	Clostridia	Lachnospirales	Lachnospiraceae	Coprococcus
ASV1135	Glacial	LacAmpRUFtf-b	0	Bacteria	Firmicutes_A	Clostridia	Lachnospirales	Lachnospiraceae	Coprococcus
ASV1136	Glacial	LacAmpRUFtf-c	0,000580973	Bacteria	Myxococcota	Polyangia	Polyangiales	Polyangiaceae	Labilithrix
ASV1136	Baie de la Table	BdT0-2	0	Bacteria	Myxococcota	Polyangia	Polyangiales	Polyangiaceae	Labilithrix

ASV1136	Control	Ctr-tf-IIb	0	Bacteria	Myxococcota	Polyangia	Polyangiales	Polyangiaceae	Labilithrix
ASV1136	Non-glacial	LacADNRUFtfa	0	Bacteria	Myxococcota	Polyangia	Polyangiales	Polyangiaceae	Labilithrix
ASV1136	Non-glacial	LacADNRUFtfb	0	Bacteria	Myxococcota	Polyangia	Polyangiales	Polyangiaceae	Labilithrix
ASV1136	Non-glacial	LacADNRUFtfc	0	Bacteria	Myxococcota	Polyangia	Polyangiales	Polyangiaceae	Labilithrix
ASV1136	Glacial	LacAmpRUFtf-a	0	Bacteria	Myxococcota	Polyangia	Polyangiales	Polyangiaceae	Labilithrix
ASV1136	Glacial	LacAmpRUFtf-b	0	Bacteria	Myxococcota	Polyangia	Polyangiales	Polyangiaceae	Labilithrix
ASV114	Baie de la Table	BdT0-2	0,002240682	Bacteria	Proteobacteria	Alphaproteobacteria	Sphingomonadales	Emcibacteraceae	Emcibacter
ASV114	Non-glacial	LacADNRUFtfb	0,001412013	Bacteria	Proteobacteria	Alphaproteobacteria	Sphingomonadales	Emcibacteraceae	Emcibacter
ASV114	Non-glacial	LacADNRUFtfa	0,001062405	Bacteria	Proteobacteria	Alphaproteobacteria	Sphingomonadales	Emcibacteraceae	Emcibacter
ASV114	Control	Ctr-tf-IIb	0,000408672	Bacteria	Proteobacteria	Alphaproteobacteria	Sphingomonadales	Emcibacteraceae	Emcibacter
ASV114	Non-glacial	LacADNRUFtfc	0,000218235	Bacteria	Proteobacteria	Alphaproteobacteria	Sphingomonadales	Emcibacteraceae	Emcibacter
ASV114	Glacial	LacAmpRUFtf-a	0,000120559	Bacteria	Proteobacteria	Alphaproteobacteria	Sphingomonadales	Emcibacteraceae	Emcibacter
ASV114	Glacial	LacAmpRUFtf-b	0	Bacteria	Proteobacteria	Alphaproteobacteria	Sphingomonadales	Emcibacteraceae	Emcibacter
ASV114	Glacial	LacAmpRUFtf-c	0	Bacteria	Proteobacteria	Alphaproteobacteria	Sphingomonadales	Emcibacteraceae	Emcibacter
ASV1144	Control	Ctr-tf-IIb	4,41E-05	Bacteria	Proteobacteria	Alphaproteobacteria	Rhodobacterales	Rhodobacteraceae	Monaibacterium
ASV1144	Baie de la Table	BdT0-2	0	Bacteria	Proteobacteria	Alphaproteobacteria	Rhodobacterales	Rhodobacteraceae	Monaibacterium
ASV1144	Non-glacial	LacADNRUFtfa	0	Bacteria	Proteobacteria	Alphaproteobacteria	Rhodobacterales	Rhodobacteraceae	Monaibacterium
ASV1144	Non-glacial	LacADNRUFtfb	0	Bacteria	Proteobacteria	Alphaproteobacteria	Rhodobacterales	Rhodobacteraceae	Monaibacterium
ASV1144	Non-glacial	LacADNRUFtfc	0	Bacteria	Proteobacteria	Alphaproteobacteria	Rhodobacterales	Rhodobacteraceae	Monaibacterium
ASV1144	Glacial	LacAmpRUFtf-a	0	Bacteria	Proteobacteria	Alphaproteobacteria	Rhodobacterales	Rhodobacteraceae	Monaibacterium
ASV1144	Glacial	LacAmpRUFtf-b	0	Bacteria	Proteobacteria	Alphaproteobacteria	Rhodobacterales	Rhodobacteraceae	Monaibacterium
ASV1144	Glacial	LacAmpRUFtf-c	0	Bacteria	Proteobacteria	Alphaproteobacteria	Rhodobacterales	Rhodobacteraceae	Monaibacterium
ASV1154	Non-glacial	LacADNRUFtfb	0,000231823	Bacteria	Bacteroidota	Bacteroidia	Flavobacteriales	Flavobacteriaceae	Wenyingzhuangia
ASV1154	Baie de la Table	BdT0-2	0	Bacteria	Bacteroidota	Bacteroidia	Flavobacteriales	Flavobacteriaceae	Wenyingzhuangia
ASV1154	Control	Ctr-tf-IIb	0	Bacteria	Bacteroidota	Bacteroidia	Flavobacteriales	Flavobacteriaceae	Wenyingzhuangia
ASV1154	Non-glacial	LacADNRUFtfa	0	Bacteria	Bacteroidota	Bacteroidia	Flavobacteriales	Flavobacteriaceae	Wenyingzhuangia
ASV1154	Non-glacial	LacADNRUFtfc	0	Bacteria	Bacteroidota	Bacteroidia	Flavobacteriales	Flavobacteriaceae	Wenyingzhuangia
ASV1154	Glacial	LacAmpRUFtf-a	0	Bacteria	Bacteroidota	Bacteroidia	Flavobacteriales	Flavobacteriaceae	Wenyingzhuangia
ASV1154	Glacial	LacAmpRUFtf-b	0	Bacteria	Bacteroidota	Bacteroidia	Flavobacteriales	Flavobacteriaceae	Wenyingzhuangia
ASV1154	Glacial	LacAmpRUFtf-c	0	Bacteria	Bacteroidota	Bacteroidia	Flavobacteriales	Flavobacteriaceae	Wenyingzhuangia
ASV1158	Glacial	LacAmpRUFtf-c	0,000387315	Bacteria	Firmicutes	Bacilli	Staphylococcales	Gemellaceae	Gemella_A
ASV1158	Baie de la Table	BdT0-2	0	Bacteria	Firmicutes	Bacilli	Staphylococcales	Gemellaceae	Gemella_A
ASV1158	Control	Ctr-tf-IIb	0	Bacteria	Firmicutes	Bacilli	Staphylococcales	Gemellaceae	Gemella_A
ASV1158	Non-glacial	LacADNRUFtfa	0	Bacteria	Firmicutes	Bacilli	Staphylococcales	Gemellaceae	Gemella_A

ASV1158	Non-glacial	LacADNRUFtfb	0	Bacteria	Firmicutes	Bacilli	Staphylococcales	Gemellaceae	Gemella_A
ASV1158	Non-glacial	LacADNRUFtfc	0	Bacteria	Firmicutes	Bacilli	Staphylococcales	Gemellaceae	Gemella_A
ASV1158	Glacial	LacAmpRUFtf-a	0	Bacteria	Firmicutes	Bacilli	Staphylococcales	Gemellaceae	Gemella_A
ASV1158	Glacial	LacAmpRUFtf-b	0	Bacteria	Firmicutes	Bacilli	Staphylococcales	Gemellaceae	Gemella_A
ASV1170	Baie de la Table	BdT0-2	0	Bacteria	Chloroflexota	Dehalococcoidia	UBA2979	GCA-002718395	GCA-002718395
ASV1170	Control	Ctr-tf-IIb	0	Bacteria	Chloroflexota	Dehalococcoidia	UBA2979	GCA-002718395	GCA-002718395
ASV1170	Non-glacial	LacADNRUFtfa	0	Bacteria	Chloroflexota	Dehalococcoidia	UBA2979	GCA-002718395	GCA-002718395
ASV1170	Non-glacial	LacADNRUFtfb	0	Bacteria	Chloroflexota	Dehalococcoidia	UBA2979	GCA-002718395	GCA-002718395
ASV1170	Non-glacial	LacADNRUFtfc	0	Bacteria	Chloroflexota	Dehalococcoidia	UBA2979	GCA-002718395	GCA-002718395
ASV1170	Glacial	LacAmpRUFtf-a	0	Bacteria	Chloroflexota	Dehalococcoidia	UBA2979	GCA-002718395	GCA-002718395
ASV1170	Glacial	LacAmpRUFtf-b	0	Bacteria	Chloroflexota	Dehalococcoidia	UBA2979	GCA-002718395	GCA-002718395
ASV1170	Glacial	LacAmpRUFtf-c	0	Bacteria	Chloroflexota	Dehalococcoidia	UBA2979	GCA-002718395	GCA-002718395
ASV1176	Glacial	LacAmpRUFtf-c	0,000532559	Bacteria	Actinobacteriota	Actinobacteria	Actinomycetales	Dermatophilaceae	Janibacter
ASV1176	Baie de la Table	BdT0-2	0	Bacteria	Actinobacteriota	Actinobacteria	Actinomycetales	Dermatophilaceae	Janibacter
ASV1176	Control	Ctr-tf-IIb	0	Bacteria	Actinobacteriota	Actinobacteria	Actinomycetales	Dermatophilaceae	Janibacter
ASV1176	Non-glacial	LacADNRUFtfa	0	Bacteria	Actinobacteriota	Actinobacteria	Actinomycetales	Dermatophilaceae	Janibacter
ASV1176	Non-glacial	LacADNRUFtfb	0	Bacteria	Actinobacteriota	Actinobacteria	Actinomycetales	Dermatophilaceae	Janibacter
ASV1176	Non-glacial	LacADNRUFtfc	0	Bacteria	Actinobacteriota	Actinobacteria	Actinomycetales	Dermatophilaceae	Janibacter
ASV1176	Glacial	LacAmpRUFtf-a	0	Bacteria	Actinobacteriota	Actinobacteria	Actinomycetales	Dermatophilaceae	Janibacter
ASV1176	Glacial	LacAmpRUFtf-b	0	Bacteria	Actinobacteriota	Actinobacteria	Actinomycetales	Dermatophilaceae	Janibacter
ASV1180	Baie de la Table	BdT0-2	0	Bacteria	Bacteroidota	Bacteroidia	Chitinophagales	Chitinophagaceae	UBA1931
ASV1180	Control	Ctr-tf-IIb	0	Bacteria	Bacteroidota	Bacteroidia	Chitinophagales	Chitinophagaceae	UBA1931
ASV1180	Non-glacial	LacADNRUFtfa	0	Bacteria	Bacteroidota	Bacteroidia	Chitinophagales	Chitinophagaceae	UBA1931
ASV1180	Non-glacial	LacADNRUFtfb	0	Bacteria	Bacteroidota	Bacteroidia	Chitinophagales	Chitinophagaceae	UBA1931
ASV1180	Non-glacial	LacADNRUFtfc	0	Bacteria	Bacteroidota	Bacteroidia	Chitinophagales	Chitinophagaceae	UBA1931
ASV1180	Glacial	LacAmpRUFtf-a	0	Bacteria	Bacteroidota	Bacteroidia	Chitinophagales	Chitinophagaceae	UBA1931
ASV1180	Glacial	LacAmpRUFtf-b	0	Bacteria	Bacteroidota	Bacteroidia	Chitinophagales	Chitinophagaceae	UBA1931
ASV1180	Glacial	LacAmpRUFtf-c	0	Bacteria	Bacteroidota	Bacteroidia	Chitinophagales	Chitinophagaceae	UBA1931
ASV1188	Baie de la Table	BdT0-2	0	Bacteria	Proteobacteria	Alphaproteobacteria	Rickettsiales	Rickettsiaceae	GCF-002259525
ASV1188	Control	Ctr-tf-IIb	0	Bacteria	Proteobacteria	Alphaproteobacteria	Rickettsiales	Rickettsiaceae	GCF-002259525
ASV1188	Non-glacial	LacADNRUFtfa	0	Bacteria	Proteobacteria	Alphaproteobacteria	Rickettsiales	Rickettsiaceae	GCF-002259525
ASV1188	Non-glacial	LacADNRUFtfb	0	Bacteria	Proteobacteria	Alphaproteobacteria	Rickettsiales	Rickettsiaceae	GCF-002259525
ASV1188	Non-glacial	LacADNRUFtfc	0	Bacteria	Proteobacteria	Alphaproteobacteria	Rickettsiales	Rickettsiaceae	GCF-002259525
ASV1188	Glacial	LacAmpRUFtf-a	0	Bacteria	Proteobacteria	Alphaproteobacteria	Rickettsiales	Rickettsiaceae	GCF-002259525

ASV1188	Glacial	LacAmpRUFtf-b	0	Bacteria	Proteobacteria	Alphaproteobacteria	Rickettsiales	Rickettsiaceae	GCF-002259525
ASV1188	Glacial	LacAmpRUFtf-c	0	Bacteria	Proteobacteria	Alphaproteobacteria	Rickettsiales	Rickettsiaceae	GCF-002259525
ASV1197	Glacial	LacAmpRUFtf-b	0,000755858	Bacteria	Proteobacteria	Alphaproteobacteria	Rhizobiales	Rhizobiaceae	Allorhizobium
ASV1197	Non-glacial	LacADNRUFtfa	0,000138575	Bacteria	Proteobacteria	Alphaproteobacteria	Rhizobiales	Rhizobiaceae	Allorhizobium
ASV1197	Baie de la Table	BdT0-2	0	Bacteria	Proteobacteria	Alphaproteobacteria	Rhizobiales	Rhizobiaceae	Allorhizobium
ASV1197	Control	Ctr-tf-IIb	0	Bacteria	Proteobacteria	Alphaproteobacteria	Rhizobiales	Rhizobiaceae	Allorhizobium
ASV1197	Non-glacial	LacADNRUFtfb	0	Bacteria	Proteobacteria	Alphaproteobacteria	Rhizobiales	Rhizobiaceae	Allorhizobium
ASV1197	Non-glacial	LacADNRUFtfc	0	Bacteria	Proteobacteria	Alphaproteobacteria	Rhizobiales	Rhizobiaceae	Allorhizobium
ASV1197	Glacial	LacAmpRUFtf-a	0	Bacteria	Proteobacteria	Alphaproteobacteria	Rhizobiales	Rhizobiaceae	Allorhizobium
ASV1197	Glacial	LacAmpRUFtf-c	0	Bacteria	Proteobacteria	Alphaproteobacteria	Rhizobiales	Rhizobiaceae	Allorhizobium
ASV1198	Non-glacial	LacADNRUFtfb	0,000210748	Bacteria	Firmicutes	Bacilli	Bacillales	Bacillaceae	Bacillus_P
ASV1198	Baie de la Table	BdT0-2	0	Bacteria	Firmicutes	Bacilli	Bacillales	Bacillaceae	Bacillus_P
ASV1198	Control	Ctr-tf-IIb	0	Bacteria	Firmicutes	Bacilli	Bacillales	Bacillaceae	Bacillus_P
ASV1198	Non-glacial	LacADNRUFtfa	0	Bacteria	Firmicutes	Bacilli	Bacillales	Bacillaceae	Bacillus_P
ASV1198	Non-glacial	LacADNRUFtfc	0	Bacteria	Firmicutes	Bacilli	Bacillales	Bacillaceae	Bacillus_P
ASV1198	Glacial	LacAmpRUFtf-a	0	Bacteria	Firmicutes	Bacilli	Bacillales	Bacillaceae	Bacillus_P
ASV1198	Glacial	LacAmpRUFtf-b	0	Bacteria	Firmicutes	Bacilli	Bacillales	Bacillaceae	Bacillus_P
ASV1198	Glacial	LacAmpRUFtf-c	0	Bacteria	Firmicutes	Bacilli	Bacillales	Bacillaceae	Bacillus_P
ASV12	Baie de la Table	BdT0-2	0,082140125	Bacteria	Bacteroidota	Bacteroidia	Flavobacteriales	Flavobacteriaceae	Polaribacter
ASV12	Non-glacial	LacADNRUFtfa	0,021571435	Bacteria	Bacteroidota	Bacteroidia	Flavobacteriales	Flavobacteriaceae	Polaribacter
ASV12	Control	Ctr-tf-IIb	0,020193198	Bacteria	Bacteroidota	Bacteroidia	Flavobacteriales	Flavobacteriaceae	Polaribacter
ASV12	Non-glacial	LacADNRUFtfb	0,019072708	Bacteria	Bacteroidota	Bacteroidia	Flavobacteriales	Flavobacteriaceae	Polaribacter
ASV12	Glacial	LacAmpRUFtf-b	0,013713422	Bacteria	Bacteroidota	Bacteroidia	Flavobacteriales	Flavobacteriaceae	Polaribacter
ASV12	Glacial	LacAmpRUFtf-a	0,006791513	Bacteria	Bacteroidota	Bacteroidia	Flavobacteriales	Flavobacteriaceae	Polaribacter
ASV12	Glacial	LacAmpRUFtf-c	0,004212055	Bacteria	Bacteroidota	Bacteroidia	Flavobacteriales	Flavobacteriaceae	Polaribacter
ASV12	Non-glacial	LacADNRUFtfc	0,002909796	Bacteria	Bacteroidota	Bacteroidia	Flavobacteriales	Flavobacteriaceae	Polaribacter
ASV1208	Baie de la Table	BdT0-2	0	Bacteria	Acidobacteriota	Acidobacteriae	Solibacterales	Solibacteraceae	KBS-96
ASV1208	Control	Ctr-tf-IIb	0	Bacteria	Acidobacteriota	Acidobacteriae	Solibacterales	Solibacteraceae	KBS-96
ASV1208	Non-glacial	LacADNRUFtfa	0	Bacteria	Acidobacteriota	Acidobacteriae	Solibacterales	Solibacteraceae	KBS-96
ASV1208	Non-glacial	LacADNRUFtfb	0	Bacteria	Acidobacteriota	Acidobacteriae	Solibacterales	Solibacteraceae	KBS-96
ASV1208	Non-glacial	LacADNRUFtfc	0	Bacteria	Acidobacteriota	Acidobacteriae	Solibacterales	Solibacteraceae	KBS-96
ASV1208	Glacial	LacAmpRUFtf-a	0	Bacteria	Acidobacteriota	Acidobacteriae	Solibacterales	Solibacteraceae	KBS-96
ASV1208	Glacial	LacAmpRUFtf-b	0	Bacteria	Acidobacteriota	Acidobacteriae	Solibacterales	Solibacteraceae	KBS-96
ASV1208	Glacial	LacAmpRUFtf-c	0	Bacteria	Acidobacteriota	Acidobacteriae	Solibacterales	Solibacteraceae	KBS-96

ASV121	Glacial	LacAmpRUFtf-b	0,01090595	Bacteria	Proteobacteria	Gammaproteobacteria	Betaproteobacteriales	Burkholderiaceae	Janthinobacterium
ASV121	Glacial	LacAmpRUFtf-a	0,006831699	Bacteria	Proteobacteria	Gammaproteobacteria	Betaproteobacteriales	Burkholderiaceae	Janthinobacterium
ASV121	Non-glacial	LacADNRUFtfb	0,003498419	Bacteria	Proteobacteria	Gammaproteobacteria	Betaproteobacteriales	Burkholderiaceae	Janthinobacterium
ASV121	Control	Ctr-tf-IIb	0,002291768	Bacteria	Proteobacteria	Gammaproteobacteria	Betaproteobacteriales	Burkholderiaceae	Janthinobacterium
ASV121	Glacial	LacAmpRUFtf-c	0,001355604	Bacteria	Proteobacteria	Gammaproteobacteria	Betaproteobacteriales	Burkholderiaceae	Janthinobacterium
ASV121	Non-glacial	LacADNRUFtfa	0,000461915	Bacteria	Proteobacteria	Gammaproteobacteria	Betaproteobacteriales	Burkholderiaceae	Janthinobacterium
ASV121	Baie de la Table	BdT0-2	0	Bacteria	Proteobacteria	Gammaproteobacteria	Betaproteobacteriales	Burkholderiaceae	Janthinobacterium
ASV121	Non-glacial	LacADNRUFtfc	0	Bacteria	Proteobacteria	Gammaproteobacteria	Betaproteobacteriales	Burkholderiaceae	Janthinobacterium
ASV1217	Glacial	LacAmpRUFtf-a	0,000401865	Bacteria	Proteobacteria	Alphaproteobacteria	Rhodobacterales	Rhodobacteraceae	Marivita
ASV1217	Baie de la Table	BdT0-2	0	Bacteria	Proteobacteria	Alphaproteobacteria	Rhodobacterales	Rhodobacteraceae	Marivita
ASV1217	Control	Ctr-tf-IIb	0	Bacteria	Proteobacteria	Alphaproteobacteria	Rhodobacterales	Rhodobacteraceae	Marivita
ASV1217	Non-glacial	LacADNRUFtfa	0	Bacteria	Proteobacteria	Alphaproteobacteria	Rhodobacterales	Rhodobacteraceae	Marivita
ASV1217	Non-glacial	LacADNRUFtfb	0	Bacteria	Proteobacteria	Alphaproteobacteria	Rhodobacterales	Rhodobacteraceae	Marivita
ASV1217	Non-glacial	LacADNRUFtfc	0	Bacteria	Proteobacteria	Alphaproteobacteria	Rhodobacterales	Rhodobacteraceae	Marivita
ASV1217	Glacial	LacAmpRUFtf-b	0	Bacteria	Proteobacteria	Alphaproteobacteria	Rhodobacterales	Rhodobacteraceae	Marivita
ASV1217	Glacial	LacAmpRUFtf-c	0	Bacteria	Proteobacteria	Alphaproteobacteria	Rhodobacterales	Rhodobacteraceae	Marivita
ASV1222	Glacial	LacAmpRUFtf-c	0,000484144	Bacteria	Bacteroidota	Bacteroidia	Flavobacteriales	Flavobacteriaceae	Capnocytophaga
ASV1222	Baie de la Table	BdT0-2	0	Bacteria	Bacteroidota	Bacteroidia	Flavobacteriales	Flavobacteriaceae	Capnocytophaga
ASV1222	Control	Ctr-tf-IIb	0	Bacteria	Bacteroidota	Bacteroidia	Flavobacteriales	Flavobacteriaceae	Capnocytophaga
ASV1222	Non-glacial	LacADNRUFtfa	0	Bacteria	Bacteroidota	Bacteroidia	Flavobacteriales	Flavobacteriaceae	Capnocytophaga
ASV1222	Non-glacial	LacADNRUFtfb	0	Bacteria	Bacteroidota	Bacteroidia	Flavobacteriales	Flavobacteriaceae	Capnocytophaga
ASV1222	Non-glacial	LacADNRUFtfc	0	Bacteria	Bacteroidota	Bacteroidia	Flavobacteriales	Flavobacteriaceae	Capnocytophaga
ASV1222	Glacial	LacAmpRUFtf-a	0	Bacteria	Bacteroidota	Bacteroidia	Flavobacteriales	Flavobacteriaceae	Capnocytophaga
ASV1222	Glacial	LacAmpRUFtf-b	0	Bacteria	Bacteroidota	Bacteroidia	Flavobacteriales	Flavobacteriaceae	Capnocytophaga
ASV1225	Baie de la Table	BdT0-2	0	Bacteria	Proteobacteria	Gammaproteobacteria	Enterobacterales	Alteromonadaceae	Thalassomonas
ASV1225	Control	Ctr-tf-IIb	0	Bacteria	Proteobacteria	Gammaproteobacteria	Enterobacterales	Alteromonadaceae	Thalassomonas
ASV1225	Non-glacial	LacADNRUFtfa	0	Bacteria	Proteobacteria	Gammaproteobacteria	Enterobacterales	Alteromonadaceae	Thalassomonas
ASV1225	Non-glacial	LacADNRUFtfb	0	Bacteria	Proteobacteria	Gammaproteobacteria	Enterobacterales	Alteromonadaceae	Thalassomonas
ASV1225	Non-glacial	LacADNRUFtfc	0	Bacteria	Proteobacteria	Gammaproteobacteria	Enterobacterales	Alteromonadaceae	Thalassomonas
ASV1225	Glacial	LacAmpRUFtf-a	0	Bacteria	Proteobacteria	Gammaproteobacteria	Enterobacterales	Alteromonadaceae	Thalassomonas
ASV1225	Glacial	LacAmpRUFtf-b	0	Bacteria	Proteobacteria	Gammaproteobacteria	Enterobacterales	Alteromonadaceae	Thalassomonas
ASV1225	Glacial	LacAmpRUFtf-c	0	Bacteria	Proteobacteria	Gammaproteobacteria	Enterobacterales	Alteromonadaceae	Thalassomonas
ASV1235	Baie de la Table	BdT0-2	0	Bacteria	SAR324	SAR324	SAR324	NAC60-12	UBA1014
ASV1235	Control	Ctr-tf-IIb	0	Bacteria	SAR324	SAR324	SAR324	NAC60-12	UBA1014

ASV1235	Non-glacial	LacADNRUFtfa	0	Bacteria	SAR324	SAR324	SAR324	NAC60-12	UBA1014
ASV1235	Non-glacial	LacADNRUFtfb	0	Bacteria	SAR324	SAR324	SAR324	NAC60-12	UBA1014
ASV1235	Non-glacial	LacADNRUFtfc	0	Bacteria	SAR324	SAR324	SAR324	NAC60-12	UBA1014
ASV1235	Glacial	LacAmpRUFtf-a	0	Bacteria	SAR324	SAR324	SAR324	NAC60-12	UBA1014
ASV1235	Glacial	LacAmpRUFtf-b	0	Bacteria	SAR324	SAR324	SAR324	NAC60-12	UBA1014
ASV1235	Glacial	LacAmpRUFtf-c	0	Bacteria	SAR324	SAR324	SAR324	NAC60-12	UBA1014
ASV124	Baie de la Table	BdT0-2	0	Bacteria	Actinobacteriota	Acidimicrobiia	IMCC26256	IMCC26256	IMCC26256
ASV124	Control	Ctr-tf-IIb	0	Bacteria	Actinobacteriota	Acidimicrobiia	IMCC26256	IMCC26256	IMCC26256
ASV124	Non-glacial	LacADNRUFtfa	0	Bacteria	Actinobacteriota	Acidimicrobiia	IMCC26256	IMCC26256	IMCC26256
ASV124	Non-glacial	LacADNRUFtfb	0	Bacteria	Actinobacteriota	Acidimicrobiia	IMCC26256	IMCC26256	IMCC26256
ASV124	Non-glacial	LacADNRUFtfc	0	Bacteria	Actinobacteriota	Acidimicrobiia	IMCC26256	IMCC26256	IMCC26256
ASV124	Glacial	LacAmpRUFtf-a	0	Bacteria	Actinobacteriota	Acidimicrobiia	IMCC26256	IMCC26256	IMCC26256
ASV124	Glacial	LacAmpRUFtf-b	0	Bacteria	Actinobacteriota	Acidimicrobiia	IMCC26256	IMCC26256	IMCC26256
ASV124	Glacial	LacAmpRUFtf-c	0	Bacteria	Actinobacteriota	Acidimicrobiia	IMCC26256	IMCC26256	IMCC26256
ASV125	Baie de la Table	BdT0-2	0	Bacteria	Proteobacteria	Gammaproteobacteria	Enterobacterales	Enterobacteriaceae	Salmonella
ASV125	Control	Ctr-tf-IIb	0	Bacteria	Proteobacteria	Gammaproteobacteria	Enterobacterales	Enterobacteriaceae	Salmonella
ASV125	Non-glacial	LacADNRUFtfa	0	Bacteria	Proteobacteria	Gammaproteobacteria	Enterobacterales	Enterobacteriaceae	Salmonella
ASV125	Non-glacial	LacADNRUFtfb	0	Bacteria	Proteobacteria	Gammaproteobacteria	Enterobacterales	Enterobacteriaceae	Salmonella
ASV125	Non-glacial	LacADNRUFtfc	0	Bacteria	Proteobacteria	Gammaproteobacteria	Enterobacterales	Enterobacteriaceae	Salmonella
ASV125	Glacial	LacAmpRUFtf-a	0	Bacteria	Proteobacteria	Gammaproteobacteria	Enterobacterales	Enterobacteriaceae	Salmonella
ASV125	Glacial	LacAmpRUFtf-b	0	Bacteria	Proteobacteria	Gammaproteobacteria	Enterobacterales	Enterobacteriaceae	Salmonella
ASV125	Glacial	LacAmpRUFtf-c	0	Bacteria	Proteobacteria	Gammaproteobacteria	Enterobacterales	Enterobacteriaceae	Salmonella
ASV1251	Baie de la Table	BdT0-2	0	Bacteria	Bacteroidota	Bacteroidia	Chitinophagales	Saprospiraceae	OLB8
ASV1251	Control	Ctr-tf-IIb	0	Bacteria	Bacteroidota	Bacteroidia	Chitinophagales	Saprospiraceae	OLB8
ASV1251	Non-glacial	LacADNRUFtfa	0	Bacteria	Bacteroidota	Bacteroidia	Chitinophagales	Saprospiraceae	OLB8
ASV1251	Non-glacial	LacADNRUFtfb	0	Bacteria	Bacteroidota	Bacteroidia	Chitinophagales	Saprospiraceae	OLB8
ASV1251	Non-glacial	LacADNRUFtfc	0	Bacteria	Bacteroidota	Bacteroidia	Chitinophagales	Saprospiraceae	OLB8
ASV1251	Glacial	LacAmpRUFtf-a	0	Bacteria	Bacteroidota	Bacteroidia	Chitinophagales	Saprospiraceae	OLB8
ASV1251	Glacial	LacAmpRUFtf-b	0	Bacteria	Bacteroidota	Bacteroidia	Chitinophagales	Saprospiraceae	OLB8
ASV1251	Glacial	LacAmpRUFtf-c	0	Bacteria	Bacteroidota	Bacteroidia	Chitinophagales	Saprospiraceae	OLB8
ASV1260	Baie de la Table	BdT0-2	0	Archaea	Crenarchaeota	Nitrososphaeria	Nitrososphaerales	Nitrosopumilaceae	Nitrosopelagicus
ASV1260	Control	Ctr-tf-IIb	0	Archaea	Crenarchaeota	Nitrososphaeria	Nitrososphaerales	Nitrosopumilaceae	Nitrosopelagicus
ASV1260	Non-glacial	LacADNRUFtfa	0	Archaea	Crenarchaeota	Nitrososphaeria	Nitrososphaerales	Nitrosopumilaceae	Nitrosopelagicus
ASV1260	Non-glacial	LacADNRUFtfb	0	Archaea	Crenarchaeota	Nitrososphaeria	Nitrososphaerales	Nitrosopumilaceae	Nitrosopelagicus

ASV126(	Non-glacial	LacADNRUFtfc	0	Archaea	Crenarchaeota	Nitrososphaeria	Nitrososphaerales	Nitrosopumilaceae	Nitrosopelagicus
ASV126(	Glacial	LacAmpRUFtf-a	0	Archaea	Crenarchaeota	Nitrososphaeria	Nitrososphaerales	Nitrosopumilaceae	Nitrosopelagicus
ASV126(	Glacial	LacAmpRUFtf-b	0	Archaea	Crenarchaeota	Nitrososphaeria	Nitrososphaerales	Nitrosopumilaceae	Nitrosopelagicus
ASV126(	Glacial	LacAmpRUFtf-c	0	Archaea	Crenarchaeota	Nitrososphaeria	Nitrososphaerales	Nitrosopumilaceae	Nitrosopelagicus
ASV1264	Baie de la Table	BdT0-2	0	Bacteria	Proteobacteria	Gammaproteobacteria	Xanthomonadales	Xanthomonadaceae	Lysobacter
ASV1264	Control	Ctr-tf-IIb	0	Bacteria	Proteobacteria	Gammaproteobacteria	Xanthomonadales	Xanthomonadaceae	Lysobacter
ASV1264	Non-glacial	LacADNRUFtfa	0	Bacteria	Proteobacteria	Gammaproteobacteria	Xanthomonadales	Xanthomonadaceae	Lysobacter
ASV1264	Non-glacial	LacADNRUFtfb	0	Bacteria	Proteobacteria	Gammaproteobacteria	Xanthomonadales	Xanthomonadaceae	Lysobacter
ASV1264	Non-glacial	LacADNRUFtfc	0	Bacteria	Proteobacteria	Gammaproteobacteria	Xanthomonadales	Xanthomonadaceae	Lysobacter
ASV1264	Glacial	LacAmpRUFtf-a	0	Bacteria	Proteobacteria	Gammaproteobacteria	Xanthomonadales	Xanthomonadaceae	Lysobacter
ASV1264	Glacial	LacAmpRUFtf-b	0	Bacteria	Proteobacteria	Gammaproteobacteria	Xanthomonadales	Xanthomonadaceae	Lysobacter
ASV1264	Glacial	LacAmpRUFtf-c	0	Bacteria	Proteobacteria	Gammaproteobacteria	Xanthomonadales	Xanthomonadaceae	Lysobacter
ASV1265	Baie de la Table	BdT0-2	0	Bacteria	Bacteroidota	Bacteroidia	Flavobacteriales	Flavobacteriaceae	Maribacter
ASV1265	Control	Ctr-tf-IIb	0	Bacteria	Bacteroidota	Bacteroidia	Flavobacteriales	Flavobacteriaceae	Maribacter
ASV1265	Non-glacial	LacADNRUFtfa	0	Bacteria	Bacteroidota	Bacteroidia	Flavobacteriales	Flavobacteriaceae	Maribacter
ASV1265	Non-glacial	LacADNRUFtfb	0	Bacteria	Bacteroidota	Bacteroidia	Flavobacteriales	Flavobacteriaceae	Maribacter
ASV1265	Non-glacial	LacADNRUFtfc	0	Bacteria	Bacteroidota	Bacteroidia	Flavobacteriales	Flavobacteriaceae	Maribacter
ASV1265	Glacial	LacAmpRUFtf-a	0	Bacteria	Bacteroidota	Bacteroidia	Flavobacteriales	Flavobacteriaceae	Maribacter
ASV1265	Glacial	LacAmpRUFtf-b	0	Bacteria	Bacteroidota	Bacteroidia	Flavobacteriales	Flavobacteriaceae	Maribacter
ASV1265	Glacial	LacAmpRUFtf-c	0	Bacteria	Bacteroidota	Bacteroidia	Flavobacteriales	Flavobacteriaceae	Maribacter
ASV1271	Glacial	LacAmpRUFtf-b	0,000971817	Bacteria	Bacteroidota	Bacteroidia	Flavobacteriales	Flavobacteriaceae	Olleya
ASV1271	Baie de la Table	BdT0-2	0	Bacteria	Bacteroidota	Bacteroidia	Flavobacteriales	Flavobacteriaceae	Olleya
ASV1271	Control	Ctr-tf-IIb	0	Bacteria	Bacteroidota	Bacteroidia	Flavobacteriales	Flavobacteriaceae	Olleya
ASV1271	Non-glacial	LacADNRUFtfa	0	Bacteria	Bacteroidota	Bacteroidia	Flavobacteriales	Flavobacteriaceae	Olleya
ASV1271	Non-glacial	LacADNRUFtfb	0	Bacteria	Bacteroidota	Bacteroidia	Flavobacteriales	Flavobacteriaceae	Olleya
ASV1271	Non-glacial	LacADNRUFtfc	0	Bacteria	Bacteroidota	Bacteroidia	Flavobacteriales	Flavobacteriaceae	Olleya
ASV1271	Glacial	LacAmpRUFtf-a	0	Bacteria	Bacteroidota	Bacteroidia	Flavobacteriales	Flavobacteriaceae	Olleya
ASV1271	Glacial	LacAmpRUFtf-c	0	Bacteria	Bacteroidota	Bacteroidia	Flavobacteriales	Flavobacteriaceae	Olleya
ASV1272	Glacial	LacAmpRUFtf-c	0,00043573	Bacteria	Bacteroidota	Bacteroidia	Flavobacteriales	Weeksellaceae	Chryseobacterium_D
ASV1272	Baie de la Table	BdT0-2	0	Bacteria	Bacteroidota	Bacteroidia	Flavobacteriales	Weeksellaceae	Chryseobacterium_D
ASV1272	Control	Ctr-tf-IIb	0	Bacteria	Bacteroidota	Bacteroidia	Flavobacteriales	Weeksellaceae	Chryseobacterium_D
ASV1272	Non-glacial	LacADNRUFtfa	0	Bacteria	Bacteroidota	Bacteroidia	Flavobacteriales	Weeksellaceae	Chryseobacterium_D
ASV1272	Non-glacial	LacADNRUFtfb	0	Bacteria	Bacteroidota	Bacteroidia	Flavobacteriales	Weeksellaceae	Chryseobacterium_D
ASV1272	Non-glacial	LacADNRUFtfc	0	Bacteria	Bacteroidota	Bacteroidia	Flavobacteriales	Weeksellaceae	Chryseobacterium_D

ASV1272	Glacial	LacAmpRUFtf-a	0	Bacteria	Bacteroidota	Bacteroidia	Flavobacteriales	Weeksellaceae	Chryseobacterium_D
ASV1272	Glacial	LacAmpRUFtf-b	0	Bacteria	Bacteroidota	Bacteroidia	Flavobacteriales	Weeksellaceae	Chryseobacterium_D
ASV1273	Glacial	LacAmpRUFtf-c	0,00043573	Bacteria	Actinobacteriota	Actinobacteria	Actinomycetales	Actinomycetaceae	Actinomyces_B
ASV1273	Baie de la Table	BdT0-2	0	Bacteria	Actinobacteriota	Actinobacteria	Actinomycetales	Actinomycetaceae	Actinomyces_B
ASV1273	Control	Ctr-tf-IIb	0	Bacteria	Actinobacteriota	Actinobacteria	Actinomycetales	Actinomycetaceae	Actinomyces_B
ASV1273	Non-glacial	LacADNRUFtfa	0	Bacteria	Actinobacteriota	Actinobacteria	Actinomycetales	Actinomycetaceae	Actinomyces_B
ASV1273	Non-glacial	LacADNRUFtfb	0	Bacteria	Actinobacteriota	Actinobacteria	Actinomycetales	Actinomycetaceae	Actinomyces_B
ASV1273	Non-glacial	LacADNRUFtfc	0	Bacteria	Actinobacteriota	Actinobacteria	Actinomycetales	Actinomycetaceae	Actinomyces_B
ASV1273	Glacial	LacAmpRUFtf-a	0	Bacteria	Actinobacteriota	Actinobacteria	Actinomycetales	Actinomycetaceae	Actinomyces_B
ASV1273	Glacial	LacAmpRUFtf-b	0	Bacteria	Actinobacteriota	Actinobacteria	Actinomycetales	Actinomycetaceae	Actinomyces_B
ASV1275	Glacial	LacAmpRUFtf-c	0,00043573	Bacteria	Firmicutes_A	Clostridia	Oscillospirales	Ruminococcaceae	Bittarella
ASV1275	Baie de la Table	BdT0-2	0	Bacteria	Firmicutes_A	Clostridia	Oscillospirales	Ruminococcaceae	Bittarella
ASV1275	Control	Ctr-tf-IIb	0	Bacteria	Firmicutes_A	Clostridia	Oscillospirales	Ruminococcaceae	Bittarella
ASV1275	Non-glacial	LacADNRUFtfa	0	Bacteria	Firmicutes_A	Clostridia	Oscillospirales	Ruminococcaceae	Bittarella
ASV1275	Non-glacial	LacADNRUFtfb	0	Bacteria	Firmicutes_A	Clostridia	Oscillospirales	Ruminococcaceae	Bittarella
ASV1275	Non-glacial	LacADNRUFtfc	0	Bacteria	Firmicutes_A	Clostridia	Oscillospirales	Ruminococcaceae	Bittarella
ASV1275	Glacial	LacAmpRUFtf-a	0	Bacteria	Firmicutes_A	Clostridia	Oscillospirales	Ruminococcaceae	Bittarella
ASV1275	Glacial	LacAmpRUFtf-b	0	Bacteria	Firmicutes_A	Clostridia	Oscillospirales	Ruminococcaceae	Bittarella
ASV1276	Glacial	LacAmpRUFtf-c	0,00043573	Bacteria	Actinobacteriota	Actinobacteria	Actinomycetales	Micrococcaceae	Rothia
ASV1276	Baie de la Table	BdT0-2	0	Bacteria	Actinobacteriota	Actinobacteria	Actinomycetales	Micrococcaceae	Rothia
ASV1276	Control	Ctr-tf-IIb	0	Bacteria	Actinobacteriota	Actinobacteria	Actinomycetales	Micrococcaceae	Rothia
ASV1276	Non-glacial	LacADNRUFtfa	0	Bacteria	Actinobacteriota	Actinobacteria	Actinomycetales	Micrococcaceae	Rothia
ASV1276	Non-glacial	LacADNRUFtfb	0	Bacteria	Actinobacteriota	Actinobacteria	Actinomycetales	Micrococcaceae	Rothia
ASV1276	Non-glacial	LacADNRUFtfc	0	Bacteria	Actinobacteriota	Actinobacteria	Actinomycetales	Micrococcaceae	Rothia
ASV1276	Glacial	LacAmpRUFtf-a	0	Bacteria	Actinobacteriota	Actinobacteria	Actinomycetales	Micrococcaceae	Rothia
ASV1276	Glacial	LacAmpRUFtf-b	0	Bacteria	Actinobacteriota	Actinobacteria	Actinomycetales	Micrococcaceae	Rothia
ASV1284	Baie de la Table	BdT0-2	0	Bacteria	Omnitrophota	koll11	UBA10015	kpj58rc	UBA12451
ASV1284	Control	Ctr-tf-IIb	0	Bacteria	Omnitrophota	koll11	UBA10015	kpj58rc	UBA12451
ASV1284	Non-glacial	LacADNRUFtfa	0	Bacteria	Omnitrophota	koll11	UBA10015	kpj58rc	UBA12451
ASV1284	Non-glacial	LacADNRUFtfb	0	Bacteria	Omnitrophota	koll11	UBA10015	kpj58rc	UBA12451
ASV1284	Non-glacial	LacADNRUFtfc	0	Bacteria	Omnitrophota	koll11	UBA10015	kpj58rc	UBA12451
ASV1284	Glacial	LacAmpRUFtf-a	0	Bacteria	Omnitrophota	koll11	UBA10015	kpj58rc	UBA12451
ASV1284	Glacial	LacAmpRUFtf-b	0	Bacteria	Omnitrophota	koll11	UBA10015	kpj58rc	UBA12451
ASV1284	Glacial	LacAmpRUFtf-c	0	Bacteria	Omnitrophota	koll11	UBA10015	kpj58rc	UBA12451

ASV1285	Baie de la Table	BdT0-2	0	Bacteria	Bacteroidota	Bacteroidia	Chitinophagales	Chitinophagaceae	UBA8621
ASV1285	Control	Ctr-tf-IIb	0	Bacteria	Bacteroidota	Bacteroidia	Chitinophagales	Chitinophagaceae	UBA8621
ASV1285	Non-glacial	LacADNRUFtfa	0	Bacteria	Bacteroidota	Bacteroidia	Chitinophagales	Chitinophagaceae	UBA8621
ASV1285	Non-glacial	LacADNRUFtfb	0	Bacteria	Bacteroidota	Bacteroidia	Chitinophagales	Chitinophagaceae	UBA8621
ASV1285	Non-glacial	LacADNRUFtfc	0	Bacteria	Bacteroidota	Bacteroidia	Chitinophagales	Chitinophagaceae	UBA8621
ASV1285	Glacial	LacAmpRUFtf-a	0	Bacteria	Bacteroidota	Bacteroidia	Chitinophagales	Chitinophagaceae	UBA8621
ASV1285	Glacial	LacAmpRUFtf-b	0	Bacteria	Bacteroidota	Bacteroidia	Chitinophagales	Chitinophagaceae	UBA8621
ASV1285	Glacial	LacAmpRUFtf-c	0	Bacteria	Bacteroidota	Bacteroidia	Chitinophagales	Chitinophagaceae	UBA8621
ASV1286	Baie de la Table	BdT0-2	0	Bacteria	Proteobacteria	Gammaproteobacteria	Betaproteobacteriales	UKL13-2	UKL13-2
ASV1286	Control	Ctr-tf-IIb	0	Bacteria	Proteobacteria	Gammaproteobacteria	Betaproteobacteriales	UKL13-2	UKL13-2
ASV1286	Non-glacial	LacADNRUFtfa	0	Bacteria	Proteobacteria	Gammaproteobacteria	Betaproteobacteriales	UKL13-2	UKL13-2
ASV1286	Non-glacial	LacADNRUFtfb	0	Bacteria	Proteobacteria	Gammaproteobacteria	Betaproteobacteriales	UKL13-2	UKL13-2
ASV1286	Non-glacial	LacADNRUFtfc	0	Bacteria	Proteobacteria	Gammaproteobacteria	Betaproteobacteriales	UKL13-2	UKL13-2
ASV1286	Glacial	LacAmpRUFtf-a	0	Bacteria	Proteobacteria	Gammaproteobacteria	Betaproteobacteriales	UKL13-2	UKL13-2
ASV1286	Glacial	LacAmpRUFtf-b	0	Bacteria	Proteobacteria	Gammaproteobacteria	Betaproteobacteriales	UKL13-2	UKL13-2
ASV1286	Glacial	LacAmpRUFtf-c	0	Bacteria	Proteobacteria	Gammaproteobacteria	Betaproteobacteriales	UKL13-2	UKL13-2
ASV1288	Baie de la Table	BdT0-2	0	Bacteria	Cyanobacteriota	Cyanobacteriia	Cyanobacteriales	Nostocaceae	Tolypothrix_C
ASV1288	Control	Ctr-tf-IIb	0	Bacteria	Cyanobacteriota	Cyanobacteriia	Cyanobacteriales	Nostocaceae	Tolypothrix_C
ASV1288	Non-glacial	LacADNRUFtfa	0	Bacteria	Cyanobacteriota	Cyanobacteriia	Cyanobacteriales	Nostocaceae	Tolypothrix_C
ASV1288	Non-glacial	LacADNRUFtfb	0	Bacteria	Cyanobacteriota	Cyanobacteriia	Cyanobacteriales	Nostocaceae	Tolypothrix_C
ASV1288	Non-glacial	LacADNRUFtfc	0	Bacteria	Cyanobacteriota	Cyanobacteriia	Cyanobacteriales	Nostocaceae	Tolypothrix_C
ASV1288	Glacial	LacAmpRUFtf-a	0	Bacteria	Cyanobacteriota	Cyanobacteriia	Cyanobacteriales	Nostocaceae	Tolypothrix_C
ASV1288	Glacial	LacAmpRUFtf-b	0	Bacteria	Cyanobacteriota	Cyanobacteriia	Cyanobacteriales	Nostocaceae	Tolypothrix_C
ASV1288	Glacial	LacAmpRUFtf-c	0	Bacteria	Cyanobacteriota	Cyanobacteriia	Cyanobacteriales	Nostocaceae	Tolypothrix_C
ASV1295	Baie de la Table	BdT0-2	0	Bacteria	Planctomycetota	Phycisphaerae	Phycisphaerales	SM1A02	UBA12014
ASV1295	Control	Ctr-tf-IIb	0	Bacteria	Planctomycetota	Phycisphaerae	Phycisphaerales	SM1A02	UBA12014
ASV1295	Non-glacial	LacADNRUFtfa	0	Bacteria	Planctomycetota	Phycisphaerae	Phycisphaerales	SM1A02	UBA12014
ASV1295	Non-glacial	LacADNRUFtfb	0	Bacteria	Planctomycetota	Phycisphaerae	Phycisphaerales	SM1A02	UBA12014
ASV1295	Non-glacial	LacADNRUFtfc	0	Bacteria	Planctomycetota	Phycisphaerae	Phycisphaerales	SM1A02	UBA12014
ASV1295	Glacial	LacAmpRUFtf-a	0	Bacteria	Planctomycetota	Phycisphaerae	Phycisphaerales	SM1A02	UBA12014
ASV1295	Glacial	LacAmpRUFtf-b	0	Bacteria	Planctomycetota	Phycisphaerae	Phycisphaerales	SM1A02	UBA12014
ASV1295	Glacial	LacAmpRUFtf-c	0	Bacteria	Planctomycetota	Phycisphaerae	Phycisphaerales	SM1A02	UBA12014
ASV13	Glacial	LacAmpRUFtf-c	0,195884774	Bacteria	Proteobacteria	Gammaproteobacteria	Betaproteobacteriales	Burkholderiaceae	Delftia
ASV13	Non-glacial	LacADNRUFtfb	0,098356164	Bacteria	Proteobacteria	Gammaproteobacteria	Betaproteobacteriales	Burkholderiaceae	Delftia

ASV13	Glacial	LacAmpRUFtf-b	0,078393262	Bacteria	Proteobacteria	Gammaproteobacteria	Betaproteobacteriales	Burkholderiaceae	Delftia
ASV13	Non-glacial	LacADNRUFtfa	0,026052012	Bacteria	Proteobacteria	Gammaproteobacteria	Betaproteobacteriales	Burkholderiaceae	Delftia
ASV13	Non-glacial	LacADNRUFtfc	0,009796314	Bacteria	Proteobacteria	Gammaproteobacteria	Betaproteobacteriales	Burkholderiaceae	Delftia
ASV13	Glacial	LacAmpRUFtf-a	0,004058833	Bacteria	Proteobacteria	Gammaproteobacteria	Betaproteobacteriales	Burkholderiaceae	Delftia
ASV13	Baie de la Table	BdT0-2	0	Bacteria	Proteobacteria	Gammaproteobacteria	Betaproteobacteriales	Burkholderiaceae	Delftia
ASV13	Control	Ctr-tf-IIb	0	Bacteria	Proteobacteria	Gammaproteobacteria	Betaproteobacteriales	Burkholderiaceae	Delftia
ASV130	Baie de la Table	BdT0-2	0,00562903	Bacteria	Verrucomicrobio	Verrucomicrobiae	Opitutales	Puniceicoccaceae	GCA-2690565
ASV130	Glacial	LacAmpRUFtf-a	0,000723356	Bacteria	Verrucomicrobio	Verrucomicrobiae	Opitutales	Puniceicoccaceae	GCA-2690565
ASV130	Non-glacial	LacADNRUFtfa	0,000415724	Bacteria	Verrucomicrobio	Verrucomicrobiae	Opitutales	Puniceicoccaceae	GCA-2690565
ASV130	Non-glacial	LacADNRUFtfb	0,000337197	Bacteria	Verrucomicrobio	Verrucomicrobiae	Opitutales	Puniceicoccaceae	GCA-2690565
ASV130	Control	Ctr-tf-IIb	0,000228375	Bacteria	Verrucomicrobio	Verrucomicrobiae	Opitutales	Puniceicoccaceae	GCA-2690565
ASV130	Non-glacial	LacADNRUFtfc	0	Bacteria	Verrucomicrobio	Verrucomicrobiae	Opitutales	Puniceicoccaceae	GCA-2690565
ASV130	Glacial	LacAmpRUFtf-b	0	Bacteria	Verrucomicrobio	Verrucomicrobiae	Opitutales	Puniceicoccaceae	GCA-2690565
ASV130	Glacial	LacAmpRUFtf-c	0	Bacteria	Verrucomicrobio	Verrucomicrobiae	Opitutales	Puniceicoccaceae	GCA-2690565
ASV131	Non-glacial	LacADNRUFtfb	0,003962065	Bacteria	Proteobacteria	Alphaproteobacteria	Sphingomonadales	Sphingomonadaceae	Porphyrobacter
ASV131	Non-glacial	LacADNRUFtfa	0,000554298	Bacteria	Proteobacteria	Alphaproteobacteria	Sphingomonadales	Sphingomonadaceae	Porphyrobacter
ASV131	Glacial	LacAmpRUFtf-c	0,000484144	Bacteria	Proteobacteria	Alphaproteobacteria	Sphingomonadales	Sphingomonadaceae	Porphyrobacter
ASV131	Baie de la Table	BdT0-2	0	Bacteria	Proteobacteria	Alphaproteobacteria	Sphingomonadales	Sphingomonadaceae	Porphyrobacter
ASV131	Control	Ctr-tf-IIb	0	Bacteria	Proteobacteria	Alphaproteobacteria	Sphingomonadales	Sphingomonadaceae	Porphyrobacter
ASV131	Non-glacial	LacADNRUFtfc	0	Bacteria	Proteobacteria	Alphaproteobacteria	Sphingomonadales	Sphingomonadaceae	Porphyrobacter
ASV131	Glacial	LacAmpRUFtf-a	0	Bacteria	Proteobacteria	Alphaproteobacteria	Sphingomonadales	Sphingomonadaceae	Porphyrobacter
ASV131	Glacial	LacAmpRUFtf-b	0	Bacteria	Proteobacteria	Alphaproteobacteria	Sphingomonadales	Sphingomonadaceae	Porphyrobacter
ASV1311	Baie de la Table	BdT0-2	0	Bacteria	Firmicutes_C	Negativicutes	Veillonellales	Veillonellaceae	F0422
ASV1311	Control	Ctr-tf-IIb	0	Bacteria	Firmicutes_C	Negativicutes	Veillonellales	Veillonellaceae	F0422
ASV1311	Non-glacial	LacADNRUFtfa	0	Bacteria	Firmicutes_C	Negativicutes	Veillonellales	Veillonellaceae	F0422
ASV1311	Non-glacial	LacADNRUFtfb	0	Bacteria	Firmicutes_C	Negativicutes	Veillonellales	Veillonellaceae	F0422
ASV1311	Non-glacial	LacADNRUFtfc	0	Bacteria	Firmicutes_C	Negativicutes	Veillonellales	Veillonellaceae	F0422
ASV1311	Glacial	LacAmpRUFtf-a	0	Bacteria	Firmicutes_C	Negativicutes	Veillonellales	Veillonellaceae	F0422
ASV1311	Glacial	LacAmpRUFtf-b	0	Bacteria	Firmicutes_C	Negativicutes	Veillonellales	Veillonellaceae	F0422
ASV1311	Glacial	LacAmpRUFtf-c	0	Bacteria	Firmicutes_C	Negativicutes	Veillonellales	Veillonellaceae	F0422
ASV1316	Baie de la Table	BdT0-2	0	Bacteria	Proteobacteria	Gammaproteobacteria	Pseudomonadales	Saccharospirillaceae	Reinekea
ASV1316	Control	Ctr-tf-IIb	0	Bacteria	Proteobacteria	Gammaproteobacteria	Pseudomonadales	Saccharospirillaceae	Reinekea
ASV1316	Non-glacial	LacADNRUFtfa	0	Bacteria	Proteobacteria	Gammaproteobacteria	Pseudomonadales	Saccharospirillaceae	Reinekea
ASV1316	Non-glacial	LacADNRUFtfb	0	Bacteria	Proteobacteria	Gammaproteobacteria	Pseudomonadales	Saccharospirillaceae	Reinekea

ASV1316	Non-glacial	LacADNRUFtfc	0	Bacteria	Proteobacteria	Gammaproteobacteria	Pseudomonadales	Saccharospirillaceae	Reinekea
ASV1316	Glacial	LacAmpRUFtf-a	0	Bacteria	Proteobacteria	Gammaproteobacteria	Pseudomonadales	Saccharospirillaceae	Reinekea
ASV1316	Glacial	LacAmpRUFtf-b	0	Bacteria	Proteobacteria	Gammaproteobacteria	Pseudomonadales	Saccharospirillaceae	Reinekea
ASV1316	Glacial	LacAmpRUFtf-c	0	Bacteria	Proteobacteria	Gammaproteobacteria	Pseudomonadales	Saccharospirillaceae	Reinekea
ASV1320	Baie de la Table	BdT0-2	0	Bacteria	Nitrospirota	Nitrospiria	2-01-FULL-66-17	2-01-FULL-66-17	2-01-FULL-66-17
ASV1320	Control	Ctr-tf-IIb	0	Bacteria	Nitrospirota	Nitrospiria	2-01-FULL-66-17	2-01-FULL-66-17	2-01-FULL-66-17
ASV1320	Non-glacial	LacADNRUFtfa	0	Bacteria	Nitrospirota	Nitrospiria	2-01-FULL-66-17	2-01-FULL-66-17	2-01-FULL-66-17
ASV1320	Non-glacial	LacADNRUFtfb	0	Bacteria	Nitrospirota	Nitrospiria	2-01-FULL-66-17	2-01-FULL-66-17	2-01-FULL-66-17
ASV1320	Non-glacial	LacADNRUFtfc	0	Bacteria	Nitrospirota	Nitrospiria	2-01-FULL-66-17	2-01-FULL-66-17	2-01-FULL-66-17
ASV1320	Glacial	LacAmpRUFtf-a	0	Bacteria	Nitrospirota	Nitrospiria	2-01-FULL-66-17	2-01-FULL-66-17	2-01-FULL-66-17
ASV1320	Glacial	LacAmpRUFtf-b	0	Bacteria	Nitrospirota	Nitrospiria	2-01-FULL-66-17	2-01-FULL-66-17	2-01-FULL-66-17
ASV1320	Glacial	LacAmpRUFtf-c	0	Bacteria	Nitrospirota	Nitrospiria	2-01-FULL-66-17	2-01-FULL-66-17	2-01-FULL-66-17
ASV1323	Baie de la Table	BdT0-2	0	Bacteria	Chloroflexota	Ellin6529	CSP1-4	CSP1-4	GWC2-73-18
ASV1323	Control	Ctr-tf-IIb	0	Bacteria	Chloroflexota	Ellin6529	CSP1-4	CSP1-4	GWC2-73-18
ASV1323	Non-glacial	LacADNRUFtfa	0	Bacteria	Chloroflexota	Ellin6529	CSP1-4	CSP1-4	GWC2-73-18
ASV1323	Non-glacial	LacADNRUFtfb	0	Bacteria	Chloroflexota	Ellin6529	CSP1-4	CSP1-4	GWC2-73-18
ASV1323	Non-glacial	LacADNRUFtfc	0	Bacteria	Chloroflexota	Ellin6529	CSP1-4	CSP1-4	GWC2-73-18
ASV1323	Glacial	LacAmpRUFtf-a	0	Bacteria	Chloroflexota	Ellin6529	CSP1-4	CSP1-4	GWC2-73-18
ASV1323	Glacial	LacAmpRUFtf-b	0	Bacteria	Chloroflexota	Ellin6529	CSP1-4	CSP1-4	GWC2-73-18
ASV1323	Glacial	LacAmpRUFtf-c	0	Bacteria	Chloroflexota	Ellin6529	CSP1-4	CSP1-4	GWC2-73-18
ASV1326	Baie de la Table	BdT0-2	0	Archaea	Nanoarchaeota	Woesearchaeia	SM23-78	UBA9642	UBA9642
ASV1326	Control	Ctr-tf-IIb	0	Archaea	Nanoarchaeota	Woesearchaeia	SM23-78	UBA9642	UBA9642
ASV1326	Non-glacial	LacADNRUFtfa	0	Archaea	Nanoarchaeota	Woesearchaeia	SM23-78	UBA9642	UBA9642
ASV1326	Non-glacial	LacADNRUFtfb	0	Archaea	Nanoarchaeota	Woesearchaeia	SM23-78	UBA9642	UBA9642
ASV1326	Non-glacial	LacADNRUFtfc	0	Archaea	Nanoarchaeota	Woesearchaeia	SM23-78	UBA9642	UBA9642
ASV1326	Glacial	LacAmpRUFtf-a	0	Archaea	Nanoarchaeota	Woesearchaeia	SM23-78	UBA9642	UBA9642
ASV1326	Glacial	LacAmpRUFtf-b	0	Archaea	Nanoarchaeota	Woesearchaeia	SM23-78	UBA9642	UBA9642
ASV1326	Glacial	LacAmpRUFtf-c	0	Archaea	Nanoarchaeota	Woesearchaeia	SM23-78	UBA9642	UBA9642
ASV1327	Baie de la Table	BdT0-2	0	Bacteria	Proteobacteria	Alphaproteobacteria	Rhizobiales	Rhizobiaceae	Shinella
ASV1327	Control	Ctr-tf-IIb	0	Bacteria	Proteobacteria	Alphaproteobacteria	Rhizobiales	Rhizobiaceae	Shinella
ASV1327	Non-glacial	LacADNRUFtfa	0	Bacteria	Proteobacteria	Alphaproteobacteria	Rhizobiales	Rhizobiaceae	Shinella
ASV1327	Non-glacial	LacADNRUFtfb	0	Bacteria	Proteobacteria	Alphaproteobacteria	Rhizobiales	Rhizobiaceae	Shinella
ASV1327	Non-glacial	LacADNRUFtfc	0	Bacteria	Proteobacteria	Alphaproteobacteria	Rhizobiales	Rhizobiaceae	Shinella
ASV1327	Glacial	LacAmpRUFtf-a	0	Bacteria	Proteobacteria	Alphaproteobacteria	Rhizobiales	Rhizobiaceae	Shinella

ASV1327	Glacial	LacAmpRUFtf-b	0	Bacteria	Proteobacteria	Alphaproteobacteria	Rhizobiales	Rhizobiaceae	Shinella
ASV1327	Glacial	LacAmpRUFtf-c	0	Bacteria	Proteobacteria	Alphaproteobacteria	Rhizobiales	Rhizobiaceae	Shinella
ASV133	Non-glacial	LacADNRUFtfa	0,006004896	Bacteria	Proteobacteria	Gammaproteobacteria	Pseudomonadales	Spongiibacteraceae	Oceanicoccus
ASV133	Non-glacial	LacADNRUFtfb	0,002676502	Bacteria	Proteobacteria	Gammaproteobacteria	Pseudomonadales	Spongiibacteraceae	Oceanicoccus
ASV133	Glacial	LacAmpRUFtf-a	0,001848577	Bacteria	Proteobacteria	Gammaproteobacteria	Pseudomonadales	Spongiibacteraceae	Oceanicoccus
ASV133	Control	Ctr-tf-IIb	0,001774918	Bacteria	Proteobacteria	Gammaproteobacteria	Pseudomonadales	Spongiibacteraceae	Oceanicoccus
ASV133	Non-glacial	LacADNRUFtfc	0,001721629	Bacteria	Proteobacteria	Gammaproteobacteria	Pseudomonadales	Spongiibacteraceae	Oceanicoccus
ASV133	Glacial	LacAmpRUFtf-c	0,000677802	Bacteria	Proteobacteria	Gammaproteobacteria	Pseudomonadales	Spongiibacteraceae	Oceanicoccus
ASV133	Glacial	LacAmpRUFtf-b	0,000539898	Bacteria	Proteobacteria	Gammaproteobacteria	Pseudomonadales	Spongiibacteraceae	Oceanicoccus
ASV133	Baie de la Table	BdT0-2	0	Bacteria	Proteobacteria	Gammaproteobacteria	Pseudomonadales	Spongiibacteraceae	Oceanicoccus
ASV1338	Glacial	LacAmpRUFtf-b	0,000863838	Bacteria	Bacteroidota	Bacteroidia	Bacteroidales	Porphyromonadaceae	Porphyromonas
ASV1338	Baie de la Table	BdT0-2	0	Bacteria	Bacteroidota	Bacteroidia	Bacteroidales	Porphyromonadaceae	Porphyromonas
ASV1338	Control	Ctr-tf-IIb	0	Bacteria	Bacteroidota	Bacteroidia	Bacteroidales	Porphyromonadaceae	Porphyromonas
ASV1338	Non-glacial	LacADNRUFtfa	0	Bacteria	Bacteroidota	Bacteroidia	Bacteroidales	Porphyromonadaceae	Porphyromonas
ASV1338	Non-glacial	LacADNRUFtfb	0	Bacteria	Bacteroidota	Bacteroidia	Bacteroidales	Porphyromonadaceae	Porphyromonas
ASV1338	Non-glacial	LacADNRUFtfc	0	Bacteria	Bacteroidota	Bacteroidia	Bacteroidales	Porphyromonadaceae	Porphyromonas
ASV1338	Glacial	LacAmpRUFtf-a	0	Bacteria	Bacteroidota	Bacteroidia	Bacteroidales	Porphyromonadaceae	Porphyromonas
ASV1338	Glacial	LacAmpRUFtf-c	0	Bacteria	Bacteroidota	Bacteroidia	Bacteroidales	Porphyromonadaceae	Porphyromonas
ASV1339	Glacial	LacAmpRUFtf-c	0,000387315	Bacteria	Proteobacteria	Gammaproteobacteria	Xanthomonadales	Xanthomonadaceae	Luteimonas_A
ASV1339	Baie de la Table	BdT0-2	0	Bacteria	Proteobacteria	Gammaproteobacteria	Xanthomonadales	Xanthomonadaceae	Luteimonas_A
ASV1339	Control	Ctr-tf-IIb	0	Bacteria	Proteobacteria	Gammaproteobacteria	Xanthomonadales	Xanthomonadaceae	Luteimonas_A
ASV1339	Non-glacial	LacADNRUFtfa	0	Bacteria	Proteobacteria	Gammaproteobacteria	Xanthomonadales	Xanthomonadaceae	Luteimonas_A
ASV1339	Non-glacial	LacADNRUFtfb	0	Bacteria	Proteobacteria	Gammaproteobacteria	Xanthomonadales	Xanthomonadaceae	Luteimonas_A
ASV1339	Non-glacial	LacADNRUFtfc	0	Bacteria	Proteobacteria	Gammaproteobacteria	Xanthomonadales	Xanthomonadaceae	Luteimonas_A
ASV1339	Glacial	LacAmpRUFtf-a	0	Bacteria	Proteobacteria	Gammaproteobacteria	Xanthomonadales	Xanthomonadaceae	Luteimonas_A
ASV1339	Glacial	LacAmpRUFtf-b	0	Bacteria	Proteobacteria	Gammaproteobacteria	Xanthomonadales	Xanthomonadaceae	Luteimonas_A
ASV134	Control	Ctr-tf-IIb	0,003000934	Bacteria	Proteobacteria	Alphaproteobacteria	Caulobacterales	Maricaulaceae	Robiginitomaculum
ASV134	Non-glacial	LacADNRUFtfb	0,001369863	Bacteria	Proteobacteria	Alphaproteobacteria	Caulobacterales	Maricaulaceae	Robiginitomaculum
ASV134	Baie de la Table	BdT0-2	0,000655809	Bacteria	Proteobacteria	Alphaproteobacteria	Caulobacterales	Maricaulaceae	Robiginitomaculum
ASV134	Non-glacial	LacADNRUFtfc	0,000509214	Bacteria	Proteobacteria	Alphaproteobacteria	Caulobacterales	Maricaulaceae	Robiginitomaculum
ASV134	Glacial	LacAmpRUFtf-b	0,000323939	Bacteria	Proteobacteria	Alphaproteobacteria	Caulobacterales	Maricaulaceae	Robiginitomaculum
ASV134	Non-glacial	LacADNRUFtfa	0,000323341	Bacteria	Proteobacteria	Alphaproteobacteria	Caulobacterales	Maricaulaceae	Robiginitomaculum
ASV134	Glacial	LacAmpRUFtf-a	0,000281305	Bacteria	Proteobacteria	Alphaproteobacteria	Caulobacterales	Maricaulaceae	Robiginitomaculum
ASV134	Glacial	LacAmpRUFtf-c	0,000145243	Bacteria	Proteobacteria	Alphaproteobacteria	Caulobacterales	Maricaulaceae	Robiginitomaculum

ASV1351	Baie de la Table	BdT0-2	0	Bacteria	Proteobacteria	Gammaproteobacteria	Thiomicrospirales	Thioglobaceae	SUP05
ASV1351	Control	Ctr-tf-IIb	0	Bacteria	Proteobacteria	Gammaproteobacteria	Thiomicrospirales	Thioglobaceae	SUP05
ASV1351	Non-glacial	LacADNRUFtfa	0	Bacteria	Proteobacteria	Gammaproteobacteria	Thiomicrospirales	Thioglobaceae	SUP05
ASV1351	Non-glacial	LacADNRUFtfb	0	Bacteria	Proteobacteria	Gammaproteobacteria	Thiomicrospirales	Thioglobaceae	SUP05
ASV1351	Non-glacial	LacADNRUFtfc	0	Bacteria	Proteobacteria	Gammaproteobacteria	Thiomicrospirales	Thioglobaceae	SUP05
ASV1351	Glacial	LacAmpRUFtf-a	0	Bacteria	Proteobacteria	Gammaproteobacteria	Thiomicrospirales	Thioglobaceae	SUP05
ASV1351	Glacial	LacAmpRUFtf-b	0	Bacteria	Proteobacteria	Gammaproteobacteria	Thiomicrospirales	Thioglobaceae	SUP05
ASV1351	Glacial	LacAmpRUFtf-c	0	Bacteria	Proteobacteria	Gammaproteobacteria	Thiomicrospirales	Thioglobaceae	SUP05
ASV1353	Non-glacial	LacADNRUFtfc	7,27E-05	Bacteria	Proteobacteria	Gammaproteobacteria	Granulosicoccales	Granulosicoccaceae	Granulosicoccus
ASV1353	Baie de la Table	BdT0-2	0	Bacteria	Proteobacteria	Gammaproteobacteria	Granulosicoccales	Granulosicoccaceae	Granulosicoccus
ASV1353	Control	Ctr-tf-IIb	0	Bacteria	Proteobacteria	Gammaproteobacteria	Granulosicoccales	Granulosicoccaceae	Granulosicoccus
ASV1353	Non-glacial	LacADNRUFtfa	0	Bacteria	Proteobacteria	Gammaproteobacteria	Granulosicoccales	Granulosicoccaceae	Granulosicoccus
ASV1353	Non-glacial	LacADNRUFtfb	0	Bacteria	Proteobacteria	Gammaproteobacteria	Granulosicoccales	Granulosicoccaceae	Granulosicoccus
ASV1353	Glacial	LacAmpRUFtf-a	0	Bacteria	Proteobacteria	Gammaproteobacteria	Granulosicoccales	Granulosicoccaceae	Granulosicoccus
ASV1353	Glacial	LacAmpRUFtf-b	0	Bacteria	Proteobacteria	Gammaproteobacteria	Granulosicoccales	Granulosicoccaceae	Granulosicoccus
ASV1353	Glacial	LacAmpRUFtf-c	0	Bacteria	Proteobacteria	Gammaproteobacteria	Granulosicoccales	Granulosicoccaceae	Granulosicoccus
ASV1359	Baie de la Table	BdT0-2	0	Bacteria	Proteobacteria	Gammaproteobacteria	Betaproteobacteriales	Chitinibacteraceae	B9-8
ASV1359	Control	Ctr-tf-IIb	0	Bacteria	Proteobacteria	Gammaproteobacteria	Betaproteobacteriales	Chitinibacteraceae	B9-8
ASV1359	Non-glacial	LacADNRUFtfa	0	Bacteria	Proteobacteria	Gammaproteobacteria	Betaproteobacteriales	Chitinibacteraceae	B9-8
ASV1359	Non-glacial	LacADNRUFtfb	0	Bacteria	Proteobacteria	Gammaproteobacteria	Betaproteobacteriales	Chitinibacteraceae	B9-8
ASV1359	Non-glacial	LacADNRUFtfc	0	Bacteria	Proteobacteria	Gammaproteobacteria	Betaproteobacteriales	Chitinibacteraceae	B9-8
ASV1359	Glacial	LacAmpRUFtf-a	0	Bacteria	Proteobacteria	Gammaproteobacteria	Betaproteobacteriales	Chitinibacteraceae	B9-8
ASV1359	Glacial	LacAmpRUFtf-b	0	Bacteria	Proteobacteria	Gammaproteobacteria	Betaproteobacteriales	Chitinibacteraceae	B9-8
ASV1359	Glacial	LacAmpRUFtf-c	0	Bacteria	Proteobacteria	Gammaproteobacteria	Betaproteobacteriales	Chitinibacteraceae	B9-8
ASV137	Baie de la Table	BdT0-2	0,002841841	Bacteria	Proteobacteria	Gammaproteobacteria	Betaproteobacteriales	Methylophilaceae	BACL14
ASV137	Non-glacial	LacADNRUFtfb	0,000990516	Bacteria	Proteobacteria	Gammaproteobacteria	Betaproteobacteriales	Methylophilaceae	BACL14
ASV137	Non-glacial	LacADNRUFtfa	0,000508107	Bacteria	Proteobacteria	Gammaproteobacteria	Betaproteobacteriales	Methylophilaceae	BACL14
ASV137	Control	Ctr-tf-IIb	0,000312514	Bacteria	Proteobacteria	Gammaproteobacteria	Betaproteobacteriales	Methylophilaceae	BACL14
ASV137	Non-glacial	LacADNRUFtfc	0	Bacteria	Proteobacteria	Gammaproteobacteria	Betaproteobacteriales	Methylophilaceae	BACL14
ASV137	Glacial	LacAmpRUFtf-a	0	Bacteria	Proteobacteria	Gammaproteobacteria	Betaproteobacteriales	Methylophilaceae	BACL14
ASV137	Glacial	LacAmpRUFtf-b	0	Bacteria	Proteobacteria	Gammaproteobacteria	Betaproteobacteriales	Methylophilaceae	BACL14
ASV137	Glacial	LacAmpRUFtf-c	0	Bacteria	Proteobacteria	Gammaproteobacteria	Betaproteobacteriales	Methylophilaceae	BACL14
ASV1370	Baie de la Table	BdT0-2	0	Bacteria	Planctomycetota	Planctomycetes	Gemmatales	Gemmataceae	Fimbriiglobus
ASV1370	Control	Ctr-tf-IIb	0	Bacteria	Planctomycetota	Planctomycetes	Gemmatales	Gemmataceae	Fimbriiglobus

ASV137(	Non-glacial	LacADNRUFtfa	0	Bacteria	Planctomycetota	Planctomycetes	Gemmatales	Gemmataceae	Fimbriiglobus
ASV137(	Non-glacial	LacADNRUFtfb	0	Bacteria	Planctomycetota	Planctomycetes	Gemmatales	Gemmataceae	Fimbriiglobus
ASV137(	Non-glacial	LacADNRUFtfc	0	Bacteria	Planctomycetota	Planctomycetes	Gemmatales	Gemmataceae	Fimbriiglobus
ASV137(	Glacial	LacAmpRUFtf-a	0	Bacteria	Planctomycetota	Planctomycetes	Gemmatales	Gemmataceae	Fimbriiglobus
ASV137(	Glacial	LacAmpRUFtf-b	0	Bacteria	Planctomycetota	Planctomycetes	Gemmatales	Gemmataceae	Fimbriiglobus
ASV137(	Glacial	LacAmpRUFtf-c	0	Bacteria	Planctomycetota	Planctomycetes	Gemmatales	Gemmataceae	Fimbriiglobus
ASV138	Baie de la Table	BdT0-2	0,006558094	Bacteria	Proteobacteria	Alphaproteobacteria	Pelagibacterales	Pelagibacteraceae	Pelagibacter_A
ASV138	Glacial	LacAmpRUFtf-a	0,003214917	Bacteria	Proteobacteria	Alphaproteobacteria	Pelagibacterales	Pelagibacteraceae	Pelagibacter_A
ASV138	Non-glacial	LacADNRUFtfa	0,001478128	Bacteria	Proteobacteria	Alphaproteobacteria	Pelagibacterales	Pelagibacteraceae	Pelagibacter_A
ASV138	Glacial	LacAmpRUFtf-b	0,001187777	Bacteria	Proteobacteria	Alphaproteobacteria	Pelagibacterales	Pelagibacteraceae	Pelagibacter_A
ASV138	Control	Ctr-tf-IIb	0,000701153	Bacteria	Proteobacteria	Alphaproteobacteria	Pelagibacterales	Pelagibacteraceae	Pelagibacter_A
ASV138	Non-glacial	LacADNRUFtfc	0,000169738	Bacteria	Proteobacteria	Alphaproteobacteria	Pelagibacterales	Pelagibacteraceae	Pelagibacter_A
ASV138	Non-glacial	LacADNRUFtfb	0	Bacteria	Proteobacteria	Alphaproteobacteria	Pelagibacterales	Pelagibacteraceae	Pelagibacter_A
ASV138	Glacial	LacAmpRUFtf-c	0	Bacteria	Proteobacteria	Alphaproteobacteria	Pelagibacterales	Pelagibacteraceae	Pelagibacter_A
ASV1384	Baie de la Table	BdT0-2	0	Bacteria	Proteobacteria	Gammaproteobacteria	Betaproteobacteriales	Hydrogenophilaceae	Thiobacillus
ASV1384	Control	Ctr-tf-IIb	0	Bacteria	Proteobacteria	Gammaproteobacteria	Betaproteobacteriales	Hydrogenophilaceae	Thiobacillus
ASV1384	Non-glacial	LacADNRUFtfa	0	Bacteria	Proteobacteria	Gammaproteobacteria	Betaproteobacteriales	Hydrogenophilaceae	Thiobacillus
ASV1384	Non-glacial	LacADNRUFtfb	0	Bacteria	Proteobacteria	Gammaproteobacteria	Betaproteobacteriales	Hydrogenophilaceae	Thiobacillus
ASV1384	Non-glacial	LacADNRUFtfc	0	Bacteria	Proteobacteria	Gammaproteobacteria	Betaproteobacteriales	Hydrogenophilaceae	Thiobacillus
ASV1384	Glacial	LacAmpRUFtf-a	0	Bacteria	Proteobacteria	Gammaproteobacteria	Betaproteobacteriales	Hydrogenophilaceae	Thiobacillus
ASV1384	Glacial	LacAmpRUFtf-b	0	Bacteria	Proteobacteria	Gammaproteobacteria	Betaproteobacteriales	Hydrogenophilaceae	Thiobacillus
ASV1384	Glacial	LacAmpRUFtf-c	0	Bacteria	Proteobacteria	Gammaproteobacteria	Betaproteobacteriales	Hydrogenophilaceae	Thiobacillus
ASV1396	Baie de la Table	BdT0-2	0	Bacteria	Patescibacteria	Doudnabacteria	UBA920	UBA920	UBA920
ASV1396	Control	Ctr-tf-IIb	0	Bacteria	Patescibacteria	Doudnabacteria	UBA920	UBA920	UBA920
ASV1396	Non-glacial	LacADNRUFtfa	0	Bacteria	Patescibacteria	Doudnabacteria	UBA920	UBA920	UBA920
ASV1396	Non-glacial	LacADNRUFtfb	0	Bacteria	Patescibacteria	Doudnabacteria	UBA920	UBA920	UBA920
ASV1396	Non-glacial	LacADNRUFtfc	0	Bacteria	Patescibacteria	Doudnabacteria	UBA920	UBA920	UBA920
ASV1396	Glacial	LacAmpRUFtf-a	0	Bacteria	Patescibacteria	Doudnabacteria	UBA920	UBA920	UBA920
ASV1396	Glacial	LacAmpRUFtf-b	0	Bacteria	Patescibacteria	Doudnabacteria	UBA920	UBA920	UBA920
ASV1396	Glacial	LacAmpRUFtf-c	0	Bacteria	Patescibacteria	Doudnabacteria	UBA920	UBA920	UBA920
ASV1397	Baie de la Table	BdT0-2	0	Bacteria	Bacteroidota	Bacteroidia	Chitinophagales	Chitinophagaceae	Flavihumibacter
ASV1397	Control	Ctr-tf-IIb	0	Bacteria	Bacteroidota	Bacteroidia	Chitinophagales	Chitinophagaceae	Flavihumibacter
ASV1397	Non-glacial	LacADNRUFtfa	0	Bacteria	Bacteroidota	Bacteroidia	Chitinophagales	Chitinophagaceae	Flavihumibacter
ASV1397	Non-glacial	LacADNRUFtfb	0	Bacteria	Bacteroidota	Bacteroidia	Chitinophagales	Chitinophagaceae	Flavihumibacter

ASV1397	Non-glacial	LacADNRUFtfc	0	Bacteria	Bacteroidota	Bacteroidia	Chitinophagales	Chitinophagaceae	Flavihumibacter
ASV1397	Glacial	LacAmpRUFtf-a	0	Bacteria	Bacteroidota	Bacteroidia	Chitinophagales	Chitinophagaceae	Flavihumibacter
ASV1397	Glacial	LacAmpRUFtf-b	0	Bacteria	Bacteroidota	Bacteroidia	Chitinophagales	Chitinophagaceae	Flavihumibacter
ASV1397	Glacial	LacAmpRUFtf-c	0	Bacteria	Bacteroidota	Bacteroidia	Chitinophagales	Chitinophagaceae	Flavihumibacter
ASV14	Non-glacial	LacADNRUFtfc	0,23171678	Bacteria	Proteobacteria	Alphaproteobacteria	Rhizobiales	Hyphomicrobiaceae	Filomicrobium
ASV14	Glacial	LacAmpRUFtf-b	0,035633301	Bacteria	Proteobacteria	Alphaproteobacteria	Rhizobiales	Hyphomicrobiaceae	Filomicrobium
ASV14	Control	Ctr-tf-IIb	0,035554452	Bacteria	Proteobacteria	Alphaproteobacteria	Rhizobiales	Hyphomicrobiaceae	Filomicrobium
ASV14	Glacial	LacAmpRUFtf-a	0,012297058	Bacteria	Proteobacteria	Alphaproteobacteria	Rhizobiales	Hyphomicrobiaceae	Filomicrobium
ASV14	Non-glacial	LacADNRUFtfb	0,004847208	Bacteria	Proteobacteria	Alphaproteobacteria	Rhizobiales	Hyphomicrobiaceae	Filomicrobium
ASV14	Non-glacial	LacADNRUFtfa	0,000230958	Bacteria	Proteobacteria	Alphaproteobacteria	Rhizobiales	Hyphomicrobiaceae	Filomicrobium
ASV14	Glacial	LacAmpRUFtf-c	0,000145243	Bacteria	Proteobacteria	Alphaproteobacteria	Rhizobiales	Hyphomicrobiaceae	Filomicrobium
ASV14	Baie de la Table	BdT0-2	0	Bacteria	Proteobacteria	Alphaproteobacteria	Rhizobiales	Hyphomicrobiaceae	Filomicrobium
ASV140	Baie de la Table	BdT0-2	0	Bacteria	Planctomycetota	Planctomycetes	Planctomycetales	Planctomycetaceae	UBA10327
ASV140	Control	Ctr-tf-IIb	0	Bacteria	Planctomycetota	Planctomycetes	Planctomycetales	Planctomycetaceae	UBA10327
ASV140	Non-glacial	LacADNRUFtfa	0	Bacteria	Planctomycetota	Planctomycetes	Planctomycetales	Planctomycetaceae	UBA10327
ASV140	Non-glacial	LacADNRUFtfb	0	Bacteria	Planctomycetota	Planctomycetes	Planctomycetales	Planctomycetaceae	UBA10327
ASV140	Non-glacial	LacADNRUFtfc	0	Bacteria	Planctomycetota	Planctomycetes	Planctomycetales	Planctomycetaceae	UBA10327
ASV140	Glacial	LacAmpRUFtf-a	0	Bacteria	Planctomycetota	Planctomycetes	Planctomycetales	Planctomycetaceae	UBA10327
ASV140	Glacial	LacAmpRUFtf-b	0	Bacteria	Planctomycetota	Planctomycetes	Planctomycetales	Planctomycetaceae	UBA10327
ASV140	Glacial	LacAmpRUFtf-c	0	Bacteria	Planctomycetota	Planctomycetes	Planctomycetales	Planctomycetaceae	UBA10327
ASV1400	Baie de la Table	BdT0-2	0	Bacteria	Proteobacteria	Gammaproteobacteria	UBA5158	UBA5158	SCGC-AG-212-F23
ASV1400	Control	Ctr-tf-IIb	0	Bacteria	Proteobacteria	Gammaproteobacteria	UBA5158	UBA5158	SCGC-AG-212-F23
ASV1400	Non-glacial	LacADNRUFtfa	0	Bacteria	Proteobacteria	Gammaproteobacteria	UBA5158	UBA5158	SCGC-AG-212-F23
ASV1400	Non-glacial	LacADNRUFtfb	0	Bacteria	Proteobacteria	Gammaproteobacteria	UBA5158	UBA5158	SCGC-AG-212-F23
ASV1400	Non-glacial	LacADNRUFtfc	0	Bacteria	Proteobacteria	Gammaproteobacteria	UBA5158	UBA5158	SCGC-AG-212-F23
ASV1400	Glacial	LacAmpRUFtf-a	0	Bacteria	Proteobacteria	Gammaproteobacteria	UBA5158	UBA5158	SCGC-AG-212-F23
ASV1400	Glacial	LacAmpRUFtf-b	0	Bacteria	Proteobacteria	Gammaproteobacteria	UBA5158	UBA5158	SCGC-AG-212-F23
ASV1400	Glacial	LacAmpRUFtf-c	0	Bacteria	Proteobacteria	Gammaproteobacteria	UBA5158	UBA5158	SCGC-AG-212-F23
ASV141	Baie de la Table	BdT0-2	0,003005793	Bacteria	Proteobacteria	Alphaproteobacteria	Puniceispirillales	AAA536-G10	AAA536-G10
ASV141	Glacial	LacAmpRUFtf-a	0,001567272	Bacteria	Proteobacteria	Alphaproteobacteria	Puniceispirillales	AAA536-G10	AAA536-G10
ASV141	Non-glacial	LacADNRUFtfb	0,000400421	Bacteria	Proteobacteria	Alphaproteobacteria	Puniceispirillales	AAA536-G10	AAA536-G10
ASV141	Non-glacial	LacADNRUFtfc	0,00014549	Bacteria	Proteobacteria	Alphaproteobacteria	Puniceispirillales	AAA536-G10	AAA536-G10
ASV141	Control	Ctr-tf-IIb	0,000120198	Bacteria	Proteobacteria	Alphaproteobacteria	Puniceispirillales	AAA536-G10	AAA536-G10
ASV141	Non-glacial	LacADNRUFtfa	0	Bacteria	Proteobacteria	Alphaproteobacteria	Puniceispirillales	AAA536-G10	AAA536-G10

ASV141	Glacial	LacAmpRUFtf-b	0	Bacteria	Proteobacteria	Alphaproteobacteria	Puniceispirillales	AAA536-G10	AAA536-G10
ASV141	Glacial	LacAmpRUFtf-c	0	Bacteria	Proteobacteria	Alphaproteobacteria	Puniceispirillales	AAA536-G10	AAA536-G10
ASV1415	Glacial	LacAmpRUFtf-c	0,000338901	Bacteria	Acidobacteriota	Blastocatellia	Pyrinomonadales	Pyrinomonadaceae	OLB17
ASV1415	Baie de la Table	BdT0-2	0	Bacteria	Acidobacteriota	Blastocatellia	Pyrinomonadales	Pyrinomonadaceae	OLB17
ASV1415	Control	Ctr-tf-IIb	0	Bacteria	Acidobacteriota	Blastocatellia	Pyrinomonadales	Pyrinomonadaceae	OLB17
ASV1415	Non-glacial	LacADNRUFtfa	0	Bacteria	Acidobacteriota	Blastocatellia	Pyrinomonadales	Pyrinomonadaceae	OLB17
ASV1415	Non-glacial	LacADNRUFtfb	0	Bacteria	Acidobacteriota	Blastocatellia	Pyrinomonadales	Pyrinomonadaceae	OLB17
ASV1415	Non-glacial	LacADNRUFtfc	0	Bacteria	Acidobacteriota	Blastocatellia	Pyrinomonadales	Pyrinomonadaceae	OLB17
ASV1415	Glacial	LacAmpRUFtf-a	0	Bacteria	Acidobacteriota	Blastocatellia	Pyrinomonadales	Pyrinomonadaceae	OLB17
ASV1415	Glacial	LacAmpRUFtf-b	0	Bacteria	Acidobacteriota	Blastocatellia	Pyrinomonadales	Pyrinomonadaceae	OLB17
ASV1416	Glacial	LacAmpRUFtf-c	0,000338901	Bacteria	Proteobacteria	Alphaproteobacteria	Acetobacterales	Acetobacteraceae	Belnapia
ASV1416	Baie de la Table	BdT0-2	0	Bacteria	Proteobacteria	Alphaproteobacteria	Acetobacterales	Acetobacteraceae	Belnapia
ASV1416	Control	Ctr-tf-IIb	0	Bacteria	Proteobacteria	Alphaproteobacteria	Acetobacterales	Acetobacteraceae	Belnapia
ASV1416	Non-glacial	LacADNRUFtfa	0	Bacteria	Proteobacteria	Alphaproteobacteria	Acetobacterales	Acetobacteraceae	Belnapia
ASV1416	Non-glacial	LacADNRUFtfb	0	Bacteria	Proteobacteria	Alphaproteobacteria	Acetobacterales	Acetobacteraceae	Belnapia
ASV1416	Non-glacial	LacADNRUFtfc	0	Bacteria	Proteobacteria	Alphaproteobacteria	Acetobacterales	Acetobacteraceae	Belnapia
ASV1416	Glacial	LacAmpRUFtf-a	0	Bacteria	Proteobacteria	Alphaproteobacteria	Acetobacterales	Acetobacteraceae	Belnapia
ASV1416	Glacial	LacAmpRUFtf-b	0	Bacteria	Proteobacteria	Alphaproteobacteria	Acetobacterales	Acetobacteraceae	Belnapia
ASV1417	Glacial	LacAmpRUFtf-c	0,000338901	Bacteria	Actinobacteriota	Actinobacteria	Actinomycetales	Actinomycetaceae	Actinomyces
ASV1417	Baie de la Table	BdT0-2	0	Bacteria	Actinobacteriota	Actinobacteria	Actinomycetales	Actinomycetaceae	Actinomyces
ASV1417	Control	Ctr-tf-IIb	0	Bacteria	Actinobacteriota	Actinobacteria	Actinomycetales	Actinomycetaceae	Actinomyces
ASV1417	Non-glacial	LacADNRUFtfa	0	Bacteria	Actinobacteriota	Actinobacteria	Actinomycetales	Actinomycetaceae	Actinomyces
ASV1417	Non-glacial	LacADNRUFtfb	0	Bacteria	Actinobacteriota	Actinobacteria	Actinomycetales	Actinomycetaceae	Actinomyces
ASV1417	Non-glacial	LacADNRUFtfc	0	Bacteria	Actinobacteriota	Actinobacteria	Actinomycetales	Actinomycetaceae	Actinomyces
ASV1417	Glacial	LacAmpRUFtf-a	0	Bacteria	Actinobacteriota	Actinobacteria	Actinomycetales	Actinomycetaceae	Actinomyces
ASV1417	Glacial	LacAmpRUFtf-b	0	Bacteria	Actinobacteriota	Actinobacteria	Actinomycetales	Actinomycetaceae	Actinomyces
ASV142	Glacial	LacAmpRUFtf-a	0,002893425	Bacteria	Actinobacteriota	Acidimicrobiia	Microtrichales	Ilumatobacteraceae	Ilumatobacter_A
ASV142	Non-glacial	LacADNRUFtfc	0,002109602	Bacteria	Actinobacteriota	Acidimicrobiia	Microtrichales	Ilumatobacteraceae	Ilumatobacter_A
ASV142	Glacial	LacAmpRUFtf-b	0,001835655	Bacteria	Actinobacteriota	Acidimicrobiia	Microtrichales	Ilumatobacteraceae	Ilumatobacter_A
ASV142	Non-glacial	LacADNRUFtfa	0,001616703	Bacteria	Actinobacteriota	Acidimicrobiia	Microtrichales	Ilumatobacteraceae	Ilumatobacter_A
ASV142	Non-glacial	LacADNRUFtfb	0,001074816	Bacteria	Actinobacteriota	Acidimicrobiia	Microtrichales	Ilumatobacteraceae	Ilumatobacter_A
ASV142	Glacial	LacAmpRUFtf-c	0,000919874	Bacteria	Actinobacteriota	Acidimicrobiia	Microtrichales	Ilumatobacteraceae	Ilumatobacter_A
ASV142	Control	Ctr-tf-IIb	0,000713172	Bacteria	Actinobacteriota	Acidimicrobiia	Microtrichales	Ilumatobacteraceae	Ilumatobacter_A
ASV142	Baie de la Table	BdT0-2	0	Bacteria	Actinobacteriota	Acidimicrobiia	Microtrichales	Ilumatobacteraceae	Ilumatobacter_A

ASV1422	Baie de la Table	BdT0-2	0	Bacteria	Dependentiae	Babeliae	Babeliales	UBA12411	UBA12411
ASV1422	Control	Ctr-tf-IIb	0	Bacteria	Dependentiae	Babeliae	Babeliales	UBA12411	UBA12411
ASV1422	Non-glacial	LacADNRUFtfa	0	Bacteria	Dependentiae	Babeliae	Babeliales	UBA12411	UBA12411
ASV1422	Non-glacial	LacADNRUFtfb	0	Bacteria	Dependentiae	Babeliae	Babeliales	UBA12411	UBA12411
ASV1422	Non-glacial	LacADNRUFtfc	0	Bacteria	Dependentiae	Babeliae	Babeliales	UBA12411	UBA12411
ASV1422	Glacial	LacAmpRUFtf-a	0	Bacteria	Dependentiae	Babeliae	Babeliales	UBA12411	UBA12411
ASV1422	Glacial	LacAmpRUFtf-b	0	Bacteria	Dependentiae	Babeliae	Babeliales	UBA12411	UBA12411
ASV1422	Glacial	LacAmpRUFtf-c	0	Bacteria	Dependentiae	Babeliae	Babeliales	UBA12411	UBA12411
ASV1425	Baie de la Table	BdT0-2	0	Bacteria	Omnitrophota	Omnitrophia	Omnitrophales	UBA2337	XYB12-FULL-50-7
ASV1425	Control	Ctr-tf-IIb	0	Bacteria	Omnitrophota	Omnitrophia	Omnitrophales	UBA2337	XYB12-FULL-50-7
ASV1425	Non-glacial	LacADNRUFtfa	0	Bacteria	Omnitrophota	Omnitrophia	Omnitrophales	UBA2337	XYB12-FULL-50-7
ASV1425	Non-glacial	LacADNRUFtfb	0	Bacteria	Omnitrophota	Omnitrophia	Omnitrophales	UBA2337	XYB12-FULL-50-7
ASV1425	Non-glacial	LacADNRUFtfc	0	Bacteria	Omnitrophota	Omnitrophia	Omnitrophales	UBA2337	XYB12-FULL-50-7
ASV1425	Glacial	LacAmpRUFtf-a	0	Bacteria	Omnitrophota	Omnitrophia	Omnitrophales	UBA2337	XYB12-FULL-50-7
ASV1425	Glacial	LacAmpRUFtf-b	0	Bacteria	Omnitrophota	Omnitrophia	Omnitrophales	UBA2337	XYB12-FULL-50-7
ASV1425	Glacial	LacAmpRUFtf-c	0	Bacteria	Omnitrophota	Omnitrophia	Omnitrophales	UBA2337	XYB12-FULL-50-7
ASV1427	Baie de la Table	BdT0-2	0	Bacteria	Proteobacteria	Alphaproteobacteria	Caulobacterales	Hyphomonadaceae	Aquidulcibacter
ASV1427	Control	Ctr-tf-IIb	0	Bacteria	Proteobacteria	Alphaproteobacteria	Caulobacterales	Hyphomonadaceae	Aquidulcibacter
ASV1427	Non-glacial	LacADNRUFtfa	0	Bacteria	Proteobacteria	Alphaproteobacteria	Caulobacterales	Hyphomonadaceae	Aquidulcibacter
ASV1427	Non-glacial	LacADNRUFtfb	0	Bacteria	Proteobacteria	Alphaproteobacteria	Caulobacterales	Hyphomonadaceae	Aquidulcibacter
ASV1427	Non-glacial	LacADNRUFtfc	0	Bacteria	Proteobacteria	Alphaproteobacteria	Caulobacterales	Hyphomonadaceae	Aquidulcibacter
ASV1427	Glacial	LacAmpRUFtf-a	0	Bacteria	Proteobacteria	Alphaproteobacteria	Caulobacterales	Hyphomonadaceae	Aquidulcibacter
ASV1427	Glacial	LacAmpRUFtf-b	0	Bacteria	Proteobacteria	Alphaproteobacteria	Caulobacterales	Hyphomonadaceae	Aquidulcibacter
ASV1427	Glacial	LacAmpRUFtf-c	0	Bacteria	Proteobacteria	Alphaproteobacteria	Caulobacterales	Hyphomonadaceae	Aquidulcibacter
ASV143	Glacial	LacAmpRUFtf-c	0,019365771	Bacteria	Firmicutes	Bacilli_A	Paenibacillales	Paenibacillaceae	Paenibacillus_G
ASV143	Non-glacial	LacADNRUFtfb	0,009884089	Bacteria	Firmicutes	Bacilli_A	Paenibacillales	Paenibacillaceae	Paenibacillus_G
ASV143	Glacial	LacAmpRUFtf-b	0,005506965	Bacteria	Firmicutes	Bacilli_A	Paenibacillales	Paenibacillaceae	Paenibacillus_G
ASV143	Non-glacial	LacADNRUFtfc	0,001721629	Bacteria	Firmicutes	Bacilli_A	Paenibacillales	Paenibacillaceae	Paenibacillus_G
ASV143	Glacial	LacAmpRUFtf-a	0,000602797	Bacteria	Firmicutes	Bacilli_A	Paenibacillales	Paenibacillaceae	Paenibacillus_G
ASV143	Non-glacial	LacADNRUFtfa	0,000138575	Bacteria	Firmicutes	Bacilli_A	Paenibacillales	Paenibacillaceae	Paenibacillus_G
ASV143	Baie de la Table	BdT0-2	0	Bacteria	Firmicutes	Bacilli_A	Paenibacillales	Paenibacillaceae	Paenibacillus_G
ASV143	Control	Ctr-tf-IIb	0	Bacteria	Firmicutes	Bacilli_A	Paenibacillales	Paenibacillaceae	Paenibacillus_G
ASV144	Non-glacial	LacADNRUFtfb	0,000273973	Bacteria	Proteobacteria	Gammaproteobacteria	Betaproteobacteriales	Methylophilaceae	Methylotenera
ASV144	Baie de la Table	BdT0-2	0	Bacteria	Proteobacteria	Gammaproteobacteria	Betaproteobacteriales	Methylophilaceae	Methylotenera

ASV144	Control	Ctr-tf-IIb	0	Bacteria	Proteobacteria	Gammaproteobacteria	Betaproteobacteriales	Methylophilaceae	Methylotenera
ASV144	Non-glacial	LacADNRUFtfa	0	Bacteria	Proteobacteria	Gammaproteobacteria	Betaproteobacteriales	Methylophilaceae	Methylotenera
ASV144	Non-glacial	LacADNRUFtfc	0	Bacteria	Proteobacteria	Gammaproteobacteria	Betaproteobacteriales	Methylophilaceae	Methylotenera
ASV144	Glacial	LacAmpRUFtf-a	0	Bacteria	Proteobacteria	Gammaproteobacteria	Betaproteobacteriales	Methylophilaceae	Methylotenera
ASV144	Glacial	LacAmpRUFtf-b	0	Bacteria	Proteobacteria	Gammaproteobacteria	Betaproteobacteriales	Methylophilaceae	Methylotenera
ASV144	Glacial	LacAmpRUFtf-c	0	Bacteria	Proteobacteria	Gammaproteobacteria	Betaproteobacteriales	Methylophilaceae	Methylotenera
ASV1450	Non-glacial	LacADNRUFtfc	4,85E-05	Bacteria	Proteobacteria	Gammaproteobacteria	Diplorickettsiales	Diplorickettsiaceae	Rickettsiella
ASV1450	Baie de la Table	BdT0-2	0	Bacteria	Proteobacteria	Gammaproteobacteria	Diplorickettsiales	Diplorickettsiaceae	Rickettsiella
ASV1450	Control	Ctr-tf-IIb	0	Bacteria	Proteobacteria	Gammaproteobacteria	Diplorickettsiales	Diplorickettsiaceae	Rickettsiella
ASV1450	Non-glacial	LacADNRUFtfa	0	Bacteria	Proteobacteria	Gammaproteobacteria	Diplorickettsiales	Diplorickettsiaceae	Rickettsiella
ASV1450	Non-glacial	LacADNRUFtfb	0	Bacteria	Proteobacteria	Gammaproteobacteria	Diplorickettsiales	Diplorickettsiaceae	Rickettsiella
ASV1450	Glacial	LacAmpRUFtf-a	0	Bacteria	Proteobacteria	Gammaproteobacteria	Diplorickettsiales	Diplorickettsiaceae	Rickettsiella
ASV1450	Glacial	LacAmpRUFtf-b	0	Bacteria	Proteobacteria	Gammaproteobacteria	Diplorickettsiales	Diplorickettsiaceae	Rickettsiella
ASV1450	Glacial	LacAmpRUFtf-c	0	Bacteria	Proteobacteria	Gammaproteobacteria	Diplorickettsiales	Diplorickettsiaceae	Rickettsiella
ASV1452	Baie de la Table	BdT0-2	0	Bacteria	Bacteroidota	Bacteroidia	Cytophagales	Cyclobacteriaceae	ELB16-189
ASV1452	Control	Ctr-tf-IIb	0	Bacteria	Bacteroidota	Bacteroidia	Cytophagales	Cyclobacteriaceae	ELB16-189
ASV1452	Non-glacial	LacADNRUFtfa	0	Bacteria	Bacteroidota	Bacteroidia	Cytophagales	Cyclobacteriaceae	ELB16-189
ASV1452	Non-glacial	LacADNRUFtfb	0	Bacteria	Bacteroidota	Bacteroidia	Cytophagales	Cyclobacteriaceae	ELB16-189
ASV1452	Non-glacial	LacADNRUFtfc	0	Bacteria	Bacteroidota	Bacteroidia	Cytophagales	Cyclobacteriaceae	ELB16-189
ASV1452	Glacial	LacAmpRUFtf-a	0	Bacteria	Bacteroidota	Bacteroidia	Cytophagales	Cyclobacteriaceae	ELB16-189
ASV1452	Glacial	LacAmpRUFtf-b	0	Bacteria	Bacteroidota	Bacteroidia	Cytophagales	Cyclobacteriaceae	ELB16-189
ASV1452	Glacial	LacAmpRUFtf-c	0	Bacteria	Bacteroidota	Bacteroidia	Cytophagales	Cyclobacteriaceae	ELB16-189
ASV1455	Baie de la Table	BdT0-2	0	Bacteria	Desulfobacterota	Desulfobulbia	Desulfobulbales	Desulfocapsaceae	Desulfotalea
ASV1455	Control	Ctr-tf-IIb	0	Bacteria	Desulfobacterota	Desulfobulbia	Desulfobulbales	Desulfocapsaceae	Desulfotalea
ASV1455	Non-glacial	LacADNRUFtfa	0	Bacteria	Desulfobacterota	Desulfobulbia	Desulfobulbales	Desulfocapsaceae	Desulfotalea
ASV1455	Non-glacial	LacADNRUFtfb	0	Bacteria	Desulfobacterota	Desulfobulbia	Desulfobulbales	Desulfocapsaceae	Desulfotalea
ASV1455	Non-glacial	LacADNRUFtfc	0	Bacteria	Desulfobacterota	Desulfobulbia	Desulfobulbales	Desulfocapsaceae	Desulfotalea
ASV1455	Glacial	LacAmpRUFtf-a	0	Bacteria	Desulfobacterota	Desulfobulbia	Desulfobulbales	Desulfocapsaceae	Desulfotalea
ASV1455	Glacial	LacAmpRUFtf-b	0	Bacteria	Desulfobacterota	Desulfobulbia	Desulfobulbales	Desulfocapsaceae	Desulfotalea
ASV1455	Glacial	LacAmpRUFtf-c	0	Bacteria	Desulfobacterota	Desulfobulbia	Desulfobulbales	Desulfocapsaceae	Desulfotalea
ASV1458	Baie de la Table	BdT0-2	0	Bacteria	Omnitrophota	koll11	GIF10	UBA6249	GCA-002774445
ASV1458	Control	Ctr-tf-IIb	0	Bacteria	Omnitrophota	koll11	GIF10	UBA6249	GCA-002774445
ASV1458	Non-glacial	LacADNRUFtfa	0	Bacteria	Omnitrophota	koll11	GIF10	UBA6249	GCA-002774445
ASV1458	Non-glacial	LacADNRUFtfb	0	Bacteria	Omnitrophota	koll11	GIF10	UBA6249	GCA-002774445

ASV1458	Non-glacial	LacADNRUFtfc	0	Bacteria	Omnitrophota	koll11	GIF10	UBA6249	GCA-002774445
ASV1458	Glacial	LacAmpRUFtf-a	0	Bacteria	Omnitrophota	koll11	GIF10	UBA6249	GCA-002774445
ASV1458	Glacial	LacAmpRUFtf-b	0	Bacteria	Omnitrophota	koll11	GIF10	UBA6249	GCA-002774445
ASV1458	Glacial	LacAmpRUFtf-c	0	Bacteria	Omnitrophota	koll11	GIF10	UBA6249	GCA-002774445
ASV1459	Baie de la Table	BdT0-2	0	Bacteria	Proteobacteria	Gammaproteobacteria	Berkiellales	Berkiellaceae	Berkiella
ASV1459	Control	Ctr-tf-IIb	0	Bacteria	Proteobacteria	Gammaproteobacteria	Berkiellales	Berkiellaceae	Berkiella
ASV1459	Non-glacial	LacADNRUFtfa	0	Bacteria	Proteobacteria	Gammaproteobacteria	Berkiellales	Berkiellaceae	Berkiella
ASV1459	Non-glacial	LacADNRUFtfb	0	Bacteria	Proteobacteria	Gammaproteobacteria	Berkiellales	Berkiellaceae	Berkiella
ASV1459	Non-glacial	LacADNRUFtfc	0	Bacteria	Proteobacteria	Gammaproteobacteria	Berkiellales	Berkiellaceae	Berkiella
ASV1459	Glacial	LacAmpRUFtf-a	0	Bacteria	Proteobacteria	Gammaproteobacteria	Berkiellales	Berkiellaceae	Berkiella
ASV1459	Glacial	LacAmpRUFtf-b	0	Bacteria	Proteobacteria	Gammaproteobacteria	Berkiellales	Berkiellaceae	Berkiella
ASV1459	Glacial	LacAmpRUFtf-c	0	Bacteria	Proteobacteria	Gammaproteobacteria	Berkiellales	Berkiellaceae	Berkiella
ASV146	Glacial	LacAmpRUFtf-c	0,000677802	Bacteria	Proteobacteria	Alphaproteobacteria	Caulobacterales	Caulobacteraceae	Caulobacter
ASV146	Control	Ctr-tf-IIb	5,61E-05	Bacteria	Proteobacteria	Alphaproteobacteria	Caulobacterales	Caulobacteraceae	Caulobacter
ASV146	Baie de la Table	BdT0-2	0	Bacteria	Proteobacteria	Alphaproteobacteria	Caulobacterales	Caulobacteraceae	Caulobacter
ASV146	Non-glacial	LacADNRUFtfa	0	Bacteria	Proteobacteria	Alphaproteobacteria	Caulobacterales	Caulobacteraceae	Caulobacter
ASV146	Non-glacial	LacADNRUFtfb	0	Bacteria	Proteobacteria	Alphaproteobacteria	Caulobacterales	Caulobacteraceae	Caulobacter
ASV146	Non-glacial	LacADNRUFtfc	0	Bacteria	Proteobacteria	Alphaproteobacteria	Caulobacterales	Caulobacteraceae	Caulobacter
ASV146	Glacial	LacAmpRUFtf-a	0	Bacteria	Proteobacteria	Alphaproteobacteria	Caulobacterales	Caulobacteraceae	Caulobacter
ASV146	Glacial	LacAmpRUFtf-b	0	Bacteria	Proteobacteria	Alphaproteobacteria	Caulobacterales	Caulobacteraceae	Caulobacter
ASV1476	Non-glacial	LacADNRUFtfb	0,000126449	Bacteria	Proteobacteria	Alphaproteobacteria	Rhodospirillales	Rhodospirillaceae	Caenispirillum
ASV1476	Baie de la Table	BdT0-2	0	Bacteria	Proteobacteria	Alphaproteobacteria	Rhodospirillales	Rhodospirillaceae	Caenispirillum
ASV1476	Control	Ctr-tf-IIb	0	Bacteria	Proteobacteria	Alphaproteobacteria	Rhodospirillales	Rhodospirillaceae	Caenispirillum
ASV1476	Non-glacial	LacADNRUFtfa	0	Bacteria	Proteobacteria	Alphaproteobacteria	Rhodospirillales	Rhodospirillaceae	Caenispirillum
ASV1476	Non-glacial	LacADNRUFtfc	0	Bacteria	Proteobacteria	Alphaproteobacteria	Rhodospirillales	Rhodospirillaceae	Caenispirillum
ASV1476	Glacial	LacAmpRUFtf-a	0	Bacteria	Proteobacteria	Alphaproteobacteria	Rhodospirillales	Rhodospirillaceae	Caenispirillum
ASV1476	Glacial	LacAmpRUFtf-b	0	Bacteria	Proteobacteria	Alphaproteobacteria	Rhodospirillales	Rhodospirillaceae	Caenispirillum
ASV1476	Glacial	LacAmpRUFtf-c	0	Bacteria	Proteobacteria	Alphaproteobacteria	Rhodospirillales	Rhodospirillaceae	Caenispirillum
ASV1477	Non-glacial	LacADNRUFtfb	0,000126449	Bacteria	Actinobacteriota	Actinobacteria	Actinomycetales	Bifidobacteriaceae	Bifidobacterium
ASV1477	Baie de la Table	BdT0-2	0	Bacteria	Actinobacteriota	Actinobacteria	Actinomycetales	Bifidobacteriaceae	Bifidobacterium
ASV1477	Control	Ctr-tf-IIb	0	Bacteria	Actinobacteriota	Actinobacteria	Actinomycetales	Bifidobacteriaceae	Bifidobacterium
ASV1477	Non-glacial	LacADNRUFtfa	0	Bacteria	Actinobacteriota	Actinobacteria	Actinomycetales	Bifidobacteriaceae	Bifidobacterium
ASV1477	Non-glacial	LacADNRUFtfc	0	Bacteria	Actinobacteriota	Actinobacteria	Actinomycetales	Bifidobacteriaceae	Bifidobacterium
ASV1477	Glacial	LacAmpRUFtf-a	0	Bacteria	Actinobacteriota	Actinobacteria	Actinomycetales	Bifidobacteriaceae	Bifidobacterium

ASV1477	Glacial	LacAmpRUFtf-b	0	Bacteria	Actinobacteriota	Actinobacteria	Actinomycetales	Bifidobacteriaceae	Bifidobacterium
ASV1477	Glacial	LacAmpRUFtf-c	0	Bacteria	Actinobacteriota	Actinobacteria	Actinomycetales	Bifidobacteriaceae	Bifidobacterium
ASV148	Glacial	LacAmpRUFtf-a	0,009765311	Bacteria	Proteobacteria	Gammaproteobacteria	Enterobacterales	Alteromonadaceae	Alteromonas
ASV148	Glacial	LacAmpRUFtf-b	0,005614944	Bacteria	Proteobacteria	Gammaproteobacteria	Enterobacterales	Alteromonadaceae	Alteromonas
ASV148	Glacial	LacAmpRUFtf-c	0,001355604	Bacteria	Proteobacteria	Gammaproteobacteria	Enterobacterales	Alteromonadaceae	Alteromonas
ASV148	Non-glacial	LacADNRUFtfc	9,70E-05	Bacteria	Proteobacteria	Gammaproteobacteria	Enterobacterales	Alteromonadaceae	Alteromonas
ASV148	Baie de la Table	BdT0-2	0	Bacteria	Proteobacteria	Gammaproteobacteria	Enterobacterales	Alteromonadaceae	Alteromonas
ASV148	Control	Ctr-tf-IIb	0	Bacteria	Proteobacteria	Gammaproteobacteria	Enterobacterales	Alteromonadaceae	Alteromonas
ASV148	Non-glacial	LacADNRUFtfa	0	Bacteria	Proteobacteria	Gammaproteobacteria	Enterobacterales	Alteromonadaceae	Alteromonas
ASV148	Non-glacial	LacADNRUFtfb	0	Bacteria	Proteobacteria	Gammaproteobacteria	Enterobacterales	Alteromonadaceae	Alteromonas
ASV1489	Baie de la Table	BdT0-2	0	Bacteria	Firmicutes	Bacilli	Bacillales	Bacillaceae_H	Bacillus_C
ASV1489	Control	Ctr-tf-IIb	0	Bacteria	Firmicutes	Bacilli	Bacillales	Bacillaceae_H	Bacillus_C
ASV1489	Non-glacial	LacADNRUFtfa	0	Bacteria	Firmicutes	Bacilli	Bacillales	Bacillaceae_H	Bacillus_C
ASV1489	Non-glacial	LacADNRUFtfb	0	Bacteria	Firmicutes	Bacilli	Bacillales	Bacillaceae_H	Bacillus_C
ASV1489	Non-glacial	LacADNRUFtfc	0	Bacteria	Firmicutes	Bacilli	Bacillales	Bacillaceae_H	Bacillus_C
ASV1489	Glacial	LacAmpRUFtf-a	0	Bacteria	Firmicutes	Bacilli	Bacillales	Bacillaceae_H	Bacillus_C
ASV1489	Glacial	LacAmpRUFtf-b	0	Bacteria	Firmicutes	Bacilli	Bacillales	Bacillaceae_H	Bacillus_C
ASV1489	Glacial	LacAmpRUFtf-c	0	Bacteria	Firmicutes	Bacilli	Bacillales	Bacillaceae_H	Bacillus_C
ASV149	Baie de la Table	BdT0-2	0,004098809	Bacteria	Bacteroidota	Bacteroidia	Flavobacteriales	Flavobacteriaceae	Lutibacter
ASV149	Non-glacial	LacADNRUFtfa	0,000739064	Bacteria	Bacteroidota	Bacteroidia	Flavobacteriales	Flavobacteriaceae	Lutibacter
ASV149	Non-glacial	LacADNRUFtfb	0,000547945	Bacteria	Bacteroidota	Bacteroidia	Flavobacteriales	Flavobacteriaceae	Lutibacter
ASV149	Control	Ctr-tf-IIb	0,000232382	Bacteria	Bacteroidota	Bacteroidia	Flavobacteriales	Flavobacteriaceae	Lutibacter
ASV149	Non-glacial	LacADNRUFtfc	0	Bacteria	Bacteroidota	Bacteroidia	Flavobacteriales	Flavobacteriaceae	Lutibacter
ASV149	Glacial	LacAmpRUFtf-a	0	Bacteria	Bacteroidota	Bacteroidia	Flavobacteriales	Flavobacteriaceae	Lutibacter
ASV149	Glacial	LacAmpRUFtf-b	0	Bacteria	Bacteroidota	Bacteroidia	Flavobacteriales	Flavobacteriaceae	Lutibacter
ASV149	Glacial	LacAmpRUFtf-c	0	Bacteria	Bacteroidota	Bacteroidia	Flavobacteriales	Flavobacteriaceae	Lutibacter
ASV1491	Baie de la Table	BdT0-2	0	Bacteria	Actinobacteriota	Actinobacteria	Corynebacteriales	Corynebacteriaceae	Williamsia
ASV1491	Control	Ctr-tf-IIb	0	Bacteria	Actinobacteriota	Actinobacteria	Corynebacteriales	Corynebacteriaceae	Williamsia
ASV1491	Non-glacial	LacADNRUFtfa	0	Bacteria	Actinobacteriota	Actinobacteria	Corynebacteriales	Corynebacteriaceae	Williamsia
ASV1491	Non-glacial	LacADNRUFtfb	0	Bacteria	Actinobacteriota	Actinobacteria	Corynebacteriales	Corynebacteriaceae	Williamsia
ASV1491	Non-glacial	LacADNRUFtfc	0	Bacteria	Actinobacteriota	Actinobacteria	Corynebacteriales	Corynebacteriaceae	Williamsia
ASV1491	Glacial	LacAmpRUFtf-a	0	Bacteria	Actinobacteriota	Actinobacteria	Corynebacteriales	Corynebacteriaceae	Williamsia
ASV1491	Glacial	LacAmpRUFtf-b	0	Bacteria	Actinobacteriota	Actinobacteria	Corynebacteriales	Corynebacteriaceae	Williamsia
ASV1491	Glacial	LacAmpRUFtf-c	0	Bacteria	Actinobacteriota	Actinobacteria	Corynebacteriales	Corynebacteriaceae	Williamsia

ASV1499	Baie de la Table	BdT0-2	0	Bacteria	Dependentiae	Babeliae	Babeliales	GCA-2401785	GCA-2401785
ASV1499	Control	Ctr-tf-IIb	0	Bacteria	Dependentiae	Babeliae	Babeliales	GCA-2401785	GCA-2401785
ASV1499	Non-glacial	LacADNRUFtfa	0	Bacteria	Dependentiae	Babeliae	Babeliales	GCA-2401785	GCA-2401785
ASV1499	Non-glacial	LacADNRUFtfb	0	Bacteria	Dependentiae	Babeliae	Babeliales	GCA-2401785	GCA-2401785
ASV1499	Non-glacial	LacADNRUFtfc	0	Bacteria	Dependentiae	Babeliae	Babeliales	GCA-2401785	GCA-2401785
ASV1499	Glacial	LacAmpRUFtf-a	0	Bacteria	Dependentiae	Babeliae	Babeliales	GCA-2401785	GCA-2401785
ASV1499	Glacial	LacAmpRUFtf-b	0	Bacteria	Dependentiae	Babeliae	Babeliales	GCA-2401785	GCA-2401785
ASV1499	Glacial	LacAmpRUFtf-c	0	Bacteria	Dependentiae	Babeliae	Babeliales	GCA-2401785	GCA-2401785
ASV15	Control	Ctr-tf-IIb	0,105998261	Bacteria	Proteobacteria	Alphaproteobacteria	Sphingomonadales	Sphingomonadaceae	Sphingomonas
ASV15	Glacial	LacAmpRUFtf-b	0,01090595	Bacteria	Proteobacteria	Alphaproteobacteria	Sphingomonadales	Sphingomonadaceae	Sphingomonas
ASV15	Glacial	LacAmpRUFtf-c	0,006245461	Bacteria	Proteobacteria	Alphaproteobacteria	Sphingomonadales	Sphingomonadaceae	Sphingomonas
ASV15	Non-glacial	LacADNRUFtfb	0,001032666	Bacteria	Proteobacteria	Alphaproteobacteria	Sphingomonadales	Sphingomonadaceae	Sphingomonas
ASV15	Glacial	LacAmpRUFtf-a	0,000482238	Bacteria	Proteobacteria	Alphaproteobacteria	Sphingomonadales	Sphingomonadaceae	Sphingomonas
ASV15	Non-glacial	LacADNRUFtfa	0,000369532	Bacteria	Proteobacteria	Alphaproteobacteria	Sphingomonadales	Sphingomonadaceae	Sphingomonas
ASV15	Non-glacial	LacADNRUFtfc	0,000218235	Bacteria	Proteobacteria	Alphaproteobacteria	Sphingomonadales	Sphingomonadaceae	Sphingomonas
ASV15	Baie de la Table	BdT0-2	0	Bacteria	Proteobacteria	Alphaproteobacteria	Sphingomonadales	Sphingomonadaceae	Sphingomonas
ASV150	Non-glacial	LacADNRUFtfa	0,000277149	Bacteria	Proteobacteria	Alphaproteobacteria	Pelagibacterales	Pelagibacteraceae	Fonsibacter
ASV150	Control	Ctr-tf-IIb	5,21E-05	Bacteria	Proteobacteria	Alphaproteobacteria	Pelagibacterales	Pelagibacteraceae	Fonsibacter
ASV150	Baie de la Table	BdT0-2	0	Bacteria	Proteobacteria	Alphaproteobacteria	Pelagibacterales	Pelagibacteraceae	Fonsibacter
ASV150	Non-glacial	LacADNRUFtfb	0	Bacteria	Proteobacteria	Alphaproteobacteria	Pelagibacterales	Pelagibacteraceae	Fonsibacter
ASV150	Non-glacial	LacADNRUFtfc	0	Bacteria	Proteobacteria	Alphaproteobacteria	Pelagibacterales	Pelagibacteraceae	Fonsibacter
ASV150	Glacial	LacAmpRUFtf-a	0	Bacteria	Proteobacteria	Alphaproteobacteria	Pelagibacterales	Pelagibacteraceae	Fonsibacter
ASV150	Glacial	LacAmpRUFtf-b	0	Bacteria	Proteobacteria	Alphaproteobacteria	Pelagibacterales	Pelagibacteraceae	Fonsibacter
ASV150	Glacial	LacAmpRUFtf-c	0	Bacteria	Proteobacteria	Alphaproteobacteria	Pelagibacterales	Pelagibacteraceae	Fonsibacter
ASV1511	Glacial	LacAmpRUFtf-a	0,000241119	Bacteria	Bacteroidota	Bacteroidia	Cytophagales	Marinoscillaceae	Marinoscillum
ASV1511	Baie de la Table	BdT0-2	0	Bacteria	Bacteroidota	Bacteroidia	Cytophagales	Marinoscillaceae	Marinoscillum
ASV1511	Control	Ctr-tf-IIb	0	Bacteria	Bacteroidota	Bacteroidia	Cytophagales	Marinoscillaceae	Marinoscillum
ASV1511	Non-glacial	LacADNRUFtfa	0	Bacteria	Bacteroidota	Bacteroidia	Cytophagales	Marinoscillaceae	Marinoscillum
ASV1511	Non-glacial	LacADNRUFtfb	0	Bacteria	Bacteroidota	Bacteroidia	Cytophagales	Marinoscillaceae	Marinoscillum
ASV1511	Non-glacial	LacADNRUFtfc	0	Bacteria	Bacteroidota	Bacteroidia	Cytophagales	Marinoscillaceae	Marinoscillum
ASV1511	Glacial	LacAmpRUFtf-b	0	Bacteria	Bacteroidota	Bacteroidia	Cytophagales	Marinoscillaceae	Marinoscillum
ASV1511	Glacial	LacAmpRUFtf-c	0	Bacteria	Bacteroidota	Bacteroidia	Cytophagales	Marinoscillaceae	Marinoscillum
ASV1514	Glacial	LacAmpRUFtf-a	0,000241119	Archaea	Thermoplasmato	MGII	MGII	MGIIA	UBA253
ASV1514	Baie de la Table	BdT0-2	0	Archaea	Thermoplasmato	MGII	MGII	MGIIA	UBA253

ASV1514	Control	Ctr-tf-IIb	0	Archaea	Thermoplasmat	MGII	MGII	MGIIA	UBA253
ASV1514	Non-glacial	LacADNRUFtfa	0	Archaea	Thermoplasmat	MGII	MGII	MGIIA	UBA253
ASV1514	Non-glacial	LacADNRUFtfb	0	Archaea	Thermoplasmat	MGII	MGII	MGIIA	UBA253
ASV1514	Non-glacial	LacADNRUFtfc	0	Archaea	Thermoplasmat	MGII	MGII	MGIIA	UBA253
ASV1514	Glacial	LacAmpRUFtf-b	0	Archaea	Thermoplasmat	MGII	MGII	MGIIA	UBA253
ASV1514	Glacial	LacAmpRUFtf-c	0	Archaea	Thermoplasmat	MGII	MGII	MGIIA	UBA253
ASV1540	Baie de la Table	BdT0-2	0	Bacteria	Verrucomicrobio	Verrucomicrobiae	Opitutales	Opitutaceae	Lacunisphaera
ASV1540	Control	Ctr-tf-IIb	0	Bacteria	Verrucomicrobio	Verrucomicrobiae	Opitutales	Opitutaceae	Lacunisphaera
ASV1540	Non-glacial	LacADNRUFtfa	0	Bacteria	Verrucomicrobio	Verrucomicrobiae	Opitutales	Opitutaceae	Lacunisphaera
ASV1540	Non-glacial	LacADNRUFtfb	0	Bacteria	Verrucomicrobio	Verrucomicrobiae	Opitutales	Opitutaceae	Lacunisphaera
ASV1540	Non-glacial	LacADNRUFtfc	0	Bacteria	Verrucomicrobio	Verrucomicrobiae	Opitutales	Opitutaceae	Lacunisphaera
ASV1540	Glacial	LacAmpRUFtf-a	0	Bacteria	Verrucomicrobio	Verrucomicrobiae	Opitutales	Opitutaceae	Lacunisphaera
ASV1540	Glacial	LacAmpRUFtf-b	0	Bacteria	Verrucomicrobio	Verrucomicrobiae	Opitutales	Opitutaceae	Lacunisphaera
ASV1540	Glacial	LacAmpRUFtf-c	0	Bacteria	Verrucomicrobio	Verrucomicrobiae	Opitutales	Opitutaceae	Lacunisphaera
ASV1556	Baie de la Table	BdT0-2	0	Bacteria	Bdellovibrionota	Oligoflexia	Oligoflexales	bin106	bin106
ASV1556	Control	Ctr-tf-IIb	0	Bacteria	Bdellovibrionota	Oligoflexia	Oligoflexales	bin106	bin106
ASV1556	Non-glacial	LacADNRUFtfa	0	Bacteria	Bdellovibrionota	Oligoflexia	Oligoflexales	bin106	bin106
ASV1556	Non-glacial	LacADNRUFtfb	0	Bacteria	Bdellovibrionota	Oligoflexia	Oligoflexales	bin106	bin106
ASV1556	Non-glacial	LacADNRUFtfc	0	Bacteria	Bdellovibrionota	Oligoflexia	Oligoflexales	bin106	bin106
ASV1556	Glacial	LacAmpRUFtf-a	0	Bacteria	Bdellovibrionota	Oligoflexia	Oligoflexales	bin106	bin106
ASV1556	Glacial	LacAmpRUFtf-b	0	Bacteria	Bdellovibrionota	Oligoflexia	Oligoflexales	bin106	bin106
ASV1556	Glacial	LacAmpRUFtf-c	0	Bacteria	Bdellovibrionota	Oligoflexia	Oligoflexales	bin106	bin106
ASV1557	Baie de la Table	BdT0-2	0	Bacteria	Bacteroidota	Kapabacteria	Kapabacteriales	Kapabacteriaceae	OLB6
ASV1557	Control	Ctr-tf-IIb	0	Bacteria	Bacteroidota	Kapabacteria	Kapabacteriales	Kapabacteriaceae	OLB6
ASV1557	Non-glacial	LacADNRUFtfa	0	Bacteria	Bacteroidota	Kapabacteria	Kapabacteriales	Kapabacteriaceae	OLB6
ASV1557	Non-glacial	LacADNRUFtfb	0	Bacteria	Bacteroidota	Kapabacteria	Kapabacteriales	Kapabacteriaceae	OLB6
ASV1557	Non-glacial	LacADNRUFtfc	0	Bacteria	Bacteroidota	Kapabacteria	Kapabacteriales	Kapabacteriaceae	OLB6
ASV1557	Glacial	LacAmpRUFtf-a	0	Bacteria	Bacteroidota	Kapabacteria	Kapabacteriales	Kapabacteriaceae	OLB6
ASV1557	Glacial	LacAmpRUFtf-b	0	Bacteria	Bacteroidota	Kapabacteria	Kapabacteriales	Kapabacteriaceae	OLB6
ASV1557	Glacial	LacAmpRUFtf-c	0	Bacteria	Bacteroidota	Kapabacteria	Kapabacteriales	Kapabacteriaceae	OLB6
ASV1559	Baie de la Table	BdT0-2	0	Bacteria	Bacteroidota	Rhodothermia	Rhodothermales	Rubricoccaceae	Rubrivirga
ASV1559	Control	Ctr-tf-IIb	0	Bacteria	Bacteroidota	Rhodothermia	Rhodothermales	Rubricoccaceae	Rubrivirga
ASV1559	Non-glacial	LacADNRUFtfa	0	Bacteria	Bacteroidota	Rhodothermia	Rhodothermales	Rubricoccaceae	Rubrivirga
ASV1559	Non-glacial	LacADNRUFtfb	0	Bacteria	Bacteroidota	Rhodothermia	Rhodothermales	Rubricoccaceae	Rubrivirga

ASV1559	Non-glacial	LacADNRUFtfc	0	Bacteria	Bacteroidota	Rhodothermia	Rhodothermales	Rubricoccaceae	Rubrivirga
ASV1559	Glacial	LacAmpRUFtf-a	0	Bacteria	Bacteroidota	Rhodothermia	Rhodothermales	Rubricoccaceae	Rubrivirga
ASV1559	Glacial	LacAmpRUFtf-b	0	Bacteria	Bacteroidota	Rhodothermia	Rhodothermales	Rubricoccaceae	Rubrivirga
ASV1559	Glacial	LacAmpRUFtf-c	0	Bacteria	Bacteroidota	Rhodothermia	Rhodothermales	Rubricoccaceae	Rubrivirga
ASV1562	Baie de la Table	BdT0-2	0	Bacteria	Patescibacteria	Berkelbacteria	UBA12157	UBA12157	RBG-13-40-8
ASV1562	Control	Ctr-tf-IIb	0	Bacteria	Patescibacteria	Berkelbacteria	UBA12157	UBA12157	RBG-13-40-8
ASV1562	Non-glacial	LacADNRUFtfa	0	Bacteria	Patescibacteria	Berkelbacteria	UBA12157	UBA12157	RBG-13-40-8
ASV1562	Non-glacial	LacADNRUFtfb	0	Bacteria	Patescibacteria	Berkelbacteria	UBA12157	UBA12157	RBG-13-40-8
ASV1562	Non-glacial	LacADNRUFtfc	0	Bacteria	Patescibacteria	Berkelbacteria	UBA12157	UBA12157	RBG-13-40-8
ASV1562	Glacial	LacAmpRUFtf-a	0	Bacteria	Patescibacteria	Berkelbacteria	UBA12157	UBA12157	RBG-13-40-8
ASV1562	Glacial	LacAmpRUFtf-b	0	Bacteria	Patescibacteria	Berkelbacteria	UBA12157	UBA12157	RBG-13-40-8
ASV1562	Glacial	LacAmpRUFtf-c	0	Bacteria	Patescibacteria	Berkelbacteria	UBA12157	UBA12157	RBG-13-40-8
ASV1568	Baie de la Table	BdT0-2	0	Bacteria	Firmicutes	Bacilli	Mycoplasmatales	Hepatoplasmataceae	Hepatoplasma
ASV1568	Control	Ctr-tf-IIb	0	Bacteria	Firmicutes	Bacilli	Mycoplasmatales	Hepatoplasmataceae	Hepatoplasma
ASV1568	Non-glacial	LacADNRUFtfa	0	Bacteria	Firmicutes	Bacilli	Mycoplasmatales	Hepatoplasmataceae	Hepatoplasma
ASV1568	Non-glacial	LacADNRUFtfb	0	Bacteria	Firmicutes	Bacilli	Mycoplasmatales	Hepatoplasmataceae	Hepatoplasma
ASV1568	Non-glacial	LacADNRUFtfc	0	Bacteria	Firmicutes	Bacilli	Mycoplasmatales	Hepatoplasmataceae	Hepatoplasma
ASV1568	Glacial	LacAmpRUFtf-a	0	Bacteria	Firmicutes	Bacilli	Mycoplasmatales	Hepatoplasmataceae	Hepatoplasma
ASV1568	Glacial	LacAmpRUFtf-b	0	Bacteria	Firmicutes	Bacilli	Mycoplasmatales	Hepatoplasmataceae	Hepatoplasma
ASV1568	Glacial	LacAmpRUFtf-c	0	Bacteria	Firmicutes	Bacilli	Mycoplasmatales	Hepatoplasmataceae	Hepatoplasma
ASV1571	Glacial	LacAmpRUFtf-c	0,000242072	Bacteria	Actinobacteriota	Actinobacteria	Actinomycetales	Microbacteriaceae	Microbacterium
ASV1571	Baie de la Table	BdT0-2	0	Bacteria	Actinobacteriota	Actinobacteria	Actinomycetales	Microbacteriaceae	Microbacterium
ASV1571	Control	Ctr-tf-IIb	0	Bacteria	Actinobacteriota	Actinobacteria	Actinomycetales	Microbacteriaceae	Microbacterium
ASV1571	Non-glacial	LacADNRUFtfa	0	Bacteria	Actinobacteriota	Actinobacteria	Actinomycetales	Microbacteriaceae	Microbacterium
ASV1571	Non-glacial	LacADNRUFtfb	0	Bacteria	Actinobacteriota	Actinobacteria	Actinomycetales	Microbacteriaceae	Microbacterium
ASV1571	Non-glacial	LacADNRUFtfc	0	Bacteria	Actinobacteriota	Actinobacteria	Actinomycetales	Microbacteriaceae	Microbacterium
ASV1571	Glacial	LacAmpRUFtf-a	0	Bacteria	Actinobacteriota	Actinobacteria	Actinomycetales	Microbacteriaceae	Microbacterium
ASV1571	Glacial	LacAmpRUFtf-b	0	Bacteria	Actinobacteriota	Actinobacteria	Actinomycetales	Microbacteriaceae	Microbacterium
ASV1573	Baie de la Table	BdT0-2	0	Bacteria	Proteobacteria	Alphaproteobacteria	Sphingomonadales	Sphingomonadaceae	Erythrobacter_A
ASV1573	Control	Ctr-tf-IIb	0	Bacteria	Proteobacteria	Alphaproteobacteria	Sphingomonadales	Sphingomonadaceae	Erythrobacter_A
ASV1573	Non-glacial	LacADNRUFtfa	0	Bacteria	Proteobacteria	Alphaproteobacteria	Sphingomonadales	Sphingomonadaceae	Erythrobacter_A
ASV1573	Non-glacial	LacADNRUFtfb	0	Bacteria	Proteobacteria	Alphaproteobacteria	Sphingomonadales	Sphingomonadaceae	Erythrobacter_A
ASV1573	Non-glacial	LacADNRUFtfc	0	Bacteria	Proteobacteria	Alphaproteobacteria	Sphingomonadales	Sphingomonadaceae	Erythrobacter_A
ASV1573	Glacial	LacAmpRUFtf-a	0	Bacteria	Proteobacteria	Alphaproteobacteria	Sphingomonadales	Sphingomonadaceae	Erythrobacter_A

ASV1573	Glacial	LacAmpRUFtf-b	0	Bacteria	Proteobacteria	Alphaproteobacteria	Sphingomonadales	Sphingomonadaceae	Erythrobacter_A
ASV1573	Glacial	LacAmpRUFtf-c	0	Bacteria	Proteobacteria	Alphaproteobacteria	Sphingomonadales	Sphingomonadaceae	Erythrobacter_A
ASV158	Baie de la Table	BdT0-2	0	Bacteria	Verrucomicrobio	Verrucomicrobiae	Opitutales	Opitutaceae	Opitutus
ASV158	Control	Ctr-tf-IIb	0	Bacteria	Verrucomicrobio	Verrucomicrobiae	Opitutales	Opitutaceae	Opitutus
ASV158	Non-glacial	LacADNRUFtfa	0	Bacteria	Verrucomicrobio	Verrucomicrobiae	Opitutales	Opitutaceae	Opitutus
ASV158	Non-glacial	LacADNRUFtfb	0	Bacteria	Verrucomicrobio	Verrucomicrobiae	Opitutales	Opitutaceae	Opitutus
ASV158	Non-glacial	LacADNRUFtfc	0	Bacteria	Verrucomicrobio	Verrucomicrobiae	Opitutales	Opitutaceae	Opitutus
ASV158	Glacial	LacAmpRUFtf-a	0	Bacteria	Verrucomicrobio	Verrucomicrobiae	Opitutales	Opitutaceae	Opitutus
ASV158	Glacial	LacAmpRUFtf-b	0	Bacteria	Verrucomicrobio	Verrucomicrobiae	Opitutales	Opitutaceae	Opitutus
ASV158	Glacial	LacAmpRUFtf-c	0	Bacteria	Verrucomicrobio	Verrucomicrobiae	Opitutales	Opitutaceae	Opitutus
ASV1581	Non-glacial	LacADNRUFtfc	0,000121242	Bacteria	Bacteroidota	Bacteroidia	Sphingobacteriales	Sphingobacteriaceae	Mucilaginibacter
ASV1581	Non-glacial	LacADNRUFtfb	6,32E-05	Bacteria	Bacteroidota	Bacteroidia	Sphingobacteriales	Sphingobacteriaceae	Mucilaginibacter
ASV1581	Baie de la Table	BdT0-2	0	Bacteria	Bacteroidota	Bacteroidia	Sphingobacteriales	Sphingobacteriaceae	Mucilaginibacter
ASV1581	Control	Ctr-tf-IIb	0	Bacteria	Bacteroidota	Bacteroidia	Sphingobacteriales	Sphingobacteriaceae	Mucilaginibacter
ASV1581	Non-glacial	LacADNRUFtfa	0	Bacteria	Bacteroidota	Bacteroidia	Sphingobacteriales	Sphingobacteriaceae	Mucilaginibacter
ASV1581	Glacial	LacAmpRUFtf-a	0	Bacteria	Bacteroidota	Bacteroidia	Sphingobacteriales	Sphingobacteriaceae	Mucilaginibacter
ASV1581	Glacial	LacAmpRUFtf-b	0	Bacteria	Bacteroidota	Bacteroidia	Sphingobacteriales	Sphingobacteriaceae	Mucilaginibacter
ASV1581	Glacial	LacAmpRUFtf-c	0	Bacteria	Bacteroidota	Bacteroidia	Sphingobacteriales	Sphingobacteriaceae	Mucilaginibacter
ASV1592	Glacial	LacAmpRUFtf-b	0,000323939	Bacteria	Bacteroidota	Bacteroidia	Bacteroidales	Bacteroidaceae	Prevotella
ASV1592	Baie de la Table	BdT0-2	0	Bacteria	Bacteroidota	Bacteroidia	Bacteroidales	Bacteroidaceae	Prevotella
ASV1592	Control	Ctr-tf-IIb	0	Bacteria	Bacteroidota	Bacteroidia	Bacteroidales	Bacteroidaceae	Prevotella
ASV1592	Non-glacial	LacADNRUFtfa	0	Bacteria	Bacteroidota	Bacteroidia	Bacteroidales	Bacteroidaceae	Prevotella
ASV1592	Non-glacial	LacADNRUFtfb	0	Bacteria	Bacteroidota	Bacteroidia	Bacteroidales	Bacteroidaceae	Prevotella
ASV1592	Non-glacial	LacADNRUFtfc	0	Bacteria	Bacteroidota	Bacteroidia	Bacteroidales	Bacteroidaceae	Prevotella
ASV1592	Glacial	LacAmpRUFtf-a	0	Bacteria	Bacteroidota	Bacteroidia	Bacteroidales	Bacteroidaceae	Prevotella
ASV1592	Glacial	LacAmpRUFtf-c	0	Bacteria	Bacteroidota	Bacteroidia	Bacteroidales	Bacteroidaceae	Prevotella
ASV1593	Baie de la Table	BdT0-2	0	Bacteria	Nitrospirota	Nitrospiria	Nitrospirales	Nitrospiraceae	Nitrospira
ASV1593	Control	Ctr-tf-IIb	0	Bacteria	Nitrospirota	Nitrospiria	Nitrospirales	Nitrospiraceae	Nitrospira
ASV1593	Non-glacial	LacADNRUFtfa	0	Bacteria	Nitrospirota	Nitrospiria	Nitrospirales	Nitrospiraceae	Nitrospira
ASV1593	Non-glacial	LacADNRUFtfb	0	Bacteria	Nitrospirota	Nitrospiria	Nitrospirales	Nitrospiraceae	Nitrospira
ASV1593	Non-glacial	LacADNRUFtfc	0	Bacteria	Nitrospirota	Nitrospiria	Nitrospirales	Nitrospiraceae	Nitrospira
ASV1593	Glacial	LacAmpRUFtf-a	0	Bacteria	Nitrospirota	Nitrospiria	Nitrospirales	Nitrospiraceae	Nitrospira
ASV1593	Glacial	LacAmpRUFtf-b	0	Bacteria	Nitrospirota	Nitrospiria	Nitrospirales	Nitrospiraceae	Nitrospira
ASV1593	Glacial	LacAmpRUFtf-c	0	Bacteria	Nitrospirota	Nitrospiria	Nitrospirales	Nitrospiraceae	Nitrospira

ASV1602	Baie de la Table	BdT0-2	0	Bacteria	Proteobacteria	Alphaproteobacteria	Caulobacterales	Maricaulaceae	Maricaulis
ASV1602	Control	Ctr-tf-IIb	0	Bacteria	Proteobacteria	Alphaproteobacteria	Caulobacterales	Maricaulaceae	Maricaulis
ASV1602	Non-glacial	LacADNRUFtfa	0	Bacteria	Proteobacteria	Alphaproteobacteria	Caulobacterales	Maricaulaceae	Maricaulis
ASV1602	Non-glacial	LacADNRUFtfb	0	Bacteria	Proteobacteria	Alphaproteobacteria	Caulobacterales	Maricaulaceae	Maricaulis
ASV1602	Non-glacial	LacADNRUFtfc	0	Bacteria	Proteobacteria	Alphaproteobacteria	Caulobacterales	Maricaulaceae	Maricaulis
ASV1602	Glacial	LacAmpRUFtf-a	0	Bacteria	Proteobacteria	Alphaproteobacteria	Caulobacterales	Maricaulaceae	Maricaulis
ASV1602	Glacial	LacAmpRUFtf-b	0	Bacteria	Proteobacteria	Alphaproteobacteria	Caulobacterales	Maricaulaceae	Maricaulis
ASV1602	Glacial	LacAmpRUFtf-c	0	Bacteria	Proteobacteria	Alphaproteobacteria	Caulobacterales	Maricaulaceae	Maricaulis
ASV1622	Baie de la Table	BdT0-2	0	Bacteria	Proteobacteria	Gammaproteobacteria	Enterobacterales	Pasteurellaceae	Aggregatibacter
ASV1622	Control	Ctr-tf-IIb	0	Bacteria	Proteobacteria	Gammaproteobacteria	Enterobacterales	Pasteurellaceae	Aggregatibacter
ASV1622	Non-glacial	LacADNRUFtfa	0	Bacteria	Proteobacteria	Gammaproteobacteria	Enterobacterales	Pasteurellaceae	Aggregatibacter
ASV1622	Non-glacial	LacADNRUFtfb	0	Bacteria	Proteobacteria	Gammaproteobacteria	Enterobacterales	Pasteurellaceae	Aggregatibacter
ASV1622	Non-glacial	LacADNRUFtfc	0	Bacteria	Proteobacteria	Gammaproteobacteria	Enterobacterales	Pasteurellaceae	Aggregatibacter
ASV1622	Glacial	LacAmpRUFtf-a	0	Bacteria	Proteobacteria	Gammaproteobacteria	Enterobacterales	Pasteurellaceae	Aggregatibacter
ASV1622	Glacial	LacAmpRUFtf-b	0	Bacteria	Proteobacteria	Gammaproteobacteria	Enterobacterales	Pasteurellaceae	Aggregatibacter
ASV1622	Glacial	LacAmpRUFtf-c	0	Bacteria	Proteobacteria	Gammaproteobacteria	Enterobacterales	Pasteurellaceae	Aggregatibacter
ASV1628	Baie de la Table	BdT0-2	0	Bacteria	Bacteroidota	Bacteroidia	Chitinophagales	Saprospiraceae	UBA10441
ASV1628	Control	Ctr-tf-IIb	0	Bacteria	Bacteroidota	Bacteroidia	Chitinophagales	Saprospiraceae	UBA10441
ASV1628	Non-glacial	LacADNRUFtfa	0	Bacteria	Bacteroidota	Bacteroidia	Chitinophagales	Saprospiraceae	UBA10441
ASV1628	Non-glacial	LacADNRUFtfb	0	Bacteria	Bacteroidota	Bacteroidia	Chitinophagales	Saprospiraceae	UBA10441
ASV1628	Non-glacial	LacADNRUFtfc	0	Bacteria	Bacteroidota	Bacteroidia	Chitinophagales	Saprospiraceae	UBA10441
ASV1628	Glacial	LacAmpRUFtf-a	0	Bacteria	Bacteroidota	Bacteroidia	Chitinophagales	Saprospiraceae	UBA10441
ASV1628	Glacial	LacAmpRUFtf-b	0	Bacteria	Bacteroidota	Bacteroidia	Chitinophagales	Saprospiraceae	UBA10441
ASV1628	Glacial	LacAmpRUFtf-c	0	Bacteria	Bacteroidota	Bacteroidia	Chitinophagales	Saprospiraceae	UBA10441
ASV1629	Baie de la Table	BdT0-2	0	Bacteria	Planctomycetota	Planctomycetes	Planctomycetales	Planctomycetaceae	Planctopirus
ASV1629	Control	Ctr-tf-IIb	0	Bacteria	Planctomycetota	Planctomycetes	Planctomycetales	Planctomycetaceae	Planctopirus
ASV1629	Non-glacial	LacADNRUFtfa	0	Bacteria	Planctomycetota	Planctomycetes	Planctomycetales	Planctomycetaceae	Planctopirus
ASV1629	Non-glacial	LacADNRUFtfb	0	Bacteria	Planctomycetota	Planctomycetes	Planctomycetales	Planctomycetaceae	Planctopirus
ASV1629	Non-glacial	LacADNRUFtfc	0	Bacteria	Planctomycetota	Planctomycetes	Planctomycetales	Planctomycetaceae	Planctopirus
ASV1629	Glacial	LacAmpRUFtf-a	0	Bacteria	Planctomycetota	Planctomycetes	Planctomycetales	Planctomycetaceae	Planctopirus
ASV1629	Glacial	LacAmpRUFtf-b	0	Bacteria	Planctomycetota	Planctomycetes	Planctomycetales	Planctomycetaceae	Planctopirus
ASV1629	Glacial	LacAmpRUFtf-c	0	Bacteria	Planctomycetota	Planctomycetes	Planctomycetales	Planctomycetaceae	Planctopirus
ASV1637	Baie de la Table	BdT0-2	0	Bacteria	Proteobacteria	Zetaproteobacteria	Mariprofundales	Mariprofundaceae	Mariprofundus
ASV1637	Control	Ctr-tf-IIb	0	Bacteria	Proteobacteria	Zetaproteobacteria	Mariprofundales	Mariprofundaceae	Mariprofundus

ASV1637	Non-glacial	LacADNRUFtfa	0	Bacteria	Proteobacteria	Zetaproteobacteria	Mariprofundales	Mariprofundaceae	Mariprofundus
ASV1637	Non-glacial	LacADNRUFtfb	0	Bacteria	Proteobacteria	Zetaproteobacteria	Mariprofundales	Mariprofundaceae	Mariprofundus
ASV1637	Non-glacial	LacADNRUFtfc	0	Bacteria	Proteobacteria	Zetaproteobacteria	Mariprofundales	Mariprofundaceae	Mariprofundus
ASV1637	Glacial	LacAmpRUFtf-a	0	Bacteria	Proteobacteria	Zetaproteobacteria	Mariprofundales	Mariprofundaceae	Mariprofundus
ASV1637	Glacial	LacAmpRUFtf-b	0	Bacteria	Proteobacteria	Zetaproteobacteria	Mariprofundales	Mariprofundaceae	Mariprofundus
ASV1637	Glacial	LacAmpRUFtf-c	0	Bacteria	Proteobacteria	Zetaproteobacteria	Mariprofundales	Mariprofundaceae	Mariprofundus
ASV1638	Baie de la Table	BdT0-2	0	Bacteria	Firmicutes_C	Negativicutes	Veillonellales	Veillonellaceae	Veillonella
ASV1638	Control	Ctr-tf-IIb	0	Bacteria	Firmicutes_C	Negativicutes	Veillonellales	Veillonellaceae	Veillonella
ASV1638	Non-glacial	LacADNRUFtfa	0	Bacteria	Firmicutes_C	Negativicutes	Veillonellales	Veillonellaceae	Veillonella
ASV1638	Non-glacial	LacADNRUFtfb	0	Bacteria	Firmicutes_C	Negativicutes	Veillonellales	Veillonellaceae	Veillonella
ASV1638	Non-glacial	LacADNRUFtfc	0	Bacteria	Firmicutes_C	Negativicutes	Veillonellales	Veillonellaceae	Veillonella
ASV1638	Glacial	LacAmpRUFtf-a	0	Bacteria	Firmicutes_C	Negativicutes	Veillonellales	Veillonellaceae	Veillonella
ASV1638	Glacial	LacAmpRUFtf-b	0	Bacteria	Firmicutes_C	Negativicutes	Veillonellales	Veillonellaceae	Veillonella
ASV1638	Glacial	LacAmpRUFtf-c	0	Bacteria	Firmicutes_C	Negativicutes	Veillonellales	Veillonellaceae	Veillonella
ASV164	Glacial	LacAmpRUFtf-a	0,00124578	Bacteria	Bacteroidota	Bacteroidia	Flavobacteriales	Flavobacteriaceae	UBA7446
ASV164	Baie de la Table	BdT0-2	0,001147666	Bacteria	Bacteroidota	Bacteroidia	Flavobacteriales	Flavobacteriaceae	UBA7446
ASV164	Glacial	LacAmpRUFtf-b	0,000863838	Bacteria	Bacteroidota	Bacteroidia	Flavobacteriales	Flavobacteriaceae	UBA7446
ASV164	Non-glacial	LacADNRUFtfa	0,00060049	Bacteria	Bacteroidota	Bacteroidia	Flavobacteriales	Flavobacteriaceae	UBA7446
ASV164	Non-glacial	LacADNRUFtfb	0,000505796	Bacteria	Bacteroidota	Bacteroidia	Flavobacteriales	Flavobacteriaceae	UBA7446
ASV164	Control	Ctr-tf-IIb	0,000356586	Bacteria	Bacteroidota	Bacteroidia	Flavobacteriales	Flavobacteriaceae	UBA7446
ASV164	Non-glacial	LacADNRUFtfc	0	Bacteria	Bacteroidota	Bacteroidia	Flavobacteriales	Flavobacteriaceae	UBA7446
ASV164	Glacial	LacAmpRUFtf-c	0	Bacteria	Bacteroidota	Bacteroidia	Flavobacteriales	Flavobacteriaceae	UBA7446
ASV1645	Glacial	LacAmpRUFtf-a	0,000200932	Bacteria	Proteobacteria	Gammaproteobacteria	Enterobacterales	Alteromonadaceae	Thalassotalea_A
ASV1645	Baie de la Table	BdT0-2	0	Bacteria	Proteobacteria	Gammaproteobacteria	Enterobacterales	Alteromonadaceae	Thalassotalea_A
ASV1645	Control	Ctr-tf-IIb	0	Bacteria	Proteobacteria	Gammaproteobacteria	Enterobacterales	Alteromonadaceae	Thalassotalea_A
ASV1645	Non-glacial	LacADNRUFtfa	0	Bacteria	Proteobacteria	Gammaproteobacteria	Enterobacterales	Alteromonadaceae	Thalassotalea_A
ASV1645	Non-glacial	LacADNRUFtfb	0	Bacteria	Proteobacteria	Gammaproteobacteria	Enterobacterales	Alteromonadaceae	Thalassotalea_A
ASV1645	Non-glacial	LacADNRUFtfc	0	Bacteria	Proteobacteria	Gammaproteobacteria	Enterobacterales	Alteromonadaceae	Thalassotalea_A
ASV1645	Glacial	LacAmpRUFtf-b	0	Bacteria	Proteobacteria	Gammaproteobacteria	Enterobacterales	Alteromonadaceae	Thalassotalea_A
ASV1645	Glacial	LacAmpRUFtf-c	0	Bacteria	Proteobacteria	Gammaproteobacteria	Enterobacterales	Alteromonadaceae	Thalassotalea_A
ASV1647	Glacial	LacAmpRUFtf-b	0,000539898	Bacteria	Actinobacteriota	Coriobacteriia	Coriobacteriales	Atopobiaceae	Olsenella_B
ASV1647	Baie de la Table	BdT0-2	0	Bacteria	Actinobacteriota	Coriobacteriia	Coriobacteriales	Atopobiaceae	Olsenella_B
ASV1647	Control	Ctr-tf-IIb	0	Bacteria	Actinobacteriota	Coriobacteriia	Coriobacteriales	Atopobiaceae	Olsenella_B
ASV1647	Non-glacial	LacADNRUFtfa	0	Bacteria	Actinobacteriota	Coriobacteriia	Coriobacteriales	Atopobiaceae	Olsenella_B

ASV1647	Non-glacial	LacADNRUFtfb	0	Bacteria	Actinobacteriota	Coriobacteriia	Coriobacteriales	Atopobiaceae	Olsenella_B
ASV1647	Non-glacial	LacADNRUFtfc	0	Bacteria	Actinobacteriota	Coriobacteriia	Coriobacteriales	Atopobiaceae	Olsenella_B
ASV1647	Glacial	LacAmpRUFtf-a	0	Bacteria	Actinobacteriota	Coriobacteriia	Coriobacteriales	Atopobiaceae	Olsenella_B
ASV1647	Glacial	LacAmpRUFtf-c	0	Bacteria	Actinobacteriota	Coriobacteriia	Coriobacteriales	Atopobiaceae	Olsenella_B
ASV1650	Glacial	LacAmpRUFtf-c	0,000242072	Bacteria	Actinobacteriota	Actinobacteria	Streptomycetales	Streptomycetaceae	Streptomyces_B
ASV1650	Baie de la Table	BdT0-2	0	Bacteria	Actinobacteriota	Actinobacteria	Streptomycetales	Streptomycetaceae	Streptomyces_B
ASV1650	Control	Ctr-tf-IIb	0	Bacteria	Actinobacteriota	Actinobacteria	Streptomycetales	Streptomycetaceae	Streptomyces_B
ASV1650	Non-glacial	LacADNRUFtfa	0	Bacteria	Actinobacteriota	Actinobacteria	Streptomycetales	Streptomycetaceae	Streptomyces_B
ASV1650	Non-glacial	LacADNRUFtfb	0	Bacteria	Actinobacteriota	Actinobacteria	Streptomycetales	Streptomycetaceae	Streptomyces_B
ASV1650	Non-glacial	LacADNRUFtfc	0	Bacteria	Actinobacteriota	Actinobacteria	Streptomycetales	Streptomycetaceae	Streptomyces_B
ASV1650	Glacial	LacAmpRUFtf-a	0	Bacteria	Actinobacteriota	Actinobacteria	Streptomycetales	Streptomycetaceae	Streptomyces_B
ASV1650	Glacial	LacAmpRUFtf-b	0	Bacteria	Actinobacteriota	Actinobacteria	Streptomycetales	Streptomycetaceae	Streptomyces_B
ASV1651	Glacial	LacAmpRUFtf-c	0,000242072	Bacteria	Bacteroidota	Bacteroidia	Bacteroidales	Bacteroidaceae	F0040
ASV1651	Baie de la Table	BdT0-2	0	Bacteria	Bacteroidota	Bacteroidia	Bacteroidales	Bacteroidaceae	F0040
ASV1651	Control	Ctr-tf-IIb	0	Bacteria	Bacteroidota	Bacteroidia	Bacteroidales	Bacteroidaceae	F0040
ASV1651	Non-glacial	LacADNRUFtfa	0	Bacteria	Bacteroidota	Bacteroidia	Bacteroidales	Bacteroidaceae	F0040
ASV1651	Non-glacial	LacADNRUFtfb	0	Bacteria	Bacteroidota	Bacteroidia	Bacteroidales	Bacteroidaceae	F0040
ASV1651	Non-glacial	LacADNRUFtfc	0	Bacteria	Bacteroidota	Bacteroidia	Bacteroidales	Bacteroidaceae	F0040
ASV1651	Glacial	LacAmpRUFtf-a	0	Bacteria	Bacteroidota	Bacteroidia	Bacteroidales	Bacteroidaceae	F0040
ASV1651	Glacial	LacAmpRUFtf-b	0	Bacteria	Bacteroidota	Bacteroidia	Bacteroidales	Bacteroidaceae	F0040
ASV1662	Baie de la Table	BdT0-2	0	Bacteria	Proteobacteria	Alphaproteobacteria	Caulobacterales	Caulobacteraceae	PMMR1
ASV1662	Control	Ctr-tf-IIb	0	Bacteria	Proteobacteria	Alphaproteobacteria	Caulobacterales	Caulobacteraceae	PMMR1
ASV1662	Non-glacial	LacADNRUFtfa	0	Bacteria	Proteobacteria	Alphaproteobacteria	Caulobacterales	Caulobacteraceae	PMMR1
ASV1662	Non-glacial	LacADNRUFtfb	0	Bacteria	Proteobacteria	Alphaproteobacteria	Caulobacterales	Caulobacteraceae	PMMR1
ASV1662	Non-glacial	LacADNRUFtfc	0	Bacteria	Proteobacteria	Alphaproteobacteria	Caulobacterales	Caulobacteraceae	PMMR1
ASV1662	Glacial	LacAmpRUFtf-a	0	Bacteria	Proteobacteria	Alphaproteobacteria	Caulobacterales	Caulobacteraceae	PMMR1
ASV1662	Glacial	LacAmpRUFtf-b	0	Bacteria	Proteobacteria	Alphaproteobacteria	Caulobacterales	Caulobacteraceae	PMMR1
ASV1662	Glacial	LacAmpRUFtf-c	0	Bacteria	Proteobacteria	Alphaproteobacteria	Caulobacterales	Caulobacteraceae	PMMR1
ASV1664	Baie de la Table	BdT0-2	0	Bacteria	Desulfuromonad	Desulfuromonadia	Geobacterales	Geobacteraceae	Geobacter_A
ASV1664	Control	Ctr-tf-IIb	0	Bacteria	Desulfuromonad	Desulfuromonadia	Geobacterales	Geobacteraceae	Geobacter_A
ASV1664	Non-glacial	LacADNRUFtfa	0	Bacteria	Desulfuromonad	Desulfuromonadia	Geobacterales	Geobacteraceae	Geobacter_A
ASV1664	Non-glacial	LacADNRUFtfb	0	Bacteria	Desulfuromonad	Desulfuromonadia	Geobacterales	Geobacteraceae	Geobacter_A
ASV1664	Non-glacial	LacADNRUFtfc	0	Bacteria	Desulfuromonad	Desulfuromonadia	Geobacterales	Geobacteraceae	Geobacter_A
ASV1664	Glacial	LacAmpRUFtf-a	0	Bacteria	Desulfuromonad	Desulfuromonadia	Geobacterales	Geobacteraceae	Geobacter_A

ASV1664	Glacial	LacAmpRUFtf-b	0	Bacteria	Desulfuromonad	Desulfuromonadia	Geobacterales	Geobacteraceae	Geobacter_A
ASV1664	Glacial	LacAmpRUFtf-c	0	Bacteria	Desulfuromonad	Desulfuromonadia	Geobacterales	Geobacteraceae	Geobacter_A
ASV1665	Baie de la Table	BdT0-2	0	Bacteria	Proteobacteria	Gammaproteobacteria	Methylococcales	Methylomonadaceae	KS41
ASV1665	Control	Ctr-tf-IIb	0	Bacteria	Proteobacteria	Gammaproteobacteria	Methylococcales	Methylomonadaceae	KS41
ASV1665	Non-glacial	LacADNRUFtfa	0	Bacteria	Proteobacteria	Gammaproteobacteria	Methylococcales	Methylomonadaceae	KS41
ASV1665	Non-glacial	LacADNRUFtfb	0	Bacteria	Proteobacteria	Gammaproteobacteria	Methylococcales	Methylomonadaceae	KS41
ASV1665	Non-glacial	LacADNRUFtfc	0	Bacteria	Proteobacteria	Gammaproteobacteria	Methylococcales	Methylomonadaceae	KS41
ASV1665	Glacial	LacAmpRUFtf-a	0	Bacteria	Proteobacteria	Gammaproteobacteria	Methylococcales	Methylomonadaceae	KS41
ASV1665	Glacial	LacAmpRUFtf-b	0	Bacteria	Proteobacteria	Gammaproteobacteria	Methylococcales	Methylomonadaceae	KS41
ASV1665	Glacial	LacAmpRUFtf-c	0	Bacteria	Proteobacteria	Gammaproteobacteria	Methylococcales	Methylomonadaceae	KS41
ASV1666	Non-glacial	LacADNRUFtfc	4,85E-05	Bacteria	Bacteroidota	Bacteroidia	Chitinophagales	Chitinophagaceae	Niabella
ASV1666	Baie de la Table	BdT0-2	0	Bacteria	Bacteroidota	Bacteroidia	Chitinophagales	Chitinophagaceae	Niabella
ASV1666	Control	Ctr-tf-IIb	0	Bacteria	Bacteroidota	Bacteroidia	Chitinophagales	Chitinophagaceae	Niabella
ASV1666	Non-glacial	LacADNRUFtfa	0	Bacteria	Bacteroidota	Bacteroidia	Chitinophagales	Chitinophagaceae	Niabella
ASV1666	Non-glacial	LacADNRUFtfb	0	Bacteria	Bacteroidota	Bacteroidia	Chitinophagales	Chitinophagaceae	Niabella
ASV1666	Glacial	LacAmpRUFtf-a	0	Bacteria	Bacteroidota	Bacteroidia	Chitinophagales	Chitinophagaceae	Niabella
ASV1666	Glacial	LacAmpRUFtf-b	0	Bacteria	Bacteroidota	Bacteroidia	Chitinophagales	Chitinophagaceae	Niabella
ASV1666	Glacial	LacAmpRUFtf-c	0	Bacteria	Bacteroidota	Bacteroidia	Chitinophagales	Chitinophagaceae	Niabella
ASV1668	Baie de la Table	BdT0-2	0	Bacteria	Proteobacteria	Gammaproteobacteria	Pseudomonadales	Halieaceae	Congregibacter
ASV1668	Control	Ctr-tf-IIb	0	Bacteria	Proteobacteria	Gammaproteobacteria	Pseudomonadales	Halieaceae	Congregibacter
ASV1668	Non-glacial	LacADNRUFtfa	0	Bacteria	Proteobacteria	Gammaproteobacteria	Pseudomonadales	Halieaceae	Congregibacter
ASV1668	Non-glacial	LacADNRUFtfb	0	Bacteria	Proteobacteria	Gammaproteobacteria	Pseudomonadales	Halieaceae	Congregibacter
ASV1668	Non-glacial	LacADNRUFtfc	0	Bacteria	Proteobacteria	Gammaproteobacteria	Pseudomonadales	Halieaceae	Congregibacter
ASV1668	Glacial	LacAmpRUFtf-a	0	Bacteria	Proteobacteria	Gammaproteobacteria	Pseudomonadales	Halieaceae	Congregibacter
ASV1668	Glacial	LacAmpRUFtf-b	0	Bacteria	Proteobacteria	Gammaproteobacteria	Pseudomonadales	Halieaceae	Congregibacter
ASV1668	Glacial	LacAmpRUFtf-c	0	Bacteria	Proteobacteria	Gammaproteobacteria	Pseudomonadales	Halieaceae	Congregibacter
ASV1674	Control	Ctr-tf-IIb	1,60E-05	Bacteria	Campylobactero	Campylobacteria	Campylobacterales	Sulfurovaceae	Sulfurovum
ASV1674	Baie de la Table	BdT0-2	0	Bacteria	Campylobactero	Campylobacteria	Campylobacterales	Sulfurovaceae	Sulfurovum
ASV1674	Non-glacial	LacADNRUFtfa	0	Bacteria	Campylobactero	Campylobacteria	Campylobacterales	Sulfurovaceae	Sulfurovum
ASV1674	Non-glacial	LacADNRUFtfb	0	Bacteria	Campylobactero	Campylobacteria	Campylobacterales	Sulfurovaceae	Sulfurovum
ASV1674	Non-glacial	LacADNRUFtfc	0	Bacteria	Campylobactero	Campylobacteria	Campylobacterales	Sulfurovaceae	Sulfurovum
ASV1674	Glacial	LacAmpRUFtf-a	0	Bacteria	Campylobactero	Campylobacteria	Campylobacterales	Sulfurovaceae	Sulfurovum
ASV1674	Glacial	LacAmpRUFtf-b	0	Bacteria	Campylobactero	Campylobacteria	Campylobacterales	Sulfurovaceae	Sulfurovum
ASV1674	Glacial	LacAmpRUFtf-c	0	Bacteria	Campylobactero	Campylobacteria	Campylobacterales	Sulfurovaceae	Sulfurovum

ASV168(	Baie de la Table	BdT0-2	0	Bacteria	Patescibacteria	Paceibacteria	UBA9983	UBA5272	UBA5272
ASV168(	Control	Ctr-tf-IIb	0	Bacteria	Patescibacteria	Paceibacteria	UBA9983	UBA5272	UBA5272
ASV168(	Non-glacial	LacADNRUFtfa	0	Bacteria	Patescibacteria	Paceibacteria	UBA9983	UBA5272	UBA5272
ASV168(	Non-glacial	LacADNRUFtfb	0	Bacteria	Patescibacteria	Paceibacteria	UBA9983	UBA5272	UBA5272
ASV168(	Non-glacial	LacADNRUFtfc	0	Bacteria	Patescibacteria	Paceibacteria	UBA9983	UBA5272	UBA5272
ASV168(	Glacial	LacAmpRUFtf-a	0	Bacteria	Patescibacteria	Paceibacteria	UBA9983	UBA5272	UBA5272
ASV168(	Glacial	LacAmpRUFtf-b	0	Bacteria	Patescibacteria	Paceibacteria	UBA9983	UBA5272	UBA5272
ASV168(	Glacial	LacAmpRUFtf-c	0	Bacteria	Patescibacteria	Paceibacteria	UBA9983	UBA5272	UBA5272
ASV168:	Baie de la Table	BdT0-2	0	Bacteria	Acidobacteriota	Holophagae	Holophagales	Holophagaceae	Holophaga
ASV168:	Control	Ctr-tf-IIb	0	Bacteria	Acidobacteriota	Holophagae	Holophagales	Holophagaceae	Holophaga
ASV168:	Non-glacial	LacADNRUFtfa	0	Bacteria	Acidobacteriota	Holophagae	Holophagales	Holophagaceae	Holophaga
ASV168:	Non-glacial	LacADNRUFtfb	0	Bacteria	Acidobacteriota	Holophagae	Holophagales	Holophagaceae	Holophaga
ASV168:	Non-glacial	LacADNRUFtfc	0	Bacteria	Acidobacteriota	Holophagae	Holophagales	Holophagaceae	Holophaga
ASV168:	Glacial	LacAmpRUFtf-a	0	Bacteria	Acidobacteriota	Holophagae	Holophagales	Holophagaceae	Holophaga
ASV168:	Glacial	LacAmpRUFtf-b	0	Bacteria	Acidobacteriota	Holophagae	Holophagales	Holophagaceae	Holophaga
ASV168:	Glacial	LacAmpRUFtf-c	0	Bacteria	Acidobacteriota	Holophagae	Holophagales	Holophagaceae	Holophaga
ASV168!	Baie de la Table	BdT0-2	0	Bacteria	Armatimonadota	Fimbriimonadia	Fimbriimonadales	Fimbriimonadaceae	Fimbriimonas
ASV168!	Control	Ctr-tf-IIb	0	Bacteria	Armatimonadota	Fimbriimonadia	Fimbriimonadales	Fimbriimonadaceae	Fimbriimonas
ASV168!	Non-glacial	LacADNRUFtfa	0	Bacteria	Armatimonadota	Fimbriimonadia	Fimbriimonadales	Fimbriimonadaceae	Fimbriimonas
ASV168!	Non-glacial	LacADNRUFtfb	0	Bacteria	Armatimonadota	Fimbriimonadia	Fimbriimonadales	Fimbriimonadaceae	Fimbriimonas
ASV168!	Non-glacial	LacADNRUFtfc	0	Bacteria	Armatimonadota	Fimbriimonadia	Fimbriimonadales	Fimbriimonadaceae	Fimbriimonas
ASV168!	Glacial	LacAmpRUFtf-a	0	Bacteria	Armatimonadota	Fimbriimonadia	Fimbriimonadales	Fimbriimonadaceae	Fimbriimonas
ASV168!	Glacial	LacAmpRUFtf-b	0	Bacteria	Armatimonadota	Fimbriimonadia	Fimbriimonadales	Fimbriimonadaceae	Fimbriimonas
ASV168!	Glacial	LacAmpRUFtf-c	0	Bacteria	Armatimonadota	Fimbriimonadia	Fimbriimonadales	Fimbriimonadaceae	Fimbriimonas
ASV169:	Baie de la Table	BdT0-2	0	Bacteria	Armatimonadota	Chthonomonadetes	Chthonomonadales	Chthonomonadaceae	Chthonomonas
ASV169:	Control	Ctr-tf-IIb	0	Bacteria	Armatimonadota	Chthonomonadetes	Chthonomonadales	Chthonomonadaceae	Chthonomonas
ASV169:	Non-glacial	LacADNRUFtfa	0	Bacteria	Armatimonadota	Chthonomonadetes	Chthonomonadales	Chthonomonadaceae	Chthonomonas
ASV169:	Non-glacial	LacADNRUFtfb	0	Bacteria	Armatimonadota	Chthonomonadetes	Chthonomonadales	Chthonomonadaceae	Chthonomonas
ASV169:	Non-glacial	LacADNRUFtfc	0	Bacteria	Armatimonadota	Chthonomonadetes	Chthonomonadales	Chthonomonadaceae	Chthonomonas
ASV169:	Glacial	LacAmpRUFtf-a	0	Bacteria	Armatimonadota	Chthonomonadetes	Chthonomonadales	Chthonomonadaceae	Chthonomonas
ASV169:	Glacial	LacAmpRUFtf-b	0	Bacteria	Armatimonadota	Chthonomonadetes	Chthonomonadales	Chthonomonadaceae	Chthonomonas
ASV169:	Glacial	LacAmpRUFtf-c	0	Bacteria	Armatimonadota	Chthonomonadetes	Chthonomonadales	Chthonomonadaceae	Chthonomonas
ASV169!	Baie de la Table	BdT0-2	0	Archaea	Nanoarchaeota	Woesearchaeia	Pacearchaeales	GW2011-AR1	GW2011-AR1
ASV169!	Control	Ctr-tf-IIb	0	Archaea	Nanoarchaeota	Woesearchaeia	Pacearchaeales	GW2011-AR1	GW2011-AR1

ASV1695	Non-glacial	LacADNRUFtfa	0	Archaea	Nanoarchaeota	Woesearchaeia	Pacearchaeales	GW2011-AR1	GW2011-AR1
ASV1695	Non-glacial	LacADNRUFtfb	0	Archaea	Nanoarchaeota	Woesearchaeia	Pacearchaeales	GW2011-AR1	GW2011-AR1
ASV1695	Non-glacial	LacADNRUFtfc	0	Archaea	Nanoarchaeota	Woesearchaeia	Pacearchaeales	GW2011-AR1	GW2011-AR1
ASV1695	Glacial	LacAmpRUFtf-a	0	Archaea	Nanoarchaeota	Woesearchaeia	Pacearchaeales	GW2011-AR1	GW2011-AR1
ASV1695	Glacial	LacAmpRUFtf-b	0	Archaea	Nanoarchaeota	Woesearchaeia	Pacearchaeales	GW2011-AR1	GW2011-AR1
ASV1695	Glacial	LacAmpRUFtf-c	0	Archaea	Nanoarchaeota	Woesearchaeia	Pacearchaeales	GW2011-AR1	GW2011-AR1
ASV17	Baie de la Table	BdT0-2	0,028746311	Bacteria	Bacteroidota	Bacteroidia	Flavobacteriales	Cryomorphaceae	UBA10364
ASV17	Non-glacial	LacADNRUFtfa	0,003325789	Bacteria	Bacteroidota	Bacteroidia	Flavobacteriales	Cryomorphaceae	UBA10364
ASV17	Non-glacial	LacADNRUFtfb	0,002592202	Bacteria	Bacteroidota	Bacteroidia	Flavobacteriales	Cryomorphaceae	UBA10364
ASV17	Glacial	LacAmpRUFtf-b	0,001943635	Bacteria	Bacteroidota	Bacteroidia	Flavobacteriales	Cryomorphaceae	UBA10364
ASV17	Glacial	LacAmpRUFtf-a	0,001728018	Bacteria	Bacteroidota	Bacteroidia	Flavobacteriales	Cryomorphaceae	UBA10364
ASV17	Control	Ctr-tf-IIb	0,000969594	Bacteria	Bacteroidota	Bacteroidia	Flavobacteriales	Cryomorphaceae	UBA10364
ASV17	Glacial	LacAmpRUFtf-c	0,000774631	Bacteria	Bacteroidota	Bacteroidia	Flavobacteriales	Cryomorphaceae	UBA10364
ASV17	Non-glacial	LacADNRUFtfc	0,00014549	Bacteria	Bacteroidota	Bacteroidia	Flavobacteriales	Cryomorphaceae	UBA10364
ASV170	Glacial	LacAmpRUFtf-a	0,001486899	Bacteria	Campylobactero	Campylobacteria	Campylobacterales	Arcobacteraceae	Arcobacter
ASV170	Control	Ctr-tf-IIb	0,000769265	Bacteria	Campylobactero	Campylobacteria	Campylobacterales	Arcobacteraceae	Arcobacter
ASV170	Non-glacial	LacADNRUFtfc	0,000242483	Bacteria	Campylobactero	Campylobacteria	Campylobacterales	Arcobacteraceae	Arcobacter
ASV170	Baie de la Table	BdT0-2	0	Bacteria	Campylobactero	Campylobacteria	Campylobacterales	Arcobacteraceae	Arcobacter
ASV170	Non-glacial	LacADNRUFtfa	0	Bacteria	Campylobactero	Campylobacteria	Campylobacterales	Arcobacteraceae	Arcobacter
ASV170	Non-glacial	LacADNRUFtfb	0	Bacteria	Campylobactero	Campylobacteria	Campylobacterales	Arcobacteraceae	Arcobacter
ASV170	Glacial	LacAmpRUFtf-b	0	Bacteria	Campylobactero	Campylobacteria	Campylobacterales	Arcobacteraceae	Arcobacter
ASV170	Glacial	LacAmpRUFtf-c	0	Bacteria	Campylobactero	Campylobacteria	Campylobacterales	Arcobacteraceae	Arcobacter
ASV1706	Baie de la Table	BdT0-2	0	Bacteria	Proteobacteria	Alphaproteobacteria	Rhodobacterales	Rhodobacteraceae	Rubellimicrobium
ASV1706	Control	Ctr-tf-IIb	0	Bacteria	Proteobacteria	Alphaproteobacteria	Rhodobacterales	Rhodobacteraceae	Rubellimicrobium
ASV1706	Non-glacial	LacADNRUFtfa	0	Bacteria	Proteobacteria	Alphaproteobacteria	Rhodobacterales	Rhodobacteraceae	Rubellimicrobium
ASV1706	Non-glacial	LacADNRUFtfb	0	Bacteria	Proteobacteria	Alphaproteobacteria	Rhodobacterales	Rhodobacteraceae	Rubellimicrobium
ASV1706	Non-glacial	LacADNRUFtfc	0	Bacteria	Proteobacteria	Alphaproteobacteria	Rhodobacterales	Rhodobacteraceae	Rubellimicrobium
ASV1706	Glacial	LacAmpRUFtf-a	0	Bacteria	Proteobacteria	Alphaproteobacteria	Rhodobacterales	Rhodobacteraceae	Rubellimicrobium
ASV1706	Glacial	LacAmpRUFtf-b	0	Bacteria	Proteobacteria	Alphaproteobacteria	Rhodobacterales	Rhodobacteraceae	Rubellimicrobium
ASV1706	Glacial	LacAmpRUFtf-c	0	Bacteria	Proteobacteria	Alphaproteobacteria	Rhodobacterales	Rhodobacteraceae	Rubellimicrobium
ASV1709	Non-glacial	LacADNRUFtfa	9,24E-05	Bacteria	Dependentiae	Babeliae	Babeliales	UBA12409	2-12-FULL-36-22
ASV1709	Baie de la Table	BdT0-2	0	Bacteria	Dependentiae	Babeliae	Babeliales	UBA12409	2-12-FULL-36-22
ASV1709	Control	Ctr-tf-IIb	0	Bacteria	Dependentiae	Babeliae	Babeliales	UBA12409	2-12-FULL-36-22
ASV1709	Non-glacial	LacADNRUFtfb	0	Bacteria	Dependentiae	Babeliae	Babeliales	UBA12409	2-12-FULL-36-22

ASV1709	Non-glacial	LacADNRUFtfc	0	Bacteria	Dependentiae	Babeliae	Babeliales	UBA12409	2-12-FULL-36-22
ASV1709	Glacial	LacAmpRUFtf-a	0	Bacteria	Dependentiae	Babeliae	Babeliales	UBA12409	2-12-FULL-36-22
ASV1709	Glacial	LacAmpRUFtf-b	0	Bacteria	Dependentiae	Babeliae	Babeliales	UBA12409	2-12-FULL-36-22
ASV1709	Glacial	LacAmpRUFtf-c	0	Bacteria	Dependentiae	Babeliae	Babeliales	UBA12409	2-12-FULL-36-22
ASV1712	Baie de la Table	BdT0-2	0	Bacteria	Bdellovibrionota	Bdellovibrionia	Bdellovibrionales	Bdellovibrionaceae	Bdellovibrio
ASV1712	Control	Ctr-tf-IIb	0	Bacteria	Bdellovibrionota	Bdellovibrionia	Bdellovibrionales	Bdellovibrionaceae	Bdellovibrio
ASV1712	Non-glacial	LacADNRUFtfa	0	Bacteria	Bdellovibrionota	Bdellovibrionia	Bdellovibrionales	Bdellovibrionaceae	Bdellovibrio
ASV1712	Non-glacial	LacADNRUFtfb	0	Bacteria	Bdellovibrionota	Bdellovibrionia	Bdellovibrionales	Bdellovibrionaceae	Bdellovibrio
ASV1712	Non-glacial	LacADNRUFtfc	0	Bacteria	Bdellovibrionota	Bdellovibrionia	Bdellovibrionales	Bdellovibrionaceae	Bdellovibrio
ASV1712	Glacial	LacAmpRUFtf-a	0	Bacteria	Bdellovibrionota	Bdellovibrionia	Bdellovibrionales	Bdellovibrionaceae	Bdellovibrio
ASV1712	Glacial	LacAmpRUFtf-b	0	Bacteria	Bdellovibrionota	Bdellovibrionia	Bdellovibrionales	Bdellovibrionaceae	Bdellovibrio
ASV1712	Glacial	LacAmpRUFtf-c	0	Bacteria	Bdellovibrionota	Bdellovibrionia	Bdellovibrionales	Bdellovibrionaceae	Bdellovibrio
ASV172	Baie de la Table	BdT0-2	0	Bacteria	Planctomycetota	Planctomycetes	Pirellulales	Pirellulaceae	Pirellula
ASV172	Control	Ctr-tf-IIb	0	Bacteria	Planctomycetota	Planctomycetes	Pirellulales	Pirellulaceae	Pirellula
ASV172	Non-glacial	LacADNRUFtfa	0	Bacteria	Planctomycetota	Planctomycetes	Pirellulales	Pirellulaceae	Pirellula
ASV172	Non-glacial	LacADNRUFtfb	0	Bacteria	Planctomycetota	Planctomycetes	Pirellulales	Pirellulaceae	Pirellula
ASV172	Non-glacial	LacADNRUFtfc	0	Bacteria	Planctomycetota	Planctomycetes	Pirellulales	Pirellulaceae	Pirellula
ASV172	Glacial	LacAmpRUFtf-a	0	Bacteria	Planctomycetota	Planctomycetes	Pirellulales	Pirellulaceae	Pirellula
ASV172	Glacial	LacAmpRUFtf-b	0	Bacteria	Planctomycetota	Planctomycetes	Pirellulales	Pirellulaceae	Pirellula
ASV172	Glacial	LacAmpRUFtf-c	0	Bacteria	Planctomycetota	Planctomycetes	Pirellulales	Pirellulaceae	Pirellula
ASV1723	Non-glacial	LacADNRUFtfb	8,43E-05	Bacteria	Proteobacteria	Alphaproteobacteria	Rhizobiales	Rhizobiaceae	Hoeflea
ASV1723	Baie de la Table	BdT0-2	0	Bacteria	Proteobacteria	Alphaproteobacteria	Rhizobiales	Rhizobiaceae	Hoeflea
ASV1723	Control	Ctr-tf-IIb	0	Bacteria	Proteobacteria	Alphaproteobacteria	Rhizobiales	Rhizobiaceae	Hoeflea
ASV1723	Non-glacial	LacADNRUFtfa	0	Bacteria	Proteobacteria	Alphaproteobacteria	Rhizobiales	Rhizobiaceae	Hoeflea
ASV1723	Non-glacial	LacADNRUFtfc	0	Bacteria	Proteobacteria	Alphaproteobacteria	Rhizobiales	Rhizobiaceae	Hoeflea
ASV1723	Glacial	LacAmpRUFtf-a	0	Bacteria	Proteobacteria	Alphaproteobacteria	Rhizobiales	Rhizobiaceae	Hoeflea
ASV1723	Glacial	LacAmpRUFtf-b	0	Bacteria	Proteobacteria	Alphaproteobacteria	Rhizobiales	Rhizobiaceae	Hoeflea
ASV1723	Glacial	LacAmpRUFtf-c	0	Bacteria	Proteobacteria	Alphaproteobacteria	Rhizobiales	Rhizobiaceae	Hoeflea
ASV1729	Non-glacial	LacADNRUFtfc	9,70E-05	Bacteria	Firmicutes	Bacilli	Exiguobacterales	Exiguobacteraceae	Exiguobacterium
ASV1729	Baie de la Table	BdT0-2	0	Bacteria	Firmicutes	Bacilli	Exiguobacterales	Exiguobacteraceae	Exiguobacterium
ASV1729	Control	Ctr-tf-IIb	0	Bacteria	Firmicutes	Bacilli	Exiguobacterales	Exiguobacteraceae	Exiguobacterium
ASV1729	Non-glacial	LacADNRUFtfa	0	Bacteria	Firmicutes	Bacilli	Exiguobacterales	Exiguobacteraceae	Exiguobacterium
ASV1729	Non-glacial	LacADNRUFtfb	0	Bacteria	Firmicutes	Bacilli	Exiguobacterales	Exiguobacteraceae	Exiguobacterium
ASV1729	Glacial	LacAmpRUFtf-a	0	Bacteria	Firmicutes	Bacilli	Exiguobacterales	Exiguobacteraceae	Exiguobacterium

ASV1729	Glacial	LacAmpRUFtf-b	0	Bacteria	Firmicutes	Bacilli	Exiguobacterales	Exiguobacteraceae	Exiguobacterium
ASV1729	Glacial	LacAmpRUFtf-c	0	Bacteria	Firmicutes	Bacilli	Exiguobacterales	Exiguobacteraceae	Exiguobacterium
ASV1731	Baie de la Table	BdT0-2	0	Bacteria	Proteobacteria	Alphaproteobacteria	Rhodospirillales	Casp-alpha2	UBA1479
ASV1731	Control	Ctr-tf-IIb	0	Bacteria	Proteobacteria	Alphaproteobacteria	Rhodospirillales	Casp-alpha2	UBA1479
ASV1731	Non-glacial	LacADNRUFtfa	0	Bacteria	Proteobacteria	Alphaproteobacteria	Rhodospirillales	Casp-alpha2	UBA1479
ASV1731	Non-glacial	LacADNRUFtfb	0	Bacteria	Proteobacteria	Alphaproteobacteria	Rhodospirillales	Casp-alpha2	UBA1479
ASV1731	Non-glacial	LacADNRUFtfc	0	Bacteria	Proteobacteria	Alphaproteobacteria	Rhodospirillales	Casp-alpha2	UBA1479
ASV1731	Glacial	LacAmpRUFtf-a	0	Bacteria	Proteobacteria	Alphaproteobacteria	Rhodospirillales	Casp-alpha2	UBA1479
ASV1731	Glacial	LacAmpRUFtf-b	0	Bacteria	Proteobacteria	Alphaproteobacteria	Rhodospirillales	Casp-alpha2	UBA1479
ASV1731	Glacial	LacAmpRUFtf-c	0	Bacteria	Proteobacteria	Alphaproteobacteria	Rhodospirillales	Casp-alpha2	UBA1479
ASV1735	Baie de la Table	BdT0-2	0	Bacteria	Firmicutes_A	Clostridia	Peptostreptococcales	Anaerovoracaceae	Eubacterium_M
ASV1735	Control	Ctr-tf-IIb	0	Bacteria	Firmicutes_A	Clostridia	Peptostreptococcales	Anaerovoracaceae	Eubacterium_M
ASV1735	Non-glacial	LacADNRUFtfa	0	Bacteria	Firmicutes_A	Clostridia	Peptostreptococcales	Anaerovoracaceae	Eubacterium_M
ASV1735	Non-glacial	LacADNRUFtfb	0	Bacteria	Firmicutes_A	Clostridia	Peptostreptococcales	Anaerovoracaceae	Eubacterium_M
ASV1735	Non-glacial	LacADNRUFtfc	0	Bacteria	Firmicutes_A	Clostridia	Peptostreptococcales	Anaerovoracaceae	Eubacterium_M
ASV1735	Glacial	LacAmpRUFtf-a	0	Bacteria	Firmicutes_A	Clostridia	Peptostreptococcales	Anaerovoracaceae	Eubacterium_M
ASV1735	Glacial	LacAmpRUFtf-b	0	Bacteria	Firmicutes_A	Clostridia	Peptostreptococcales	Anaerovoracaceae	Eubacterium_M
ASV1735	Glacial	LacAmpRUFtf-c	0	Bacteria	Firmicutes_A	Clostridia	Peptostreptococcales	Anaerovoracaceae	Eubacterium_M
ASV1736	Baie de la Table	BdT0-2	0	Bacteria	Planctomycetota	Planctomycetes	Planctomycetales	Planctomycetaceae	Gimesia
ASV1736	Control	Ctr-tf-IIb	0	Bacteria	Planctomycetota	Planctomycetes	Planctomycetales	Planctomycetaceae	Gimesia
ASV1736	Non-glacial	LacADNRUFtfa	0	Bacteria	Planctomycetota	Planctomycetes	Planctomycetales	Planctomycetaceae	Gimesia
ASV1736	Non-glacial	LacADNRUFtfb	0	Bacteria	Planctomycetota	Planctomycetes	Planctomycetales	Planctomycetaceae	Gimesia
ASV1736	Non-glacial	LacADNRUFtfc	0	Bacteria	Planctomycetota	Planctomycetes	Planctomycetales	Planctomycetaceae	Gimesia
ASV1736	Glacial	LacAmpRUFtf-a	0	Bacteria	Planctomycetota	Planctomycetes	Planctomycetales	Planctomycetaceae	Gimesia
ASV1736	Glacial	LacAmpRUFtf-b	0	Bacteria	Planctomycetota	Planctomycetes	Planctomycetales	Planctomycetaceae	Gimesia
ASV1736	Glacial	LacAmpRUFtf-c	0	Bacteria	Planctomycetota	Planctomycetes	Planctomycetales	Planctomycetaceae	Gimesia
ASV1738	Baie de la Table	BdT0-2	0	Bacteria	Actinobacteriota	Coriobacteriia	Coriobacteriales	Atopobiaceae	Atopobium_B
ASV1738	Control	Ctr-tf-IIb	0	Bacteria	Actinobacteriota	Coriobacteriia	Coriobacteriales	Atopobiaceae	Atopobium_B
ASV1738	Non-glacial	LacADNRUFtfa	0	Bacteria	Actinobacteriota	Coriobacteriia	Coriobacteriales	Atopobiaceae	Atopobium_B
ASV1738	Non-glacial	LacADNRUFtfb	0	Bacteria	Actinobacteriota	Coriobacteriia	Coriobacteriales	Atopobiaceae	Atopobium_B
ASV1738	Non-glacial	LacADNRUFtfc	0	Bacteria	Actinobacteriota	Coriobacteriia	Coriobacteriales	Atopobiaceae	Atopobium_B
ASV1738	Glacial	LacAmpRUFtf-a	0	Bacteria	Actinobacteriota	Coriobacteriia	Coriobacteriales	Atopobiaceae	Atopobium_B
ASV1738	Glacial	LacAmpRUFtf-b	0	Bacteria	Actinobacteriota	Coriobacteriia	Coriobacteriales	Atopobiaceae	Atopobium_B
ASV1738	Glacial	LacAmpRUFtf-c	0	Bacteria	Actinobacteriota	Coriobacteriia	Coriobacteriales	Atopobiaceae	Atopobium_B

ASV174	Non-glacial	LacADNRUFtfa	0,012379325	Bacteria	Proteobacteria	Gammaproteobacteria	Betaproteobacteriales	Burkholderiaceae	Rugamonas
ASV174	Non-glacial	LacADNRUFtfb	0,011190727	Bacteria	Proteobacteria	Gammaproteobacteria	Betaproteobacteriales	Burkholderiaceae	Rugamonas
ASV174	Non-glacial	LacADNRUFtfc	0,003370514	Bacteria	Proteobacteria	Gammaproteobacteria	Betaproteobacteriales	Burkholderiaceae	Rugamonas
ASV174	Baie de la Table	BdT0-2	0	Bacteria	Proteobacteria	Gammaproteobacteria	Betaproteobacteriales	Burkholderiaceae	Rugamonas
ASV174	Control	Ctr-tf-IIb	0	Bacteria	Proteobacteria	Gammaproteobacteria	Betaproteobacteriales	Burkholderiaceae	Rugamonas
ASV174	Glacial	LacAmpRUFtf-a	0	Bacteria	Proteobacteria	Gammaproteobacteria	Betaproteobacteriales	Burkholderiaceae	Rugamonas
ASV174	Glacial	LacAmpRUFtf-b	0	Bacteria	Proteobacteria	Gammaproteobacteria	Betaproteobacteriales	Burkholderiaceae	Rugamonas
ASV174	Glacial	LacAmpRUFtf-c	0	Bacteria	Proteobacteria	Gammaproteobacteria	Betaproteobacteriales	Burkholderiaceae	Rugamonas
ASV1744	Baie de la Table	BdT0-2	0	Bacteria	Proteobacteria	Alphaproteobacteria	Rhizobiales	Stappiaceae	Pseudovibrio
ASV1744	Control	Ctr-tf-IIb	0	Bacteria	Proteobacteria	Alphaproteobacteria	Rhizobiales	Stappiaceae	Pseudovibrio
ASV1744	Non-glacial	LacADNRUFtfa	0	Bacteria	Proteobacteria	Alphaproteobacteria	Rhizobiales	Stappiaceae	Pseudovibrio
ASV1744	Non-glacial	LacADNRUFtfb	0	Bacteria	Proteobacteria	Alphaproteobacteria	Rhizobiales	Stappiaceae	Pseudovibrio
ASV1744	Non-glacial	LacADNRUFtfc	0	Bacteria	Proteobacteria	Alphaproteobacteria	Rhizobiales	Stappiaceae	Pseudovibrio
ASV1744	Glacial	LacAmpRUFtf-a	0	Bacteria	Proteobacteria	Alphaproteobacteria	Rhizobiales	Stappiaceae	Pseudovibrio
ASV1744	Glacial	LacAmpRUFtf-b	0	Bacteria	Proteobacteria	Alphaproteobacteria	Rhizobiales	Stappiaceae	Pseudovibrio
ASV1744	Glacial	LacAmpRUFtf-c	0	Bacteria	Proteobacteria	Alphaproteobacteria	Rhizobiales	Stappiaceae	Pseudovibrio
ASV1745	Baie de la Table	BdT0-2	0	Bacteria	Proteobacteria	Gammaproteobacteria	Pseudomonadales	Saccharospirillaceae	Oleibacter
ASV1745	Control	Ctr-tf-IIb	0	Bacteria	Proteobacteria	Gammaproteobacteria	Pseudomonadales	Saccharospirillaceae	Oleibacter
ASV1745	Non-glacial	LacADNRUFtfa	0	Bacteria	Proteobacteria	Gammaproteobacteria	Pseudomonadales	Saccharospirillaceae	Oleibacter
ASV1745	Non-glacial	LacADNRUFtfb	0	Bacteria	Proteobacteria	Gammaproteobacteria	Pseudomonadales	Saccharospirillaceae	Oleibacter
ASV1745	Non-glacial	LacADNRUFtfc	0	Bacteria	Proteobacteria	Gammaproteobacteria	Pseudomonadales	Saccharospirillaceae	Oleibacter
ASV1745	Glacial	LacAmpRUFtf-a	0	Bacteria	Proteobacteria	Gammaproteobacteria	Pseudomonadales	Saccharospirillaceae	Oleibacter
ASV1745	Glacial	LacAmpRUFtf-b	0	Bacteria	Proteobacteria	Gammaproteobacteria	Pseudomonadales	Saccharospirillaceae	Oleibacter
ASV1745	Glacial	LacAmpRUFtf-c	0	Bacteria	Proteobacteria	Gammaproteobacteria	Pseudomonadales	Saccharospirillaceae	Oleibacter
ASV1749	Baie de la Table	BdT0-2	0	Bacteria	Firmicutes	Bacilli_A	Paenibacillales	Paenibacillaceae	Paenibacillus
ASV1749	Control	Ctr-tf-IIb	0	Bacteria	Firmicutes	Bacilli_A	Paenibacillales	Paenibacillaceae	Paenibacillus
ASV1749	Non-glacial	LacADNRUFtfa	0	Bacteria	Firmicutes	Bacilli_A	Paenibacillales	Paenibacillaceae	Paenibacillus
ASV1749	Non-glacial	LacADNRUFtfb	0	Bacteria	Firmicutes	Bacilli_A	Paenibacillales	Paenibacillaceae	Paenibacillus
ASV1749	Non-glacial	LacADNRUFtfc	0	Bacteria	Firmicutes	Bacilli_A	Paenibacillales	Paenibacillaceae	Paenibacillus
ASV1749	Glacial	LacAmpRUFtf-a	0	Bacteria	Firmicutes	Bacilli_A	Paenibacillales	Paenibacillaceae	Paenibacillus
ASV1749	Glacial	LacAmpRUFtf-b	0	Bacteria	Firmicutes	Bacilli_A	Paenibacillales	Paenibacillaceae	Paenibacillus
ASV1749	Glacial	LacAmpRUFtf-c	0	Bacteria	Firmicutes	Bacilli_A	Paenibacillales	Paenibacillaceae	Paenibacillus
ASV175	Glacial	LacAmpRUFtf-a	0,001527086	Bacteria	Bacteroidota	Bacteroidia	Flavobacteriales	Flavobacteriaceae	Cellulophaga
ASV175	Non-glacial	LacADNRUFtfa	0,00092383	Bacteria	Bacteroidota	Bacteroidia	Flavobacteriales	Flavobacteriaceae	Cellulophaga

ASV175	Non-glacial	LacADNRUFtfb	0,000758693	Bacteria	Bacteroidota	Bacteroidia	Flavobacteriales	Flavobacteriaceae	Cellulophaga
ASV175	Baie de la Table	BdT0-2	0,000437206	Bacteria	Bacteroidota	Bacteroidia	Flavobacteriales	Flavobacteriaceae	Cellulophaga
ASV175	Control	Ctr-tf-IIb	0,000252415	Bacteria	Bacteroidota	Bacteroidia	Flavobacteriales	Flavobacteriaceae	Cellulophaga
ASV175	Non-glacial	LacADNRUFtfc	0,000121242	Bacteria	Bacteroidota	Bacteroidia	Flavobacteriales	Flavobacteriaceae	Cellulophaga
ASV175	Glacial	LacAmpRUFtf-b	0	Bacteria	Bacteroidota	Bacteroidia	Flavobacteriales	Flavobacteriaceae	Cellulophaga
ASV175	Glacial	LacAmpRUFtf-c	0	Bacteria	Bacteroidota	Bacteroidia	Flavobacteriales	Flavobacteriaceae	Cellulophaga
ASV1754	Baie de la Table	BdT0-2	0	Bacteria	Proteobacteria	Alphaproteobacteria	UBA9655	UBA9655	UBA9655
ASV1754	Control	Ctr-tf-IIb	0	Bacteria	Proteobacteria	Alphaproteobacteria	UBA9655	UBA9655	UBA9655
ASV1754	Non-glacial	LacADNRUFtfa	0	Bacteria	Proteobacteria	Alphaproteobacteria	UBA9655	UBA9655	UBA9655
ASV1754	Non-glacial	LacADNRUFtfb	0	Bacteria	Proteobacteria	Alphaproteobacteria	UBA9655	UBA9655	UBA9655
ASV1754	Non-glacial	LacADNRUFtfc	0	Bacteria	Proteobacteria	Alphaproteobacteria	UBA9655	UBA9655	UBA9655
ASV1754	Glacial	LacAmpRUFtf-a	0	Bacteria	Proteobacteria	Alphaproteobacteria	UBA9655	UBA9655	UBA9655
ASV1754	Glacial	LacAmpRUFtf-b	0	Bacteria	Proteobacteria	Alphaproteobacteria	UBA9655	UBA9655	UBA9655
ASV1754	Glacial	LacAmpRUFtf-c	0	Bacteria	Proteobacteria	Alphaproteobacteria	UBA9655	UBA9655	UBA9655
ASV1755	Baie de la Table	BdT0-2	0	Bacteria	Actinobacteriota	Actinobacteria	Actinomycetales	Dermabacteraceae	Brachybacterium
ASV1755	Control	Ctr-tf-IIb	0	Bacteria	Actinobacteriota	Actinobacteria	Actinomycetales	Dermabacteraceae	Brachybacterium
ASV1755	Non-glacial	LacADNRUFtfa	0	Bacteria	Actinobacteriota	Actinobacteria	Actinomycetales	Dermabacteraceae	Brachybacterium
ASV1755	Non-glacial	LacADNRUFtfb	0	Bacteria	Actinobacteriota	Actinobacteria	Actinomycetales	Dermabacteraceae	Brachybacterium
ASV1755	Non-glacial	LacADNRUFtfc	0	Bacteria	Actinobacteriota	Actinobacteria	Actinomycetales	Dermabacteraceae	Brachybacterium
ASV1755	Glacial	LacAmpRUFtf-a	0	Bacteria	Actinobacteriota	Actinobacteria	Actinomycetales	Dermabacteraceae	Brachybacterium
ASV1755	Glacial	LacAmpRUFtf-b	0	Bacteria	Actinobacteriota	Actinobacteria	Actinomycetales	Dermabacteraceae	Brachybacterium
ASV1755	Glacial	LacAmpRUFtf-c	0	Bacteria	Actinobacteriota	Actinobacteria	Actinomycetales	Dermabacteraceae	Brachybacterium
ASV176	Glacial	LacAmpRUFtf-c	0,013701283	Bacteria	Actinobacteriota	Actinobacteria	Propionibacteriales	Propionibacteriaceae	Cutibacterium
ASV176	Glacial	LacAmpRUFtf-b	0,009070295	Bacteria	Actinobacteriota	Actinobacteria	Propionibacteriales	Propionibacteriaceae	Cutibacterium
ASV176	Non-glacial	LacADNRUFtfb	0,00398314	Bacteria	Actinobacteriota	Actinobacteria	Propionibacteriales	Propionibacteriaceae	Cutibacterium
ASV176	Glacial	LacAmpRUFtf-a	0,000924289	Bacteria	Actinobacteriota	Actinobacteria	Propionibacteriales	Propionibacteriaceae	Cutibacterium
ASV176	Non-glacial	LacADNRUFtfa	0,00092383	Bacteria	Actinobacteriota	Actinobacteria	Propionibacteriales	Propionibacteriaceae	Cutibacterium
ASV176	Non-glacial	LacADNRUFtfc	0,000460718	Bacteria	Actinobacteriota	Actinobacteria	Propionibacteriales	Propionibacteriaceae	Cutibacterium
ASV176	Control	Ctr-tf-IIb	0,000104171	Bacteria	Actinobacteriota	Actinobacteria	Propionibacteriales	Propionibacteriaceae	Cutibacterium
ASV176	Baie de la Table	BdT0-2	0	Bacteria	Actinobacteriota	Actinobacteria	Propionibacteriales	Propionibacteriaceae	Cutibacterium
ASV1765	Baie de la Table	BdT0-2	0	Bacteria	Bacteroidota	Bacteroidia	AKYH767-A	2013-40CM-41-45	2013-40CM-41-45
ASV1765	Control	Ctr-tf-IIb	0	Bacteria	Bacteroidota	Bacteroidia	AKYH767-A	2013-40CM-41-45	2013-40CM-41-45
ASV1765	Non-glacial	LacADNRUFtfa	0	Bacteria	Bacteroidota	Bacteroidia	AKYH767-A	2013-40CM-41-45	2013-40CM-41-45
ASV1765	Non-glacial	LacADNRUFtfb	0	Bacteria	Bacteroidota	Bacteroidia	AKYH767-A	2013-40CM-41-45	2013-40CM-41-45

ASV1765	Non-glacial	LacADNRUFtfc	0	Bacteria	Bacteroidota	Bacteroidia	AKYH767-A	2013-40CM-41-45	2013-40CM-41-45
ASV1765	Glacial	LacAmpRUFtf-a	0	Bacteria	Bacteroidota	Bacteroidia	AKYH767-A	2013-40CM-41-45	2013-40CM-41-45
ASV1765	Glacial	LacAmpRUFtf-b	0	Bacteria	Bacteroidota	Bacteroidia	AKYH767-A	2013-40CM-41-45	2013-40CM-41-45
ASV1765	Glacial	LacAmpRUFtf-c	0	Bacteria	Bacteroidota	Bacteroidia	AKYH767-A	2013-40CM-41-45	2013-40CM-41-45
ASV177	Baie de la Table	BdT0-2	0	Bacteria	Actinobacteriota	Actinobacteria	Nanopelagicales	Nanopelagicaceae	Nanopelagicus
ASV177	Control	Ctr-tf-IIb	0	Bacteria	Actinobacteriota	Actinobacteria	Nanopelagicales	Nanopelagicaceae	Nanopelagicus
ASV177	Non-glacial	LacADNRUFtfa	0	Bacteria	Actinobacteriota	Actinobacteria	Nanopelagicales	Nanopelagicaceae	Nanopelagicus
ASV177	Non-glacial	LacADNRUFtfb	0	Bacteria	Actinobacteriota	Actinobacteria	Nanopelagicales	Nanopelagicaceae	Nanopelagicus
ASV177	Non-glacial	LacADNRUFtfc	0	Bacteria	Actinobacteriota	Actinobacteria	Nanopelagicales	Nanopelagicaceae	Nanopelagicus
ASV177	Glacial	LacAmpRUFtf-a	0	Bacteria	Actinobacteriota	Actinobacteria	Nanopelagicales	Nanopelagicaceae	Nanopelagicus
ASV177	Glacial	LacAmpRUFtf-b	0	Bacteria	Actinobacteriota	Actinobacteria	Nanopelagicales	Nanopelagicaceae	Nanopelagicus
ASV177	Glacial	LacAmpRUFtf-c	0	Bacteria	Actinobacteriota	Actinobacteria	Nanopelagicales	Nanopelagicaceae	Nanopelagicus
ASV1770	Baie de la Table	BdT0-2	0	Bacteria	Proteobacteria	Gammaproteobacteria	Acidiferrobacterales	Sulfurifustaceae	Sulfuricaulis
ASV1770	Control	Ctr-tf-IIb	0	Bacteria	Proteobacteria	Gammaproteobacteria	Acidiferrobacterales	Sulfurifustaceae	Sulfuricaulis
ASV1770	Non-glacial	LacADNRUFtfa	0	Bacteria	Proteobacteria	Gammaproteobacteria	Acidiferrobacterales	Sulfurifustaceae	Sulfuricaulis
ASV1770	Non-glacial	LacADNRUFtfb	0	Bacteria	Proteobacteria	Gammaproteobacteria	Acidiferrobacterales	Sulfurifustaceae	Sulfuricaulis
ASV1770	Non-glacial	LacADNRUFtfc	0	Bacteria	Proteobacteria	Gammaproteobacteria	Acidiferrobacterales	Sulfurifustaceae	Sulfuricaulis
ASV1770	Glacial	LacAmpRUFtf-a	0	Bacteria	Proteobacteria	Gammaproteobacteria	Acidiferrobacterales	Sulfurifustaceae	Sulfuricaulis
ASV1770	Glacial	LacAmpRUFtf-b	0	Bacteria	Proteobacteria	Gammaproteobacteria	Acidiferrobacterales	Sulfurifustaceae	Sulfuricaulis
ASV1770	Glacial	LacAmpRUFtf-c	0	Bacteria	Proteobacteria	Gammaproteobacteria	Acidiferrobacterales	Sulfurifustaceae	Sulfuricaulis
ASV178	Control	Ctr-tf-IIb	0,002155544	Bacteria	Proteobacteria	Gammaproteobacteria	Pseudomonadales	Halieaceae	IMCC3088
ASV178	Non-glacial	LacADNRUFtfc	0,000727449	Bacteria	Proteobacteria	Gammaproteobacteria	Pseudomonadales	Halieaceae	IMCC3088
ASV178	Non-glacial	LacADNRUFtfb	0,000442571	Bacteria	Proteobacteria	Gammaproteobacteria	Pseudomonadales	Halieaceae	IMCC3088
ASV178	Glacial	LacAmpRUFtf-a	0,000200932	Bacteria	Proteobacteria	Gammaproteobacteria	Pseudomonadales	Halieaceae	IMCC3088
ASV178	Baie de la Table	BdT0-2	0	Bacteria	Proteobacteria	Gammaproteobacteria	Pseudomonadales	Halieaceae	IMCC3088
ASV178	Non-glacial	LacADNRUFtfa	0	Bacteria	Proteobacteria	Gammaproteobacteria	Pseudomonadales	Halieaceae	IMCC3088
ASV178	Glacial	LacAmpRUFtf-b	0	Bacteria	Proteobacteria	Gammaproteobacteria	Pseudomonadales	Halieaceae	IMCC3088
ASV178	Glacial	LacAmpRUFtf-c	0	Bacteria	Proteobacteria	Gammaproteobacteria	Pseudomonadales	Halieaceae	IMCC3088
ASV1781	Baie de la Table	BdT0-2	0	Bacteria	Verrucomicrobio	Kiritimatiellae	LD1-PB3	Lenti-01	Lenti-01
ASV1781	Control	Ctr-tf-IIb	0	Bacteria	Verrucomicrobio	Kiritimatiellae	LD1-PB3	Lenti-01	Lenti-01
ASV1781	Non-glacial	LacADNRUFtfa	0	Bacteria	Verrucomicrobio	Kiritimatiellae	LD1-PB3	Lenti-01	Lenti-01
ASV1781	Non-glacial	LacADNRUFtfb	0	Bacteria	Verrucomicrobio	Kiritimatiellae	LD1-PB3	Lenti-01	Lenti-01
ASV1781	Non-glacial	LacADNRUFtfc	0	Bacteria	Verrucomicrobio	Kiritimatiellae	LD1-PB3	Lenti-01	Lenti-01
ASV1781	Glacial	LacAmpRUFtf-a	0	Bacteria	Verrucomicrobio	Kiritimatiellae	LD1-PB3	Lenti-01	Lenti-01

ASV1781	Glacial	LacAmpRUFtf-b	0	Bacteria	Verrucomicrobio	Kiritimatiellae	LD1-PB3	Lenti-01	Lenti-01
ASV1781	Glacial	LacAmpRUFtf-c	0	Bacteria	Verrucomicrobio	Kiritimatiellae	LD1-PB3	Lenti-01	Lenti-01
ASV1792	Glacial	LacAmpRUFtf-a	0,000160746	Bacteria	Proteobacteria	Gammaproteobacteria	Nevskiales	Nevskiaceae	Hydrocarboniphaga
ASV1792	Baie de la Table	BdT0-2	0	Bacteria	Proteobacteria	Gammaproteobacteria	Nevskiales	Nevskiaceae	Hydrocarboniphaga
ASV1792	Control	Ctr-tf-IIb	0	Bacteria	Proteobacteria	Gammaproteobacteria	Nevskiales	Nevskiaceae	Hydrocarboniphaga
ASV1792	Non-glacial	LacADNRUFtfa	0	Bacteria	Proteobacteria	Gammaproteobacteria	Nevskiales	Nevskiaceae	Hydrocarboniphaga
ASV1792	Non-glacial	LacADNRUFtfb	0	Bacteria	Proteobacteria	Gammaproteobacteria	Nevskiales	Nevskiaceae	Hydrocarboniphaga
ASV1792	Non-glacial	LacADNRUFtfc	0	Bacteria	Proteobacteria	Gammaproteobacteria	Nevskiales	Nevskiaceae	Hydrocarboniphaga
ASV1792	Glacial	LacAmpRUFtf-b	0	Bacteria	Proteobacteria	Gammaproteobacteria	Nevskiales	Nevskiaceae	Hydrocarboniphaga
ASV1792	Glacial	LacAmpRUFtf-c	0	Bacteria	Proteobacteria	Gammaproteobacteria	Nevskiales	Nevskiaceae	Hydrocarboniphaga
ASV1795	Glacial	LacAmpRUFtf-a	0,000160746	Bacteria	Actinobacteriota	Actinobacteria	Actinomycetales	Microbacteriaceae	UBA1487
ASV1795	Baie de la Table	BdT0-2	0	Bacteria	Actinobacteriota	Actinobacteria	Actinomycetales	Microbacteriaceae	UBA1487
ASV1795	Control	Ctr-tf-IIb	0	Bacteria	Actinobacteriota	Actinobacteria	Actinomycetales	Microbacteriaceae	UBA1487
ASV1795	Non-glacial	LacADNRUFtfa	0	Bacteria	Actinobacteriota	Actinobacteria	Actinomycetales	Microbacteriaceae	UBA1487
ASV1795	Non-glacial	LacADNRUFtfb	0	Bacteria	Actinobacteriota	Actinobacteria	Actinomycetales	Microbacteriaceae	UBA1487
ASV1795	Non-glacial	LacADNRUFtfc	0	Bacteria	Actinobacteriota	Actinobacteria	Actinomycetales	Microbacteriaceae	UBA1487
ASV1795	Glacial	LacAmpRUFtf-b	0	Bacteria	Actinobacteriota	Actinobacteria	Actinomycetales	Microbacteriaceae	UBA1487
ASV1795	Glacial	LacAmpRUFtf-c	0	Bacteria	Actinobacteriota	Actinobacteria	Actinomycetales	Microbacteriaceae	UBA1487
ASV1802	Baie de la Table	BdT0-2	0	Bacteria	Proteobacteria	Alphaproteobacteria	Rhodospirillales	Rhodospirillaceae	Roseospirillum
ASV1802	Control	Ctr-tf-IIb	0	Bacteria	Proteobacteria	Alphaproteobacteria	Rhodospirillales	Rhodospirillaceae	Roseospirillum
ASV1802	Non-glacial	LacADNRUFtfa	0	Bacteria	Proteobacteria	Alphaproteobacteria	Rhodospirillales	Rhodospirillaceae	Roseospirillum
ASV1802	Non-glacial	LacADNRUFtfb	0	Bacteria	Proteobacteria	Alphaproteobacteria	Rhodospirillales	Rhodospirillaceae	Roseospirillum
ASV1802	Non-glacial	LacADNRUFtfc	0	Bacteria	Proteobacteria	Alphaproteobacteria	Rhodospirillales	Rhodospirillaceae	Roseospirillum
ASV1802	Glacial	LacAmpRUFtf-a	0	Bacteria	Proteobacteria	Alphaproteobacteria	Rhodospirillales	Rhodospirillaceae	Roseospirillum
ASV1802	Glacial	LacAmpRUFtf-b	0	Bacteria	Proteobacteria	Alphaproteobacteria	Rhodospirillales	Rhodospirillaceae	Roseospirillum
ASV1802	Glacial	LacAmpRUFtf-c	0	Bacteria	Proteobacteria	Alphaproteobacteria	Rhodospirillales	Rhodospirillaceae	Roseospirillum
ASV1804	Baie de la Table	BdT0-2	0	Bacteria	Bacteroidota	Bacteroidia	Chitinophagales	Chitinophagaceae	OLB11
ASV1804	Control	Ctr-tf-IIb	0	Bacteria	Bacteroidota	Bacteroidia	Chitinophagales	Chitinophagaceae	OLB11
ASV1804	Non-glacial	LacADNRUFtfa	0	Bacteria	Bacteroidota	Bacteroidia	Chitinophagales	Chitinophagaceae	OLB11
ASV1804	Non-glacial	LacADNRUFtfb	0	Bacteria	Bacteroidota	Bacteroidia	Chitinophagales	Chitinophagaceae	OLB11
ASV1804	Non-glacial	LacADNRUFtfc	0	Bacteria	Bacteroidota	Bacteroidia	Chitinophagales	Chitinophagaceae	OLB11
ASV1804	Glacial	LacAmpRUFtf-a	0	Bacteria	Bacteroidota	Bacteroidia	Chitinophagales	Chitinophagaceae	OLB11
ASV1804	Glacial	LacAmpRUFtf-b	0	Bacteria	Bacteroidota	Bacteroidia	Chitinophagales	Chitinophagaceae	OLB11
ASV1804	Glacial	LacAmpRUFtf-c	0	Bacteria	Bacteroidota	Bacteroidia	Chitinophagales	Chitinophagaceae	OLB11

ASV1809	Baie de la Table	BdT0-2	0	Bacteria	Bacteroidota	Bacteroidia	Bacteroidales	Prolixibacteraceae	UBA6024
ASV1809	Control	Ctr-tf-IIb	0	Bacteria	Bacteroidota	Bacteroidia	Bacteroidales	Prolixibacteraceae	UBA6024
ASV1809	Non-glacial	LacADNRUFtfa	0	Bacteria	Bacteroidota	Bacteroidia	Bacteroidales	Prolixibacteraceae	UBA6024
ASV1809	Non-glacial	LacADNRUFtfb	0	Bacteria	Bacteroidota	Bacteroidia	Bacteroidales	Prolixibacteraceae	UBA6024
ASV1809	Non-glacial	LacADNRUFtfc	0	Bacteria	Bacteroidota	Bacteroidia	Bacteroidales	Prolixibacteraceae	UBA6024
ASV1809	Glacial	LacAmpRUFtf-a	0	Bacteria	Bacteroidota	Bacteroidia	Bacteroidales	Prolixibacteraceae	UBA6024
ASV1809	Glacial	LacAmpRUFtf-b	0	Bacteria	Bacteroidota	Bacteroidia	Bacteroidales	Prolixibacteraceae	UBA6024
ASV1809	Glacial	LacAmpRUFtf-c	0	Bacteria	Bacteroidota	Bacteroidia	Bacteroidales	Prolixibacteraceae	UBA6024
ASV182	Baie de la Table	BdT0-2	0,001202317	Bacteria	Bacteroidota	Bacteroidia	Flavobacteriales	Flavobacteriaceae	MED-G11
ASV182	Non-glacial	LacADNRUFtfa	0,000415724	Bacteria	Bacteroidota	Bacteroidia	Flavobacteriales	Flavobacteriaceae	MED-G11
ASV182	Non-glacial	LacADNRUFtfb	0,000231823	Bacteria	Bacteroidota	Bacteroidia	Flavobacteriales	Flavobacteriaceae	MED-G11
ASV182	Control	Ctr-tf-IIb	0,000108178	Bacteria	Bacteroidota	Bacteroidia	Flavobacteriales	Flavobacteriaceae	MED-G11
ASV182	Non-glacial	LacADNRUFtfc	0	Bacteria	Bacteroidota	Bacteroidia	Flavobacteriales	Flavobacteriaceae	MED-G11
ASV182	Glacial	LacAmpRUFtf-a	0	Bacteria	Bacteroidota	Bacteroidia	Flavobacteriales	Flavobacteriaceae	MED-G11
ASV182	Glacial	LacAmpRUFtf-b	0	Bacteria	Bacteroidota	Bacteroidia	Flavobacteriales	Flavobacteriaceae	MED-G11
ASV182	Glacial	LacAmpRUFtf-c	0	Bacteria	Bacteroidota	Bacteroidia	Flavobacteriales	Flavobacteriaceae	MED-G11
ASV1823	Baie de la Table	BdT0-2	0	Bacteria	Actinobacteriota	Acidimicrobiia	Microtrichales	Microtrichaceae	IMCC26207
ASV1823	Control	Ctr-tf-IIb	0	Bacteria	Actinobacteriota	Acidimicrobiia	Microtrichales	Microtrichaceae	IMCC26207
ASV1823	Non-glacial	LacADNRUFtfa	0	Bacteria	Actinobacteriota	Acidimicrobiia	Microtrichales	Microtrichaceae	IMCC26207
ASV1823	Non-glacial	LacADNRUFtfb	0	Bacteria	Actinobacteriota	Acidimicrobiia	Microtrichales	Microtrichaceae	IMCC26207
ASV1823	Non-glacial	LacADNRUFtfc	0	Bacteria	Actinobacteriota	Acidimicrobiia	Microtrichales	Microtrichaceae	IMCC26207
ASV1823	Glacial	LacAmpRUFtf-a	0	Bacteria	Actinobacteriota	Acidimicrobiia	Microtrichales	Microtrichaceae	IMCC26207
ASV1823	Glacial	LacAmpRUFtf-b	0	Bacteria	Actinobacteriota	Acidimicrobiia	Microtrichales	Microtrichaceae	IMCC26207
ASV1823	Glacial	LacAmpRUFtf-c	0	Bacteria	Actinobacteriota	Acidimicrobiia	Microtrichales	Microtrichaceae	IMCC26207
ASV1836	Baie de la Table	BdT0-2	0	Bacteria	Actinobacteriota	Actinobacteria	Actinomycetales	Microbacteriaceae	Aquiluna
ASV1836	Control	Ctr-tf-IIb	0	Bacteria	Actinobacteriota	Actinobacteria	Actinomycetales	Microbacteriaceae	Aquiluna
ASV1836	Non-glacial	LacADNRUFtfa	0	Bacteria	Actinobacteriota	Actinobacteria	Actinomycetales	Microbacteriaceae	Aquiluna
ASV1836	Non-glacial	LacADNRUFtfb	0	Bacteria	Actinobacteriota	Actinobacteria	Actinomycetales	Microbacteriaceae	Aquiluna
ASV1836	Non-glacial	LacADNRUFtfc	0	Bacteria	Actinobacteriota	Actinobacteria	Actinomycetales	Microbacteriaceae	Aquiluna
ASV1836	Glacial	LacAmpRUFtf-a	0	Bacteria	Actinobacteriota	Actinobacteria	Actinomycetales	Microbacteriaceae	Aquiluna
ASV1836	Glacial	LacAmpRUFtf-b	0	Bacteria	Actinobacteriota	Actinobacteria	Actinomycetales	Microbacteriaceae	Aquiluna
ASV1836	Glacial	LacAmpRUFtf-c	0	Bacteria	Actinobacteriota	Actinobacteria	Actinomycetales	Microbacteriaceae	Aquiluna
ASV184	Baie de la Table	BdT0-2	0	Bacteria	Actinobacteriota	Actinobacteria	Nanopelagicales	Nanopelagicaceae	AAA044-D11
ASV184	Control	Ctr-tf-IIb	0	Bacteria	Actinobacteriota	Actinobacteria	Nanopelagicales	Nanopelagicaceae	AAA044-D11

ASV184	Non-glacial	LacADNRUFtfa	0	Bacteria	Actinobacteriota	Actinobacteria	Nanopelagicales	Nanopelagicaceae	AAA044-D11
ASV184	Non-glacial	LacADNRUFtfb	0	Bacteria	Actinobacteriota	Actinobacteria	Nanopelagicales	Nanopelagicaceae	AAA044-D11
ASV184	Non-glacial	LacADNRUFtfc	0	Bacteria	Actinobacteriota	Actinobacteria	Nanopelagicales	Nanopelagicaceae	AAA044-D11
ASV184	Glacial	LacAmpRUFtf-a	0	Bacteria	Actinobacteriota	Actinobacteria	Nanopelagicales	Nanopelagicaceae	AAA044-D11
ASV184	Glacial	LacAmpRUFtf-b	0	Bacteria	Actinobacteriota	Actinobacteria	Nanopelagicales	Nanopelagicaceae	AAA044-D11
ASV184	Glacial	LacAmpRUFtf-c	0	Bacteria	Actinobacteriota	Actinobacteria	Nanopelagicales	Nanopelagicaceae	AAA044-D11
ASV1845	Baie de la Table	BdT0-2	0	Bacteria	Proteobacteria	Gammaproteobacteria	Coxiellales	Coxiellaceae	Coxiella
ASV1845	Control	Ctr-tf-IIb	0	Bacteria	Proteobacteria	Gammaproteobacteria	Coxiellales	Coxiellaceae	Coxiella
ASV1845	Non-glacial	LacADNRUFtfa	0	Bacteria	Proteobacteria	Gammaproteobacteria	Coxiellales	Coxiellaceae	Coxiella
ASV1845	Non-glacial	LacADNRUFtfb	0	Bacteria	Proteobacteria	Gammaproteobacteria	Coxiellales	Coxiellaceae	Coxiella
ASV1845	Non-glacial	LacADNRUFtfc	0	Bacteria	Proteobacteria	Gammaproteobacteria	Coxiellales	Coxiellaceae	Coxiella
ASV1845	Glacial	LacAmpRUFtf-a	0	Bacteria	Proteobacteria	Gammaproteobacteria	Coxiellales	Coxiellaceae	Coxiella
ASV1845	Glacial	LacAmpRUFtf-b	0	Bacteria	Proteobacteria	Gammaproteobacteria	Coxiellales	Coxiellaceae	Coxiella
ASV1845	Glacial	LacAmpRUFtf-c	0	Bacteria	Proteobacteria	Gammaproteobacteria	Coxiellales	Coxiellaceae	Coxiella
ASV1847	Baie de la Table	BdT0-2	0	Bacteria	Proteobacteria	Alphaproteobacteria	Rhizobiales	Beijerinckiaceae	Bosea
ASV1847	Control	Ctr-tf-IIb	0	Bacteria	Proteobacteria	Alphaproteobacteria	Rhizobiales	Beijerinckiaceae	Bosea
ASV1847	Non-glacial	LacADNRUFtfa	0	Bacteria	Proteobacteria	Alphaproteobacteria	Rhizobiales	Beijerinckiaceae	Bosea
ASV1847	Non-glacial	LacADNRUFtfb	0	Bacteria	Proteobacteria	Alphaproteobacteria	Rhizobiales	Beijerinckiaceae	Bosea
ASV1847	Non-glacial	LacADNRUFtfc	0	Bacteria	Proteobacteria	Alphaproteobacteria	Rhizobiales	Beijerinckiaceae	Bosea
ASV1847	Glacial	LacAmpRUFtf-a	0	Bacteria	Proteobacteria	Alphaproteobacteria	Rhizobiales	Beijerinckiaceae	Bosea
ASV1847	Glacial	LacAmpRUFtf-b	0	Bacteria	Proteobacteria	Alphaproteobacteria	Rhizobiales	Beijerinckiaceae	Bosea
ASV1847	Glacial	LacAmpRUFtf-c	0	Bacteria	Proteobacteria	Alphaproteobacteria	Rhizobiales	Beijerinckiaceae	Bosea
ASV1848	Baie de la Table	BdT0-2	0	Archaea	Thermoplasmato	MGII	MGII	MGIIA	UBA120
ASV1848	Control	Ctr-tf-IIb	0	Archaea	Thermoplasmato	MGII	MGII	MGIIA	UBA120
ASV1848	Non-glacial	LacADNRUFtfa	0	Archaea	Thermoplasmato	MGII	MGII	MGIIA	UBA120
ASV1848	Non-glacial	LacADNRUFtfb	0	Archaea	Thermoplasmato	MGII	MGII	MGIIA	UBA120
ASV1848	Non-glacial	LacADNRUFtfc	0	Archaea	Thermoplasmato	MGII	MGII	MGIIA	UBA120
ASV1848	Glacial	LacAmpRUFtf-a	0	Archaea	Thermoplasmato	MGII	MGII	MGIIA	UBA120
ASV1848	Glacial	LacAmpRUFtf-b	0	Archaea	Thermoplasmato	MGII	MGII	MGIIA	UBA120
ASV1848	Glacial	LacAmpRUFtf-c	0	Archaea	Thermoplasmato	MGII	MGII	MGIIA	UBA120
ASV1852	Baie de la Table	BdT0-2	0	Bacteria	Verrucomicrobio	Verrucomicrobiae	Opitutales	Opitutaceae	Didymococcus
ASV1852	Control	Ctr-tf-IIb	0	Bacteria	Verrucomicrobio	Verrucomicrobiae	Opitutales	Opitutaceae	Didymococcus
ASV1852	Non-glacial	LacADNRUFtfa	0	Bacteria	Verrucomicrobio	Verrucomicrobiae	Opitutales	Opitutaceae	Didymococcus
ASV1852	Non-glacial	LacADNRUFtfb	0	Bacteria	Verrucomicrobio	Verrucomicrobiae	Opitutales	Opitutaceae	Didymococcus

ASV1852	Non-glacial	LacADNRUFtfc	0	Bacteria	Verrucomicrobio	Verrucomicrobiae	Opitutales	Opitutaceae	Didymococcus
ASV1852	Glacial	LacAmpRUFtf-a	0	Bacteria	Verrucomicrobio	Verrucomicrobiae	Opitutales	Opitutaceae	Didymococcus
ASV1852	Glacial	LacAmpRUFtf-b	0	Bacteria	Verrucomicrobio	Verrucomicrobiae	Opitutales	Opitutaceae	Didymococcus
ASV1852	Glacial	LacAmpRUFtf-c	0	Bacteria	Verrucomicrobio	Verrucomicrobiae	Opitutales	Opitutaceae	Didymococcus
ASV1854	Baie de la Table	BdT0-2	0	Bacteria	Bacteroidota	Bacteroidia	Chitinophagales	Saprospiraceae	Saprospira
ASV1854	Control	Ctr-tf-IIb	0	Bacteria	Bacteroidota	Bacteroidia	Chitinophagales	Saprospiraceae	Saprospira
ASV1854	Non-glacial	LacADNRUFtfa	0	Bacteria	Bacteroidota	Bacteroidia	Chitinophagales	Saprospiraceae	Saprospira
ASV1854	Non-glacial	LacADNRUFtfb	0	Bacteria	Bacteroidota	Bacteroidia	Chitinophagales	Saprospiraceae	Saprospira
ASV1854	Non-glacial	LacADNRUFtfc	0	Bacteria	Bacteroidota	Bacteroidia	Chitinophagales	Saprospiraceae	Saprospira
ASV1854	Glacial	LacAmpRUFtf-a	0	Bacteria	Bacteroidota	Bacteroidia	Chitinophagales	Saprospiraceae	Saprospira
ASV1854	Glacial	LacAmpRUFtf-b	0	Bacteria	Bacteroidota	Bacteroidia	Chitinophagales	Saprospiraceae	Saprospira
ASV1854	Glacial	LacAmpRUFtf-c	0	Bacteria	Bacteroidota	Bacteroidia	Chitinophagales	Saprospiraceae	Saprospira
ASV1855	Baie de la Table	BdT0-2	0	Bacteria	Cyanobacteriota	Cyanobacteriia	Pseudanabaenales	Pseudanabaenaceae	PCC-7502
ASV1855	Control	Ctr-tf-IIb	0	Bacteria	Cyanobacteriota	Cyanobacteriia	Pseudanabaenales	Pseudanabaenaceae	PCC-7502
ASV1855	Non-glacial	LacADNRUFtfa	0	Bacteria	Cyanobacteriota	Cyanobacteriia	Pseudanabaenales	Pseudanabaenaceae	PCC-7502
ASV1855	Non-glacial	LacADNRUFtfb	0	Bacteria	Cyanobacteriota	Cyanobacteriia	Pseudanabaenales	Pseudanabaenaceae	PCC-7502
ASV1855	Non-glacial	LacADNRUFtfc	0	Bacteria	Cyanobacteriota	Cyanobacteriia	Pseudanabaenales	Pseudanabaenaceae	PCC-7502
ASV1855	Glacial	LacAmpRUFtf-a	0	Bacteria	Cyanobacteriota	Cyanobacteriia	Pseudanabaenales	Pseudanabaenaceae	PCC-7502
ASV1855	Glacial	LacAmpRUFtf-b	0	Bacteria	Cyanobacteriota	Cyanobacteriia	Pseudanabaenales	Pseudanabaenaceae	PCC-7502
ASV1855	Glacial	LacAmpRUFtf-c	0	Bacteria	Cyanobacteriota	Cyanobacteriia	Pseudanabaenales	Pseudanabaenaceae	PCC-7502
ASV187	Glacial	LacAmpRUFtf-a	0,008921395	Bacteria	Proteobacteria	Alphaproteobacteria	Rhodobacterales	Rhodobacteraceae	Thalassobacter
ASV187	Glacial	LacAmpRUFtf-b	0,000863838	Bacteria	Proteobacteria	Alphaproteobacteria	Rhodobacterales	Rhodobacteraceae	Thalassobacter
ASV187	Glacial	LacAmpRUFtf-c	0,000580973	Bacteria	Proteobacteria	Alphaproteobacteria	Rhodobacterales	Rhodobacteraceae	Thalassobacter
ASV187	Baie de la Table	BdT0-2	0	Bacteria	Proteobacteria	Alphaproteobacteria	Rhodobacterales	Rhodobacteraceae	Thalassobacter
ASV187	Control	Ctr-tf-IIb	0	Bacteria	Proteobacteria	Alphaproteobacteria	Rhodobacterales	Rhodobacteraceae	Thalassobacter
ASV187	Non-glacial	LacADNRUFtfa	0	Bacteria	Proteobacteria	Alphaproteobacteria	Rhodobacterales	Rhodobacteraceae	Thalassobacter
ASV187	Non-glacial	LacADNRUFtfb	0	Bacteria	Proteobacteria	Alphaproteobacteria	Rhodobacterales	Rhodobacteraceae	Thalassobacter
ASV187	Non-glacial	LacADNRUFtfc	0	Bacteria	Proteobacteria	Alphaproteobacteria	Rhodobacterales	Rhodobacteraceae	Thalassobacter
ASV1874	Baie de la Table	BdT0-2	0	Bacteria	Proteobacteria	Alphaproteobacteria	Micavibrionales	Micavibrionaceae	UBA2705
ASV1874	Control	Ctr-tf-IIb	0	Bacteria	Proteobacteria	Alphaproteobacteria	Micavibrionales	Micavibrionaceae	UBA2705
ASV1874	Non-glacial	LacADNRUFtfa	0	Bacteria	Proteobacteria	Alphaproteobacteria	Micavibrionales	Micavibrionaceae	UBA2705
ASV1874	Non-glacial	LacADNRUFtfb	0	Bacteria	Proteobacteria	Alphaproteobacteria	Micavibrionales	Micavibrionaceae	UBA2705
ASV1874	Non-glacial	LacADNRUFtfc	0	Bacteria	Proteobacteria	Alphaproteobacteria	Micavibrionales	Micavibrionaceae	UBA2705
ASV1874	Glacial	LacAmpRUFtf-a	0	Bacteria	Proteobacteria	Alphaproteobacteria	Micavibrionales	Micavibrionaceae	UBA2705

ASV1874	Glacial	LacAmpRUFtf-b	0	Bacteria	Proteobacteria	Alphaproteobacteria	Micavibrionales	Micavibrionaceae	UBA2705
ASV1874	Glacial	LacAmpRUFtf-c	0	Bacteria	Proteobacteria	Alphaproteobacteria	Micavibrionales	Micavibrionaceae	UBA2705
ASV1877	Baie de la Table	BdT0-2	0	Bacteria	Bacteroidota	Bacteroidia	Flavobacteriales	Flavobacteriaceae	Tamlana
ASV1877	Control	Ctr-tf-IIb	0	Bacteria	Bacteroidota	Bacteroidia	Flavobacteriales	Flavobacteriaceae	Tamlana
ASV1877	Non-glacial	LacADNRUFtfa	0	Bacteria	Bacteroidota	Bacteroidia	Flavobacteriales	Flavobacteriaceae	Tamlana
ASV1877	Non-glacial	LacADNRUFtfb	0	Bacteria	Bacteroidota	Bacteroidia	Flavobacteriales	Flavobacteriaceae	Tamlana
ASV1877	Non-glacial	LacADNRUFtfc	0	Bacteria	Bacteroidota	Bacteroidia	Flavobacteriales	Flavobacteriaceae	Tamlana
ASV1877	Glacial	LacAmpRUFtf-a	0	Bacteria	Bacteroidota	Bacteroidia	Flavobacteriales	Flavobacteriaceae	Tamlana
ASV1877	Glacial	LacAmpRUFtf-b	0	Bacteria	Bacteroidota	Bacteroidia	Flavobacteriales	Flavobacteriaceae	Tamlana
ASV1877	Glacial	LacAmpRUFtf-c	0	Bacteria	Bacteroidota	Bacteroidia	Flavobacteriales	Flavobacteriaceae	Tamlana
ASV1882	Baie de la Table	BdT0-2	0	Bacteria	Bacteroidota	Rhodothermia	Balneolales	Balneolaceae	Gracilimonas
ASV1882	Control	Ctr-tf-IIb	0	Bacteria	Bacteroidota	Rhodothermia	Balneolales	Balneolaceae	Gracilimonas
ASV1882	Non-glacial	LacADNRUFtfa	0	Bacteria	Bacteroidota	Rhodothermia	Balneolales	Balneolaceae	Gracilimonas
ASV1882	Non-glacial	LacADNRUFtfb	0	Bacteria	Bacteroidota	Rhodothermia	Balneolales	Balneolaceae	Gracilimonas
ASV1882	Non-glacial	LacADNRUFtfc	0	Bacteria	Bacteroidota	Rhodothermia	Balneolales	Balneolaceae	Gracilimonas
ASV1882	Glacial	LacAmpRUFtf-a	0	Bacteria	Bacteroidota	Rhodothermia	Balneolales	Balneolaceae	Gracilimonas
ASV1882	Glacial	LacAmpRUFtf-b	0	Bacteria	Bacteroidota	Rhodothermia	Balneolales	Balneolaceae	Gracilimonas
ASV1882	Glacial	LacAmpRUFtf-c	0	Bacteria	Bacteroidota	Rhodothermia	Balneolales	Balneolaceae	Gracilimonas
ASV19	Glacial	LacAmpRUFtf-b	0,073966094	Bacteria	Proteobacteria	Gammaproteobacteria	Pseudomonadales	Marinomonadaceae	Marinomonas
ASV19	Control	Ctr-tf-IIb	0,03125939	Bacteria	Proteobacteria	Gammaproteobacteria	Pseudomonadales	Marinomonadaceae	Marinomonas
ASV19	Non-glacial	LacADNRUFtfc	0,024563531	Bacteria	Proteobacteria	Gammaproteobacteria	Pseudomonadales	Marinomonadaceae	Marinomonas
ASV19	Glacial	LacAmpRUFtf-a	0,023428709	Bacteria	Proteobacteria	Gammaproteobacteria	Pseudomonadales	Marinomonadaceae	Marinomonas
ASV19	Non-glacial	LacADNRUFtfb	0,005226554	Bacteria	Proteobacteria	Gammaproteobacteria	Pseudomonadales	Marinomonadaceae	Marinomonas
ASV19	Non-glacial	LacADNRUFtfa	0,003510555	Bacteria	Proteobacteria	Gammaproteobacteria	Pseudomonadales	Marinomonadaceae	Marinomonas
ASV19	Glacial	LacAmpRUFtf-c	0,002808037	Bacteria	Proteobacteria	Gammaproteobacteria	Pseudomonadales	Marinomonadaceae	Marinomonas
ASV19	Baie de la Table	BdT0-2	0	Bacteria	Proteobacteria	Gammaproteobacteria	Pseudomonadales	Marinomonadaceae	Marinomonas
ASV1906	Baie de la Table	BdT0-2	0	Bacteria	Bacteroidota	Bacteroidia	Flavobacteriales	Weeksellaceae	Chryseobacterium_A
ASV1906	Control	Ctr-tf-IIb	0	Bacteria	Bacteroidota	Bacteroidia	Flavobacteriales	Weeksellaceae	Chryseobacterium_A
ASV1906	Non-glacial	LacADNRUFtfa	0	Bacteria	Bacteroidota	Bacteroidia	Flavobacteriales	Weeksellaceae	Chryseobacterium_A
ASV1906	Non-glacial	LacADNRUFtfb	0	Bacteria	Bacteroidota	Bacteroidia	Flavobacteriales	Weeksellaceae	Chryseobacterium_A
ASV1906	Non-glacial	LacADNRUFtfc	0	Bacteria	Bacteroidota	Bacteroidia	Flavobacteriales	Weeksellaceae	Chryseobacterium_A
ASV1906	Glacial	LacAmpRUFtf-a	0	Bacteria	Bacteroidota	Bacteroidia	Flavobacteriales	Weeksellaceae	Chryseobacterium_A
ASV1906	Glacial	LacAmpRUFtf-b	0	Bacteria	Bacteroidota	Bacteroidia	Flavobacteriales	Weeksellaceae	Chryseobacterium_A
ASV1906	Glacial	LacAmpRUFtf-c	0	Bacteria	Bacteroidota	Bacteroidia	Flavobacteriales	Weeksellaceae	Chryseobacterium_A

ASV1907	Baie de la Table	BdT0-2	0	Bacteria	Actinobacteriota	Rubrobacteria	Rubrobacterales	Rubrobacteraceae	Rubrobacter_B
ASV1907	Control	Ctr-tf-IIb	0	Bacteria	Actinobacteriota	Rubrobacteria	Rubrobacterales	Rubrobacteraceae	Rubrobacter_B
ASV1907	Non-glacial	LacADNRUFtfa	0	Bacteria	Actinobacteriota	Rubrobacteria	Rubrobacterales	Rubrobacteraceae	Rubrobacter_B
ASV1907	Non-glacial	LacADNRUFtfb	0	Bacteria	Actinobacteriota	Rubrobacteria	Rubrobacterales	Rubrobacteraceae	Rubrobacter_B
ASV1907	Non-glacial	LacADNRUFtfc	0	Bacteria	Actinobacteriota	Rubrobacteria	Rubrobacterales	Rubrobacteraceae	Rubrobacter_B
ASV1907	Glacial	LacAmpRUFtf-a	0	Bacteria	Actinobacteriota	Rubrobacteria	Rubrobacterales	Rubrobacteraceae	Rubrobacter_B
ASV1907	Glacial	LacAmpRUFtf-b	0	Bacteria	Actinobacteriota	Rubrobacteria	Rubrobacterales	Rubrobacteraceae	Rubrobacter_B
ASV1907	Glacial	LacAmpRUFtf-c	0	Bacteria	Actinobacteriota	Rubrobacteria	Rubrobacterales	Rubrobacteraceae	Rubrobacter_B
ASV191	Glacial	LacAmpRUFtf-c	0,009005084	Bacteria	Proteobacteria	Alphaproteobacteria	Rhizobiales	Xanthobacteraceae	Bradyrhizobium
ASV191	Non-glacial	LacADNRUFtfb	0,003224447	Bacteria	Proteobacteria	Alphaproteobacteria	Rhizobiales	Xanthobacteraceae	Bradyrhizobium
ASV191	Glacial	LacAmpRUFtf-b	0,000971817	Bacteria	Proteobacteria	Alphaproteobacteria	Rhizobiales	Xanthobacteraceae	Bradyrhizobium
ASV191	Non-glacial	LacADNRUFtfa	0,000692873	Bacteria	Proteobacteria	Alphaproteobacteria	Rhizobiales	Xanthobacteraceae	Bradyrhizobium
ASV191	Non-glacial	LacADNRUFtfc	0,000460718	Bacteria	Proteobacteria	Alphaproteobacteria	Rhizobiales	Xanthobacteraceae	Bradyrhizobium
ASV191	Glacial	LacAmpRUFtf-a	0,000200932	Bacteria	Proteobacteria	Alphaproteobacteria	Rhizobiales	Xanthobacteraceae	Bradyrhizobium
ASV191	Control	Ctr-tf-IIb	6,01E-05	Bacteria	Proteobacteria	Alphaproteobacteria	Rhizobiales	Xanthobacteraceae	Bradyrhizobium
ASV191	Baie de la Table	BdT0-2	0	Bacteria	Proteobacteria	Alphaproteobacteria	Rhizobiales	Xanthobacteraceae	Bradyrhizobium
ASV1914	Baie de la Table	BdT0-2	0	Bacteria	Proteobacteria	Gammaproteobacteria	Betaproteobacteriales	Nitrosomonadaceae	Nitrosospira
ASV1914	Control	Ctr-tf-IIb	0	Bacteria	Proteobacteria	Gammaproteobacteria	Betaproteobacteriales	Nitrosomonadaceae	Nitrosospira
ASV1914	Non-glacial	LacADNRUFtfa	0	Bacteria	Proteobacteria	Gammaproteobacteria	Betaproteobacteriales	Nitrosomonadaceae	Nitrosospira
ASV1914	Non-glacial	LacADNRUFtfb	0	Bacteria	Proteobacteria	Gammaproteobacteria	Betaproteobacteriales	Nitrosomonadaceae	Nitrosospira
ASV1914	Non-glacial	LacADNRUFtfc	0	Bacteria	Proteobacteria	Gammaproteobacteria	Betaproteobacteriales	Nitrosomonadaceae	Nitrosospira
ASV1914	Glacial	LacAmpRUFtf-a	0	Bacteria	Proteobacteria	Gammaproteobacteria	Betaproteobacteriales	Nitrosomonadaceae	Nitrosospira
ASV1914	Glacial	LacAmpRUFtf-b	0	Bacteria	Proteobacteria	Gammaproteobacteria	Betaproteobacteriales	Nitrosomonadaceae	Nitrosospira
ASV1914	Glacial	LacAmpRUFtf-c	0	Bacteria	Proteobacteria	Gammaproteobacteria	Betaproteobacteriales	Nitrosomonadaceae	Nitrosospira
ASV1919	Baie de la Table	BdT0-2	0	Bacteria	Bacteroidota	Bacteroidia	Flavobacteriales	Flavobacteriaceae	Lacinutrix_A
ASV1919	Control	Ctr-tf-IIb	0	Bacteria	Bacteroidota	Bacteroidia	Flavobacteriales	Flavobacteriaceae	Lacinutrix_A
ASV1919	Non-glacial	LacADNRUFtfa	0	Bacteria	Bacteroidota	Bacteroidia	Flavobacteriales	Flavobacteriaceae	Lacinutrix_A
ASV1919	Non-glacial	LacADNRUFtfb	0	Bacteria	Bacteroidota	Bacteroidia	Flavobacteriales	Flavobacteriaceae	Lacinutrix_A
ASV1919	Non-glacial	LacADNRUFtfc	0	Bacteria	Bacteroidota	Bacteroidia	Flavobacteriales	Flavobacteriaceae	Lacinutrix_A
ASV1919	Glacial	LacAmpRUFtf-a	0	Bacteria	Bacteroidota	Bacteroidia	Flavobacteriales	Flavobacteriaceae	Lacinutrix_A
ASV1919	Glacial	LacAmpRUFtf-b	0	Bacteria	Bacteroidota	Bacteroidia	Flavobacteriales	Flavobacteriaceae	Lacinutrix_A
ASV1919	Glacial	LacAmpRUFtf-c	0	Bacteria	Bacteroidota	Bacteroidia	Flavobacteriales	Flavobacteriaceae	Lacinutrix_A
ASV1920	Baie de la Table	BdT0-2	0	Bacteria	Nitrospirota	Thermodesulfovibrionia	Thermodesulfovibrionales	SM23-35	0-14-3-00-41-53
ASV1920	Control	Ctr-tf-IIb	0	Bacteria	Nitrospirota	Thermodesulfovibrionia	Thermodesulfovibrionales	SM23-35	0-14-3-00-41-53

ASV192(	Non-glacial	LacADNRUFtfa	0	Bacteria	Nitrospirota	Thermodesulfovibrionia	Thermodesulfovibrionales	SM23-35	0-14-3-00-41-53
ASV192(	Non-glacial	LacADNRUFtfb	0	Bacteria	Nitrospirota	Thermodesulfovibrionia	Thermodesulfovibrionales	SM23-35	0-14-3-00-41-53
ASV192(	Non-glacial	LacADNRUFtfc	0	Bacteria	Nitrospirota	Thermodesulfovibrionia	Thermodesulfovibrionales	SM23-35	0-14-3-00-41-53
ASV192(	Glacial	LacAmpRUFtf-a	0	Bacteria	Nitrospirota	Thermodesulfovibrionia	Thermodesulfovibrionales	SM23-35	0-14-3-00-41-53
ASV192(	Glacial	LacAmpRUFtf-b	0	Bacteria	Nitrospirota	Thermodesulfovibrionia	Thermodesulfovibrionales	SM23-35	0-14-3-00-41-53
ASV192(	Glacial	LacAmpRUFtf-c	0	Bacteria	Nitrospirota	Thermodesulfovibrionia	Thermodesulfovibrionales	SM23-35	0-14-3-00-41-53
ASV1941	Glacial	LacAmpRUFtf-c	0,000145243	Bacteria	Firmicutes_A	Clostridia	Lachnospirales	Lachnospiraceae	Agathobacter
ASV1941	Baie de la Table	BdT0-2	0	Bacteria	Firmicutes_A	Clostridia	Lachnospirales	Lachnospiraceae	Agathobacter
ASV1941	Control	Ctr-tf-IIb	0	Bacteria	Firmicutes_A	Clostridia	Lachnospirales	Lachnospiraceae	Agathobacter
ASV1941	Non-glacial	LacADNRUFtfa	0	Bacteria	Firmicutes_A	Clostridia	Lachnospirales	Lachnospiraceae	Agathobacter
ASV1941	Non-glacial	LacADNRUFtfb	0	Bacteria	Firmicutes_A	Clostridia	Lachnospirales	Lachnospiraceae	Agathobacter
ASV1941	Non-glacial	LacADNRUFtfc	0	Bacteria	Firmicutes_A	Clostridia	Lachnospirales	Lachnospiraceae	Agathobacter
ASV1941	Glacial	LacAmpRUFtf-a	0	Bacteria	Firmicutes_A	Clostridia	Lachnospirales	Lachnospiraceae	Agathobacter
ASV1941	Glacial	LacAmpRUFtf-b	0	Bacteria	Firmicutes_A	Clostridia	Lachnospirales	Lachnospiraceae	Agathobacter
ASV1947	Baie de la Table	BdT0-2	0	Bacteria	Planctomycetota	Planctomycetes	Pirellulales	Pirellulaceae	Rubripirellula
ASV1947	Control	Ctr-tf-IIb	0	Bacteria	Planctomycetota	Planctomycetes	Pirellulales	Pirellulaceae	Rubripirellula
ASV1947	Non-glacial	LacADNRUFtfa	0	Bacteria	Planctomycetota	Planctomycetes	Pirellulales	Pirellulaceae	Rubripirellula
ASV1947	Non-glacial	LacADNRUFtfb	0	Bacteria	Planctomycetota	Planctomycetes	Pirellulales	Pirellulaceae	Rubripirellula
ASV1947	Non-glacial	LacADNRUFtfc	0	Bacteria	Planctomycetota	Planctomycetes	Pirellulales	Pirellulaceae	Rubripirellula
ASV1947	Glacial	LacAmpRUFtf-a	0	Bacteria	Planctomycetota	Planctomycetes	Pirellulales	Pirellulaceae	Rubripirellula
ASV1947	Glacial	LacAmpRUFtf-b	0	Bacteria	Planctomycetota	Planctomycetes	Pirellulales	Pirellulaceae	Rubripirellula
ASV1947	Glacial	LacAmpRUFtf-c	0	Bacteria	Planctomycetota	Planctomycetes	Pirellulales	Pirellulaceae	Rubripirellula
ASV1951	Baie de la Table	BdT0-2	0	Bacteria	Planctomycetota	Planctomycetes	Gemmatales	Gemmataceae	Gemmata
ASV1951	Control	Ctr-tf-IIb	0	Bacteria	Planctomycetota	Planctomycetes	Gemmatales	Gemmataceae	Gemmata
ASV1951	Non-glacial	LacADNRUFtfa	0	Bacteria	Planctomycetota	Planctomycetes	Gemmatales	Gemmataceae	Gemmata
ASV1951	Non-glacial	LacADNRUFtfb	0	Bacteria	Planctomycetota	Planctomycetes	Gemmatales	Gemmataceae	Gemmata
ASV1951	Non-glacial	LacADNRUFtfc	0	Bacteria	Planctomycetota	Planctomycetes	Gemmatales	Gemmataceae	Gemmata
ASV1951	Glacial	LacAmpRUFtf-a	0	Bacteria	Planctomycetota	Planctomycetes	Gemmatales	Gemmataceae	Gemmata
ASV1951	Glacial	LacAmpRUFtf-b	0	Bacteria	Planctomycetota	Planctomycetes	Gemmatales	Gemmataceae	Gemmata
ASV1951	Glacial	LacAmpRUFtf-c	0	Bacteria	Planctomycetota	Planctomycetes	Gemmatales	Gemmataceae	Gemmata
ASV195(	Baie de la Table	BdT0-2	0	Bacteria	Bdellovibrionota	Oligoflexia	Silvanigrellales	Silvanigrellaceae	Silvanigrella
ASV195(	Control	Ctr-tf-IIb	0	Bacteria	Bdellovibrionota	Oligoflexia	Silvanigrellales	Silvanigrellaceae	Silvanigrella
ASV195(	Non-glacial	LacADNRUFtfa	0	Bacteria	Bdellovibrionota	Oligoflexia	Silvanigrellales	Silvanigrellaceae	Silvanigrella
ASV195(	Non-glacial	LacADNRUFtfb	0	Bacteria	Bdellovibrionota	Oligoflexia	Silvanigrellales	Silvanigrellaceae	Silvanigrella

ASV1956	Non-glacial	LacADNRUFtfc	0	Bacteria	Bdellovibrionota	Oligoflexia	Silvanigrellales	Silvanigrellaceae	Silvanigrella
ASV1956	Glacial	LacAmpRUFtf-a	0	Bacteria	Bdellovibrionota	Oligoflexia	Silvanigrellales	Silvanigrellaceae	Silvanigrella
ASV1956	Glacial	LacAmpRUFtf-b	0	Bacteria	Bdellovibrionota	Oligoflexia	Silvanigrellales	Silvanigrellaceae	Silvanigrella
ASV1956	Glacial	LacAmpRUFtf-c	0	Bacteria	Bdellovibrionota	Oligoflexia	Silvanigrellales	Silvanigrellaceae	Silvanigrella
ASV197	Control	Ctr-tf-IIb	0,002215643	Bacteria	Bacteroidota	Bacteroidia	Flavobacteriales	Flavobacteriaceae	Lacinutrix
ASV197	Glacial	LacAmpRUFtf-b	0,001511716	Bacteria	Bacteroidota	Bacteroidia	Flavobacteriales	Flavobacteriaceae	Lacinutrix
ASV197	Glacial	LacAmpRUFtf-a	0,000642983	Bacteria	Bacteroidota	Bacteroidia	Flavobacteriales	Flavobacteriaceae	Lacinutrix
ASV197	Non-glacial	LacADNRUFtfc	0,000557711	Bacteria	Bacteroidota	Bacteroidia	Flavobacteriales	Flavobacteriaceae	Lacinutrix
ASV197	Baie de la Table	BdT0-2	0	Bacteria	Bacteroidota	Bacteroidia	Flavobacteriales	Flavobacteriaceae	Lacinutrix
ASV197	Non-glacial	LacADNRUFtfa	0	Bacteria	Bacteroidota	Bacteroidia	Flavobacteriales	Flavobacteriaceae	Lacinutrix
ASV197	Non-glacial	LacADNRUFtfb	0	Bacteria	Bacteroidota	Bacteroidia	Flavobacteriales	Flavobacteriaceae	Lacinutrix
ASV197	Glacial	LacAmpRUFtf-c	0	Bacteria	Bacteroidota	Bacteroidia	Flavobacteriales	Flavobacteriaceae	Lacinutrix
ASV1974	Baie de la Table	BdT0-2	0	Bacteria	Bacteroidota	Bacteroidia	Chitinophagales	Saprospiraceae	Lewinella_A
ASV1974	Control	Ctr-tf-IIb	0	Bacteria	Bacteroidota	Bacteroidia	Chitinophagales	Saprospiraceae	Lewinella_A
ASV1974	Non-glacial	LacADNRUFtfa	0	Bacteria	Bacteroidota	Bacteroidia	Chitinophagales	Saprospiraceae	Lewinella_A
ASV1974	Non-glacial	LacADNRUFtfb	0	Bacteria	Bacteroidota	Bacteroidia	Chitinophagales	Saprospiraceae	Lewinella_A
ASV1974	Non-glacial	LacADNRUFtfc	0	Bacteria	Bacteroidota	Bacteroidia	Chitinophagales	Saprospiraceae	Lewinella_A
ASV1974	Glacial	LacAmpRUFtf-a	0	Bacteria	Bacteroidota	Bacteroidia	Chitinophagales	Saprospiraceae	Lewinella_A
ASV1974	Glacial	LacAmpRUFtf-b	0	Bacteria	Bacteroidota	Bacteroidia	Chitinophagales	Saprospiraceae	Lewinella_A
ASV1974	Glacial	LacAmpRUFtf-c	0	Bacteria	Bacteroidota	Bacteroidia	Chitinophagales	Saprospiraceae	Lewinella_A
ASV1985	Baie de la Table	BdT0-2	0	Bacteria	Acidobacteriota	Mor1	Mor1	Mor1	Mor1
ASV1985	Control	Ctr-tf-IIb	0	Bacteria	Acidobacteriota	Mor1	Mor1	Mor1	Mor1
ASV1985	Non-glacial	LacADNRUFtfa	0	Bacteria	Acidobacteriota	Mor1	Mor1	Mor1	Mor1
ASV1985	Non-glacial	LacADNRUFtfb	0	Bacteria	Acidobacteriota	Mor1	Mor1	Mor1	Mor1
ASV1985	Non-glacial	LacADNRUFtfc	0	Bacteria	Acidobacteriota	Mor1	Mor1	Mor1	Mor1
ASV1985	Glacial	LacAmpRUFtf-a	0	Bacteria	Acidobacteriota	Mor1	Mor1	Mor1	Mor1
ASV1985	Glacial	LacAmpRUFtf-b	0	Bacteria	Acidobacteriota	Mor1	Mor1	Mor1	Mor1
ASV1985	Glacial	LacAmpRUFtf-c	0	Bacteria	Acidobacteriota	Mor1	Mor1	Mor1	Mor1
ASV199	Baie de la Table	BdT0-2	0	Bacteria	Proteobacteria	Alphaproteobacteria	Acetobacterales	Acetobacteraceae	Acidiphilium
ASV199	Control	Ctr-tf-IIb	0	Bacteria	Proteobacteria	Alphaproteobacteria	Acetobacterales	Acetobacteraceae	Acidiphilium
ASV199	Non-glacial	LacADNRUFtfa	0	Bacteria	Proteobacteria	Alphaproteobacteria	Acetobacterales	Acetobacteraceae	Acidiphilium
ASV199	Non-glacial	LacADNRUFtfb	0	Bacteria	Proteobacteria	Alphaproteobacteria	Acetobacterales	Acetobacteraceae	Acidiphilium
ASV199	Non-glacial	LacADNRUFtfc	0	Bacteria	Proteobacteria	Alphaproteobacteria	Acetobacterales	Acetobacteraceae	Acidiphilium
ASV199	Glacial	LacAmpRUFtf-a	0	Bacteria	Proteobacteria	Alphaproteobacteria	Acetobacterales	Acetobacteraceae	Acidiphilium

ASV199	Glacial	LacAmpRUFtf-b	0	Bacteria	Proteobacteria	Alphaproteobacteria	Acetobacterales	Acetobacteraceae	Acidiphilium
ASV199	Glacial	LacAmpRUFtf-c	0	Bacteria	Proteobacteria	Alphaproteobacteria	Acetobacterales	Acetobacteraceae	Acidiphilium
ASV1993	Baie de la Table	BdT0-2	0	Bacteria	Verrucomicrobio	Chlamydiia	Parachlamydiales	Parachlamydiaceae	Neochlamydia
ASV1993	Control	Ctr-tf-IIb	0	Bacteria	Verrucomicrobio	Chlamydiia	Parachlamydiales	Parachlamydiaceae	Neochlamydia
ASV1993	Non-glacial	LacADNRUFtfa	0	Bacteria	Verrucomicrobio	Chlamydiia	Parachlamydiales	Parachlamydiaceae	Neochlamydia
ASV1993	Non-glacial	LacADNRUFtfb	0	Bacteria	Verrucomicrobio	Chlamydiia	Parachlamydiales	Parachlamydiaceae	Neochlamydia
ASV1993	Non-glacial	LacADNRUFtfc	0	Bacteria	Verrucomicrobio	Chlamydiia	Parachlamydiales	Parachlamydiaceae	Neochlamydia
ASV1993	Glacial	LacAmpRUFtf-a	0	Bacteria	Verrucomicrobio	Chlamydiia	Parachlamydiales	Parachlamydiaceae	Neochlamydia
ASV1993	Glacial	LacAmpRUFtf-b	0	Bacteria	Verrucomicrobio	Chlamydiia	Parachlamydiales	Parachlamydiaceae	Neochlamydia
ASV1993	Glacial	LacAmpRUFtf-c	0	Bacteria	Verrucomicrobio	Chlamydiia	Parachlamydiales	Parachlamydiaceae	Neochlamydia
ASV1998	Baie de la Table	BdT0-2	0	Bacteria	Bdellovibrionota	Bdellovibrionia	Bdellovibrionales	Bdellovibrionaceae	Bdellovibrio_A
ASV1998	Control	Ctr-tf-IIb	0	Bacteria	Bdellovibrionota	Bdellovibrionia	Bdellovibrionales	Bdellovibrionaceae	Bdellovibrio_A
ASV1998	Non-glacial	LacADNRUFtfa	0	Bacteria	Bdellovibrionota	Bdellovibrionia	Bdellovibrionales	Bdellovibrionaceae	Bdellovibrio_A
ASV1998	Non-glacial	LacADNRUFtfb	0	Bacteria	Bdellovibrionota	Bdellovibrionia	Bdellovibrionales	Bdellovibrionaceae	Bdellovibrio_A
ASV1998	Non-glacial	LacADNRUFtfc	0	Bacteria	Bdellovibrionota	Bdellovibrionia	Bdellovibrionales	Bdellovibrionaceae	Bdellovibrio_A
ASV1998	Glacial	LacAmpRUFtf-a	0	Bacteria	Bdellovibrionota	Bdellovibrionia	Bdellovibrionales	Bdellovibrionaceae	Bdellovibrio_A
ASV1998	Glacial	LacAmpRUFtf-b	0	Bacteria	Bdellovibrionota	Bdellovibrionia	Bdellovibrionales	Bdellovibrionaceae	Bdellovibrio_A
ASV1998	Glacial	LacAmpRUFtf-c	0	Bacteria	Bdellovibrionota	Bdellovibrionia	Bdellovibrionales	Bdellovibrionaceae	Bdellovibrio_A
ASV2	Non-glacial	LacADNRUFtfa	0,386807705	Bacteria	Proteobacteria	Gammaproteobacteria	Enterobacterales	Alteromonadaceae	Colwellia
ASV2	Glacial	LacAmpRUFtf-c	0,082498184	Bacteria	Proteobacteria	Gammaproteobacteria	Enterobacterales	Alteromonadaceae	Colwellia
ASV2	Non-glacial	LacADNRUFtfb	0,037365648	Bacteria	Proteobacteria	Gammaproteobacteria	Enterobacterales	Alteromonadaceae	Colwellia
ASV2	Control	Ctr-tf-IIb	0,033823606	Bacteria	Proteobacteria	Gammaproteobacteria	Enterobacterales	Alteromonadaceae	Colwellia
ASV2	Glacial	LacAmpRUFtf-a	0,023910947	Bacteria	Proteobacteria	Gammaproteobacteria	Enterobacterales	Alteromonadaceae	Colwellia
ASV2	Non-glacial	LacADNRUFtfc	0,015082444	Bacteria	Proteobacteria	Gammaproteobacteria	Enterobacterales	Alteromonadaceae	Colwellia
ASV2	Glacial	LacAmpRUFtf-b	0,010258071	Bacteria	Proteobacteria	Gammaproteobacteria	Enterobacterales	Alteromonadaceae	Colwellia
ASV2	Baie de la Table	BdT0-2	0	Bacteria	Proteobacteria	Gammaproteobacteria	Enterobacterales	Alteromonadaceae	Colwellia
ASV2000	Baie de la Table	BdT0-2	0	Bacteria	Proteobacteria	Alphaproteobacteria	Rickettsiales	Midichloriaceae	Midichloria
ASV2000	Control	Ctr-tf-IIb	0	Bacteria	Proteobacteria	Alphaproteobacteria	Rickettsiales	Midichloriaceae	Midichloria
ASV2000	Non-glacial	LacADNRUFtfa	0	Bacteria	Proteobacteria	Alphaproteobacteria	Rickettsiales	Midichloriaceae	Midichloria
ASV2000	Non-glacial	LacADNRUFtfb	0	Bacteria	Proteobacteria	Alphaproteobacteria	Rickettsiales	Midichloriaceae	Midichloria
ASV2000	Non-glacial	LacADNRUFtfc	0	Bacteria	Proteobacteria	Alphaproteobacteria	Rickettsiales	Midichloriaceae	Midichloria
ASV2000	Glacial	LacAmpRUFtf-a	0	Bacteria	Proteobacteria	Alphaproteobacteria	Rickettsiales	Midichloriaceae	Midichloria
ASV2000	Glacial	LacAmpRUFtf-b	0	Bacteria	Proteobacteria	Alphaproteobacteria	Rickettsiales	Midichloriaceae	Midichloria
ASV2000	Glacial	LacAmpRUFtf-c	0	Bacteria	Proteobacteria	Alphaproteobacteria	Rickettsiales	Midichloriaceae	Midichloria

ASV2007	Baie de la Table	BdT0-2	0	Bacteria	Patescibacteria	Gracilibacteria	BD1-5	UBA6164	UBA5194
ASV2007	Control	Ctr-tf-IIb	0	Bacteria	Patescibacteria	Gracilibacteria	BD1-5	UBA6164	UBA5194
ASV2007	Non-glacial	LacADNRUFtfa	0	Bacteria	Patescibacteria	Gracilibacteria	BD1-5	UBA6164	UBA5194
ASV2007	Non-glacial	LacADNRUFtfb	0	Bacteria	Patescibacteria	Gracilibacteria	BD1-5	UBA6164	UBA5194
ASV2007	Non-glacial	LacADNRUFtfc	0	Bacteria	Patescibacteria	Gracilibacteria	BD1-5	UBA6164	UBA5194
ASV2007	Glacial	LacAmpRUFtf-a	0	Bacteria	Patescibacteria	Gracilibacteria	BD1-5	UBA6164	UBA5194
ASV2007	Glacial	LacAmpRUFtf-b	0	Bacteria	Patescibacteria	Gracilibacteria	BD1-5	UBA6164	UBA5194
ASV2007	Glacial	LacAmpRUFtf-c	0	Bacteria	Patescibacteria	Gracilibacteria	BD1-5	UBA6164	UBA5194
ASV2012	Baie de la Table	BdT0-2	0	Bacteria	Myxococcota	Polyangia	Kofleriales	Kofleriaceae	Haliangium
ASV2012	Control	Ctr-tf-IIb	0	Bacteria	Myxococcota	Polyangia	Kofleriales	Kofleriaceae	Haliangium
ASV2012	Non-glacial	LacADNRUFtfa	0	Bacteria	Myxococcota	Polyangia	Kofleriales	Kofleriaceae	Haliangium
ASV2012	Non-glacial	LacADNRUFtfb	0	Bacteria	Myxococcota	Polyangia	Kofleriales	Kofleriaceae	Haliangium
ASV2012	Non-glacial	LacADNRUFtfc	0	Bacteria	Myxococcota	Polyangia	Kofleriales	Kofleriaceae	Haliangium
ASV2012	Glacial	LacAmpRUFtf-a	0	Bacteria	Myxococcota	Polyangia	Kofleriales	Kofleriaceae	Haliangium
ASV2012	Glacial	LacAmpRUFtf-b	0	Bacteria	Myxococcota	Polyangia	Kofleriales	Kofleriaceae	Haliangium
ASV2012	Glacial	LacAmpRUFtf-c	0	Bacteria	Myxococcota	Polyangia	Kofleriales	Kofleriaceae	Haliangium
ASV2021	Baie de la Table	BdT0-2	0	Bacteria	Cyanobacteriota	Cyanobacteriia	Neosynechococcales	Neosynechococcaceae	Neosynechococcus
ASV2021	Control	Ctr-tf-IIb	0	Bacteria	Cyanobacteriota	Cyanobacteriia	Neosynechococcales	Neosynechococcaceae	Neosynechococcus
ASV2021	Non-glacial	LacADNRUFtfa	0	Bacteria	Cyanobacteriota	Cyanobacteriia	Neosynechococcales	Neosynechococcaceae	Neosynechococcus
ASV2021	Non-glacial	LacADNRUFtfb	0	Bacteria	Cyanobacteriota	Cyanobacteriia	Neosynechococcales	Neosynechococcaceae	Neosynechococcus
ASV2021	Non-glacial	LacADNRUFtfc	0	Bacteria	Cyanobacteriota	Cyanobacteriia	Neosynechococcales	Neosynechococcaceae	Neosynechococcus
ASV2021	Glacial	LacAmpRUFtf-a	0	Bacteria	Cyanobacteriota	Cyanobacteriia	Neosynechococcales	Neosynechococcaceae	Neosynechococcus
ASV2021	Glacial	LacAmpRUFtf-b	0	Bacteria	Cyanobacteriota	Cyanobacteriia	Neosynechococcales	Neosynechococcaceae	Neosynechococcus
ASV2021	Glacial	LacAmpRUFtf-c	0	Bacteria	Cyanobacteriota	Cyanobacteriia	Neosynechococcales	Neosynechococcaceae	Neosynechococcus
ASV2027	Baie de la Table	BdT0-2	0	Bacteria	Cyanobacteriota	Vampirovibrionia	Obscuribacterales	Obscuribacteraceae	Obscuribacter
ASV2027	Control	Ctr-tf-IIb	0	Bacteria	Cyanobacteriota	Vampirovibrionia	Obscuribacterales	Obscuribacteraceae	Obscuribacter
ASV2027	Non-glacial	LacADNRUFtfa	0	Bacteria	Cyanobacteriota	Vampirovibrionia	Obscuribacterales	Obscuribacteraceae	Obscuribacter
ASV2027	Non-glacial	LacADNRUFtfb	0	Bacteria	Cyanobacteriota	Vampirovibrionia	Obscuribacterales	Obscuribacteraceae	Obscuribacter
ASV2027	Non-glacial	LacADNRUFtfc	0	Bacteria	Cyanobacteriota	Vampirovibrionia	Obscuribacterales	Obscuribacteraceae	Obscuribacter
ASV2027	Glacial	LacAmpRUFtf-a	0	Bacteria	Cyanobacteriota	Vampirovibrionia	Obscuribacterales	Obscuribacteraceae	Obscuribacter
ASV2027	Glacial	LacAmpRUFtf-b	0	Bacteria	Cyanobacteriota	Vampirovibrionia	Obscuribacterales	Obscuribacteraceae	Obscuribacter
ASV2027	Glacial	LacAmpRUFtf-c	0	Bacteria	Cyanobacteriota	Vampirovibrionia	Obscuribacterales	Obscuribacteraceae	Obscuribacter
ASV203	Non-glacial	LacADNRUFtfb	0,000590095	Bacteria	Bacteroidota	Bacteroidia	Cytophagales	Cyclobacteriaceae	Roseivirga
ASV203	Non-glacial	LacADNRUFtfc	0,000242483	Bacteria	Bacteroidota	Bacteroidia	Cytophagales	Cyclobacteriaceae	Roseivirga

ASV203	Control	Ctr-tf-IIb	0,000148244	Bacteria	Bacteroidota	Bacteroidia	Cytophagales	Cyclobacteriaceae	Roseivirga
ASV203	Baie de la Table	BdT0-2	0	Bacteria	Bacteroidota	Bacteroidia	Cytophagales	Cyclobacteriaceae	Roseivirga
ASV203	Non-glacial	LacADNRUFtfa	0	Bacteria	Bacteroidota	Bacteroidia	Cytophagales	Cyclobacteriaceae	Roseivirga
ASV203	Glacial	LacAmpRUFtf-a	0	Bacteria	Bacteroidota	Bacteroidia	Cytophagales	Cyclobacteriaceae	Roseivirga
ASV203	Glacial	LacAmpRUFtf-b	0	Bacteria	Bacteroidota	Bacteroidia	Cytophagales	Cyclobacteriaceae	Roseivirga
ASV203	Glacial	LacAmpRUFtf-c	0	Bacteria	Bacteroidota	Bacteroidia	Cytophagales	Cyclobacteriaceae	Roseivirga
ASV203(	Baie de la Table	BdT0-2	0	Bacteria	Fusobacteriota	Fusobacteriia	Fusobacteriales	Fusobacteriaceae	Psychrilyobacter
ASV203(	Control	Ctr-tf-IIb	0	Bacteria	Fusobacteriota	Fusobacteriia	Fusobacteriales	Fusobacteriaceae	Psychrilyobacter
ASV203(	Non-glacial	LacADNRUFtfa	0	Bacteria	Fusobacteriota	Fusobacteriia	Fusobacteriales	Fusobacteriaceae	Psychrilyobacter
ASV203(	Non-glacial	LacADNRUFtfb	0	Bacteria	Fusobacteriota	Fusobacteriia	Fusobacteriales	Fusobacteriaceae	Psychrilyobacter
ASV203(	Non-glacial	LacADNRUFtfc	0	Bacteria	Fusobacteriota	Fusobacteriia	Fusobacteriales	Fusobacteriaceae	Psychrilyobacter
ASV203(	Glacial	LacAmpRUFtf-a	0	Bacteria	Fusobacteriota	Fusobacteriia	Fusobacteriales	Fusobacteriaceae	Psychrilyobacter
ASV203(	Glacial	LacAmpRUFtf-b	0	Bacteria	Fusobacteriota	Fusobacteriia	Fusobacteriales	Fusobacteriaceae	Psychrilyobacter
ASV203(	Glacial	LacAmpRUFtf-c	0	Bacteria	Fusobacteriota	Fusobacteriia	Fusobacteriales	Fusobacteriaceae	Psychrilyobacter
ASV204	Baie de la Table	BdT0-2	0	Bacteria	Omnitrophota	UBA9649	UBA9649	UBA9649	UBA9649
ASV204	Control	Ctr-tf-IIb	0	Bacteria	Omnitrophota	UBA9649	UBA9649	UBA9649	UBA9649
ASV204	Non-glacial	LacADNRUFtfa	0	Bacteria	Omnitrophota	UBA9649	UBA9649	UBA9649	UBA9649
ASV204	Non-glacial	LacADNRUFtfb	0	Bacteria	Omnitrophota	UBA9649	UBA9649	UBA9649	UBA9649
ASV204	Non-glacial	LacADNRUFtfc	0	Bacteria	Omnitrophota	UBA9649	UBA9649	UBA9649	UBA9649
ASV204	Glacial	LacAmpRUFtf-a	0	Bacteria	Omnitrophota	UBA9649	UBA9649	UBA9649	UBA9649
ASV204	Glacial	LacAmpRUFtf-b	0	Bacteria	Omnitrophota	UBA9649	UBA9649	UBA9649	UBA9649
ASV204	Glacial	LacAmpRUFtf-c	0	Bacteria	Omnitrophota	UBA9649	UBA9649	UBA9649	UBA9649
ASV205	Non-glacial	LacADNRUFtfb	0,001601686	Bacteria	Proteobacteria	Alphaproteobacteria	Acetobacterales	Acetobacteraceae	Acidocella
ASV205	Glacial	LacAmpRUFtf-c	0,001210361	Bacteria	Proteobacteria	Alphaproteobacteria	Acetobacterales	Acetobacteraceae	Acidocella
ASV205	Control	Ctr-tf-IIb	0,000769265	Bacteria	Proteobacteria	Alphaproteobacteria	Acetobacterales	Acetobacteraceae	Acidocella
ASV205	Glacial	LacAmpRUFtf-b	0,000755858	Bacteria	Proteobacteria	Alphaproteobacteria	Acetobacterales	Acetobacteraceae	Acidocella
ASV205	Non-glacial	LacADNRUFtfa	0,00060049	Bacteria	Proteobacteria	Alphaproteobacteria	Acetobacterales	Acetobacteraceae	Acidocella
ASV205	Glacial	LacAmpRUFtf-a	0,000482238	Bacteria	Proteobacteria	Alphaproteobacteria	Acetobacterales	Acetobacteraceae	Acidocella
ASV205	Non-glacial	LacADNRUFtfc	0,000266731	Bacteria	Proteobacteria	Alphaproteobacteria	Acetobacterales	Acetobacteraceae	Acidocella
ASV205	Baie de la Table	BdT0-2	0	Bacteria	Proteobacteria	Alphaproteobacteria	Acetobacterales	Acetobacteraceae	Acidocella
ASV2057	Non-glacial	LacADNRUFtfa	9,24E-05	Bacteria	Bacteroidota	Bacteroidia	Flavobacteriales	Weeksellaceae	Cloacibacterium
ASV2057	Baie de la Table	BdT0-2	0	Bacteria	Bacteroidota	Bacteroidia	Flavobacteriales	Weeksellaceae	Cloacibacterium
ASV2057	Control	Ctr-tf-IIb	0	Bacteria	Bacteroidota	Bacteroidia	Flavobacteriales	Weeksellaceae	Cloacibacterium
ASV2057	Non-glacial	LacADNRUFtfb	0	Bacteria	Bacteroidota	Bacteroidia	Flavobacteriales	Weeksellaceae	Cloacibacterium

ASV2057	Non-glacial	LacADNRUFtfc	0	Bacteria	Bacteroidota	Bacteroidia	Flavobacteriales	Weeksellaceae	Cloacibacterium
ASV2057	Glacial	LacAmpRUFtf-a	0	Bacteria	Bacteroidota	Bacteroidia	Flavobacteriales	Weeksellaceae	Cloacibacterium
ASV2057	Glacial	LacAmpRUFtf-b	0	Bacteria	Bacteroidota	Bacteroidia	Flavobacteriales	Weeksellaceae	Cloacibacterium
ASV2057	Glacial	LacAmpRUFtf-c	0	Bacteria	Bacteroidota	Bacteroidia	Flavobacteriales	Weeksellaceae	Cloacibacterium
ASV2059	Non-glacial	LacADNRUFtfa	9,24E-05	Bacteria	Patescibacteria	Microgenomatia	UBA10105	UBA927	UBA927
ASV2059	Baie de la Table	BdT0-2	0	Bacteria	Patescibacteria	Microgenomatia	UBA10105	UBA927	UBA927
ASV2059	Control	Ctr-tf-IIb	0	Bacteria	Patescibacteria	Microgenomatia	UBA10105	UBA927	UBA927
ASV2059	Non-glacial	LacADNRUFtfb	0	Bacteria	Patescibacteria	Microgenomatia	UBA10105	UBA927	UBA927
ASV2059	Non-glacial	LacADNRUFtfc	0	Bacteria	Patescibacteria	Microgenomatia	UBA10105	UBA927	UBA927
ASV2059	Glacial	LacAmpRUFtf-a	0	Bacteria	Patescibacteria	Microgenomatia	UBA10105	UBA927	UBA927
ASV2059	Glacial	LacAmpRUFtf-b	0	Bacteria	Patescibacteria	Microgenomatia	UBA10105	UBA927	UBA927
ASV2059	Glacial	LacAmpRUFtf-c	0	Bacteria	Patescibacteria	Microgenomatia	UBA10105	UBA927	UBA927
ASV2060	Non-glacial	LacADNRUFtfa	9,24E-05	Bacteria	Actinobacteriota	Actinobacteria	Corynebacteriales	Corynebacteriaceae	Rhodococcus
ASV2060	Baie de la Table	BdT0-2	0	Bacteria	Actinobacteriota	Actinobacteria	Corynebacteriales	Corynebacteriaceae	Rhodococcus
ASV2060	Control	Ctr-tf-IIb	0	Bacteria	Actinobacteriota	Actinobacteria	Corynebacteriales	Corynebacteriaceae	Rhodococcus
ASV2060	Non-glacial	LacADNRUFtfb	0	Bacteria	Actinobacteriota	Actinobacteria	Corynebacteriales	Corynebacteriaceae	Rhodococcus
ASV2060	Non-glacial	LacADNRUFtfc	0	Bacteria	Actinobacteriota	Actinobacteria	Corynebacteriales	Corynebacteriaceae	Rhodococcus
ASV2060	Glacial	LacAmpRUFtf-a	0	Bacteria	Actinobacteriota	Actinobacteria	Corynebacteriales	Corynebacteriaceae	Rhodococcus
ASV2060	Glacial	LacAmpRUFtf-b	0	Bacteria	Actinobacteriota	Actinobacteria	Corynebacteriales	Corynebacteriaceae	Rhodococcus
ASV2060	Glacial	LacAmpRUFtf-c	0	Bacteria	Actinobacteriota	Actinobacteria	Corynebacteriales	Corynebacteriaceae	Rhodococcus
ASV2066	Baie de la Table	BdT0-2	0	Archaea	Thermoplasmato	MGII	MGII	MGIIB	UBA463
ASV2066	Control	Ctr-tf-IIb	0	Archaea	Thermoplasmato	MGII	MGII	MGIIB	UBA463
ASV2066	Non-glacial	LacADNRUFtfa	0	Archaea	Thermoplasmato	MGII	MGII	MGIIB	UBA463
ASV2066	Non-glacial	LacADNRUFtfb	0	Archaea	Thermoplasmato	MGII	MGII	MGIIB	UBA463
ASV2066	Non-glacial	LacADNRUFtfc	0	Archaea	Thermoplasmato	MGII	MGII	MGIIB	UBA463
ASV2066	Glacial	LacAmpRUFtf-a	0	Archaea	Thermoplasmato	MGII	MGII	MGIIB	UBA463
ASV2066	Glacial	LacAmpRUFtf-b	0	Archaea	Thermoplasmato	MGII	MGII	MGIIB	UBA463
ASV2066	Glacial	LacAmpRUFtf-c	0	Archaea	Thermoplasmato	MGII	MGII	MGIIB	UBA463
ASV2071	Baie de la Table	BdT0-2	0	Bacteria	Proteobacteria	Alphaproteobacteria	Sphingomonadales	Sphingomonadaceae	Erythrobacter_C
ASV2071	Control	Ctr-tf-IIb	0	Bacteria	Proteobacteria	Alphaproteobacteria	Sphingomonadales	Sphingomonadaceae	Erythrobacter_C
ASV2071	Non-glacial	LacADNRUFtfa	0	Bacteria	Proteobacteria	Alphaproteobacteria	Sphingomonadales	Sphingomonadaceae	Erythrobacter_C
ASV2071	Non-glacial	LacADNRUFtfb	0	Bacteria	Proteobacteria	Alphaproteobacteria	Sphingomonadales	Sphingomonadaceae	Erythrobacter_C
ASV2071	Non-glacial	LacADNRUFtfc	0	Bacteria	Proteobacteria	Alphaproteobacteria	Sphingomonadales	Sphingomonadaceae	Erythrobacter_C
ASV2071	Glacial	LacAmpRUFtf-a	0	Bacteria	Proteobacteria	Alphaproteobacteria	Sphingomonadales	Sphingomonadaceae	Erythrobacter_C

ASV2071	Glacial	LacAmpRUFtf-b	0	Bacteria	Proteobacteria	Alphaproteobacteria	Sphingomonadales	Sphingomonadaceae	Erythrobacter_C
ASV2071	Glacial	LacAmpRUFtf-c	0	Bacteria	Proteobacteria	Alphaproteobacteria	Sphingomonadales	Sphingomonadaceae	Erythrobacter_C
ASV2073	Baie de la Table	BdT0-2	0	Bacteria	Firmicutes_A	Clostridia	Peptostreptococcales	Peptostreptococcaceae	Terrisporobacter
ASV2073	Control	Ctr-tf-IIb	0	Bacteria	Firmicutes_A	Clostridia	Peptostreptococcales	Peptostreptococcaceae	Terrisporobacter
ASV2073	Non-glacial	LacADNRUFtfa	0	Bacteria	Firmicutes_A	Clostridia	Peptostreptococcales	Peptostreptococcaceae	Terrisporobacter
ASV2073	Non-glacial	LacADNRUFtfb	0	Bacteria	Firmicutes_A	Clostridia	Peptostreptococcales	Peptostreptococcaceae	Terrisporobacter
ASV2073	Non-glacial	LacADNRUFtfc	0	Bacteria	Firmicutes_A	Clostridia	Peptostreptococcales	Peptostreptococcaceae	Terrisporobacter
ASV2073	Glacial	LacAmpRUFtf-a	0	Bacteria	Firmicutes_A	Clostridia	Peptostreptococcales	Peptostreptococcaceae	Terrisporobacter
ASV2073	Glacial	LacAmpRUFtf-b	0	Bacteria	Firmicutes_A	Clostridia	Peptostreptococcales	Peptostreptococcaceae	Terrisporobacter
ASV2073	Glacial	LacAmpRUFtf-c	0	Bacteria	Firmicutes_A	Clostridia	Peptostreptococcales	Peptostreptococcaceae	Terrisporobacter
ASV2074	Baie de la Table	BdT0-2	0	Bacteria	Firmicutes_E	Symbiobacteriia	Symbiobacteriales	Symbiobacteriaceae	Symbiobacterium
ASV2074	Control	Ctr-tf-IIb	0	Bacteria	Firmicutes_E	Symbiobacteriia	Symbiobacteriales	Symbiobacteriaceae	Symbiobacterium
ASV2074	Non-glacial	LacADNRUFtfa	0	Bacteria	Firmicutes_E	Symbiobacteriia	Symbiobacteriales	Symbiobacteriaceae	Symbiobacterium
ASV2074	Non-glacial	LacADNRUFtfb	0	Bacteria	Firmicutes_E	Symbiobacteriia	Symbiobacteriales	Symbiobacteriaceae	Symbiobacterium
ASV2074	Non-glacial	LacADNRUFtfc	0	Bacteria	Firmicutes_E	Symbiobacteriia	Symbiobacteriales	Symbiobacteriaceae	Symbiobacterium
ASV2074	Glacial	LacAmpRUFtf-a	0	Bacteria	Firmicutes_E	Symbiobacteriia	Symbiobacteriales	Symbiobacteriaceae	Symbiobacterium
ASV2074	Glacial	LacAmpRUFtf-b	0	Bacteria	Firmicutes_E	Symbiobacteriia	Symbiobacteriales	Symbiobacteriaceae	Symbiobacterium
ASV2074	Glacial	LacAmpRUFtf-c	0	Bacteria	Firmicutes_E	Symbiobacteriia	Symbiobacteriales	Symbiobacteriaceae	Symbiobacterium
ASV2075	Baie de la Table	BdT0-2	0	Bacteria	Proteobacteria	Alphaproteobacteria	Rhodobacterales	Rhodobacteraceae	Gemmobacter_A
ASV2075	Control	Ctr-tf-IIb	0	Bacteria	Proteobacteria	Alphaproteobacteria	Rhodobacterales	Rhodobacteraceae	Gemmobacter_A
ASV2075	Non-glacial	LacADNRUFtfa	0	Bacteria	Proteobacteria	Alphaproteobacteria	Rhodobacterales	Rhodobacteraceae	Gemmobacter_A
ASV2075	Non-glacial	LacADNRUFtfb	0	Bacteria	Proteobacteria	Alphaproteobacteria	Rhodobacterales	Rhodobacteraceae	Gemmobacter_A
ASV2075	Non-glacial	LacADNRUFtfc	0	Bacteria	Proteobacteria	Alphaproteobacteria	Rhodobacterales	Rhodobacteraceae	Gemmobacter_A
ASV2075	Glacial	LacAmpRUFtf-a	0	Bacteria	Proteobacteria	Alphaproteobacteria	Rhodobacterales	Rhodobacteraceae	Gemmobacter_A
ASV2075	Glacial	LacAmpRUFtf-b	0	Bacteria	Proteobacteria	Alphaproteobacteria	Rhodobacterales	Rhodobacteraceae	Gemmobacter_A
ASV2075	Glacial	LacAmpRUFtf-c	0	Bacteria	Proteobacteria	Alphaproteobacteria	Rhodobacterales	Rhodobacteraceae	Gemmobacter_A
ASV2078	Baie de la Table	BdT0-2	0	Bacteria	Proteobacteria	Alphaproteobacteria	Rickettsiales	UBA6187	1-14-0-20-39-49
ASV2078	Control	Ctr-tf-IIb	0	Bacteria	Proteobacteria	Alphaproteobacteria	Rickettsiales	UBA6187	1-14-0-20-39-49
ASV2078	Non-glacial	LacADNRUFtfa	0	Bacteria	Proteobacteria	Alphaproteobacteria	Rickettsiales	UBA6187	1-14-0-20-39-49
ASV2078	Non-glacial	LacADNRUFtfb	0	Bacteria	Proteobacteria	Alphaproteobacteria	Rickettsiales	UBA6187	1-14-0-20-39-49
ASV2078	Non-glacial	LacADNRUFtfc	0	Bacteria	Proteobacteria	Alphaproteobacteria	Rickettsiales	UBA6187	1-14-0-20-39-49
ASV2078	Glacial	LacAmpRUFtf-a	0	Bacteria	Proteobacteria	Alphaproteobacteria	Rickettsiales	UBA6187	1-14-0-20-39-49
ASV2078	Glacial	LacAmpRUFtf-b	0	Bacteria	Proteobacteria	Alphaproteobacteria	Rickettsiales	UBA6187	1-14-0-20-39-49
ASV2078	Glacial	LacAmpRUFtf-c	0	Bacteria	Proteobacteria	Alphaproteobacteria	Rickettsiales	UBA6187	1-14-0-20-39-49

ASV208	Baie de la Table	BdT0-2	0,000983714	Bacteria	Bacteroidota	Bacteroidia	Cytophagales	Cyclobacteriaceae	UBA4465
ASV208	Non-glacial	LacADNRUFtfb	0,000316122	Bacteria	Bacteroidota	Bacteroidia	Cytophagales	Cyclobacteriaceae	UBA4465
ASV208	Glacial	LacAmpRUFtf-a	0,000120559	Bacteria	Bacteroidota	Bacteroidia	Cytophagales	Cyclobacteriaceae	UBA4465
ASV208	Control	Ctr-tf-IIb	0	Bacteria	Bacteroidota	Bacteroidia	Cytophagales	Cyclobacteriaceae	UBA4465
ASV208	Non-glacial	LacADNRUFtfa	0	Bacteria	Bacteroidota	Bacteroidia	Cytophagales	Cyclobacteriaceae	UBA4465
ASV208	Non-glacial	LacADNRUFtfc	0	Bacteria	Bacteroidota	Bacteroidia	Cytophagales	Cyclobacteriaceae	UBA4465
ASV208	Glacial	LacAmpRUFtf-b	0	Bacteria	Bacteroidota	Bacteroidia	Cytophagales	Cyclobacteriaceae	UBA4465
ASV208	Glacial	LacAmpRUFtf-c	0	Bacteria	Bacteroidota	Bacteroidia	Cytophagales	Cyclobacteriaceae	UBA4465
ASV2085	Baie de la Table	BdT0-2	0	Bacteria	Proteobacteria	Gammaproteobacteria	UBA4486	UBA4486	2-02-FULL-47-50
ASV2085	Control	Ctr-tf-IIb	0	Bacteria	Proteobacteria	Gammaproteobacteria	UBA4486	UBA4486	2-02-FULL-47-50
ASV2085	Non-glacial	LacADNRUFtfa	0	Bacteria	Proteobacteria	Gammaproteobacteria	UBA4486	UBA4486	2-02-FULL-47-50
ASV2085	Non-glacial	LacADNRUFtfb	0	Bacteria	Proteobacteria	Gammaproteobacteria	UBA4486	UBA4486	2-02-FULL-47-50
ASV2085	Non-glacial	LacADNRUFtfc	0	Bacteria	Proteobacteria	Gammaproteobacteria	UBA4486	UBA4486	2-02-FULL-47-50
ASV2085	Glacial	LacAmpRUFtf-a	0	Bacteria	Proteobacteria	Gammaproteobacteria	UBA4486	UBA4486	2-02-FULL-47-50
ASV2085	Glacial	LacAmpRUFtf-b	0	Bacteria	Proteobacteria	Gammaproteobacteria	UBA4486	UBA4486	2-02-FULL-47-50
ASV2085	Glacial	LacAmpRUFtf-c	0	Bacteria	Proteobacteria	Gammaproteobacteria	UBA4486	UBA4486	2-02-FULL-47-50
ASV2087	Baie de la Table	BdT0-2	0	Bacteria	Bdellovibrionota	Oligoflexia	Oligoflexales	Oligoflexaceae	Pseudobacteriovorax
ASV2087	Control	Ctr-tf-IIb	0	Bacteria	Bdellovibrionota	Oligoflexia	Oligoflexales	Oligoflexaceae	Pseudobacteriovorax
ASV2087	Non-glacial	LacADNRUFtfa	0	Bacteria	Bdellovibrionota	Oligoflexia	Oligoflexales	Oligoflexaceae	Pseudobacteriovorax
ASV2087	Non-glacial	LacADNRUFtfb	0	Bacteria	Bdellovibrionota	Oligoflexia	Oligoflexales	Oligoflexaceae	Pseudobacteriovorax
ASV2087	Non-glacial	LacADNRUFtfc	0	Bacteria	Bdellovibrionota	Oligoflexia	Oligoflexales	Oligoflexaceae	Pseudobacteriovorax
ASV2087	Glacial	LacAmpRUFtf-a	0	Bacteria	Bdellovibrionota	Oligoflexia	Oligoflexales	Oligoflexaceae	Pseudobacteriovorax
ASV2087	Glacial	LacAmpRUFtf-b	0	Bacteria	Bdellovibrionota	Oligoflexia	Oligoflexales	Oligoflexaceae	Pseudobacteriovorax
ASV2087	Glacial	LacAmpRUFtf-c	0	Bacteria	Bdellovibrionota	Oligoflexia	Oligoflexales	Oligoflexaceae	Pseudobacteriovorax
ASV2096	Baie de la Table	BdT0-2	0	Bacteria	Myxococcota	Myxococcia	Myxococcales	Myxococcaceae	Archangium
ASV2096	Control	Ctr-tf-IIb	0	Bacteria	Myxococcota	Myxococcia	Myxococcales	Myxococcaceae	Archangium
ASV2096	Non-glacial	LacADNRUFtfa	0	Bacteria	Myxococcota	Myxococcia	Myxococcales	Myxococcaceae	Archangium
ASV2096	Non-glacial	LacADNRUFtfb	0	Bacteria	Myxococcota	Myxococcia	Myxococcales	Myxococcaceae	Archangium
ASV2096	Non-glacial	LacADNRUFtfc	0	Bacteria	Myxococcota	Myxococcia	Myxococcales	Myxococcaceae	Archangium
ASV2096	Glacial	LacAmpRUFtf-a	0	Bacteria	Myxococcota	Myxococcia	Myxococcales	Myxococcaceae	Archangium
ASV2096	Glacial	LacAmpRUFtf-b	0	Bacteria	Myxococcota	Myxococcia	Myxococcales	Myxococcaceae	Archangium
ASV2096	Glacial	LacAmpRUFtf-c	0	Bacteria	Myxococcota	Myxococcia	Myxococcales	Myxococcaceae	Archangium
ASV2097	Baie de la Table	BdT0-2	0	Bacteria	Planctomycetota	Phycisphaerae	SG8-4	SG8-4	ST-NAGAB-D1
ASV2097	Control	Ctr-tf-IIb	0	Bacteria	Planctomycetota	Phycisphaerae	SG8-4	SG8-4	ST-NAGAB-D1

ASV2097	Non-glacial	LacADNRUFtfa	0	Bacteria	Planctomycetota	Phycisphaerae	SG8-4	SG8-4	ST-NAGAB-D1
ASV2097	Non-glacial	LacADNRUFtfb	0	Bacteria	Planctomycetota	Phycisphaerae	SG8-4	SG8-4	ST-NAGAB-D1
ASV2097	Non-glacial	LacADNRUFtfc	0	Bacteria	Planctomycetota	Phycisphaerae	SG8-4	SG8-4	ST-NAGAB-D1
ASV2097	Glacial	LacAmpRUFtf-a	0	Bacteria	Planctomycetota	Phycisphaerae	SG8-4	SG8-4	ST-NAGAB-D1
ASV2097	Glacial	LacAmpRUFtf-b	0	Bacteria	Planctomycetota	Phycisphaerae	SG8-4	SG8-4	ST-NAGAB-D1
ASV2097	Glacial	LacAmpRUFtf-c	0	Bacteria	Planctomycetota	Phycisphaerae	SG8-4	SG8-4	ST-NAGAB-D1
ASV21	Glacial	LacAmpRUFtf-c	0,002662794	Bacteria	Planctomycetota	Planctomycetes	Gemmatales	Gemmataceae	UBA969
ASV21	Glacial	LacAmpRUFtf-b	0,001187777	Bacteria	Planctomycetota	Planctomycetes	Gemmatales	Gemmataceae	UBA969
ASV21	Non-glacial	LacADNRUFtfb	0,000126449	Bacteria	Planctomycetota	Planctomycetes	Gemmatales	Gemmataceae	UBA969
ASV21	Non-glacial	LacADNRUFtfc	9,70E-05	Bacteria	Planctomycetota	Planctomycetes	Gemmatales	Gemmataceae	UBA969
ASV21	Baie de la Table	BdT0-2	0	Bacteria	Planctomycetota	Planctomycetes	Gemmatales	Gemmataceae	UBA969
ASV21	Control	Ctr-tf-IIb	0	Bacteria	Planctomycetota	Planctomycetes	Gemmatales	Gemmataceae	UBA969
ASV21	Non-glacial	LacADNRUFtfa	0	Bacteria	Planctomycetota	Planctomycetes	Gemmatales	Gemmataceae	UBA969
ASV21	Glacial	LacAmpRUFtf-a	0	Bacteria	Planctomycetota	Planctomycetes	Gemmatales	Gemmataceae	UBA969
ASV210	Non-glacial	LacADNRUFtfa	0,000461915	Bacteria	Proteobacteria	Gammaproteobacteria	Pseudomonadales	Saccharospirillaceae	Oleispira
ASV210	Non-glacial	LacADNRUFtfb	0,000400421	Bacteria	Proteobacteria	Gammaproteobacteria	Pseudomonadales	Saccharospirillaceae	Oleispira
ASV210	Baie de la Table	BdT0-2	0	Bacteria	Proteobacteria	Gammaproteobacteria	Pseudomonadales	Saccharospirillaceae	Oleispira
ASV210	Control	Ctr-tf-IIb	0	Bacteria	Proteobacteria	Gammaproteobacteria	Pseudomonadales	Saccharospirillaceae	Oleispira
ASV210	Non-glacial	LacADNRUFtfc	0	Bacteria	Proteobacteria	Gammaproteobacteria	Pseudomonadales	Saccharospirillaceae	Oleispira
ASV210	Glacial	LacAmpRUFtf-a	0	Bacteria	Proteobacteria	Gammaproteobacteria	Pseudomonadales	Saccharospirillaceae	Oleispira
ASV210	Glacial	LacAmpRUFtf-b	0	Bacteria	Proteobacteria	Gammaproteobacteria	Pseudomonadales	Saccharospirillaceae	Oleispira
ASV210	Glacial	LacAmpRUFtf-c	0	Bacteria	Proteobacteria	Gammaproteobacteria	Pseudomonadales	Saccharospirillaceae	Oleispira
ASV2112	Glacial	LacAmpRUFtf-a	8,04E-05	Bacteria	Proteobacteria	Gammaproteobacteria	Betaproteobacteriales	Burkholderiaceae	Orrella
ASV2112	Baie de la Table	BdT0-2	0	Bacteria	Proteobacteria	Gammaproteobacteria	Betaproteobacteriales	Burkholderiaceae	Orrella
ASV2112	Control	Ctr-tf-IIb	0	Bacteria	Proteobacteria	Gammaproteobacteria	Betaproteobacteriales	Burkholderiaceae	Orrella
ASV2112	Non-glacial	LacADNRUFtfa	0	Bacteria	Proteobacteria	Gammaproteobacteria	Betaproteobacteriales	Burkholderiaceae	Orrella
ASV2112	Non-glacial	LacADNRUFtfb	0	Bacteria	Proteobacteria	Gammaproteobacteria	Betaproteobacteriales	Burkholderiaceae	Orrella
ASV2112	Non-glacial	LacADNRUFtfc	0	Bacteria	Proteobacteria	Gammaproteobacteria	Betaproteobacteriales	Burkholderiaceae	Orrella
ASV2112	Glacial	LacAmpRUFtf-b	0	Bacteria	Proteobacteria	Gammaproteobacteria	Betaproteobacteriales	Burkholderiaceae	Orrella
ASV2112	Glacial	LacAmpRUFtf-c	0	Bacteria	Proteobacteria	Gammaproteobacteria	Betaproteobacteriales	Burkholderiaceae	Orrella
ASV2119	Baie de la Table	BdT0-2	0	Bacteria	Firestonebacteria	D2-FULL-39-29	D2-FULL-39-29	D2-FULL-39-29	D2-FULL-39-29
ASV2119	Control	Ctr-tf-IIb	0	Bacteria	Firestonebacteria	D2-FULL-39-29	D2-FULL-39-29	D2-FULL-39-29	D2-FULL-39-29
ASV2119	Non-glacial	LacADNRUFtfa	0	Bacteria	Firestonebacteria	D2-FULL-39-29	D2-FULL-39-29	D2-FULL-39-29	D2-FULL-39-29
ASV2119	Non-glacial	LacADNRUFtfb	0	Bacteria	Firestonebacteria	D2-FULL-39-29	D2-FULL-39-29	D2-FULL-39-29	D2-FULL-39-29

ASV2119	Non-glacial	LacADNRUFtfc	0	Bacteria	Firestonebacteria	D2-FULL-39-29	D2-FULL-39-29	D2-FULL-39-29	D2-FULL-39-29
ASV2119	Glacial	LacAmpRUFtf-a	0	Bacteria	Firestonebacteria	D2-FULL-39-29	D2-FULL-39-29	D2-FULL-39-29	D2-FULL-39-29
ASV2119	Glacial	LacAmpRUFtf-b	0	Bacteria	Firestonebacteria	D2-FULL-39-29	D2-FULL-39-29	D2-FULL-39-29	D2-FULL-39-29
ASV2119	Glacial	LacAmpRUFtf-c	0	Bacteria	Firestonebacteria	D2-FULL-39-29	D2-FULL-39-29	D2-FULL-39-29	D2-FULL-39-29
ASV2130	Baie de la Table	BdT0-2	0	Bacteria	Proteobacteria	Gammaproteobacteria	Pseudomonadales	Hahellaceae	Oleiphilus
ASV2130	Control	Ctr-tf-IIb	0	Bacteria	Proteobacteria	Gammaproteobacteria	Pseudomonadales	Hahellaceae	Oleiphilus
ASV2130	Non-glacial	LacADNRUFtfa	0	Bacteria	Proteobacteria	Gammaproteobacteria	Pseudomonadales	Hahellaceae	Oleiphilus
ASV2130	Non-glacial	LacADNRUFtfb	0	Bacteria	Proteobacteria	Gammaproteobacteria	Pseudomonadales	Hahellaceae	Oleiphilus
ASV2130	Non-glacial	LacADNRUFtfc	0	Bacteria	Proteobacteria	Gammaproteobacteria	Pseudomonadales	Hahellaceae	Oleiphilus
ASV2130	Glacial	LacAmpRUFtf-a	0	Bacteria	Proteobacteria	Gammaproteobacteria	Pseudomonadales	Hahellaceae	Oleiphilus
ASV2130	Glacial	LacAmpRUFtf-b	0	Bacteria	Proteobacteria	Gammaproteobacteria	Pseudomonadales	Hahellaceae	Oleiphilus
ASV2130	Glacial	LacAmpRUFtf-c	0	Bacteria	Proteobacteria	Gammaproteobacteria	Pseudomonadales	Hahellaceae	Oleiphilus
ASV2136	Baie de la Table	BdT0-2	0	Bacteria	Proteobacteria	Alphaproteobacteria	Rhodospirillales	Rhodospirillaceae	GCA-2737725
ASV2136	Control	Ctr-tf-IIb	0	Bacteria	Proteobacteria	Alphaproteobacteria	Rhodospirillales	Rhodospirillaceae	GCA-2737725
ASV2136	Non-glacial	LacADNRUFtfa	0	Bacteria	Proteobacteria	Alphaproteobacteria	Rhodospirillales	Rhodospirillaceae	GCA-2737725
ASV2136	Non-glacial	LacADNRUFtfb	0	Bacteria	Proteobacteria	Alphaproteobacteria	Rhodospirillales	Rhodospirillaceae	GCA-2737725
ASV2136	Non-glacial	LacADNRUFtfc	0	Bacteria	Proteobacteria	Alphaproteobacteria	Rhodospirillales	Rhodospirillaceae	GCA-2737725
ASV2136	Glacial	LacAmpRUFtf-a	0	Bacteria	Proteobacteria	Alphaproteobacteria	Rhodospirillales	Rhodospirillaceae	GCA-2737725
ASV2136	Glacial	LacAmpRUFtf-b	0	Bacteria	Proteobacteria	Alphaproteobacteria	Rhodospirillales	Rhodospirillaceae	GCA-2737725
ASV2136	Glacial	LacAmpRUFtf-c	0	Bacteria	Proteobacteria	Alphaproteobacteria	Rhodospirillales	Rhodospirillaceae	GCA-2737725
ASV2149	Baie de la Table	BdT0-2	0	Bacteria	Proteobacteria	Alphaproteobacteria	Caulobacterales	Hyphomonadaceae	SWB02
ASV2149	Control	Ctr-tf-IIb	0	Bacteria	Proteobacteria	Alphaproteobacteria	Caulobacterales	Hyphomonadaceae	SWB02
ASV2149	Non-glacial	LacADNRUFtfa	0	Bacteria	Proteobacteria	Alphaproteobacteria	Caulobacterales	Hyphomonadaceae	SWB02
ASV2149	Non-glacial	LacADNRUFtfb	0	Bacteria	Proteobacteria	Alphaproteobacteria	Caulobacterales	Hyphomonadaceae	SWB02
ASV2149	Non-glacial	LacADNRUFtfc	0	Bacteria	Proteobacteria	Alphaproteobacteria	Caulobacterales	Hyphomonadaceae	SWB02
ASV2149	Glacial	LacAmpRUFtf-a	0	Bacteria	Proteobacteria	Alphaproteobacteria	Caulobacterales	Hyphomonadaceae	SWB02
ASV2149	Glacial	LacAmpRUFtf-b	0	Bacteria	Proteobacteria	Alphaproteobacteria	Caulobacterales	Hyphomonadaceae	SWB02
ASV2149	Glacial	LacAmpRUFtf-c	0	Bacteria	Proteobacteria	Alphaproteobacteria	Caulobacterales	Hyphomonadaceae	SWB02
ASV2156	Baie de la Table	BdT0-2	0	Bacteria	Cyanobacteriota	Cyanobacteriia	Leptolyngbyales	Leptolyngbyaceae	JSC-12
ASV2156	Control	Ctr-tf-IIb	0	Bacteria	Cyanobacteriota	Cyanobacteriia	Leptolyngbyales	Leptolyngbyaceae	JSC-12
ASV2156	Non-glacial	LacADNRUFtfa	0	Bacteria	Cyanobacteriota	Cyanobacteriia	Leptolyngbyales	Leptolyngbyaceae	JSC-12
ASV2156	Non-glacial	LacADNRUFtfb	0	Bacteria	Cyanobacteriota	Cyanobacteriia	Leptolyngbyales	Leptolyngbyaceae	JSC-12
ASV2156	Non-glacial	LacADNRUFtfc	0	Bacteria	Cyanobacteriota	Cyanobacteriia	Leptolyngbyales	Leptolyngbyaceae	JSC-12
ASV2156	Glacial	LacAmpRUFtf-a	0	Bacteria	Cyanobacteriota	Cyanobacteriia	Leptolyngbyales	Leptolyngbyaceae	JSC-12

ASV2156	Glacial	LacAmpRUFtf-b	0	Bacteria	Cyanobacteriota	Cyanobacteriia	Leptolyngbyales	Leptolyngbyaceae	JSC-12
ASV2156	Glacial	LacAmpRUFtf-c	0	Bacteria	Cyanobacteriota	Cyanobacteriia	Leptolyngbyales	Leptolyngbyaceae	JSC-12
ASV2159	Baie de la Table	BdT0-2	0	Bacteria	Chloroflexota	Anaerolineae	SBR1031	A4b	GCA-2699585
ASV2159	Control	Ctr-tf-IIb	0	Bacteria	Chloroflexota	Anaerolineae	SBR1031	A4b	GCA-2699585
ASV2159	Non-glacial	LacADNRUFtfa	0	Bacteria	Chloroflexota	Anaerolineae	SBR1031	A4b	GCA-2699585
ASV2159	Non-glacial	LacADNRUFtfb	0	Bacteria	Chloroflexota	Anaerolineae	SBR1031	A4b	GCA-2699585
ASV2159	Non-glacial	LacADNRUFtfc	0	Bacteria	Chloroflexota	Anaerolineae	SBR1031	A4b	GCA-2699585
ASV2159	Glacial	LacAmpRUFtf-a	0	Bacteria	Chloroflexota	Anaerolineae	SBR1031	A4b	GCA-2699585
ASV2159	Glacial	LacAmpRUFtf-b	0	Bacteria	Chloroflexota	Anaerolineae	SBR1031	A4b	GCA-2699585
ASV2159	Glacial	LacAmpRUFtf-c	0	Bacteria	Chloroflexota	Anaerolineae	SBR1031	A4b	GCA-2699585
ASV2162	Baie de la Table	BdT0-2	0	Bacteria	Bacteroidota	Bacteroidia	Bacteroidales	Lentimicrobiaceae	Lentimicrobium
ASV2162	Control	Ctr-tf-IIb	0	Bacteria	Bacteroidota	Bacteroidia	Bacteroidales	Lentimicrobiaceae	Lentimicrobium
ASV2162	Non-glacial	LacADNRUFtfa	0	Bacteria	Bacteroidota	Bacteroidia	Bacteroidales	Lentimicrobiaceae	Lentimicrobium
ASV2162	Non-glacial	LacADNRUFtfb	0	Bacteria	Bacteroidota	Bacteroidia	Bacteroidales	Lentimicrobiaceae	Lentimicrobium
ASV2162	Non-glacial	LacADNRUFtfc	0	Bacteria	Bacteroidota	Bacteroidia	Bacteroidales	Lentimicrobiaceae	Lentimicrobium
ASV2162	Glacial	LacAmpRUFtf-a	0	Bacteria	Bacteroidota	Bacteroidia	Bacteroidales	Lentimicrobiaceae	Lentimicrobium
ASV2162	Glacial	LacAmpRUFtf-b	0	Bacteria	Bacteroidota	Bacteroidia	Bacteroidales	Lentimicrobiaceae	Lentimicrobium
ASV2162	Glacial	LacAmpRUFtf-c	0	Bacteria	Bacteroidota	Bacteroidia	Bacteroidales	Lentimicrobiaceae	Lentimicrobium
ASV218	Baie de la Table	BdT0-2	0,000382555	Bacteria	Bacteroidota	Bacteroidia	Flavobacteriales	UA16	UBA974
ASV218	Glacial	LacAmpRUFtf-a	0,000361678	Bacteria	Bacteroidota	Bacteroidia	Flavobacteriales	UA16	UBA974
ASV218	Control	Ctr-tf-IIb	0,000292481	Bacteria	Bacteroidota	Bacteroidia	Flavobacteriales	UA16	UBA974
ASV218	Non-glacial	LacADNRUFtfa	0	Bacteria	Bacteroidota	Bacteroidia	Flavobacteriales	UA16	UBA974
ASV218	Non-glacial	LacADNRUFtfb	0	Bacteria	Bacteroidota	Bacteroidia	Flavobacteriales	UA16	UBA974
ASV218	Non-glacial	LacADNRUFtfc	0	Bacteria	Bacteroidota	Bacteroidia	Flavobacteriales	UA16	UBA974
ASV218	Glacial	LacAmpRUFtf-b	0	Bacteria	Bacteroidota	Bacteroidia	Flavobacteriales	UA16	UBA974
ASV218	Glacial	LacAmpRUFtf-c	0	Bacteria	Bacteroidota	Bacteroidia	Flavobacteriales	UA16	UBA974
ASV2188	Baie de la Table	BdT0-2	0	Bacteria	Actinobacteriota	Actinobacteria	Actinomycetales	Dermatophilaceae	Kytococcus
ASV2188	Control	Ctr-tf-IIb	0	Bacteria	Actinobacteriota	Actinobacteria	Actinomycetales	Dermatophilaceae	Kytococcus
ASV2188	Non-glacial	LacADNRUFtfa	0	Bacteria	Actinobacteriota	Actinobacteria	Actinomycetales	Dermatophilaceae	Kytococcus
ASV2188	Non-glacial	LacADNRUFtfb	0	Bacteria	Actinobacteriota	Actinobacteria	Actinomycetales	Dermatophilaceae	Kytococcus
ASV2188	Non-glacial	LacADNRUFtfc	0	Bacteria	Actinobacteriota	Actinobacteria	Actinomycetales	Dermatophilaceae	Kytococcus
ASV2188	Glacial	LacAmpRUFtf-a	0	Bacteria	Actinobacteriota	Actinobacteria	Actinomycetales	Dermatophilaceae	Kytococcus
ASV2188	Glacial	LacAmpRUFtf-b	0	Bacteria	Actinobacteriota	Actinobacteria	Actinomycetales	Dermatophilaceae	Kytococcus
ASV2188	Glacial	LacAmpRUFtf-c	0	Bacteria	Actinobacteriota	Actinobacteria	Actinomycetales	Dermatophilaceae	Kytococcus

ASV219	Non-glacial	LacADNRUFtfb	0,000126449	Bacteria	Proteobacteria	Alphaproteobacteria	Rhizobiales	Rhizobiaceae	Leaf454
ASV219	Baie de la Table	BdT0-2	0	Bacteria	Proteobacteria	Alphaproteobacteria	Rhizobiales	Rhizobiaceae	Leaf454
ASV219	Control	Ctr-tf-IIb	0	Bacteria	Proteobacteria	Alphaproteobacteria	Rhizobiales	Rhizobiaceae	Leaf454
ASV219	Non-glacial	LacADNRUFtfa	0	Bacteria	Proteobacteria	Alphaproteobacteria	Rhizobiales	Rhizobiaceae	Leaf454
ASV219	Non-glacial	LacADNRUFtfc	0	Bacteria	Proteobacteria	Alphaproteobacteria	Rhizobiales	Rhizobiaceae	Leaf454
ASV219	Glacial	LacAmpRUFtf-a	0	Bacteria	Proteobacteria	Alphaproteobacteria	Rhizobiales	Rhizobiaceae	Leaf454
ASV219	Glacial	LacAmpRUFtf-b	0	Bacteria	Proteobacteria	Alphaproteobacteria	Rhizobiales	Rhizobiaceae	Leaf454
ASV219	Glacial	LacAmpRUFtf-c	0	Bacteria	Proteobacteria	Alphaproteobacteria	Rhizobiales	Rhizobiaceae	Leaf454
ASV2191	Baie de la Table	BdT0-2	0	Bacteria	Actinobacteriota	Actinobacteria	Actinomycetales	Microbacteriaceae	Rhodoluna
ASV2191	Control	Ctr-tf-IIb	0	Bacteria	Actinobacteriota	Actinobacteria	Actinomycetales	Microbacteriaceae	Rhodoluna
ASV2191	Non-glacial	LacADNRUFtfa	0	Bacteria	Actinobacteriota	Actinobacteria	Actinomycetales	Microbacteriaceae	Rhodoluna
ASV2191	Non-glacial	LacADNRUFtfb	0	Bacteria	Actinobacteriota	Actinobacteria	Actinomycetales	Microbacteriaceae	Rhodoluna
ASV2191	Non-glacial	LacADNRUFtfc	0	Bacteria	Actinobacteriota	Actinobacteria	Actinomycetales	Microbacteriaceae	Rhodoluna
ASV2191	Glacial	LacAmpRUFtf-a	0	Bacteria	Actinobacteriota	Actinobacteria	Actinomycetales	Microbacteriaceae	Rhodoluna
ASV2191	Glacial	LacAmpRUFtf-b	0	Bacteria	Actinobacteriota	Actinobacteria	Actinomycetales	Microbacteriaceae	Rhodoluna
ASV2191	Glacial	LacAmpRUFtf-c	0	Bacteria	Actinobacteriota	Actinobacteria	Actinomycetales	Microbacteriaceae	Rhodoluna
ASV2198	Baie de la Table	BdT0-2	0	Bacteria	Proteobacteria	Gammaproteobacteria	Enterobacterales	Psychromonadaceae	Moritella
ASV2198	Control	Ctr-tf-IIb	0	Bacteria	Proteobacteria	Gammaproteobacteria	Enterobacterales	Psychromonadaceae	Moritella
ASV2198	Non-glacial	LacADNRUFtfa	0	Bacteria	Proteobacteria	Gammaproteobacteria	Enterobacterales	Psychromonadaceae	Moritella
ASV2198	Non-glacial	LacADNRUFtfb	0	Bacteria	Proteobacteria	Gammaproteobacteria	Enterobacterales	Psychromonadaceae	Moritella
ASV2198	Non-glacial	LacADNRUFtfc	0	Bacteria	Proteobacteria	Gammaproteobacteria	Enterobacterales	Psychromonadaceae	Moritella
ASV2198	Glacial	LacAmpRUFtf-a	0	Bacteria	Proteobacteria	Gammaproteobacteria	Enterobacterales	Psychromonadaceae	Moritella
ASV2198	Glacial	LacAmpRUFtf-b	0	Bacteria	Proteobacteria	Gammaproteobacteria	Enterobacterales	Psychromonadaceae	Moritella
ASV2198	Glacial	LacAmpRUFtf-c	0	Bacteria	Proteobacteria	Gammaproteobacteria	Enterobacterales	Psychromonadaceae	Moritella
ASV22	Control	Ctr-tf-IIb	0,022252583	Bacteria	Bacteroidota	Bacteroidia	NS11-12g	UBA9320	UBA9320
ASV22	Baie de la Table	BdT0-2	0,008470871	Bacteria	Bacteroidota	Bacteroidia	NS11-12g	UBA9320	UBA9320
ASV22	Non-glacial	LacADNRUFtfb	0,004573235	Bacteria	Bacteroidota	Bacteroidia	NS11-12g	UBA9320	UBA9320
ASV22	Non-glacial	LacADNRUFtfc	0,003734239	Bacteria	Bacteroidota	Bacteroidia	NS11-12g	UBA9320	UBA9320
ASV22	Non-glacial	LacADNRUFtfa	0,003094831	Bacteria	Bacteroidota	Bacteroidia	NS11-12g	UBA9320	UBA9320
ASV22	Glacial	LacAmpRUFtf-b	0,003023432	Bacteria	Bacteroidota	Bacteroidia	NS11-12g	UBA9320	UBA9320
ASV22	Glacial	LacAmpRUFtf-a	0,002250442	Bacteria	Bacteroidota	Bacteroidia	NS11-12g	UBA9320	UBA9320
ASV22	Glacial	LacAmpRUFtf-c	0,001742919	Bacteria	Bacteroidota	Bacteroidia	NS11-12g	UBA9320	UBA9320
ASV222	Non-glacial	LacADNRUFtfa	0,014088411	Bacteria	Proteobacteria	Gammaproteobacteria	Nitrosococcales	Methylophagaceae	41-12-T18
ASV222	Non-glacial	LacADNRUFtfb	0,000210748	Bacteria	Proteobacteria	Gammaproteobacteria	Nitrosococcales	Methylophagaceae	41-12-T18

ASV222	Control	Ctr-tf-IIb	0,000112184	Bacteria	Proteobacteria	Gammaproteobacteria	Nitrosococcales	Methylophagaceae	41-12-T18
ASV222	Baie de la Table	BdT0-2	0	Bacteria	Proteobacteria	Gammaproteobacteria	Nitrosococcales	Methylophagaceae	41-12-T18
ASV222	Non-glacial	LacADNRUFtfc	0	Bacteria	Proteobacteria	Gammaproteobacteria	Nitrosococcales	Methylophagaceae	41-12-T18
ASV222	Glacial	LacAmpRUFtf-a	0	Bacteria	Proteobacteria	Gammaproteobacteria	Nitrosococcales	Methylophagaceae	41-12-T18
ASV222	Glacial	LacAmpRUFtf-b	0	Bacteria	Proteobacteria	Gammaproteobacteria	Nitrosococcales	Methylophagaceae	41-12-T18
ASV222	Glacial	LacAmpRUFtf-c	0	Bacteria	Proteobacteria	Gammaproteobacteria	Nitrosococcales	Methylophagaceae	41-12-T18
ASV223(	Baie de la Table	BdT0-2	0	Bacteria	Gemmatimonad	Gemmatimonadetes	Gemmatimonadales	Gemmatimonadaceae	Gemmatirosa
ASV223(	Control	Ctr-tf-IIb	0	Bacteria	Gemmatimonad	Gemmatimonadetes	Gemmatimonadales	Gemmatimonadaceae	Gemmatirosa
ASV223(	Non-glacial	LacADNRUFtfa	0	Bacteria	Gemmatimonad	Gemmatimonadetes	Gemmatimonadales	Gemmatimonadaceae	Gemmatirosa
ASV223(	Non-glacial	LacADNRUFtfb	0	Bacteria	Gemmatimonad	Gemmatimonadetes	Gemmatimonadales	Gemmatimonadaceae	Gemmatirosa
ASV223(	Non-glacial	LacADNRUFtfc	0	Bacteria	Gemmatimonad	Gemmatimonadetes	Gemmatimonadales	Gemmatimonadaceae	Gemmatirosa
ASV223(	Glacial	LacAmpRUFtf-a	0	Bacteria	Gemmatimonad	Gemmatimonadetes	Gemmatimonadales	Gemmatimonadaceae	Gemmatirosa
ASV223(	Glacial	LacAmpRUFtf-b	0	Bacteria	Gemmatimonad	Gemmatimonadetes	Gemmatimonadales	Gemmatimonadaceae	Gemmatirosa
ASV223(	Glacial	LacAmpRUFtf-c	0	Bacteria	Gemmatimonad	Gemmatimonadetes	Gemmatimonadales	Gemmatimonadaceae	Gemmatirosa
ASV229	Baie de la Table	BdT0-2	0	Bacteria	Firmicutes	Bacilli_A	Paenibacillales	Paenibacillaceae	Paenibacillus_I
ASV229	Control	Ctr-tf-IIb	0	Bacteria	Firmicutes	Bacilli_A	Paenibacillales	Paenibacillaceae	Paenibacillus_I
ASV229	Non-glacial	LacADNRUFtfa	0	Bacteria	Firmicutes	Bacilli_A	Paenibacillales	Paenibacillaceae	Paenibacillus_I
ASV229	Non-glacial	LacADNRUFtfb	0	Bacteria	Firmicutes	Bacilli_A	Paenibacillales	Paenibacillaceae	Paenibacillus_I
ASV229	Non-glacial	LacADNRUFtfc	0	Bacteria	Firmicutes	Bacilli_A	Paenibacillales	Paenibacillaceae	Paenibacillus_I
ASV229	Glacial	LacAmpRUFtf-a	0	Bacteria	Firmicutes	Bacilli_A	Paenibacillales	Paenibacillaceae	Paenibacillus_I
ASV229	Glacial	LacAmpRUFtf-b	0	Bacteria	Firmicutes	Bacilli_A	Paenibacillales	Paenibacillaceae	Paenibacillus_I
ASV229	Glacial	LacAmpRUFtf-c	0	Bacteria	Firmicutes	Bacilli_A	Paenibacillales	Paenibacillaceae	Paenibacillus_I
ASV237	Control	Ctr-tf-IIb	0,000240395	Bacteria	Bacteroidota	Bacteroidia	Cytophagales	Cyclobacteriaceae	Fabibacter
ASV237	Non-glacial	LacADNRUFtfc	4,85E-05	Bacteria	Bacteroidota	Bacteroidia	Cytophagales	Cyclobacteriaceae	Fabibacter
ASV237	Baie de la Table	BdT0-2	0	Bacteria	Bacteroidota	Bacteroidia	Cytophagales	Cyclobacteriaceae	Fabibacter
ASV237	Non-glacial	LacADNRUFtfa	0	Bacteria	Bacteroidota	Bacteroidia	Cytophagales	Cyclobacteriaceae	Fabibacter
ASV237	Non-glacial	LacADNRUFtfb	0	Bacteria	Bacteroidota	Bacteroidia	Cytophagales	Cyclobacteriaceae	Fabibacter
ASV237	Glacial	LacAmpRUFtf-a	0	Bacteria	Bacteroidota	Bacteroidia	Cytophagales	Cyclobacteriaceae	Fabibacter
ASV237	Glacial	LacAmpRUFtf-b	0	Bacteria	Bacteroidota	Bacteroidia	Cytophagales	Cyclobacteriaceae	Fabibacter
ASV237	Glacial	LacAmpRUFtf-c	0	Bacteria	Bacteroidota	Bacteroidia	Cytophagales	Cyclobacteriaceae	Fabibacter
ASV239	Baie de la Table	BdT0-2	0	Bacteria	Bacteroidota	Kapabacteria	Kapabacteriales	Kapabacteriaceae	Kapabacteria
ASV239	Control	Ctr-tf-IIb	0	Bacteria	Bacteroidota	Kapabacteria	Kapabacteriales	Kapabacteriaceae	Kapabacteria
ASV239	Non-glacial	LacADNRUFtfa	0	Bacteria	Bacteroidota	Kapabacteria	Kapabacteriales	Kapabacteriaceae	Kapabacteria
ASV239	Non-glacial	LacADNRUFtfb	0	Bacteria	Bacteroidota	Kapabacteria	Kapabacteriales	Kapabacteriaceae	Kapabacteria

ASV239	Non-glacial	LacADNRUFtfc	0	Bacteria	Bacteroidota	Kapabacteria	Kapabacteriales	Kapabacteriaceae	Kapabacteria
ASV239	Glacial	LacAmpRUFtf-a	0	Bacteria	Bacteroidota	Kapabacteria	Kapabacteriales	Kapabacteriaceae	Kapabacteria
ASV239	Glacial	LacAmpRUFtf-b	0	Bacteria	Bacteroidota	Kapabacteria	Kapabacteriales	Kapabacteriaceae	Kapabacteria
ASV239	Glacial	LacAmpRUFtf-c	0	Bacteria	Bacteroidota	Kapabacteria	Kapabacteriales	Kapabacteriaceae	Kapabacteria
ASV25	Non-glacial	LacADNRUFtfb	0,000969442	Bacteria	Actinobacteriota	Actinobacteria	Nanopelagicales	Nanopelagicaceae	Planktophila
ASV25	Non-glacial	LacADNRUFtfc	0,000484966	Bacteria	Actinobacteriota	Actinobacteria	Nanopelagicales	Nanopelagicaceae	Planktophila
ASV25	Non-glacial	LacADNRUFtfa	0,000277149	Bacteria	Actinobacteriota	Actinobacteria	Nanopelagicales	Nanopelagicaceae	Planktophila
ASV25	Baie de la Table	BdT0-2	0	Bacteria	Actinobacteriota	Actinobacteria	Nanopelagicales	Nanopelagicaceae	Planktophila
ASV25	Control	Ctr-tf-IIb	0	Bacteria	Actinobacteriota	Actinobacteria	Nanopelagicales	Nanopelagicaceae	Planktophila
ASV25	Glacial	LacAmpRUFtf-a	0	Bacteria	Actinobacteriota	Actinobacteria	Nanopelagicales	Nanopelagicaceae	Planktophila
ASV25	Glacial	LacAmpRUFtf-b	0	Bacteria	Actinobacteriota	Actinobacteria	Nanopelagicales	Nanopelagicaceae	Planktophila
ASV25	Glacial	LacAmpRUFtf-c	0	Bacteria	Actinobacteriota	Actinobacteria	Nanopelagicales	Nanopelagicaceae	Planktophila
ASV251	Non-glacial	LacADNRUFtfb	0,001243414	Bacteria	Proteobacteria	Alphaproteobacteria	Defluviicoccales	Defluviicoccaceae	Defluviicoccus
ASV251	Non-glacial	LacADNRUFtfa	0,001062405	Bacteria	Proteobacteria	Alphaproteobacteria	Defluviicoccales	Defluviicoccaceae	Defluviicoccus
ASV251	Glacial	LacAmpRUFtf-a	0,000120559	Bacteria	Proteobacteria	Alphaproteobacteria	Defluviicoccales	Defluviicoccaceae	Defluviicoccus
ASV251	Control	Ctr-tf-IIb	7,61E-05	Bacteria	Proteobacteria	Alphaproteobacteria	Defluviicoccales	Defluviicoccaceae	Defluviicoccus
ASV251	Baie de la Table	BdT0-2	0	Bacteria	Proteobacteria	Alphaproteobacteria	Defluviicoccales	Defluviicoccaceae	Defluviicoccus
ASV251	Non-glacial	LacADNRUFtfc	0	Bacteria	Proteobacteria	Alphaproteobacteria	Defluviicoccales	Defluviicoccaceae	Defluviicoccus
ASV251	Glacial	LacAmpRUFtf-b	0	Bacteria	Proteobacteria	Alphaproteobacteria	Defluviicoccales	Defluviicoccaceae	Defluviicoccus
ASV251	Glacial	LacAmpRUFtf-c	0	Bacteria	Proteobacteria	Alphaproteobacteria	Defluviicoccales	Defluviicoccaceae	Defluviicoccus
ASV256	Baie de la Table	BdT0-2	0	Bacteria	Bacteroidota	Bacteroidia	Cytophagales	Cyclobacteriaceae	Algoriphagus
ASV256	Control	Ctr-tf-IIb	0	Bacteria	Bacteroidota	Bacteroidia	Cytophagales	Cyclobacteriaceae	Algoriphagus
ASV256	Non-glacial	LacADNRUFtfa	0	Bacteria	Bacteroidota	Bacteroidia	Cytophagales	Cyclobacteriaceae	Algoriphagus
ASV256	Non-glacial	LacADNRUFtfb	0	Bacteria	Bacteroidota	Bacteroidia	Cytophagales	Cyclobacteriaceae	Algoriphagus
ASV256	Non-glacial	LacADNRUFtfc	0	Bacteria	Bacteroidota	Bacteroidia	Cytophagales	Cyclobacteriaceae	Algoriphagus
ASV256	Glacial	LacAmpRUFtf-a	0	Bacteria	Bacteroidota	Bacteroidia	Cytophagales	Cyclobacteriaceae	Algoriphagus
ASV256	Glacial	LacAmpRUFtf-b	0	Bacteria	Bacteroidota	Bacteroidia	Cytophagales	Cyclobacteriaceae	Algoriphagus
ASV256	Glacial	LacAmpRUFtf-c	0	Bacteria	Bacteroidota	Bacteroidia	Cytophagales	Cyclobacteriaceae	Algoriphagus
ASV258	Glacial	LacAmpRUFtf-a	0,007635428	Bacteria	Proteobacteria	Gammaproteobacteria	Pseudomonadales	Nitrincolaceae	Marinobacterium
ASV258	Glacial	LacAmpRUFtf-b	0,002051614	Bacteria	Proteobacteria	Gammaproteobacteria	Pseudomonadales	Nitrincolaceae	Marinobacterium
ASV258	Baie de la Table	BdT0-2	0	Bacteria	Proteobacteria	Gammaproteobacteria	Pseudomonadales	Nitrincolaceae	Marinobacterium
ASV258	Control	Ctr-tf-IIb	0	Bacteria	Proteobacteria	Gammaproteobacteria	Pseudomonadales	Nitrincolaceae	Marinobacterium
ASV258	Non-glacial	LacADNRUFtfa	0	Bacteria	Proteobacteria	Gammaproteobacteria	Pseudomonadales	Nitrincolaceae	Marinobacterium
ASV258	Non-glacial	LacADNRUFtfb	0	Bacteria	Proteobacteria	Gammaproteobacteria	Pseudomonadales	Nitrincolaceae	Marinobacterium

ASV258	Non-glacial	LacADNRUFtfc	0	Bacteria	Proteobacteria	Gammaproteobacteria	Pseudomonadales	Nitrincolaceae	Marinobacterium
ASV258	Glacial	LacAmpRUFtf-c	0	Bacteria	Proteobacteria	Gammaproteobacteria	Pseudomonadales	Nitrincolaceae	Marinobacterium
ASV26	Non-glacial	LacADNRUFtfc	0,248254122	Bacteria	Bacteroidota	Bacteroidia	Flavobacteriales	Flavobacteriaceae	Flavobacterium
ASV26	Non-glacial	LacADNRUFtfb	0,208261328	Bacteria	Bacteroidota	Bacteroidia	Flavobacteriales	Flavobacteriaceae	Flavobacterium
ASV26	Non-glacial	LacADNRUFtfa	0,165042265	Bacteria	Bacteroidota	Bacteroidia	Flavobacteriales	Flavobacteriaceae	Flavobacterium
ASV26	Glacial	LacAmpRUFtf-b	0,024403412	Bacteria	Bacteroidota	Bacteroidia	Flavobacteriales	Flavobacteriaceae	Flavobacterium
ASV26	Glacial	LacAmpRUFtf-c	0,001936577	Bacteria	Bacteroidota	Bacteroidia	Flavobacteriales	Flavobacteriaceae	Flavobacterium
ASV26	Control	Ctr-tf-IIb	0,001346213	Bacteria	Bacteroidota	Bacteroidia	Flavobacteriales	Flavobacteriaceae	Flavobacterium
ASV26	Glacial	LacAmpRUFtf-a	0,000602797	Bacteria	Bacteroidota	Bacteroidia	Flavobacteriales	Flavobacteriaceae	Flavobacterium
ASV26	Baie de la Table	BdT0-2	0	Bacteria	Bacteroidota	Bacteroidia	Flavobacteriales	Flavobacteriaceae	Flavobacterium
ASV262	Baie de la Table	BdT0-2	0	Bacteria	Proteobacteria	Alphaproteobacteria	Caulobacterales	Caulobacteraceae	Phenylobacterium
ASV262	Control	Ctr-tf-IIb	0	Bacteria	Proteobacteria	Alphaproteobacteria	Caulobacterales	Caulobacteraceae	Phenylobacterium
ASV262	Non-glacial	LacADNRUFtfa	0	Bacteria	Proteobacteria	Alphaproteobacteria	Caulobacterales	Caulobacteraceae	Phenylobacterium
ASV262	Non-glacial	LacADNRUFtfb	0	Bacteria	Proteobacteria	Alphaproteobacteria	Caulobacterales	Caulobacteraceae	Phenylobacterium
ASV262	Non-glacial	LacADNRUFtfc	0	Bacteria	Proteobacteria	Alphaproteobacteria	Caulobacterales	Caulobacteraceae	Phenylobacterium
ASV262	Glacial	LacAmpRUFtf-a	0	Bacteria	Proteobacteria	Alphaproteobacteria	Caulobacterales	Caulobacteraceae	Phenylobacterium
ASV262	Glacial	LacAmpRUFtf-b	0	Bacteria	Proteobacteria	Alphaproteobacteria	Caulobacterales	Caulobacteraceae	Phenylobacterium
ASV262	Glacial	LacAmpRUFtf-c	0	Bacteria	Proteobacteria	Alphaproteobacteria	Caulobacterales	Caulobacteraceae	Phenylobacterium
ASV263	Baie de la Table	BdT0-2	0	Bacteria	Proteobacteria	Alphaproteobacteria	Rhizobiales	Beijerinckiaceae	Methyloferula
ASV263	Control	Ctr-tf-IIb	0	Bacteria	Proteobacteria	Alphaproteobacteria	Rhizobiales	Beijerinckiaceae	Methyloferula
ASV263	Non-glacial	LacADNRUFtfa	0	Bacteria	Proteobacteria	Alphaproteobacteria	Rhizobiales	Beijerinckiaceae	Methyloferula
ASV263	Non-glacial	LacADNRUFtfb	0	Bacteria	Proteobacteria	Alphaproteobacteria	Rhizobiales	Beijerinckiaceae	Methyloferula
ASV263	Non-glacial	LacADNRUFtfc	0	Bacteria	Proteobacteria	Alphaproteobacteria	Rhizobiales	Beijerinckiaceae	Methyloferula
ASV263	Glacial	LacAmpRUFtf-a	0	Bacteria	Proteobacteria	Alphaproteobacteria	Rhizobiales	Beijerinckiaceae	Methyloferula
ASV263	Glacial	LacAmpRUFtf-b	0	Bacteria	Proteobacteria	Alphaproteobacteria	Rhizobiales	Beijerinckiaceae	Methyloferula
ASV263	Glacial	LacAmpRUFtf-c	0	Bacteria	Proteobacteria	Alphaproteobacteria	Rhizobiales	Beijerinckiaceae	Methyloferula
ASV264	Glacial	LacAmpRUFtf-c	0,023965142	Bacteria	Actinobacteriota	Actinobacteria	Corynebacteriales	Corynebacteriaceae	Corynebacterium
ASV264	Control	Ctr-tf-IIb	0,001105818	Bacteria	Actinobacteriota	Actinobacteria	Corynebacteriales	Corynebacteriaceae	Corynebacterium
ASV264	Glacial	LacAmpRUFtf-b	0,000863838	Bacteria	Actinobacteriota	Actinobacteria	Corynebacteriales	Corynebacteriaceae	Corynebacterium
ASV264	Non-glacial	LacADNRUFtfb	0,00061117	Bacteria	Actinobacteriota	Actinobacteria	Corynebacteriales	Corynebacteriaceae	Corynebacterium
ASV264	Glacial	LacAmpRUFtf-a	0,000160746	Bacteria	Actinobacteriota	Actinobacteria	Corynebacteriales	Corynebacteriaceae	Corynebacterium
ASV264	Non-glacial	LacADNRUFtfc	4,85E-05	Bacteria	Actinobacteriota	Actinobacteria	Corynebacteriales	Corynebacteriaceae	Corynebacterium
ASV264	Baie de la Table	BdT0-2	0	Bacteria	Actinobacteriota	Actinobacteria	Corynebacteriales	Corynebacteriaceae	Corynebacterium
ASV264	Non-glacial	LacADNRUFtfa	0	Bacteria	Actinobacteriota	Actinobacteria	Corynebacteriales	Corynebacteriaceae	Corynebacterium

ASV265	Glacial	LacAmpRUFtf-a	0,004099019	Bacteria	Proteobacteria	Alphaproteobacteria	Rhodobacterales	Rhodobacteraceae	Jannaschia
ASV265	Baie de la Table	BdT0-2	0	Bacteria	Proteobacteria	Alphaproteobacteria	Rhodobacterales	Rhodobacteraceae	Jannaschia
ASV265	Control	Ctr-tf-IIb	0	Bacteria	Proteobacteria	Alphaproteobacteria	Rhodobacterales	Rhodobacteraceae	Jannaschia
ASV265	Non-glacial	LacADNRUFtfa	0	Bacteria	Proteobacteria	Alphaproteobacteria	Rhodobacterales	Rhodobacteraceae	Jannaschia
ASV265	Non-glacial	LacADNRUFtfb	0	Bacteria	Proteobacteria	Alphaproteobacteria	Rhodobacterales	Rhodobacteraceae	Jannaschia
ASV265	Non-glacial	LacADNRUFtfc	0	Bacteria	Proteobacteria	Alphaproteobacteria	Rhodobacterales	Rhodobacteraceae	Jannaschia
ASV265	Glacial	LacAmpRUFtf-b	0	Bacteria	Proteobacteria	Alphaproteobacteria	Rhodobacterales	Rhodobacteraceae	Jannaschia
ASV265	Glacial	LacAmpRUFtf-c	0	Bacteria	Proteobacteria	Alphaproteobacteria	Rhodobacterales	Rhodobacteraceae	Jannaschia
ASV27	Baie de la Table	BdT0-2	0	Bacteria	Proteobacteria	Gammaproteobacteria	Betaproteobacteriales	Burkholderiaceae	Polynucleobacter
ASV27	Control	Ctr-tf-IIb	0	Bacteria	Proteobacteria	Gammaproteobacteria	Betaproteobacteriales	Burkholderiaceae	Polynucleobacter
ASV27	Non-glacial	LacADNRUFtfa	0	Bacteria	Proteobacteria	Gammaproteobacteria	Betaproteobacteriales	Burkholderiaceae	Polynucleobacter
ASV27	Non-glacial	LacADNRUFtfb	0	Bacteria	Proteobacteria	Gammaproteobacteria	Betaproteobacteriales	Burkholderiaceae	Polynucleobacter
ASV27	Non-glacial	LacADNRUFtfc	0	Bacteria	Proteobacteria	Gammaproteobacteria	Betaproteobacteriales	Burkholderiaceae	Polynucleobacter
ASV27	Glacial	LacAmpRUFtf-a	0	Bacteria	Proteobacteria	Gammaproteobacteria	Betaproteobacteriales	Burkholderiaceae	Polynucleobacter
ASV27	Glacial	LacAmpRUFtf-b	0	Bacteria	Proteobacteria	Gammaproteobacteria	Betaproteobacteriales	Burkholderiaceae	Polynucleobacter
ASV27	Glacial	LacAmpRUFtf-c	0	Bacteria	Proteobacteria	Gammaproteobacteria	Betaproteobacteriales	Burkholderiaceae	Polynucleobacter
ASV270	Non-glacial	LacADNRUFtfa	0,004572959	Bacteria	Proteobacteria	Gammaproteobacteria	Betaproteobacteriales	Burkholderiaceae	Massilia_A
ASV270	Non-glacial	LacADNRUFtfb	0,004088514	Bacteria	Proteobacteria	Gammaproteobacteria	Betaproteobacteriales	Burkholderiaceae	Massilia_A
ASV270	Non-glacial	LacADNRUFtfc	0,001357905	Bacteria	Proteobacteria	Gammaproteobacteria	Betaproteobacteriales	Burkholderiaceae	Massilia_A
ASV270	Baie de la Table	BdT0-2	0	Bacteria	Proteobacteria	Gammaproteobacteria	Betaproteobacteriales	Burkholderiaceae	Massilia_A
ASV270	Control	Ctr-tf-IIb	0	Bacteria	Proteobacteria	Gammaproteobacteria	Betaproteobacteriales	Burkholderiaceae	Massilia_A
ASV270	Glacial	LacAmpRUFtf-a	0	Bacteria	Proteobacteria	Gammaproteobacteria	Betaproteobacteriales	Burkholderiaceae	Massilia_A
ASV270	Glacial	LacAmpRUFtf-b	0	Bacteria	Proteobacteria	Gammaproteobacteria	Betaproteobacteriales	Burkholderiaceae	Massilia_A
ASV270	Glacial	LacAmpRUFtf-c	0	Bacteria	Proteobacteria	Gammaproteobacteria	Betaproteobacteriales	Burkholderiaceae	Massilia_A
ASV273	Baie de la Table	BdT0-2	0	Bacteria	Actinobacteriota	Thermoleophilia	Solirubrobacterales	Solirubrobacteraceae	Conexibacter_A
ASV273	Control	Ctr-tf-IIb	0	Bacteria	Actinobacteriota	Thermoleophilia	Solirubrobacterales	Solirubrobacteraceae	Conexibacter_A
ASV273	Non-glacial	LacADNRUFtfa	0	Bacteria	Actinobacteriota	Thermoleophilia	Solirubrobacterales	Solirubrobacteraceae	Conexibacter_A
ASV273	Non-glacial	LacADNRUFtfb	0	Bacteria	Actinobacteriota	Thermoleophilia	Solirubrobacterales	Solirubrobacteraceae	Conexibacter_A
ASV273	Non-glacial	LacADNRUFtfc	0	Bacteria	Actinobacteriota	Thermoleophilia	Solirubrobacterales	Solirubrobacteraceae	Conexibacter_A
ASV273	Glacial	LacAmpRUFtf-a	0	Bacteria	Actinobacteriota	Thermoleophilia	Solirubrobacterales	Solirubrobacteraceae	Conexibacter_A
ASV273	Glacial	LacAmpRUFtf-b	0	Bacteria	Actinobacteriota	Thermoleophilia	Solirubrobacterales	Solirubrobacteraceae	Conexibacter_A
ASV273	Glacial	LacAmpRUFtf-c	0	Bacteria	Actinobacteriota	Thermoleophilia	Solirubrobacterales	Solirubrobacteraceae	Conexibacter_A
ASV275	Baie de la Table	BdT0-2	0	Bacteria	Proteobacteria	Gammaproteobacteria	Betaproteobacteriales	Burkholderiaceae	Mitsuaria
ASV275	Control	Ctr-tf-IIb	0	Bacteria	Proteobacteria	Gammaproteobacteria	Betaproteobacteriales	Burkholderiaceae	Mitsuaria

ASV275	Non-glacial	LacADNRUFtfa	0	Bacteria	Proteobacteria	Gammaproteobacteria	Betaproteobacteriales	Burkholderiaceae	Mitsuaria
ASV275	Non-glacial	LacADNRUFtfb	0	Bacteria	Proteobacteria	Gammaproteobacteria	Betaproteobacteriales	Burkholderiaceae	Mitsuaria
ASV275	Non-glacial	LacADNRUFtfc	0	Bacteria	Proteobacteria	Gammaproteobacteria	Betaproteobacteriales	Burkholderiaceae	Mitsuaria
ASV275	Glacial	LacAmpRUFtf-a	0	Bacteria	Proteobacteria	Gammaproteobacteria	Betaproteobacteriales	Burkholderiaceae	Mitsuaria
ASV275	Glacial	LacAmpRUFtf-b	0	Bacteria	Proteobacteria	Gammaproteobacteria	Betaproteobacteriales	Burkholderiaceae	Mitsuaria
ASV275	Glacial	LacAmpRUFtf-c	0	Bacteria	Proteobacteria	Gammaproteobacteria	Betaproteobacteriales	Burkholderiaceae	Mitsuaria
ASV277	Baie de la Table	BdT0-2	0	Bacteria	Verrucomicrobio	Verrucomicrobiae	Verrucomicrobiales	Verrucomicrobiaceae	Prosthecobacter
ASV277	Control	Ctr-tf-IIb	0	Bacteria	Verrucomicrobio	Verrucomicrobiae	Verrucomicrobiales	Verrucomicrobiaceae	Prosthecobacter
ASV277	Non-glacial	LacADNRUFtfa	0	Bacteria	Verrucomicrobio	Verrucomicrobiae	Verrucomicrobiales	Verrucomicrobiaceae	Prosthecobacter
ASV277	Non-glacial	LacADNRUFtfb	0	Bacteria	Verrucomicrobio	Verrucomicrobiae	Verrucomicrobiales	Verrucomicrobiaceae	Prosthecobacter
ASV277	Non-glacial	LacADNRUFtfc	0	Bacteria	Verrucomicrobio	Verrucomicrobiae	Verrucomicrobiales	Verrucomicrobiaceae	Prosthecobacter
ASV277	Glacial	LacAmpRUFtf-a	0	Bacteria	Verrucomicrobio	Verrucomicrobiae	Verrucomicrobiales	Verrucomicrobiaceae	Prosthecobacter
ASV277	Glacial	LacAmpRUFtf-b	0	Bacteria	Verrucomicrobio	Verrucomicrobiae	Verrucomicrobiales	Verrucomicrobiaceae	Prosthecobacter
ASV277	Glacial	LacAmpRUFtf-c	0	Bacteria	Verrucomicrobio	Verrucomicrobiae	Verrucomicrobiales	Verrucomicrobiaceae	Prosthecobacter
ASV28	Baie de la Table	BdT0-2	0,041479943	Bacteria	Bacteroidota	Bacteroidia	Flavobacteriales	Flavobacteriaceae	MS024-2A
ASV28	Non-glacial	LacADNRUFtfa	0,009700217	Bacteria	Bacteroidota	Bacteroidia	Flavobacteriales	Flavobacteriaceae	MS024-2A
ASV28	Non-glacial	LacADNRUFtfb	0,008429926	Bacteria	Bacteroidota	Bacteroidia	Flavobacteriales	Flavobacteriaceae	MS024-2A
ASV28	Control	Ctr-tf-IIb	0,005617235	Bacteria	Bacteroidota	Bacteroidia	Flavobacteriales	Flavobacteriaceae	MS024-2A
ASV28	Glacial	LacAmpRUFtf-a	0,003335477	Bacteria	Bacteroidota	Bacteroidia	Flavobacteriales	Flavobacteriaceae	MS024-2A
ASV28	Non-glacial	LacADNRUFtfc	0,000606208	Bacteria	Bacteroidota	Bacteroidia	Flavobacteriales	Flavobacteriaceae	MS024-2A
ASV28	Glacial	LacAmpRUFtf-b	0,000539898	Bacteria	Bacteroidota	Bacteroidia	Flavobacteriales	Flavobacteriaceae	MS024-2A
ASV28	Glacial	LacAmpRUFtf-c	0	Bacteria	Bacteroidota	Bacteroidia	Flavobacteriales	Flavobacteriaceae	MS024-2A
ASV285	Glacial	LacAmpRUFtf-b	0,021272001	Bacteria	Firmicutes	Bacilli	Mycoplasmatales	Ureaplasmataceae	Mycoplasma_A
ASV285	Glacial	LacAmpRUFtf-c	0,009053498	Bacteria	Firmicutes	Bacilli	Mycoplasmatales	Ureaplasmataceae	Mycoplasma_A
ASV285	Non-glacial	LacADNRUFtfa	0,001385745	Bacteria	Firmicutes	Bacilli	Mycoplasmatales	Ureaplasmataceae	Mycoplasma_A
ASV285	Non-glacial	LacADNRUFtfb	0,001159115	Bacteria	Firmicutes	Bacilli	Mycoplasmatales	Ureaplasmataceae	Mycoplasma_A
ASV285	Glacial	LacAmpRUFtf-a	0,00068317	Bacteria	Firmicutes	Bacilli	Mycoplasmatales	Ureaplasmataceae	Mycoplasma_A
ASV285	Non-glacial	LacADNRUFtfc	0,000169738	Bacteria	Firmicutes	Bacilli	Mycoplasmatales	Ureaplasmataceae	Mycoplasma_A
ASV285	Baie de la Table	BdT0-2	0	Bacteria	Firmicutes	Bacilli	Mycoplasmatales	Ureaplasmataceae	Mycoplasma_A
ASV285	Control	Ctr-tf-IIb	0	Bacteria	Firmicutes	Bacilli	Mycoplasmatales	Ureaplasmataceae	Mycoplasma_A
ASV29	Baie de la Table	BdT0-2	0,018799869	Bacteria	Proteobacteria	Gammaproteobacteria	Thiomicrospirales	Thioglobaceae	Thioglobus
ASV29	Non-glacial	LacADNRUFtfb	0,0028451	Bacteria	Proteobacteria	Gammaproteobacteria	Thiomicrospirales	Thioglobaceae	Thioglobus
ASV29	Non-glacial	LacADNRUFtfa	0,002771491	Bacteria	Proteobacteria	Gammaproteobacteria	Thiomicrospirales	Thioglobaceae	Thioglobus
ASV29	Glacial	LacAmpRUFtf-b	0,002483533	Bacteria	Proteobacteria	Gammaproteobacteria	Thiomicrospirales	Thioglobaceae	Thioglobus

ASV29	Control	Ctr-tf-IIb	0,000945554	Bacteria	Proteobacteria	Gammaproteobacteria	Thiomicrospirales	Thioglobaceae	Thioglobus
ASV29	Non-glacial	LacADNRUFtfc	0,000703201	Bacteria	Proteobacteria	Gammaproteobacteria	Thiomicrospirales	Thioglobaceae	Thioglobus
ASV29	Glacial	LacAmpRUFtf-a	0,000241119	Bacteria	Proteobacteria	Gammaproteobacteria	Thiomicrospirales	Thioglobaceae	Thioglobus
ASV29	Glacial	LacAmpRUFtf-c	0,000193658	Bacteria	Proteobacteria	Gammaproteobacteria	Thiomicrospirales	Thioglobaceae	Thioglobus
ASV296	Glacial	LacAmpRUFtf-c	0,000726216	Bacteria	Proteobacteria	Alphaproteobacteria	Caulobacterales	Caulobacteraceae	Brevundimonas
ASV296	Glacial	LacAmpRUFtf-b	0,000539898	Bacteria	Proteobacteria	Alphaproteobacteria	Caulobacterales	Caulobacteraceae	Brevundimonas
ASV296	Non-glacial	LacADNRUFtfb	0,000210748	Bacteria	Proteobacteria	Alphaproteobacteria	Caulobacterales	Caulobacteraceae	Brevundimonas
ASV296	Control	Ctr-tf-IIb	9,62E-05	Bacteria	Proteobacteria	Alphaproteobacteria	Caulobacterales	Caulobacteraceae	Brevundimonas
ASV296	Baie de la Table	BdT0-2	0	Bacteria	Proteobacteria	Alphaproteobacteria	Caulobacterales	Caulobacteraceae	Brevundimonas
ASV296	Non-glacial	LacADNRUFtfa	0	Bacteria	Proteobacteria	Alphaproteobacteria	Caulobacterales	Caulobacteraceae	Brevundimonas
ASV296	Non-glacial	LacADNRUFtfc	0	Bacteria	Proteobacteria	Alphaproteobacteria	Caulobacterales	Caulobacteraceae	Brevundimonas
ASV296	Glacial	LacAmpRUFtf-a	0	Bacteria	Proteobacteria	Alphaproteobacteria	Caulobacterales	Caulobacteraceae	Brevundimonas
ASV297	Baie de la Table	BdT0-2	0	Bacteria	Proteobacteria	Gammaproteobacteria	Betaproteobacteriales	Burkholderiaceae	Herbaspirillum
ASV297	Control	Ctr-tf-IIb	0	Bacteria	Proteobacteria	Gammaproteobacteria	Betaproteobacteriales	Burkholderiaceae	Herbaspirillum
ASV297	Non-glacial	LacADNRUFtfa	0	Bacteria	Proteobacteria	Gammaproteobacteria	Betaproteobacteriales	Burkholderiaceae	Herbaspirillum
ASV297	Non-glacial	LacADNRUFtfb	0	Bacteria	Proteobacteria	Gammaproteobacteria	Betaproteobacteriales	Burkholderiaceae	Herbaspirillum
ASV297	Non-glacial	LacADNRUFtfc	0	Bacteria	Proteobacteria	Gammaproteobacteria	Betaproteobacteriales	Burkholderiaceae	Herbaspirillum
ASV297	Glacial	LacAmpRUFtf-a	0	Bacteria	Proteobacteria	Gammaproteobacteria	Betaproteobacteriales	Burkholderiaceae	Herbaspirillum
ASV297	Glacial	LacAmpRUFtf-b	0	Bacteria	Proteobacteria	Gammaproteobacteria	Betaproteobacteriales	Burkholderiaceae	Herbaspirillum
ASV297	Glacial	LacAmpRUFtf-c	0	Bacteria	Proteobacteria	Gammaproteobacteria	Betaproteobacteriales	Burkholderiaceae	Herbaspirillum
ASV3	Baie de la Table	BdT0-2	0,436878347	Bacteria	Proteobacteria	Alphaproteobacteria	Pelagibacterales	Pelagibacteraceae	Pelagibacter
ASV3	Non-glacial	LacADNRUFtfa	0,028777311	Bacteria	Proteobacteria	Alphaproteobacteria	Pelagibacterales	Pelagibacteraceae	Pelagibacter
ASV3	Non-glacial	LacADNRUFtfb	0,01938883	Bacteria	Proteobacteria	Alphaproteobacteria	Pelagibacterales	Pelagibacteraceae	Pelagibacter
ASV3	Glacial	LacAmpRUFtf-b	0,016628874	Bacteria	Proteobacteria	Alphaproteobacteria	Pelagibacterales	Pelagibacteraceae	Pelagibacter
ASV3	Glacial	LacAmpRUFtf-a	0,011533516	Bacteria	Proteobacteria	Alphaproteobacteria	Pelagibacterales	Pelagibacteraceae	Pelagibacter
ASV3	Control	Ctr-tf-IIb	0,009864217	Bacteria	Proteobacteria	Alphaproteobacteria	Pelagibacterales	Pelagibacteraceae	Pelagibacter
ASV3	Non-glacial	LacADNRUFtfc	0,00785645	Bacteria	Proteobacteria	Alphaproteobacteria	Pelagibacterales	Pelagibacteraceae	Pelagibacter
ASV3	Glacial	LacAmpRUFtf-c	0,002565965	Bacteria	Proteobacteria	Alphaproteobacteria	Pelagibacterales	Pelagibacteraceae	Pelagibacter
ASV30	Baie de la Table	BdT0-2	0,016449885	Bacteria	Bacteroidota	Bacteroidia	Flavobacteriales	Flavobacteriaceae	Hel1-33-131
ASV30	Control	Ctr-tf-IIb	0,009559716	Bacteria	Bacteroidota	Bacteroidia	Flavobacteriales	Flavobacteriaceae	Hel1-33-131
ASV30	Non-glacial	LacADNRUFtfb	0,005542677	Bacteria	Bacteroidota	Bacteroidia	Flavobacteriales	Flavobacteriaceae	Hel1-33-131
ASV30	Glacial	LacAmpRUFtf-b	0,003347371	Bacteria	Bacteroidota	Bacteroidia	Flavobacteriales	Flavobacteriaceae	Hel1-33-131
ASV30	Non-glacial	LacADNRUFtfc	0,003128031	Bacteria	Bacteroidota	Bacteroidia	Flavobacteriales	Flavobacteriaceae	Hel1-33-131
ASV30	Non-glacial	LacADNRUFtfa	0,002586725	Bacteria	Bacteroidota	Bacteroidia	Flavobacteriales	Flavobacteriaceae	Hel1-33-131

ASV30	Glacial	LacAmpRUFtf-a	0,001004662	Bacteria	Bacteroidota	Bacteroidia	Flavobacteriales	Flavobacteriaceae	Hel1-33-131
ASV30	Glacial	LacAmpRUFtf-c	0	Bacteria	Bacteroidota	Bacteroidia	Flavobacteriales	Flavobacteriaceae	Hel1-33-131
ASV300	Baie de la Table	BdT0-2	0	Bacteria	Verrucomicrobio	Verrucomicrobiae	Opitutales	Opitutaceae	GCF-2310495
ASV300	Control	Ctr-tf-IIb	0	Bacteria	Verrucomicrobio	Verrucomicrobiae	Opitutales	Opitutaceae	GCF-2310495
ASV300	Non-glacial	LacADNRUFtfa	0	Bacteria	Verrucomicrobio	Verrucomicrobiae	Opitutales	Opitutaceae	GCF-2310495
ASV300	Non-glacial	LacADNRUFtfb	0	Bacteria	Verrucomicrobio	Verrucomicrobiae	Opitutales	Opitutaceae	GCF-2310495
ASV300	Non-glacial	LacADNRUFtfc	0	Bacteria	Verrucomicrobio	Verrucomicrobiae	Opitutales	Opitutaceae	GCF-2310495
ASV300	Glacial	LacAmpRUFtf-a	0	Bacteria	Verrucomicrobio	Verrucomicrobiae	Opitutales	Opitutaceae	GCF-2310495
ASV300	Glacial	LacAmpRUFtf-b	0	Bacteria	Verrucomicrobio	Verrucomicrobiae	Opitutales	Opitutaceae	GCF-2310495
ASV300	Glacial	LacAmpRUFtf-c	0	Bacteria	Verrucomicrobio	Verrucomicrobiae	Opitutales	Opitutaceae	GCF-2310495
ASV303	Glacial	LacAmpRUFtf-c	0,000290487	Bacteria	Gemmatimonad	Gemmatimonadetes	Gemmatimonadales	Gemmatimonadaceae	Gemmatimonas
ASV303	Baie de la Table	BdT0-2	0	Bacteria	Gemmatimonad	Gemmatimonadetes	Gemmatimonadales	Gemmatimonadaceae	Gemmatimonas
ASV303	Control	Ctr-tf-IIb	0	Bacteria	Gemmatimonad	Gemmatimonadetes	Gemmatimonadales	Gemmatimonadaceae	Gemmatimonas
ASV303	Non-glacial	LacADNRUFtfa	0	Bacteria	Gemmatimonad	Gemmatimonadetes	Gemmatimonadales	Gemmatimonadaceae	Gemmatimonas
ASV303	Non-glacial	LacADNRUFtfb	0	Bacteria	Gemmatimonad	Gemmatimonadetes	Gemmatimonadales	Gemmatimonadaceae	Gemmatimonas
ASV303	Non-glacial	LacADNRUFtfc	0	Bacteria	Gemmatimonad	Gemmatimonadetes	Gemmatimonadales	Gemmatimonadaceae	Gemmatimonas
ASV303	Glacial	LacAmpRUFtf-a	0	Bacteria	Gemmatimonad	Gemmatimonadetes	Gemmatimonadales	Gemmatimonadaceae	Gemmatimonas
ASV303	Glacial	LacAmpRUFtf-b	0	Bacteria	Gemmatimonad	Gemmatimonadetes	Gemmatimonadales	Gemmatimonadaceae	Gemmatimonas
ASV310	Baie de la Table	BdT0-2	0	Bacteria	Proteobacteria	Gammaproteobacteria	Betaproteobacteriales	Burkholderiaceae	Herminiimonas
ASV310	Control	Ctr-tf-IIb	0	Bacteria	Proteobacteria	Gammaproteobacteria	Betaproteobacteriales	Burkholderiaceae	Herminiimonas
ASV310	Non-glacial	LacADNRUFtfa	0	Bacteria	Proteobacteria	Gammaproteobacteria	Betaproteobacteriales	Burkholderiaceae	Herminiimonas
ASV310	Non-glacial	LacADNRUFtfb	0	Bacteria	Proteobacteria	Gammaproteobacteria	Betaproteobacteriales	Burkholderiaceae	Herminiimonas
ASV310	Non-glacial	LacADNRUFtfc	0	Bacteria	Proteobacteria	Gammaproteobacteria	Betaproteobacteriales	Burkholderiaceae	Herminiimonas
ASV310	Glacial	LacAmpRUFtf-a	0	Bacteria	Proteobacteria	Gammaproteobacteria	Betaproteobacteriales	Burkholderiaceae	Herminiimonas
ASV310	Glacial	LacAmpRUFtf-b	0	Bacteria	Proteobacteria	Gammaproteobacteria	Betaproteobacteriales	Burkholderiaceae	Herminiimonas
ASV310	Glacial	LacAmpRUFtf-c	0	Bacteria	Proteobacteria	Gammaproteobacteria	Betaproteobacteriales	Burkholderiaceae	Herminiimonas
ASV311	Glacial	LacAmpRUFtf-b	0,000215959	Bacteria	Proteobacteria	Gammaproteobacteria	Nitrosococcales	Methylophagaceae	Methylophaga
ASV311	Baie de la Table	BdT0-2	0	Bacteria	Proteobacteria	Gammaproteobacteria	Nitrosococcales	Methylophagaceae	Methylophaga
ASV311	Control	Ctr-tf-IIb	0	Bacteria	Proteobacteria	Gammaproteobacteria	Nitrosococcales	Methylophagaceae	Methylophaga
ASV311	Non-glacial	LacADNRUFtfa	0	Bacteria	Proteobacteria	Gammaproteobacteria	Nitrosococcales	Methylophagaceae	Methylophaga
ASV311	Non-glacial	LacADNRUFtfb	0	Bacteria	Proteobacteria	Gammaproteobacteria	Nitrosococcales	Methylophagaceae	Methylophaga
ASV311	Non-glacial	LacADNRUFtfc	0	Bacteria	Proteobacteria	Gammaproteobacteria	Nitrosococcales	Methylophagaceae	Methylophaga
ASV311	Glacial	LacAmpRUFtf-a	0	Bacteria	Proteobacteria	Gammaproteobacteria	Nitrosococcales	Methylophagaceae	Methylophaga
ASV311	Glacial	LacAmpRUFtf-c	0	Bacteria	Proteobacteria	Gammaproteobacteria	Nitrosococcales	Methylophagaceae	Methylophaga

ASV313	Glacial	LacAmpRUFtf-a	0,000884102	Bacteria	Proteobacteria	Alphaproteobacteria	Rhodobacterales	Rhodobacteraceae	Litoreibacter
ASV313	Glacial	LacAmpRUFtf-b	0,000755858	Bacteria	Proteobacteria	Alphaproteobacteria	Rhodobacterales	Rhodobacteraceae	Litoreibacter
ASV313	Baie de la Table	BdT0-2	0,000601159	Bacteria	Proteobacteria	Alphaproteobacteria	Rhodobacterales	Rhodobacteraceae	Litoreibacter
ASV313	Glacial	LacAmpRUFtf-c	0,000580973	Bacteria	Proteobacteria	Alphaproteobacteria	Rhodobacterales	Rhodobacteraceae	Litoreibacter
ASV313	Control	Ctr-tf-IIb	0	Bacteria	Proteobacteria	Alphaproteobacteria	Rhodobacterales	Rhodobacteraceae	Litoreibacter
ASV313	Non-glacial	LacADNRUFtfa	0	Bacteria	Proteobacteria	Alphaproteobacteria	Rhodobacterales	Rhodobacteraceae	Litoreibacter
ASV313	Non-glacial	LacADNRUFtfb	0	Bacteria	Proteobacteria	Alphaproteobacteria	Rhodobacterales	Rhodobacteraceae	Litoreibacter
ASV313	Non-glacial	LacADNRUFtfc	0	Bacteria	Proteobacteria	Alphaproteobacteria	Rhodobacterales	Rhodobacteraceae	Litoreibacter
ASV314	Non-glacial	LacADNRUFtfa	0,001662894	Bacteria	Proteobacteria	Gammaproteobacteria	Betaproteobacteriales	Burkholderiaceae	Rhodoferax
ASV314	Non-glacial	LacADNRUFtfb	0,001074816	Bacteria	Proteobacteria	Gammaproteobacteria	Betaproteobacteriales	Burkholderiaceae	Rhodoferax
ASV314	Non-glacial	LacADNRUFtfc	0,000193986	Bacteria	Proteobacteria	Gammaproteobacteria	Betaproteobacteriales	Burkholderiaceae	Rhodoferax
ASV314	Baie de la Table	BdT0-2	0	Bacteria	Proteobacteria	Gammaproteobacteria	Betaproteobacteriales	Burkholderiaceae	Rhodoferax
ASV314	Control	Ctr-tf-IIb	0	Bacteria	Proteobacteria	Gammaproteobacteria	Betaproteobacteriales	Burkholderiaceae	Rhodoferax
ASV314	Glacial	LacAmpRUFtf-a	0	Bacteria	Proteobacteria	Gammaproteobacteria	Betaproteobacteriales	Burkholderiaceae	Rhodoferax
ASV314	Glacial	LacAmpRUFtf-b	0	Bacteria	Proteobacteria	Gammaproteobacteria	Betaproteobacteriales	Burkholderiaceae	Rhodoferax
ASV314	Glacial	LacAmpRUFtf-c	0	Bacteria	Proteobacteria	Gammaproteobacteria	Betaproteobacteriales	Burkholderiaceae	Rhodoferax
ASV32	Glacial	LacAmpRUFtf-c	0,001113532	Bacteria	Planctomycetota	Planctomycetes	Pirellulales	Pirellulaceae	UBA721
ASV32	Baie de la Table	BdT0-2	0	Bacteria	Planctomycetota	Planctomycetes	Pirellulales	Pirellulaceae	UBA721
ASV32	Control	Ctr-tf-IIb	0	Bacteria	Planctomycetota	Planctomycetes	Pirellulales	Pirellulaceae	UBA721
ASV32	Non-glacial	LacADNRUFtfa	0	Bacteria	Planctomycetota	Planctomycetes	Pirellulales	Pirellulaceae	UBA721
ASV32	Non-glacial	LacADNRUFtfb	0	Bacteria	Planctomycetota	Planctomycetes	Pirellulales	Pirellulaceae	UBA721
ASV32	Non-glacial	LacADNRUFtfc	0	Bacteria	Planctomycetota	Planctomycetes	Pirellulales	Pirellulaceae	UBA721
ASV32	Glacial	LacAmpRUFtf-a	0	Bacteria	Planctomycetota	Planctomycetes	Pirellulales	Pirellulaceae	UBA721
ASV32	Glacial	LacAmpRUFtf-b	0	Bacteria	Planctomycetota	Planctomycetes	Pirellulales	Pirellulaceae	UBA721
ASV320	Baie de la Table	BdT0-2	0,000437206	Bacteria	Proteobacteria	Gammaproteobacteria	Enterobacterales	Psychromonadaceae	Psychromonas
ASV320	Non-glacial	LacADNRUFtfa	0,000184766	Bacteria	Proteobacteria	Gammaproteobacteria	Enterobacterales	Psychromonadaceae	Psychromonas
ASV320	Non-glacial	LacADNRUFtfc	0,00014549	Bacteria	Proteobacteria	Gammaproteobacteria	Enterobacterales	Psychromonadaceae	Psychromonas
ASV320	Control	Ctr-tf-IIb	0	Bacteria	Proteobacteria	Gammaproteobacteria	Enterobacterales	Psychromonadaceae	Psychromonas
ASV320	Non-glacial	LacADNRUFtfb	0	Bacteria	Proteobacteria	Gammaproteobacteria	Enterobacterales	Psychromonadaceae	Psychromonas
ASV320	Glacial	LacAmpRUFtf-a	0	Bacteria	Proteobacteria	Gammaproteobacteria	Enterobacterales	Psychromonadaceae	Psychromonas
ASV320	Glacial	LacAmpRUFtf-b	0	Bacteria	Proteobacteria	Gammaproteobacteria	Enterobacterales	Psychromonadaceae	Psychromonas
ASV320	Glacial	LacAmpRUFtf-c	0	Bacteria	Proteobacteria	Gammaproteobacteria	Enterobacterales	Psychromonadaceae	Psychromonas
ASV324	Glacial	LacAmpRUFtf-c	0,006632777	Bacteria	Firmicutes	Bacilli	Lactobacillales	Streptococcaceae	Streptococcus
ASV324	Glacial	LacAmpRUFtf-b	0,000755858	Bacteria	Firmicutes	Bacilli	Lactobacillales	Streptococcaceae	Streptococcus

ASV324	Non-glacial	LacADNRUFtfb	0,000547945	Bacteria	Firmicutes	Bacilli	Lactobacillales	Streptococcaceae	Streptococcus
ASV324	Glacial	LacAmpRUFtf-a	0,000361678	Bacteria	Firmicutes	Bacilli	Lactobacillales	Streptococcaceae	Streptococcus
ASV324	Non-glacial	LacADNRUFtfc	7,27E-05	Bacteria	Firmicutes	Bacilli	Lactobacillales	Streptococcaceae	Streptococcus
ASV324	Baie de la Table	BdT0-2	0	Bacteria	Firmicutes	Bacilli	Lactobacillales	Streptococcaceae	Streptococcus
ASV324	Control	Ctr-tf-IIb	0	Bacteria	Firmicutes	Bacilli	Lactobacillales	Streptococcaceae	Streptococcus
ASV324	Non-glacial	LacADNRUFtfa	0	Bacteria	Firmicutes	Bacilli	Lactobacillales	Streptococcaceae	Streptococcus
ASV329	Glacial	LacAmpRUFtf-a	0,00659058	Bacteria	Bacteroidota	Rhodothermia	Balneolales	Balneolaceae	UBA1275
ASV329	Baie de la Table	BdT0-2	0	Bacteria	Bacteroidota	Rhodothermia	Balneolales	Balneolaceae	UBA1275
ASV329	Control	Ctr-tf-IIb	0	Bacteria	Bacteroidota	Rhodothermia	Balneolales	Balneolaceae	UBA1275
ASV329	Non-glacial	LacADNRUFtfa	0	Bacteria	Bacteroidota	Rhodothermia	Balneolales	Balneolaceae	UBA1275
ASV329	Non-glacial	LacADNRUFtfb	0	Bacteria	Bacteroidota	Rhodothermia	Balneolales	Balneolaceae	UBA1275
ASV329	Non-glacial	LacADNRUFtfc	0	Bacteria	Bacteroidota	Rhodothermia	Balneolales	Balneolaceae	UBA1275
ASV329	Glacial	LacAmpRUFtf-b	0	Bacteria	Bacteroidota	Rhodothermia	Balneolales	Balneolaceae	UBA1275
ASV329	Glacial	LacAmpRUFtf-c	0	Bacteria	Bacteroidota	Rhodothermia	Balneolales	Balneolaceae	UBA1275
ASV332	Non-glacial	LacADNRUFtfb	0,000147524	Bacteria	Proteobacteria	Gammaproteobacteria	Enterobacterales	Alteromonadaceae	Paraglaciecola
ASV332	Control	Ctr-tf-IIb	0,000100165	Bacteria	Proteobacteria	Gammaproteobacteria	Enterobacterales	Alteromonadaceae	Paraglaciecola
ASV332	Baie de la Table	BdT0-2	0	Bacteria	Proteobacteria	Gammaproteobacteria	Enterobacterales	Alteromonadaceae	Paraglaciecola
ASV332	Non-glacial	LacADNRUFtfa	0	Bacteria	Proteobacteria	Gammaproteobacteria	Enterobacterales	Alteromonadaceae	Paraglaciecola
ASV332	Non-glacial	LacADNRUFtfc	0	Bacteria	Proteobacteria	Gammaproteobacteria	Enterobacterales	Alteromonadaceae	Paraglaciecola
ASV332	Glacial	LacAmpRUFtf-a	0	Bacteria	Proteobacteria	Gammaproteobacteria	Enterobacterales	Alteromonadaceae	Paraglaciecola
ASV332	Glacial	LacAmpRUFtf-b	0	Bacteria	Proteobacteria	Gammaproteobacteria	Enterobacterales	Alteromonadaceae	Paraglaciecola
ASV332	Glacial	LacAmpRUFtf-c	0	Bacteria	Proteobacteria	Gammaproteobacteria	Enterobacterales	Alteromonadaceae	Paraglaciecola
ASV343	Baie de la Table	BdT0-2	0	Bacteria	Proteobacteria	Gammaproteobacteria	Betaproteobacteriales	Burkholderiaceae	Acidovorax
ASV343	Control	Ctr-tf-IIb	0	Bacteria	Proteobacteria	Gammaproteobacteria	Betaproteobacteriales	Burkholderiaceae	Acidovorax
ASV343	Non-glacial	LacADNRUFtfa	0	Bacteria	Proteobacteria	Gammaproteobacteria	Betaproteobacteriales	Burkholderiaceae	Acidovorax
ASV343	Non-glacial	LacADNRUFtfb	0	Bacteria	Proteobacteria	Gammaproteobacteria	Betaproteobacteriales	Burkholderiaceae	Acidovorax
ASV343	Non-glacial	LacADNRUFtfc	0	Bacteria	Proteobacteria	Gammaproteobacteria	Betaproteobacteriales	Burkholderiaceae	Acidovorax
ASV343	Glacial	LacAmpRUFtf-a	0	Bacteria	Proteobacteria	Gammaproteobacteria	Betaproteobacteriales	Burkholderiaceae	Acidovorax
ASV343	Glacial	LacAmpRUFtf-b	0	Bacteria	Proteobacteria	Gammaproteobacteria	Betaproteobacteriales	Burkholderiaceae	Acidovorax
ASV343	Glacial	LacAmpRUFtf-c	0	Bacteria	Proteobacteria	Gammaproteobacteria	Betaproteobacteriales	Burkholderiaceae	Acidovorax
ASV349	Non-glacial	LacADNRUFtfb	0,000231823	Bacteria	Actinobacteriota	Actinobacteria	Propionibacteriales	Nocardioidaceae	Nocardioides
ASV349	Glacial	LacAmpRUFtf-a	8,04E-05	Bacteria	Actinobacteriota	Actinobacteria	Propionibacteriales	Nocardioidaceae	Nocardioides
ASV349	Baie de la Table	BdT0-2	0	Bacteria	Actinobacteriota	Actinobacteria	Propionibacteriales	Nocardioidaceae	Nocardioides
ASV349	Control	Ctr-tf-IIb	0	Bacteria	Actinobacteriota	Actinobacteria	Propionibacteriales	Nocardioidaceae	Nocardioides

ASV349	Non-glacial	LacADNRUFtfa	0	Bacteria	Actinobacteriota	Actinobacteria	Propionibacteriales	Nocardioidaceae	Nocardioides
ASV349	Non-glacial	LacADNRUFtfc	0	Bacteria	Actinobacteriota	Actinobacteria	Propionibacteriales	Nocardioidaceae	Nocardioides
ASV349	Glacial	LacAmpRUFtf-b	0	Bacteria	Actinobacteriota	Actinobacteria	Propionibacteriales	Nocardioidaceae	Nocardioides
ASV349	Glacial	LacAmpRUFtf-c	0	Bacteria	Actinobacteriota	Actinobacteria	Propionibacteriales	Nocardioidaceae	Nocardioides
ASV350	Non-glacial	LacADNRUFtfb	0,000168599	Bacteria	Actinobacteriota	Actinobacteria	Nanopelagicales	S36-B12	S36-B12
ASV350	Control	Ctr-tf-IIb	0,000140231	Bacteria	Actinobacteriota	Actinobacteria	Nanopelagicales	S36-B12	S36-B12
ASV350	Non-glacial	LacADNRUFtfa	9,24E-05	Bacteria	Actinobacteriota	Actinobacteria	Nanopelagicales	S36-B12	S36-B12
ASV350	Baie de la Table	BdT0-2	0	Bacteria	Actinobacteriota	Actinobacteria	Nanopelagicales	S36-B12	S36-B12
ASV350	Non-glacial	LacADNRUFtfc	0	Bacteria	Actinobacteriota	Actinobacteria	Nanopelagicales	S36-B12	S36-B12
ASV350	Glacial	LacAmpRUFtf-a	0	Bacteria	Actinobacteriota	Actinobacteria	Nanopelagicales	S36-B12	S36-B12
ASV350	Glacial	LacAmpRUFtf-b	0	Bacteria	Actinobacteriota	Actinobacteria	Nanopelagicales	S36-B12	S36-B12
ASV350	Glacial	LacAmpRUFtf-c	0	Bacteria	Actinobacteriota	Actinobacteria	Nanopelagicales	S36-B12	S36-B12
ASV356	Baie de la Table	BdT0-2	0	Bacteria	Proteobacteria	Alphaproteobacteria	Rhizobiales	Hyphomicrobiaceae	Hyphomicrobium
ASV356	Control	Ctr-tf-IIb	0	Bacteria	Proteobacteria	Alphaproteobacteria	Rhizobiales	Hyphomicrobiaceae	Hyphomicrobium
ASV356	Non-glacial	LacADNRUFtfa	0	Bacteria	Proteobacteria	Alphaproteobacteria	Rhizobiales	Hyphomicrobiaceae	Hyphomicrobium
ASV356	Non-glacial	LacADNRUFtfb	0	Bacteria	Proteobacteria	Alphaproteobacteria	Rhizobiales	Hyphomicrobiaceae	Hyphomicrobium
ASV356	Non-glacial	LacADNRUFtfc	0	Bacteria	Proteobacteria	Alphaproteobacteria	Rhizobiales	Hyphomicrobiaceae	Hyphomicrobium
ASV356	Glacial	LacAmpRUFtf-a	0	Bacteria	Proteobacteria	Alphaproteobacteria	Rhizobiales	Hyphomicrobiaceae	Hyphomicrobium
ASV356	Glacial	LacAmpRUFtf-b	0	Bacteria	Proteobacteria	Alphaproteobacteria	Rhizobiales	Hyphomicrobiaceae	Hyphomicrobium
ASV356	Glacial	LacAmpRUFtf-c	0	Bacteria	Proteobacteria	Alphaproteobacteria	Rhizobiales	Hyphomicrobiaceae	Hyphomicrobium
ASV357	Baie de la Table	BdT0-2	0	Bacteria	Acidobacteriota	Acidobacteriae	Solibacterales	Solibacteraceae	Bryobacter
ASV357	Control	Ctr-tf-IIb	0	Bacteria	Acidobacteriota	Acidobacteriae	Solibacterales	Solibacteraceae	Bryobacter
ASV357	Non-glacial	LacADNRUFtfa	0	Bacteria	Acidobacteriota	Acidobacteriae	Solibacterales	Solibacteraceae	Bryobacter
ASV357	Non-glacial	LacADNRUFtfb	0	Bacteria	Acidobacteriota	Acidobacteriae	Solibacterales	Solibacteraceae	Bryobacter
ASV357	Non-glacial	LacADNRUFtfc	0	Bacteria	Acidobacteriota	Acidobacteriae	Solibacterales	Solibacteraceae	Bryobacter
ASV357	Glacial	LacAmpRUFtf-a	0	Bacteria	Acidobacteriota	Acidobacteriae	Solibacterales	Solibacteraceae	Bryobacter
ASV357	Glacial	LacAmpRUFtf-b	0	Bacteria	Acidobacteriota	Acidobacteriae	Solibacterales	Solibacteraceae	Bryobacter
ASV357	Glacial	LacAmpRUFtf-c	0	Bacteria	Acidobacteriota	Acidobacteriae	Solibacterales	Solibacteraceae	Bryobacter
ASV36	Baie de la Table	BdT0-2	0,011039458	Bacteria	Bacteroidota	Bacteroidia	Flavobacteriales	UA16	UA16
ASV36	Non-glacial	LacADNRUFtfa	0,007529216	Bacteria	Bacteroidota	Bacteroidia	Flavobacteriales	UA16	UA16
ASV36	Non-glacial	LacADNRUFtfb	0,006954689	Bacteria	Bacteroidota	Bacteroidia	Flavobacteriales	UA16	UA16
ASV36	Control	Ctr-tf-IIb	0,003045006	Bacteria	Bacteroidota	Bacteroidia	Flavobacteriales	UA16	UA16
ASV36	Glacial	LacAmpRUFtf-b	0,001943635	Bacteria	Bacteroidota	Bacteroidia	Flavobacteriales	UA16	UA16
ASV36	Glacial	LacAmpRUFtf-a	0,000763543	Bacteria	Bacteroidota	Bacteroidia	Flavobacteriales	UA16	UA16

ASV36	Non-glacial	LacADNRUFtfc	0,000630456	Bacteria	Bacteroidota	Bacteroidia	Flavobacteriales	UA16	UA16
ASV36	Glacial	LacAmpRUFtf-c	0	Bacteria	Bacteroidota	Bacteroidia	Flavobacteriales	UA16	UA16
ASV362	Baie de la Table	BdT0-2	0	Bacteria	Actinobacteriota	Thermoleophilia	Solirubrobacterales	Solirubrobacteraceae	Solirubrobacter
ASV362	Control	Ctr-tf-IIb	0	Bacteria	Actinobacteriota	Thermoleophilia	Solirubrobacterales	Solirubrobacteraceae	Solirubrobacter
ASV362	Non-glacial	LacADNRUFtfa	0	Bacteria	Actinobacteriota	Thermoleophilia	Solirubrobacterales	Solirubrobacteraceae	Solirubrobacter
ASV362	Non-glacial	LacADNRUFtfb	0	Bacteria	Actinobacteriota	Thermoleophilia	Solirubrobacterales	Solirubrobacteraceae	Solirubrobacter
ASV362	Non-glacial	LacADNRUFtfc	0	Bacteria	Actinobacteriota	Thermoleophilia	Solirubrobacterales	Solirubrobacteraceae	Solirubrobacter
ASV362	Glacial	LacAmpRUFtf-a	0	Bacteria	Actinobacteriota	Thermoleophilia	Solirubrobacterales	Solirubrobacteraceae	Solirubrobacter
ASV362	Glacial	LacAmpRUFtf-b	0	Bacteria	Actinobacteriota	Thermoleophilia	Solirubrobacterales	Solirubrobacteraceae	Solirubrobacter
ASV362	Glacial	LacAmpRUFtf-c	0	Bacteria	Actinobacteriota	Thermoleophilia	Solirubrobacterales	Solirubrobacteraceae	Solirubrobacter
ASV368	Baie de la Table	BdT0-2	0	Bacteria	Cyanobacteriota	Cyanobacteriia	Cyanobacteriales	Nostocaceae	LE011-02
ASV368	Control	Ctr-tf-IIb	0	Bacteria	Cyanobacteriota	Cyanobacteriia	Cyanobacteriales	Nostocaceae	LE011-02
ASV368	Non-glacial	LacADNRUFtfa	0	Bacteria	Cyanobacteriota	Cyanobacteriia	Cyanobacteriales	Nostocaceae	LE011-02
ASV368	Non-glacial	LacADNRUFtfb	0	Bacteria	Cyanobacteriota	Cyanobacteriia	Cyanobacteriales	Nostocaceae	LE011-02
ASV368	Non-glacial	LacADNRUFtfc	0	Bacteria	Cyanobacteriota	Cyanobacteriia	Cyanobacteriales	Nostocaceae	LE011-02
ASV368	Glacial	LacAmpRUFtf-a	0	Bacteria	Cyanobacteriota	Cyanobacteriia	Cyanobacteriales	Nostocaceae	LE011-02
ASV368	Glacial	LacAmpRUFtf-b	0	Bacteria	Cyanobacteriota	Cyanobacteriia	Cyanobacteriales	Nostocaceae	LE011-02
ASV368	Glacial	LacAmpRUFtf-c	0	Bacteria	Cyanobacteriota	Cyanobacteriia	Cyanobacteriales	Nostocaceae	LE011-02
ASV369	Glacial	LacAmpRUFtf-c	0,000290487	Bacteria	Proteobacteria	Alphaproteobacteria	Acetobacterales	Acetobacteraceae	Roseomonas
ASV369	Baie de la Table	BdT0-2	0	Bacteria	Proteobacteria	Alphaproteobacteria	Acetobacterales	Acetobacteraceae	Roseomonas
ASV369	Control	Ctr-tf-IIb	0	Bacteria	Proteobacteria	Alphaproteobacteria	Acetobacterales	Acetobacteraceae	Roseomonas
ASV369	Non-glacial	LacADNRUFtfa	0	Bacteria	Proteobacteria	Alphaproteobacteria	Acetobacterales	Acetobacteraceae	Roseomonas
ASV369	Non-glacial	LacADNRUFtfb	0	Bacteria	Proteobacteria	Alphaproteobacteria	Acetobacterales	Acetobacteraceae	Roseomonas
ASV369	Non-glacial	LacADNRUFtfc	0	Bacteria	Proteobacteria	Alphaproteobacteria	Acetobacterales	Acetobacteraceae	Roseomonas
ASV369	Glacial	LacAmpRUFtf-a	0	Bacteria	Proteobacteria	Alphaproteobacteria	Acetobacterales	Acetobacteraceae	Roseomonas
ASV369	Glacial	LacAmpRUFtf-b	0	Bacteria	Proteobacteria	Alphaproteobacteria	Acetobacterales	Acetobacteraceae	Roseomonas
ASV37	Glacial	LacAmpRUFtf-c	0,10060518	Bacteria	Proteobacteria	Alphaproteobacteria	Rhizobiales	Beijerinckiaceae	Methylobacterium
ASV37	Glacial	LacAmpRUFtf-b	0,035093402	Bacteria	Proteobacteria	Alphaproteobacteria	Rhizobiales	Beijerinckiaceae	Methylobacterium
ASV37	Non-glacial	LacADNRUFtfb	0,03024236	Bacteria	Proteobacteria	Alphaproteobacteria	Rhizobiales	Beijerinckiaceae	Methylobacterium
ASV37	Non-glacial	LacADNRUFtfa	0,010531664	Bacteria	Proteobacteria	Alphaproteobacteria	Rhizobiales	Beijerinckiaceae	Methylobacterium
ASV37	Non-glacial	LacADNRUFtfc	0,00628031	Bacteria	Proteobacteria	Alphaproteobacteria	Rhizobiales	Beijerinckiaceae	Methylobacterium
ASV37	Glacial	LacAmpRUFtf-a	0,001607459	Bacteria	Proteobacteria	Alphaproteobacteria	Rhizobiales	Beijerinckiaceae	Methylobacterium
ASV37	Baie de la Table	BdT0-2	0	Bacteria	Proteobacteria	Alphaproteobacteria	Rhizobiales	Beijerinckiaceae	Methylobacterium
ASV37	Control	Ctr-tf-IIb	0	Bacteria	Proteobacteria	Alphaproteobacteria	Rhizobiales	Beijerinckiaceae	Methylobacterium

ASV371	Baie de la Table	BdT0-2	0,000273254	Bacteria	Proteobacteria	Gammaproteobacteria	Pseudomonadales	Cellvibrionaceae	Microbulbifer
ASV371	Control	Ctr-tf-IIb	0	Bacteria	Proteobacteria	Gammaproteobacteria	Pseudomonadales	Cellvibrionaceae	Microbulbifer
ASV371	Non-glacial	LacADNRUFtfa	0	Bacteria	Proteobacteria	Gammaproteobacteria	Pseudomonadales	Cellvibrionaceae	Microbulbifer
ASV371	Non-glacial	LacADNRUFtfb	0	Bacteria	Proteobacteria	Gammaproteobacteria	Pseudomonadales	Cellvibrionaceae	Microbulbifer
ASV371	Non-glacial	LacADNRUFtfc	0	Bacteria	Proteobacteria	Gammaproteobacteria	Pseudomonadales	Cellvibrionaceae	Microbulbifer
ASV371	Glacial	LacAmpRUFtf-a	0	Bacteria	Proteobacteria	Gammaproteobacteria	Pseudomonadales	Cellvibrionaceae	Microbulbifer
ASV371	Glacial	LacAmpRUFtf-b	0	Bacteria	Proteobacteria	Gammaproteobacteria	Pseudomonadales	Cellvibrionaceae	Microbulbifer
ASV371	Glacial	LacAmpRUFtf-c	0	Bacteria	Proteobacteria	Gammaproteobacteria	Pseudomonadales	Cellvibrionaceae	Microbulbifer
ASV373	Baie de la Table	BdT0-2	0	Bacteria	Acidobacteriota	Luteitaleia	Luteitaleales	Luteitaleaceae	Luteitalea
ASV373	Control	Ctr-tf-IIb	0	Bacteria	Acidobacteriota	Luteitaleia	Luteitaleales	Luteitaleaceae	Luteitalea
ASV373	Non-glacial	LacADNRUFtfa	0	Bacteria	Acidobacteriota	Luteitaleia	Luteitaleales	Luteitaleaceae	Luteitalea
ASV373	Non-glacial	LacADNRUFtfb	0	Bacteria	Acidobacteriota	Luteitaleia	Luteitaleales	Luteitaleaceae	Luteitalea
ASV373	Non-glacial	LacADNRUFtfc	0	Bacteria	Acidobacteriota	Luteitaleia	Luteitaleales	Luteitaleaceae	Luteitalea
ASV373	Glacial	LacAmpRUFtf-a	0	Bacteria	Acidobacteriota	Luteitaleia	Luteitaleales	Luteitaleaceae	Luteitalea
ASV373	Glacial	LacAmpRUFtf-b	0	Bacteria	Acidobacteriota	Luteitaleia	Luteitaleales	Luteitaleaceae	Luteitalea
ASV373	Glacial	LacAmpRUFtf-c	0	Bacteria	Acidobacteriota	Luteitaleia	Luteitaleales	Luteitaleaceae	Luteitalea
ASV375	Glacial	LacAmpRUFtf-c	0,000580973	Bacteria	Proteobacteria	Gammaproteobacteria	Enterobacterales	Shewanellaceae	Shewanella
ASV375	Glacial	LacAmpRUFtf-b	0,000431919	Bacteria	Proteobacteria	Gammaproteobacteria	Enterobacterales	Shewanellaceae	Shewanella
ASV375	Glacial	LacAmpRUFtf-a	0,000281305	Bacteria	Proteobacteria	Gammaproteobacteria	Enterobacterales	Shewanellaceae	Shewanella
ASV375	Non-glacial	LacADNRUFtfa	9,24E-05	Bacteria	Proteobacteria	Gammaproteobacteria	Enterobacterales	Shewanellaceae	Shewanella
ASV375	Baie de la Table	BdT0-2	0	Bacteria	Proteobacteria	Gammaproteobacteria	Enterobacterales	Shewanellaceae	Shewanella
ASV375	Control	Ctr-tf-IIb	0	Bacteria	Proteobacteria	Gammaproteobacteria	Enterobacterales	Shewanellaceae	Shewanella
ASV375	Non-glacial	LacADNRUFtfb	0	Bacteria	Proteobacteria	Gammaproteobacteria	Enterobacterales	Shewanellaceae	Shewanella
ASV375	Non-glacial	LacADNRUFtfc	0	Bacteria	Proteobacteria	Gammaproteobacteria	Enterobacterales	Shewanellaceae	Shewanella
ASV379	Glacial	LacAmpRUFtf-a	0,003255104	Bacteria	Proteobacteria	Gammaproteobacteria	Pseudomonadales	Nitrincolaceae	Neptuniibacter
ASV379	Glacial	LacAmpRUFtf-c	0,00043573	Bacteria	Proteobacteria	Gammaproteobacteria	Pseudomonadales	Nitrincolaceae	Neptuniibacter
ASV379	Glacial	LacAmpRUFtf-b	0,000323939	Bacteria	Proteobacteria	Gammaproteobacteria	Pseudomonadales	Nitrincolaceae	Neptuniibacter
ASV379	Baie de la Table	BdT0-2	0	Bacteria	Proteobacteria	Gammaproteobacteria	Pseudomonadales	Nitrincolaceae	Neptuniibacter
ASV379	Control	Ctr-tf-IIb	0	Bacteria	Proteobacteria	Gammaproteobacteria	Pseudomonadales	Nitrincolaceae	Neptuniibacter
ASV379	Non-glacial	LacADNRUFtfa	0	Bacteria	Proteobacteria	Gammaproteobacteria	Pseudomonadales	Nitrincolaceae	Neptuniibacter
ASV379	Non-glacial	LacADNRUFtfb	0	Bacteria	Proteobacteria	Gammaproteobacteria	Pseudomonadales	Nitrincolaceae	Neptuniibacter
ASV379	Non-glacial	LacADNRUFtfc	0	Bacteria	Proteobacteria	Gammaproteobacteria	Pseudomonadales	Nitrincolaceae	Neptuniibacter
ASV38	Baie de la Table	BdT0-2	0,033883485	Bacteria	Proteobacteria	Gammaproteobacteria	SAR86	D2472	D2472
ASV38	Non-glacial	LacADNRUFtfa	0,006882535	Bacteria	Proteobacteria	Gammaproteobacteria	SAR86	D2472	D2472

ASV38	Glacial	LacAmpRUFtf-b	0,004751107	Bacteria	Proteobacteria	Gammaproteobacteria	SAR86	D2472	D2472
ASV38	Non-glacial	LacADNRUFtfb	0,003140148	Bacteria	Proteobacteria	Gammaproteobacteria	SAR86	D2472	D2472
ASV38	Control	Ctr-tf-IIb	0,002219649	Bacteria	Proteobacteria	Gammaproteobacteria	SAR86	D2472	D2472
ASV38	Non-glacial	LacADNRUFtfc	0,001188167	Bacteria	Proteobacteria	Gammaproteobacteria	SAR86	D2472	D2472
ASV38	Glacial	LacAmpRUFtf-a	0,000924289	Bacteria	Proteobacteria	Gammaproteobacteria	SAR86	D2472	D2472
ASV38	Glacial	LacAmpRUFtf-c	0,000629388	Bacteria	Proteobacteria	Gammaproteobacteria	SAR86	D2472	D2472
ASV384	Baie de la Table	BdT0-2	0,000163952	Bacteria	Proteobacteria	Gammaproteobacteria	Woeseiales	Woeseiaceae	Woeseia
ASV384	Non-glacial	LacADNRUFtfa	9,24E-05	Bacteria	Proteobacteria	Gammaproteobacteria	Woeseiales	Woeseiaceae	Woeseia
ASV384	Control	Ctr-tf-IIb	0	Bacteria	Proteobacteria	Gammaproteobacteria	Woeseiales	Woeseiaceae	Woeseia
ASV384	Non-glacial	LacADNRUFtfb	0	Bacteria	Proteobacteria	Gammaproteobacteria	Woeseiales	Woeseiaceae	Woeseia
ASV384	Non-glacial	LacADNRUFtfc	0	Bacteria	Proteobacteria	Gammaproteobacteria	Woeseiales	Woeseiaceae	Woeseia
ASV384	Glacial	LacAmpRUFtf-a	0	Bacteria	Proteobacteria	Gammaproteobacteria	Woeseiales	Woeseiaceae	Woeseia
ASV384	Glacial	LacAmpRUFtf-b	0	Bacteria	Proteobacteria	Gammaproteobacteria	Woeseiales	Woeseiaceae	Woeseia
ASV384	Glacial	LacAmpRUFtf-c	0	Bacteria	Proteobacteria	Gammaproteobacteria	Woeseiales	Woeseiaceae	Woeseia
ASV385	Non-glacial	LacADNRUFtfb	0,000316122	Bacteria	Bacteroidota	Bacteroidia	Flavobacteriales	Cryomorphaceae	Coccinistipes
ASV385	Baie de la Table	BdT0-2	0	Bacteria	Bacteroidota	Bacteroidia	Flavobacteriales	Cryomorphaceae	Coccinistipes
ASV385	Control	Ctr-tf-IIb	0	Bacteria	Bacteroidota	Bacteroidia	Flavobacteriales	Cryomorphaceae	Coccinistipes
ASV385	Non-glacial	LacADNRUFtfa	0	Bacteria	Bacteroidota	Bacteroidia	Flavobacteriales	Cryomorphaceae	Coccinistipes
ASV385	Non-glacial	LacADNRUFtfc	0	Bacteria	Bacteroidota	Bacteroidia	Flavobacteriales	Cryomorphaceae	Coccinistipes
ASV385	Glacial	LacAmpRUFtf-a	0	Bacteria	Bacteroidota	Bacteroidia	Flavobacteriales	Cryomorphaceae	Coccinistipes
ASV385	Glacial	LacAmpRUFtf-b	0	Bacteria	Bacteroidota	Bacteroidia	Flavobacteriales	Cryomorphaceae	Coccinistipes
ASV385	Glacial	LacAmpRUFtf-c	0	Bacteria	Bacteroidota	Bacteroidia	Flavobacteriales	Cryomorphaceae	Coccinistipes
ASV389	Glacial	LacAmpRUFtf-c	0,003631082	Bacteria	Proteobacteria	Gammaproteobacteria	Betaproteobacteriales	Burkholderiaceae	Paucibacter
ASV389	Non-glacial	LacADNRUFtfb	0,000948367	Bacteria	Proteobacteria	Gammaproteobacteria	Betaproteobacteriales	Burkholderiaceae	Paucibacter
ASV389	Non-glacial	LacADNRUFtfa	0,00060049	Bacteria	Proteobacteria	Gammaproteobacteria	Betaproteobacteriales	Burkholderiaceae	Paucibacter
ASV389	Glacial	LacAmpRUFtf-a	0,000401865	Bacteria	Proteobacteria	Gammaproteobacteria	Betaproteobacteriales	Burkholderiaceae	Paucibacter
ASV389	Control	Ctr-tf-IIb	0,000192316	Bacteria	Proteobacteria	Gammaproteobacteria	Betaproteobacteriales	Burkholderiaceae	Paucibacter
ASV389	Baie de la Table	BdT0-2	0	Bacteria	Proteobacteria	Gammaproteobacteria	Betaproteobacteriales	Burkholderiaceae	Paucibacter
ASV389	Non-glacial	LacADNRUFtfc	0	Bacteria	Proteobacteria	Gammaproteobacteria	Betaproteobacteriales	Burkholderiaceae	Paucibacter
ASV389	Glacial	LacAmpRUFtf-b	0	Bacteria	Proteobacteria	Gammaproteobacteria	Betaproteobacteriales	Burkholderiaceae	Paucibacter
ASV4	Glacial	LacAmpRUFtf-c	0,001839748	Bacteria	Cyanobacteriota	Cyanobacteriia	Synechococcales_A	Cyanobiaceae	Cyanobium
ASV4	Glacial	LacAmpRUFtf-b	0,001295756	Bacteria	Cyanobacteriota	Cyanobacteriia	Synechococcales_A	Cyanobiaceae	Cyanobium
ASV4	Non-glacial	LacADNRUFtfa	0,000785256	Bacteria	Cyanobacteriota	Cyanobacteriia	Synechococcales_A	Cyanobiaceae	Cyanobium
ASV4	Non-glacial	LacADNRUFtfc	0,000606208	Bacteria	Cyanobacteriota	Cyanobacteriia	Synechococcales_A	Cyanobiaceae	Cyanobium

ASV4	Non-glacial	LacADNRUFtfb	0,000442571	Bacteria	Cyanobacteriota	Cyanobacteriia	Synechococcales_A	Cyanobiaceae	Cyanobium
ASV4	Control	Ctr-tf-IIb	0,000308507	Bacteria	Cyanobacteriota	Cyanobacteriia	Synechococcales_A	Cyanobiaceae	Cyanobium
ASV4	Baie de la Table	BdT0-2	0	Bacteria	Cyanobacteriota	Cyanobacteriia	Synechococcales_A	Cyanobiaceae	Cyanobium
ASV4	Glacial	LacAmpRUFtf-a	0	Bacteria	Cyanobacteriota	Cyanobacteriia	Synechococcales_A	Cyanobiaceae	Cyanobium
ASV400	Glacial	LacAmpRUFtf-a	0,002210256	Bacteria	Proteobacteria	Alphaproteobacteria	Rhodobacterales	Rhodobacteraceae	Nereida
ASV400	Baie de la Table	BdT0-2	0,000218603	Bacteria	Proteobacteria	Alphaproteobacteria	Rhodobacterales	Rhodobacteraceae	Nereida
ASV400	Non-glacial	LacADNRUFtfb	0,000126449	Bacteria	Proteobacteria	Alphaproteobacteria	Rhodobacterales	Rhodobacteraceae	Nereida
ASV400	Control	Ctr-tf-IIb	0	Bacteria	Proteobacteria	Alphaproteobacteria	Rhodobacterales	Rhodobacteraceae	Nereida
ASV400	Non-glacial	LacADNRUFtfa	0	Bacteria	Proteobacteria	Alphaproteobacteria	Rhodobacterales	Rhodobacteraceae	Nereida
ASV400	Non-glacial	LacADNRUFtfc	0	Bacteria	Proteobacteria	Alphaproteobacteria	Rhodobacterales	Rhodobacteraceae	Nereida
ASV400	Glacial	LacAmpRUFtf-b	0	Bacteria	Proteobacteria	Alphaproteobacteria	Rhodobacterales	Rhodobacteraceae	Nereida
ASV400	Glacial	LacAmpRUFtf-c	0	Bacteria	Proteobacteria	Alphaproteobacteria	Rhodobacterales	Rhodobacteraceae	Nereida
ASV402	Glacial	LacAmpRUFtf-c	0,006293876	Bacteria	Proteobacteria	Gammaproteobacteria	Pseudomonadales	Moraxellaceae	Enhydrobacter
ASV402	Non-glacial	LacADNRUFtfb	6,32E-05	Bacteria	Proteobacteria	Gammaproteobacteria	Pseudomonadales	Moraxellaceae	Enhydrobacter
ASV402	Baie de la Table	BdT0-2	0	Bacteria	Proteobacteria	Gammaproteobacteria	Pseudomonadales	Moraxellaceae	Enhydrobacter
ASV402	Control	Ctr-tf-IIb	0	Bacteria	Proteobacteria	Gammaproteobacteria	Pseudomonadales	Moraxellaceae	Enhydrobacter
ASV402	Non-glacial	LacADNRUFtfa	0	Bacteria	Proteobacteria	Gammaproteobacteria	Pseudomonadales	Moraxellaceae	Enhydrobacter
ASV402	Non-glacial	LacADNRUFtfc	0	Bacteria	Proteobacteria	Gammaproteobacteria	Pseudomonadales	Moraxellaceae	Enhydrobacter
ASV402	Glacial	LacAmpRUFtf-a	0	Bacteria	Proteobacteria	Gammaproteobacteria	Pseudomonadales	Moraxellaceae	Enhydrobacter
ASV402	Glacial	LacAmpRUFtf-b	0	Bacteria	Proteobacteria	Gammaproteobacteria	Pseudomonadales	Moraxellaceae	Enhydrobacter
ASV409	Glacial	LacAmpRUFtf-a	0,001486899	Bacteria	Bacteroidota	Bacteroidia	Flavobacteriales	Flavobacteriaceae	Muricauda
ASV409	Baie de la Table	BdT0-2	0	Bacteria	Bacteroidota	Bacteroidia	Flavobacteriales	Flavobacteriaceae	Muricauda
ASV409	Control	Ctr-tf-IIb	0	Bacteria	Bacteroidota	Bacteroidia	Flavobacteriales	Flavobacteriaceae	Muricauda
ASV409	Non-glacial	LacADNRUFtfa	0	Bacteria	Bacteroidota	Bacteroidia	Flavobacteriales	Flavobacteriaceae	Muricauda
ASV409	Non-glacial	LacADNRUFtfb	0	Bacteria	Bacteroidota	Bacteroidia	Flavobacteriales	Flavobacteriaceae	Muricauda
ASV409	Non-glacial	LacADNRUFtfc	0	Bacteria	Bacteroidota	Bacteroidia	Flavobacteriales	Flavobacteriaceae	Muricauda
ASV409	Glacial	LacAmpRUFtf-b	0	Bacteria	Bacteroidota	Bacteroidia	Flavobacteriales	Flavobacteriaceae	Muricauda
ASV409	Glacial	LacAmpRUFtf-c	0	Bacteria	Bacteroidota	Bacteroidia	Flavobacteriales	Flavobacteriaceae	Muricauda
ASV411	Glacial	LacAmpRUFtf-b	0,001079797	Bacteria	Actinobacteriota	Actinobacteria	Corynebacteriales	Corynebacteriaceae	Mycobacterium
ASV411	Non-glacial	LacADNRUFtfb	0,000126449	Bacteria	Actinobacteriota	Actinobacteria	Corynebacteriales	Corynebacteriaceae	Mycobacterium
ASV411	Control	Ctr-tf-IIb	6,81E-05	Bacteria	Actinobacteriota	Actinobacteria	Corynebacteriales	Corynebacteriaceae	Mycobacterium
ASV411	Baie de la Table	BdT0-2	0	Bacteria	Actinobacteriota	Actinobacteria	Corynebacteriales	Corynebacteriaceae	Mycobacterium
ASV411	Non-glacial	LacADNRUFtfa	0	Bacteria	Actinobacteriota	Actinobacteria	Corynebacteriales	Corynebacteriaceae	Mycobacterium
ASV411	Non-glacial	LacADNRUFtfc	0	Bacteria	Actinobacteriota	Actinobacteria	Corynebacteriales	Corynebacteriaceae	Mycobacterium

ASV411	Glacial	LacAmpRUFtf-a	0	Bacteria	Actinobacteriota	Actinobacteria	Corynebacteriales	Corynebacteriaceae	Mycobacterium
ASV411	Glacial	LacAmpRUFtf-c	0	Bacteria	Actinobacteriota	Actinobacteria	Corynebacteriales	Corynebacteriaceae	Mycobacterium
ASV416	Control	Ctr-tf-IIb	0,000172283	Bacteria	Proteobacteria	Alphaproteobacteria	Sphingomonadales	Sphingomonadaceae	Novosphingobium
ASV416	Baie de la Table	BdT0-2	0	Bacteria	Proteobacteria	Alphaproteobacteria	Sphingomonadales	Sphingomonadaceae	Novosphingobium
ASV416	Non-glacial	LacADNRUFtfa	0	Bacteria	Proteobacteria	Alphaproteobacteria	Sphingomonadales	Sphingomonadaceae	Novosphingobium
ASV416	Non-glacial	LacADNRUFtfb	0	Bacteria	Proteobacteria	Alphaproteobacteria	Sphingomonadales	Sphingomonadaceae	Novosphingobium
ASV416	Non-glacial	LacADNRUFtfc	0	Bacteria	Proteobacteria	Alphaproteobacteria	Sphingomonadales	Sphingomonadaceae	Novosphingobium
ASV416	Glacial	LacAmpRUFtf-a	0	Bacteria	Proteobacteria	Alphaproteobacteria	Sphingomonadales	Sphingomonadaceae	Novosphingobium
ASV416	Glacial	LacAmpRUFtf-b	0	Bacteria	Proteobacteria	Alphaproteobacteria	Sphingomonadales	Sphingomonadaceae	Novosphingobium
ASV416	Glacial	LacAmpRUFtf-c	0	Bacteria	Proteobacteria	Alphaproteobacteria	Sphingomonadales	Sphingomonadaceae	Novosphingobium
ASV42	Baie de la Table	BdT0-2	0,017652202	Bacteria	Proteobacteria	Gammaproteobacteria	Pseudomonadales	Porticoccaceae	HTCC2207
ASV42	Non-glacial	LacADNRUFtfa	0,009330685	Bacteria	Proteobacteria	Gammaproteobacteria	Pseudomonadales	Porticoccaceae	HTCC2207
ASV42	Non-glacial	LacADNRUFtfb	0,005943098	Bacteria	Proteobacteria	Gammaproteobacteria	Pseudomonadales	Porticoccaceae	HTCC2207
ASV42	Control	Ctr-tf-IIb	0,004972174	Bacteria	Proteobacteria	Gammaproteobacteria	Pseudomonadales	Porticoccaceae	HTCC2207
ASV42	Non-glacial	LacADNRUFtfc	0,002061106	Bacteria	Proteobacteria	Gammaproteobacteria	Pseudomonadales	Porticoccaceae	HTCC2207
ASV42	Glacial	LacAmpRUFtf-a	0,00136634	Bacteria	Proteobacteria	Gammaproteobacteria	Pseudomonadales	Porticoccaceae	HTCC2207
ASV42	Glacial	LacAmpRUFtf-b	0,001079797	Bacteria	Proteobacteria	Gammaproteobacteria	Pseudomonadales	Porticoccaceae	HTCC2207
ASV42	Glacial	LacAmpRUFtf-c	0	Bacteria	Proteobacteria	Gammaproteobacteria	Pseudomonadales	Porticoccaceae	HTCC2207
ASV424	Baie de la Table	BdT0-2	0	Bacteria	Proteobacteria	Alphaproteobacteria	UBA1301	UBA1301	UBA6038
ASV424	Control	Ctr-tf-IIb	0	Bacteria	Proteobacteria	Alphaproteobacteria	UBA1301	UBA1301	UBA6038
ASV424	Non-glacial	LacADNRUFtfa	0	Bacteria	Proteobacteria	Alphaproteobacteria	UBA1301	UBA1301	UBA6038
ASV424	Non-glacial	LacADNRUFtfb	0	Bacteria	Proteobacteria	Alphaproteobacteria	UBA1301	UBA1301	UBA6038
ASV424	Non-glacial	LacADNRUFtfc	0	Bacteria	Proteobacteria	Alphaproteobacteria	UBA1301	UBA1301	UBA6038
ASV424	Glacial	LacAmpRUFtf-a	0	Bacteria	Proteobacteria	Alphaproteobacteria	UBA1301	UBA1301	UBA6038
ASV424	Glacial	LacAmpRUFtf-b	0	Bacteria	Proteobacteria	Alphaproteobacteria	UBA1301	UBA1301	UBA6038
ASV424	Glacial	LacAmpRUFtf-c	0	Bacteria	Proteobacteria	Alphaproteobacteria	UBA1301	UBA1301	UBA6038
ASV435	Glacial	LacAmpRUFtf-a	0,001527086	Bacteria	Cyanobacteriota	Cyanobacteriia	Synechococcales_A	Cyanobiaceae	Synechococcus_C
ASV435	Baie de la Table	BdT0-2	0	Bacteria	Cyanobacteriota	Cyanobacteriia	Synechococcales_A	Cyanobiaceae	Synechococcus_C
ASV435	Control	Ctr-tf-IIb	0	Bacteria	Cyanobacteriota	Cyanobacteriia	Synechococcales_A	Cyanobiaceae	Synechococcus_C
ASV435	Non-glacial	LacADNRUFtfa	0	Bacteria	Cyanobacteriota	Cyanobacteriia	Synechococcales_A	Cyanobiaceae	Synechococcus_C
ASV435	Non-glacial	LacADNRUFtfb	0	Bacteria	Cyanobacteriota	Cyanobacteriia	Synechococcales_A	Cyanobiaceae	Synechococcus_C
ASV435	Non-glacial	LacADNRUFtfc	0	Bacteria	Cyanobacteriota	Cyanobacteriia	Synechococcales_A	Cyanobiaceae	Synechococcus_C
ASV435	Glacial	LacAmpRUFtf-b	0	Bacteria	Cyanobacteriota	Cyanobacteriia	Synechococcales_A	Cyanobiaceae	Synechococcus_C
ASV435	Glacial	LacAmpRUFtf-c	0	Bacteria	Cyanobacteriota	Cyanobacteriia	Synechococcales_A	Cyanobiaceae	Synechococcus_C

ASV436	Baie de la Table	BdT0-2	0	Bacteria	Proteobacteria	Gammaproteobacteria	Xanthomonadales	Xanthomonadaceae	Arenimonas
ASV436	Control	Ctr-tf-IIb	0	Bacteria	Proteobacteria	Gammaproteobacteria	Xanthomonadales	Xanthomonadaceae	Arenimonas
ASV436	Non-glacial	LacADNRUFtfa	0	Bacteria	Proteobacteria	Gammaproteobacteria	Xanthomonadales	Xanthomonadaceae	Arenimonas
ASV436	Non-glacial	LacADNRUFtfb	0	Bacteria	Proteobacteria	Gammaproteobacteria	Xanthomonadales	Xanthomonadaceae	Arenimonas
ASV436	Non-glacial	LacADNRUFtfc	0	Bacteria	Proteobacteria	Gammaproteobacteria	Xanthomonadales	Xanthomonadaceae	Arenimonas
ASV436	Glacial	LacAmpRUFtf-a	0	Bacteria	Proteobacteria	Gammaproteobacteria	Xanthomonadales	Xanthomonadaceae	Arenimonas
ASV436	Glacial	LacAmpRUFtf-b	0	Bacteria	Proteobacteria	Gammaproteobacteria	Xanthomonadales	Xanthomonadaceae	Arenimonas
ASV436	Glacial	LacAmpRUFtf-c	0	Bacteria	Proteobacteria	Gammaproteobacteria	Xanthomonadales	Xanthomonadaceae	Arenimonas
ASV442	Baie de la Table	BdT0-2	0,00071046	Bacteria	Marinisomatota	Marinisomatia	Marinisomatales	TCS55	UBA2126
ASV442	Glacial	LacAmpRUFtf-a	8,04E-05	Bacteria	Marinisomatota	Marinisomatia	Marinisomatales	TCS55	UBA2126
ASV442	Control	Ctr-tf-IIb	0	Bacteria	Marinisomatota	Marinisomatia	Marinisomatales	TCS55	UBA2126
ASV442	Non-glacial	LacADNRUFtfa	0	Bacteria	Marinisomatota	Marinisomatia	Marinisomatales	TCS55	UBA2126
ASV442	Non-glacial	LacADNRUFtfb	0	Bacteria	Marinisomatota	Marinisomatia	Marinisomatales	TCS55	UBA2126
ASV442	Non-glacial	LacADNRUFtfc	0	Bacteria	Marinisomatota	Marinisomatia	Marinisomatales	TCS55	UBA2126
ASV442	Glacial	LacAmpRUFtf-b	0	Bacteria	Marinisomatota	Marinisomatia	Marinisomatales	TCS55	UBA2126
ASV442	Glacial	LacAmpRUFtf-c	0	Bacteria	Marinisomatota	Marinisomatia	Marinisomatales	TCS55	UBA2126
ASV444	Glacial	LacAmpRUFtf-c	0,006293876	Bacteria	Proteobacteria	Alphaproteobacteria	Rhodobacterales	Rhodobacteraceae	Paracoccus
ASV444	Non-glacial	LacADNRUFtfb	0,000927292	Bacteria	Proteobacteria	Alphaproteobacteria	Rhodobacterales	Rhodobacteraceae	Paracoccus
ASV444	Non-glacial	LacADNRUFtfa	0,000230958	Bacteria	Proteobacteria	Alphaproteobacteria	Rhodobacterales	Rhodobacteraceae	Paracoccus
ASV444	Non-glacial	LacADNRUFtfc	7,27E-05	Bacteria	Proteobacteria	Alphaproteobacteria	Rhodobacterales	Rhodobacteraceae	Paracoccus
ASV444	Baie de la Table	BdT0-2	0	Bacteria	Proteobacteria	Alphaproteobacteria	Rhodobacterales	Rhodobacteraceae	Paracoccus
ASV444	Control	Ctr-tf-IIb	0	Bacteria	Proteobacteria	Alphaproteobacteria	Rhodobacterales	Rhodobacteraceae	Paracoccus
ASV444	Glacial	LacAmpRUFtf-a	0	Bacteria	Proteobacteria	Alphaproteobacteria	Rhodobacterales	Rhodobacteraceae	Paracoccus
ASV444	Glacial	LacAmpRUFtf-b	0	Bacteria	Proteobacteria	Alphaproteobacteria	Rhodobacterales	Rhodobacteraceae	Paracoccus
ASV451	Control	Ctr-tf-IIb	5,21E-05	Bacteria	Planctomycetota	Planctomycetes	Pirellulales	Pirellulaceae	GCA-2733575
ASV451	Baie de la Table	BdT0-2	0	Bacteria	Planctomycetota	Planctomycetes	Pirellulales	Pirellulaceae	GCA-2733575
ASV451	Non-glacial	LacADNRUFtfa	0	Bacteria	Planctomycetota	Planctomycetes	Pirellulales	Pirellulaceae	GCA-2733575
ASV451	Non-glacial	LacADNRUFtfb	0	Bacteria	Planctomycetota	Planctomycetes	Pirellulales	Pirellulaceae	GCA-2733575
ASV451	Non-glacial	LacADNRUFtfc	0	Bacteria	Planctomycetota	Planctomycetes	Pirellulales	Pirellulaceae	GCA-2733575
ASV451	Glacial	LacAmpRUFtf-a	0	Bacteria	Planctomycetota	Planctomycetes	Pirellulales	Pirellulaceae	GCA-2733575
ASV451	Glacial	LacAmpRUFtf-b	0	Bacteria	Planctomycetota	Planctomycetes	Pirellulales	Pirellulaceae	GCA-2733575
ASV451	Glacial	LacAmpRUFtf-c	0	Bacteria	Planctomycetota	Planctomycetes	Pirellulales	Pirellulaceae	GCA-2733575
ASV452	Non-glacial	LacADNRUFtfb	0,000273973	Bacteria	Proteobacteria	Alphaproteobacteria	Sphingomonadales	Sphingomonadaceae	Sphingobium
ASV452	Baie de la Table	BdT0-2	0	Bacteria	Proteobacteria	Alphaproteobacteria	Sphingomonadales	Sphingomonadaceae	Sphingobium

ASV452	Control	Ctr-tf-IIb	0	Bacteria	Proteobacteria	Alphaproteobacteria	Sphingomonadales	Sphingomonadaceae	Sphingobium
ASV452	Non-glacial	LacADNRUFtfa	0	Bacteria	Proteobacteria	Alphaproteobacteria	Sphingomonadales	Sphingomonadaceae	Sphingobium
ASV452	Non-glacial	LacADNRUFtfc	0	Bacteria	Proteobacteria	Alphaproteobacteria	Sphingomonadales	Sphingomonadaceae	Sphingobium
ASV452	Glacial	LacAmpRUFtf-a	0	Bacteria	Proteobacteria	Alphaproteobacteria	Sphingomonadales	Sphingomonadaceae	Sphingobium
ASV452	Glacial	LacAmpRUFtf-b	0	Bacteria	Proteobacteria	Alphaproteobacteria	Sphingomonadales	Sphingomonadaceae	Sphingobium
ASV452	Glacial	LacAmpRUFtf-c	0	Bacteria	Proteobacteria	Alphaproteobacteria	Sphingomonadales	Sphingomonadaceae	Sphingobium
ASV458	Baie de la Table	BdT0-2	0	Bacteria	Proteobacteria	Alphaproteobacteria	Sphingomonadales	Sphingomonadaceae	Sphingomonas_D
ASV458	Control	Ctr-tf-IIb	0	Bacteria	Proteobacteria	Alphaproteobacteria	Sphingomonadales	Sphingomonadaceae	Sphingomonas_D
ASV458	Non-glacial	LacADNRUFtfa	0	Bacteria	Proteobacteria	Alphaproteobacteria	Sphingomonadales	Sphingomonadaceae	Sphingomonas_D
ASV458	Non-glacial	LacADNRUFtfb	0	Bacteria	Proteobacteria	Alphaproteobacteria	Sphingomonadales	Sphingomonadaceae	Sphingomonas_D
ASV458	Non-glacial	LacADNRUFtfc	0	Bacteria	Proteobacteria	Alphaproteobacteria	Sphingomonadales	Sphingomonadaceae	Sphingomonas_D
ASV458	Glacial	LacAmpRUFtf-a	0	Bacteria	Proteobacteria	Alphaproteobacteria	Sphingomonadales	Sphingomonadaceae	Sphingomonas_D
ASV458	Glacial	LacAmpRUFtf-b	0	Bacteria	Proteobacteria	Alphaproteobacteria	Sphingomonadales	Sphingomonadaceae	Sphingomonas_D
ASV458	Glacial	LacAmpRUFtf-c	0	Bacteria	Proteobacteria	Alphaproteobacteria	Sphingomonadales	Sphingomonadaceae	Sphingomonas_D
ASV46	Control	Ctr-tf-IIb	0,007896983	Bacteria	Bacteroidota	Bacteroidia	Flavobacteriales	Flavobacteriaceae	Hel3-A1-48
ASV46	Baie de la Table	BdT0-2	0,006066237	Bacteria	Bacteroidota	Bacteroidia	Flavobacteriales	Flavobacteriaceae	Hel3-A1-48
ASV46	Non-glacial	LacADNRUFtfa	0,005034875	Bacteria	Bacteroidota	Bacteroidia	Flavobacteriales	Flavobacteriaceae	Hel3-A1-48
ASV46	Glacial	LacAmpRUFtf-b	0,004751107	Bacteria	Bacteroidota	Bacteroidia	Flavobacteriales	Flavobacteriaceae	Hel3-A1-48
ASV46	Non-glacial	LacADNRUFtfb	0,004299262	Bacteria	Bacteroidota	Bacteroidia	Flavobacteriales	Flavobacteriaceae	Hel3-A1-48
ASV46	Non-glacial	LacADNRUFtfc	0,002643065	Bacteria	Bacteroidota	Bacteroidia	Flavobacteriales	Flavobacteriaceae	Hel3-A1-48
ASV46	Glacial	LacAmpRUFtf-a	0,000964475	Bacteria	Bacteroidota	Bacteroidia	Flavobacteriales	Flavobacteriaceae	Hel3-A1-48
ASV46	Glacial	LacAmpRUFtf-c	0	Bacteria	Bacteroidota	Bacteroidia	Flavobacteriales	Flavobacteriaceae	Hel3-A1-48
ASV461	Non-glacial	LacADNRUFtfb	0,000273973	Bacteria	Firmicutes	Bacilli	Bacillales	Bacillaceae_B	Bacillus_F
ASV461	Baie de la Table	BdT0-2	0	Bacteria	Firmicutes	Bacilli	Bacillales	Bacillaceae_B	Bacillus_F
ASV461	Control	Ctr-tf-IIb	0	Bacteria	Firmicutes	Bacilli	Bacillales	Bacillaceae_B	Bacillus_F
ASV461	Non-glacial	LacADNRUFtfa	0	Bacteria	Firmicutes	Bacilli	Bacillales	Bacillaceae_B	Bacillus_F
ASV461	Non-glacial	LacADNRUFtfc	0	Bacteria	Firmicutes	Bacilli	Bacillales	Bacillaceae_B	Bacillus_F
ASV461	Glacial	LacAmpRUFtf-a	0	Bacteria	Firmicutes	Bacilli	Bacillales	Bacillaceae_B	Bacillus_F
ASV461	Glacial	LacAmpRUFtf-b	0	Bacteria	Firmicutes	Bacilli	Bacillales	Bacillaceae_B	Bacillus_F
ASV461	Glacial	LacAmpRUFtf-c	0	Bacteria	Firmicutes	Bacilli	Bacillales	Bacillaceae_B	Bacillus_F
ASV463	Baie de la Table	BdT0-2	0	Bacteria	Proteobacteria	Alphaproteobacteria	Rhizobiales	Beijerinckiaceae	Methylocapsa
ASV463	Control	Ctr-tf-IIb	0	Bacteria	Proteobacteria	Alphaproteobacteria	Rhizobiales	Beijerinckiaceae	Methylocapsa
ASV463	Non-glacial	LacADNRUFtfa	0	Bacteria	Proteobacteria	Alphaproteobacteria	Rhizobiales	Beijerinckiaceae	Methylocapsa
ASV463	Non-glacial	LacADNRUFtfb	0	Bacteria	Proteobacteria	Alphaproteobacteria	Rhizobiales	Beijerinckiaceae	Methylocapsa

ASV463	Non-glacial	LacADNRUFtfc	0	Bacteria	Proteobacteria	Alphaproteobacteria	Rhizobiales	Beijerinckiaceae	Methylocapsa
ASV463	Glacial	LacAmpRUFtf-a	0	Bacteria	Proteobacteria	Alphaproteobacteria	Rhizobiales	Beijerinckiaceae	Methylocapsa
ASV463	Glacial	LacAmpRUFtf-b	0	Bacteria	Proteobacteria	Alphaproteobacteria	Rhizobiales	Beijerinckiaceae	Methylocapsa
ASV463	Glacial	LacAmpRUFtf-c	0	Bacteria	Proteobacteria	Alphaproteobacteria	Rhizobiales	Beijerinckiaceae	Methylocapsa
ASV467	Baie de la Table	BdT0-2	0	Bacteria	Proteobacteria	Gammaproteobacteria	Betaproteobacteriales	Gallionellaceae	39-52-133
ASV467	Control	Ctr-tf-IIb	0	Bacteria	Proteobacteria	Gammaproteobacteria	Betaproteobacteriales	Gallionellaceae	39-52-133
ASV467	Non-glacial	LacADNRUFtfa	0	Bacteria	Proteobacteria	Gammaproteobacteria	Betaproteobacteriales	Gallionellaceae	39-52-133
ASV467	Non-glacial	LacADNRUFtfb	0	Bacteria	Proteobacteria	Gammaproteobacteria	Betaproteobacteriales	Gallionellaceae	39-52-133
ASV467	Non-glacial	LacADNRUFtfc	0	Bacteria	Proteobacteria	Gammaproteobacteria	Betaproteobacteriales	Gallionellaceae	39-52-133
ASV467	Glacial	LacAmpRUFtf-a	0	Bacteria	Proteobacteria	Gammaproteobacteria	Betaproteobacteriales	Gallionellaceae	39-52-133
ASV467	Glacial	LacAmpRUFtf-b	0	Bacteria	Proteobacteria	Gammaproteobacteria	Betaproteobacteriales	Gallionellaceae	39-52-133
ASV467	Glacial	LacAmpRUFtf-c	0	Bacteria	Proteobacteria	Gammaproteobacteria	Betaproteobacteriales	Gallionellaceae	39-52-133
ASV469	Glacial	LacAmpRUFtf-c	0,00251755	Bacteria	Actinobacteriota	Actinobacteria	Corynebacteriales	Corynebacteriaceae	Lawsonella
ASV469	Glacial	LacAmpRUFtf-b	0,001187777	Bacteria	Actinobacteriota	Actinobacteria	Corynebacteriales	Corynebacteriaceae	Lawsonella
ASV469	Non-glacial	LacADNRUFtfb	0,000231823	Bacteria	Actinobacteriota	Actinobacteria	Corynebacteriales	Corynebacteriaceae	Lawsonella
ASV469	Baie de la Table	BdT0-2	0	Bacteria	Actinobacteriota	Actinobacteria	Corynebacteriales	Corynebacteriaceae	Lawsonella
ASV469	Control	Ctr-tf-IIb	0	Bacteria	Actinobacteriota	Actinobacteria	Corynebacteriales	Corynebacteriaceae	Lawsonella
ASV469	Non-glacial	LacADNRUFtfa	0	Bacteria	Actinobacteriota	Actinobacteria	Corynebacteriales	Corynebacteriaceae	Lawsonella
ASV469	Non-glacial	LacADNRUFtfc	0	Bacteria	Actinobacteriota	Actinobacteria	Corynebacteriales	Corynebacteriaceae	Lawsonella
ASV469	Glacial	LacAmpRUFtf-a	0	Bacteria	Actinobacteriota	Actinobacteria	Corynebacteriales	Corynebacteriaceae	Lawsonella
ASV47	Baie de la Table	BdT0-2	0	Bacteria	Firmicutes	Bacilli	Bacillales	Bacillaceae	Bacillus
ASV47	Control	Ctr-tf-IIb	0	Bacteria	Firmicutes	Bacilli	Bacillales	Bacillaceae	Bacillus
ASV47	Non-glacial	LacADNRUFtfa	0	Bacteria	Firmicutes	Bacilli	Bacillales	Bacillaceae	Bacillus
ASV47	Non-glacial	LacADNRUFtfb	0	Bacteria	Firmicutes	Bacilli	Bacillales	Bacillaceae	Bacillus
ASV47	Non-glacial	LacADNRUFtfc	0	Bacteria	Firmicutes	Bacilli	Bacillales	Bacillaceae	Bacillus
ASV47	Glacial	LacAmpRUFtf-a	0	Bacteria	Firmicutes	Bacilli	Bacillales	Bacillaceae	Bacillus
ASV47	Glacial	LacAmpRUFtf-b	0	Bacteria	Firmicutes	Bacilli	Bacillales	Bacillaceae	Bacillus
ASV47	Glacial	LacAmpRUFtf-c	0	Bacteria	Firmicutes	Bacilli	Bacillales	Bacillaceae	Bacillus
ASV470	Baie de la Table	BdT0-2	0	Bacteria	Proteobacteria	Gammaproteobacteria	Legionellales	Legionellaceae	Legionella
ASV470	Control	Ctr-tf-IIb	0	Bacteria	Proteobacteria	Gammaproteobacteria	Legionellales	Legionellaceae	Legionella
ASV470	Non-glacial	LacADNRUFtfa	0	Bacteria	Proteobacteria	Gammaproteobacteria	Legionellales	Legionellaceae	Legionella
ASV470	Non-glacial	LacADNRUFtfb	0	Bacteria	Proteobacteria	Gammaproteobacteria	Legionellales	Legionellaceae	Legionella
ASV470	Non-glacial	LacADNRUFtfc	0	Bacteria	Proteobacteria	Gammaproteobacteria	Legionellales	Legionellaceae	Legionella
ASV470	Glacial	LacAmpRUFtf-a	0	Bacteria	Proteobacteria	Gammaproteobacteria	Legionellales	Legionellaceae	Legionella

ASV470	Glacial	LacAmpRUFtf-b	0	Bacteria	Proteobacteria	Gammaproteobacteria	Legionellales	Legionellaceae	Legionella
ASV470	Glacial	LacAmpRUFtf-c	0	Bacteria	Proteobacteria	Gammaproteobacteria	Legionellales	Legionellaceae	Legionella
ASV476	Non-glacial	LacADNRUFtfa	9,24E-05	Bacteria	Proteobacteria	Gammaproteobacteria	SAR86	SAR86	AEGEAN-183
ASV476	Baie de la Table	BdT0-2	0	Bacteria	Proteobacteria	Gammaproteobacteria	SAR86	SAR86	AEGEAN-183
ASV476	Control	Ctr-tf-IIb	0	Bacteria	Proteobacteria	Gammaproteobacteria	SAR86	SAR86	AEGEAN-183
ASV476	Non-glacial	LacADNRUFtfb	0	Bacteria	Proteobacteria	Gammaproteobacteria	SAR86	SAR86	AEGEAN-183
ASV476	Non-glacial	LacADNRUFtfc	0	Bacteria	Proteobacteria	Gammaproteobacteria	SAR86	SAR86	AEGEAN-183
ASV476	Glacial	LacAmpRUFtf-a	0	Bacteria	Proteobacteria	Gammaproteobacteria	SAR86	SAR86	AEGEAN-183
ASV476	Glacial	LacAmpRUFtf-b	0	Bacteria	Proteobacteria	Gammaproteobacteria	SAR86	SAR86	AEGEAN-183
ASV476	Glacial	LacAmpRUFtf-c	0	Bacteria	Proteobacteria	Gammaproteobacteria	SAR86	SAR86	AEGEAN-183
ASV48	Control	Ctr-tf-IIb	0,016094459	Bacteria	Proteobacteria	Alphaproteobacteria	Rhodobacterales	Rhodobacteraceae	Octadecabacter
ASV48	Non-glacial	LacADNRUFtfc	0,007129001	Bacteria	Proteobacteria	Alphaproteobacteria	Rhodobacterales	Rhodobacteraceae	Octadecabacter
ASV48	Non-glacial	LacADNRUFtfb	0,003203372	Bacteria	Proteobacteria	Alphaproteobacteria	Rhodobacterales	Rhodobacteraceae	Octadecabacter
ASV48	Glacial	LacAmpRUFtf-b	0,002915452	Bacteria	Proteobacteria	Alphaproteobacteria	Rhodobacterales	Rhodobacteraceae	Octadecabacter
ASV48	Glacial	LacAmpRUFtf-a	0,002813053	Bacteria	Proteobacteria	Alphaproteobacteria	Rhodobacterales	Rhodobacteraceae	Octadecabacter
ASV48	Non-glacial	LacADNRUFtfa	0,002263384	Bacteria	Proteobacteria	Alphaproteobacteria	Rhodobacterales	Rhodobacteraceae	Octadecabacter
ASV48	Baie de la Table	BdT0-2	0,000601159	Bacteria	Proteobacteria	Alphaproteobacteria	Rhodobacterales	Rhodobacteraceae	Octadecabacter
ASV48	Glacial	LacAmpRUFtf-c	0	Bacteria	Proteobacteria	Alphaproteobacteria	Rhodobacterales	Rhodobacteraceae	Octadecabacter
ASV494	Glacial	LacAmpRUFtf-a	0,00124578	Bacteria	Proteobacteria	Alphaproteobacteria	Rhodobacterales	Rhodobacteraceae	Roseovarius
ASV494	Baie de la Table	BdT0-2	0	Bacteria	Proteobacteria	Alphaproteobacteria	Rhodobacterales	Rhodobacteraceae	Roseovarius
ASV494	Control	Ctr-tf-IIb	0	Bacteria	Proteobacteria	Alphaproteobacteria	Rhodobacterales	Rhodobacteraceae	Roseovarius
ASV494	Non-glacial	LacADNRUFtfa	0	Bacteria	Proteobacteria	Alphaproteobacteria	Rhodobacterales	Rhodobacteraceae	Roseovarius
ASV494	Non-glacial	LacADNRUFtfb	0	Bacteria	Proteobacteria	Alphaproteobacteria	Rhodobacterales	Rhodobacteraceae	Roseovarius
ASV494	Non-glacial	LacADNRUFtfc	0	Bacteria	Proteobacteria	Alphaproteobacteria	Rhodobacterales	Rhodobacteraceae	Roseovarius
ASV494	Glacial	LacAmpRUFtf-b	0	Bacteria	Proteobacteria	Alphaproteobacteria	Rhodobacterales	Rhodobacteraceae	Roseovarius
ASV494	Glacial	LacAmpRUFtf-c	0	Bacteria	Proteobacteria	Alphaproteobacteria	Rhodobacterales	Rhodobacteraceae	Roseovarius
ASV5	Glacial	LacAmpRUFtf-a	0,652949687	Bacteria	Proteobacteria	Gammaproteobacteria	Enterobacterales	Vibrionaceae	Vibrio
ASV5	Glacial	LacAmpRUFtf-b	0,141777346	Bacteria	Proteobacteria	Gammaproteobacteria	Enterobacterales	Vibrionaceae	Vibrio
ASV5	Glacial	LacAmpRUFtf-c	0,072185911	Bacteria	Proteobacteria	Gammaproteobacteria	Enterobacterales	Vibrionaceae	Vibrio
ASV5	Non-glacial	LacADNRUFtfc	0,00943259	Bacteria	Proteobacteria	Gammaproteobacteria	Enterobacterales	Vibrionaceae	Vibrio
ASV5	Non-glacial	LacADNRUFtfa	0,002910065	Bacteria	Proteobacteria	Gammaproteobacteria	Enterobacterales	Vibrionaceae	Vibrio
ASV5	Non-glacial	LacADNRUFtfb	0,00056902	Bacteria	Proteobacteria	Gammaproteobacteria	Enterobacterales	Vibrionaceae	Vibrio
ASV5	Control	Ctr-tf-IIb	0,000108178	Bacteria	Proteobacteria	Gammaproteobacteria	Enterobacterales	Vibrionaceae	Vibrio
ASV5	Baie de la Table	BdT0-2	0	Bacteria	Proteobacteria	Gammaproteobacteria	Enterobacterales	Vibrionaceae	Vibrio

ASV507	Baie de la Table	BdT0-2	0	Bacteria	Actinobacteriota	Actinobacteria	Actinomycetales	Microbacteriaceae	Cryobacterium
ASV507	Control	Ctr-tf-IIb	0	Bacteria	Actinobacteriota	Actinobacteria	Actinomycetales	Microbacteriaceae	Cryobacterium
ASV507	Non-glacial	LacADNRUFtfa	0	Bacteria	Actinobacteriota	Actinobacteria	Actinomycetales	Microbacteriaceae	Cryobacterium
ASV507	Non-glacial	LacADNRUFtfb	0	Bacteria	Actinobacteriota	Actinobacteria	Actinomycetales	Microbacteriaceae	Cryobacterium
ASV507	Non-glacial	LacADNRUFtfc	0	Bacteria	Actinobacteriota	Actinobacteria	Actinomycetales	Microbacteriaceae	Cryobacterium
ASV507	Glacial	LacAmpRUFtf-a	0	Bacteria	Actinobacteriota	Actinobacteria	Actinomycetales	Microbacteriaceae	Cryobacterium
ASV507	Glacial	LacAmpRUFtf-b	0	Bacteria	Actinobacteriota	Actinobacteria	Actinomycetales	Microbacteriaceae	Cryobacterium
ASV507	Glacial	LacAmpRUFtf-c	0	Bacteria	Actinobacteriota	Actinobacteria	Actinomycetales	Microbacteriaceae	Cryobacterium
ASV514	Glacial	LacAmpRUFtf-c	0,00338901	Bacteria	Firmicutes_A	Clostridia	Tissierellales	Helcococcaceae	Finegoldia
ASV514	Non-glacial	LacADNRUFtfb	0,000231823	Bacteria	Firmicutes_A	Clostridia	Tissierellales	Helcococcaceae	Finegoldia
ASV514	Baie de la Table	BdT0-2	0	Bacteria	Firmicutes_A	Clostridia	Tissierellales	Helcococcaceae	Finegoldia
ASV514	Control	Ctr-tf-IIb	0	Bacteria	Firmicutes_A	Clostridia	Tissierellales	Helcococcaceae	Finegoldia
ASV514	Non-glacial	LacADNRUFtfa	0	Bacteria	Firmicutes_A	Clostridia	Tissierellales	Helcococcaceae	Finegoldia
ASV514	Non-glacial	LacADNRUFtfc	0	Bacteria	Firmicutes_A	Clostridia	Tissierellales	Helcococcaceae	Finegoldia
ASV514	Glacial	LacAmpRUFtf-a	0	Bacteria	Firmicutes_A	Clostridia	Tissierellales	Helcococcaceae	Finegoldia
ASV514	Glacial	LacAmpRUFtf-b	0	Bacteria	Firmicutes_A	Clostridia	Tissierellales	Helcococcaceae	Finegoldia
ASV516	Glacial	LacAmpRUFtf-a	0,003255104	Bacteria	Proteobacteria	Alphaproteobacteria	Puniceispirillales	Puniceispirillaceae	Puniceispirillum
ASV516	Glacial	LacAmpRUFtf-b	0,000863838	Bacteria	Proteobacteria	Alphaproteobacteria	Puniceispirillales	Puniceispirillaceae	Puniceispirillum
ASV516	Control	Ctr-tf-IIb	0,000260428	Bacteria	Proteobacteria	Alphaproteobacteria	Puniceispirillales	Puniceispirillaceae	Puniceispirillum
ASV516	Baie de la Table	BdT0-2	0,000218603	Bacteria	Proteobacteria	Alphaproteobacteria	Puniceispirillales	Puniceispirillaceae	Puniceispirillum
ASV516	Non-glacial	LacADNRUFtfa	0	Bacteria	Proteobacteria	Alphaproteobacteria	Puniceispirillales	Puniceispirillaceae	Puniceispirillum
ASV516	Non-glacial	LacADNRUFtfb	0	Bacteria	Proteobacteria	Alphaproteobacteria	Puniceispirillales	Puniceispirillaceae	Puniceispirillum
ASV516	Non-glacial	LacADNRUFtfc	0	Bacteria	Proteobacteria	Alphaproteobacteria	Puniceispirillales	Puniceispirillaceae	Puniceispirillum
ASV516	Glacial	LacAmpRUFtf-c	0	Bacteria	Proteobacteria	Alphaproteobacteria	Puniceispirillales	Puniceispirillaceae	Puniceispirillum
ASV52	Control	Ctr-tf-IIb	0,020645942	Bacteria	Bacteroidota	Bacteroidia	Flavobacteriales	Flavobacteriaceae	Altibacter
ASV52	Glacial	LacAmpRUFtf-b	0,012633625	Bacteria	Bacteroidota	Bacteroidia	Flavobacteriales	Flavobacteriaceae	Altibacter
ASV52	Non-glacial	LacADNRUFtfc	0,00657129	Bacteria	Bacteroidota	Bacteroidia	Flavobacteriales	Flavobacteriaceae	Altibacter
ASV52	Non-glacial	LacADNRUFtfb	0,00625922	Bacteria	Bacteroidota	Bacteroidia	Flavobacteriales	Flavobacteriaceae	Altibacter
ASV52	Non-glacial	LacADNRUFtfa	0,005589173	Bacteria	Bacteroidota	Bacteroidia	Flavobacteriales	Flavobacteriaceae	Altibacter
ASV52	Baie de la Table	BdT0-2	0,002623238	Bacteria	Bacteroidota	Bacteroidia	Flavobacteriales	Flavobacteriaceae	Altibacter
ASV52	Glacial	LacAmpRUFtf-a	0,002371001	Bacteria	Bacteroidota	Bacteroidia	Flavobacteriales	Flavobacteriaceae	Altibacter
ASV52	Glacial	LacAmpRUFtf-c	0	Bacteria	Bacteroidota	Bacteroidia	Flavobacteriales	Flavobacteriaceae	Altibacter
ASV521	Glacial	LacAmpRUFtf-c	0,001452433	Bacteria	Proteobacteria	Gammaproteobacteria	Xanthomonadales	Xanthomonadaceae	Stenotrophomonas
ASV521	Baie de la Table	BdT0-2	0	Bacteria	Proteobacteria	Gammaproteobacteria	Xanthomonadales	Xanthomonadaceae	Stenotrophomonas

ASV521	Control	Ctr-tf-IIb	0	Bacteria	Proteobacteria	Gammaproteobacteria	Xanthomonadales	Xanthomonadaceae	Stenotrophomonas
ASV521	Non-glacial	LacADNRUFtfa	0	Bacteria	Proteobacteria	Gammaproteobacteria	Xanthomonadales	Xanthomonadaceae	Stenotrophomonas
ASV521	Non-glacial	LacADNRUFtfb	0	Bacteria	Proteobacteria	Gammaproteobacteria	Xanthomonadales	Xanthomonadaceae	Stenotrophomonas
ASV521	Non-glacial	LacADNRUFtfc	0	Bacteria	Proteobacteria	Gammaproteobacteria	Xanthomonadales	Xanthomonadaceae	Stenotrophomonas
ASV521	Glacial	LacAmpRUFtf-a	0	Bacteria	Proteobacteria	Gammaproteobacteria	Xanthomonadales	Xanthomonadaceae	Stenotrophomonas
ASV521	Glacial	LacAmpRUFtf-b	0	Bacteria	Proteobacteria	Gammaproteobacteria	Xanthomonadales	Xanthomonadaceae	Stenotrophomonas
ASV523	Baie de la Table	BdT0-2	0	Archaea	Halobacterota	Halobacteria	Halobacteriales	Halococcaceae	Halococcus
ASV523	Control	Ctr-tf-IIb	0	Archaea	Halobacterota	Halobacteria	Halobacteriales	Halococcaceae	Halococcus
ASV523	Non-glacial	LacADNRUFtfa	0	Archaea	Halobacterota	Halobacteria	Halobacteriales	Halococcaceae	Halococcus
ASV523	Non-glacial	LacADNRUFtfb	0	Archaea	Halobacterota	Halobacteria	Halobacteriales	Halococcaceae	Halococcus
ASV523	Non-glacial	LacADNRUFtfc	0	Archaea	Halobacterota	Halobacteria	Halobacteriales	Halococcaceae	Halococcus
ASV523	Glacial	LacAmpRUFtf-a	0	Archaea	Halobacterota	Halobacteria	Halobacteriales	Halococcaceae	Halococcus
ASV523	Glacial	LacAmpRUFtf-b	0	Archaea	Halobacterota	Halobacteria	Halobacteriales	Halococcaceae	Halococcus
ASV523	Glacial	LacAmpRUFtf-c	0	Archaea	Halobacterota	Halobacteria	Halobacteriales	Halococcaceae	Halococcus
ASV525	Glacial	LacAmpRUFtf-a	0,00068317	Bacteria	Proteobacteria	Alphaproteobacteria	Rhodobacterales	Rhodobacteraceae	HIMB11
ASV525	Glacial	LacAmpRUFtf-c	0,000677802	Bacteria	Proteobacteria	Alphaproteobacteria	Rhodobacterales	Rhodobacteraceae	HIMB11
ASV525	Baie de la Table	BdT0-2	0	Bacteria	Proteobacteria	Alphaproteobacteria	Rhodobacterales	Rhodobacteraceae	HIMB11
ASV525	Control	Ctr-tf-IIb	0	Bacteria	Proteobacteria	Alphaproteobacteria	Rhodobacterales	Rhodobacteraceae	HIMB11
ASV525	Non-glacial	LacADNRUFtfa	0	Bacteria	Proteobacteria	Alphaproteobacteria	Rhodobacterales	Rhodobacteraceae	HIMB11
ASV525	Non-glacial	LacADNRUFtfb	0	Bacteria	Proteobacteria	Alphaproteobacteria	Rhodobacterales	Rhodobacteraceae	HIMB11
ASV525	Non-glacial	LacADNRUFtfc	0	Bacteria	Proteobacteria	Alphaproteobacteria	Rhodobacterales	Rhodobacteraceae	HIMB11
ASV525	Glacial	LacAmpRUFtf-b	0	Bacteria	Proteobacteria	Alphaproteobacteria	Rhodobacterales	Rhodobacteraceae	HIMB11
ASV535	Glacial	LacAmpRUFtf-c	0,000484144	Bacteria	Proteobacteria	Gammaproteobacteria	Betaproteobacteriales	Burkholderiaceae	Variovorax
ASV535	Baie de la Table	BdT0-2	0	Bacteria	Proteobacteria	Gammaproteobacteria	Betaproteobacteriales	Burkholderiaceae	Variovorax
ASV535	Control	Ctr-tf-IIb	0	Bacteria	Proteobacteria	Gammaproteobacteria	Betaproteobacteriales	Burkholderiaceae	Variovorax
ASV535	Non-glacial	LacADNRUFtfa	0	Bacteria	Proteobacteria	Gammaproteobacteria	Betaproteobacteriales	Burkholderiaceae	Variovorax
ASV535	Non-glacial	LacADNRUFtfb	0	Bacteria	Proteobacteria	Gammaproteobacteria	Betaproteobacteriales	Burkholderiaceae	Variovorax
ASV535	Non-glacial	LacADNRUFtfc	0	Bacteria	Proteobacteria	Gammaproteobacteria	Betaproteobacteriales	Burkholderiaceae	Variovorax
ASV535	Glacial	LacAmpRUFtf-a	0	Bacteria	Proteobacteria	Gammaproteobacteria	Betaproteobacteriales	Burkholderiaceae	Variovorax
ASV535	Glacial	LacAmpRUFtf-b	0	Bacteria	Proteobacteria	Gammaproteobacteria	Betaproteobacteriales	Burkholderiaceae	Variovorax
ASV539	Glacial	LacAmpRUFtf-b	0,001835655	Bacteria	Proteobacteria	Gammaproteobacteria	Pseudomonadales	Moraxellaceae	Psychrobacter
ASV539	Glacial	LacAmpRUFtf-c	0,000338901	Bacteria	Proteobacteria	Gammaproteobacteria	Pseudomonadales	Moraxellaceae	Psychrobacter
ASV539	Baie de la Table	BdT0-2	0	Bacteria	Proteobacteria	Gammaproteobacteria	Pseudomonadales	Moraxellaceae	Psychrobacter
ASV539	Control	Ctr-tf-IIb	0	Bacteria	Proteobacteria	Gammaproteobacteria	Pseudomonadales	Moraxellaceae	Psychrobacter

ASV539	Non-glacial	LacADNRUFtfa	0	Bacteria	Proteobacteria	Gammaproteobacteria	Pseudomonadales	Moraxellaceae	Psychrobacter
ASV539	Non-glacial	LacADNRUFtfb	0	Bacteria	Proteobacteria	Gammaproteobacteria	Pseudomonadales	Moraxellaceae	Psychrobacter
ASV539	Non-glacial	LacADNRUFtfc	0	Bacteria	Proteobacteria	Gammaproteobacteria	Pseudomonadales	Moraxellaceae	Psychrobacter
ASV539	Glacial	LacAmpRUFtf-a	0	Bacteria	Proteobacteria	Gammaproteobacteria	Pseudomonadales	Moraxellaceae	Psychrobacter
ASV544	Baie de la Table	BdT0-2	0	Bacteria	Bacteroidota	Bacteroidia	Flavobacteriales	Crocinitomicaceae	UBA952
ASV544	Control	Ctr-tf-IIb	0	Bacteria	Bacteroidota	Bacteroidia	Flavobacteriales	Crocinitomicaceae	UBA952
ASV544	Non-glacial	LacADNRUFtfa	0	Bacteria	Bacteroidota	Bacteroidia	Flavobacteriales	Crocinitomicaceae	UBA952
ASV544	Non-glacial	LacADNRUFtfb	0	Bacteria	Bacteroidota	Bacteroidia	Flavobacteriales	Crocinitomicaceae	UBA952
ASV544	Non-glacial	LacADNRUFtfc	0	Bacteria	Bacteroidota	Bacteroidia	Flavobacteriales	Crocinitomicaceae	UBA952
ASV544	Glacial	LacAmpRUFtf-a	0	Bacteria	Bacteroidota	Bacteroidia	Flavobacteriales	Crocinitomicaceae	UBA952
ASV544	Glacial	LacAmpRUFtf-b	0	Bacteria	Bacteroidota	Bacteroidia	Flavobacteriales	Crocinitomicaceae	UBA952
ASV544	Glacial	LacAmpRUFtf-c	0	Bacteria	Bacteroidota	Bacteroidia	Flavobacteriales	Crocinitomicaceae	UBA952
ASV55	Non-glacial	LacADNRUFtfa	0,004988683	Bacteria	Proteobacteria	Gammaproteobacteria	Betaproteobacteriales	Burkholderiaceae	Polaromonas
ASV55	Non-glacial	LacADNRUFtfc	0,004679922	Bacteria	Proteobacteria	Gammaproteobacteria	Betaproteobacteriales	Burkholderiaceae	Polaromonas
ASV55	Non-glacial	LacADNRUFtfb	0,00288725	Bacteria	Proteobacteria	Gammaproteobacteria	Betaproteobacteriales	Burkholderiaceae	Polaromonas
ASV55	Glacial	LacAmpRUFtf-a	0,000281305	Bacteria	Proteobacteria	Gammaproteobacteria	Betaproteobacteriales	Burkholderiaceae	Polaromonas
ASV55	Control	Ctr-tf-IIb	0,000128211	Bacteria	Proteobacteria	Gammaproteobacteria	Betaproteobacteriales	Burkholderiaceae	Polaromonas
ASV55	Baie de la Table	BdT0-2	0	Bacteria	Proteobacteria	Gammaproteobacteria	Betaproteobacteriales	Burkholderiaceae	Polaromonas
ASV55	Glacial	LacAmpRUFtf-b	0	Bacteria	Proteobacteria	Gammaproteobacteria	Betaproteobacteriales	Burkholderiaceae	Polaromonas
ASV55	Glacial	LacAmpRUFtf-c	0	Bacteria	Proteobacteria	Gammaproteobacteria	Betaproteobacteriales	Burkholderiaceae	Polaromonas
ASV568	Control	Ctr-tf-IIb	0,000240395	Bacteria	Bacteroidota	Bacteroidia	Flavobacteriales	Flavobacteriaceae	Nonlabens
ASV568	Baie de la Table	BdT0-2	0	Bacteria	Bacteroidota	Bacteroidia	Flavobacteriales	Flavobacteriaceae	Nonlabens
ASV568	Non-glacial	LacADNRUFtfa	0	Bacteria	Bacteroidota	Bacteroidia	Flavobacteriales	Flavobacteriaceae	Nonlabens
ASV568	Non-glacial	LacADNRUFtfb	0	Bacteria	Bacteroidota	Bacteroidia	Flavobacteriales	Flavobacteriaceae	Nonlabens
ASV568	Non-glacial	LacADNRUFtfc	0	Bacteria	Bacteroidota	Bacteroidia	Flavobacteriales	Flavobacteriaceae	Nonlabens
ASV568	Glacial	LacAmpRUFtf-a	0	Bacteria	Bacteroidota	Bacteroidia	Flavobacteriales	Flavobacteriaceae	Nonlabens
ASV568	Glacial	LacAmpRUFtf-b	0	Bacteria	Bacteroidota	Bacteroidia	Flavobacteriales	Flavobacteriaceae	Nonlabens
ASV568	Glacial	LacAmpRUFtf-c	0	Bacteria	Bacteroidota	Bacteroidia	Flavobacteriales	Flavobacteriaceae	Nonlabens
ASV570	Baie de la Table	BdT0-2	0	Bacteria	Myxococcota	Myxococcia	Myxococcales	Anaeromyxobacteracea	Anaeromyxobacter
ASV570	Control	Ctr-tf-IIb	0	Bacteria	Myxococcota	Myxococcia	Myxococcales	Anaeromyxobacteracea	Anaeromyxobacter
ASV570	Non-glacial	LacADNRUFtfa	0	Bacteria	Myxococcota	Myxococcia	Myxococcales	Anaeromyxobacteracea	Anaeromyxobacter
ASV570	Non-glacial	LacADNRUFtfb	0	Bacteria	Myxococcota	Myxococcia	Myxococcales	Anaeromyxobacteracea	Anaeromyxobacter
ASV570	Non-glacial	LacADNRUFtfc	0	Bacteria	Myxococcota	Myxococcia	Myxococcales	Anaeromyxobacteracea	Anaeromyxobacter
ASV570	Glacial	LacAmpRUFtf-a	0	Bacteria	Myxococcota	Myxococcia	Myxococcales	Anaeromyxobacteracea	Anaeromyxobacter

ASV570	Glacial	LacAmpRUFtf-b	0	Bacteria	Myxococcota	Myxococcia	Myxococcales	Anaeromyxobacteracea	Anaeromyxobacter
ASV570	Glacial	LacAmpRUFtf-c	0	Bacteria	Myxococcota	Myxococcia	Myxococcales	Anaeromyxobacteracea	Anaeromyxobacter
ASV571	Glacial	LacAmpRUFtf-a	0,000924289	Bacteria	Firmicutes	Bacilli	Bacillales	Bacillaceae	Bacillus_AY
ASV571	Baie de la Table	BdT0-2	0	Bacteria	Firmicutes	Bacilli	Bacillales	Bacillaceae	Bacillus_AY
ASV571	Control	Ctr-tf-IIb	0	Bacteria	Firmicutes	Bacilli	Bacillales	Bacillaceae	Bacillus_AY
ASV571	Non-glacial	LacADNRUFtfa	0	Bacteria	Firmicutes	Bacilli	Bacillales	Bacillaceae	Bacillus_AY
ASV571	Non-glacial	LacADNRUFtfb	0	Bacteria	Firmicutes	Bacilli	Bacillales	Bacillaceae	Bacillus_AY
ASV571	Non-glacial	LacADNRUFtfc	0	Bacteria	Firmicutes	Bacilli	Bacillales	Bacillaceae	Bacillus_AY
ASV571	Glacial	LacAmpRUFtf-b	0	Bacteria	Firmicutes	Bacilli	Bacillales	Bacillaceae	Bacillus_AY
ASV571	Glacial	LacAmpRUFtf-c	0	Bacteria	Firmicutes	Bacilli	Bacillales	Bacillaceae	Bacillus_AY
ASV573	Control	Ctr-tf-IIb	6,81E-05	Bacteria	Bacteroidota	Bacteroidia	Flavobacteriales	Crocinitomicaceae	Fluviicola
ASV573	Baie de la Table	BdT0-2	0	Bacteria	Bacteroidota	Bacteroidia	Flavobacteriales	Crocinitomicaceae	Fluviicola
ASV573	Non-glacial	LacADNRUFtfa	0	Bacteria	Bacteroidota	Bacteroidia	Flavobacteriales	Crocinitomicaceae	Fluviicola
ASV573	Non-glacial	LacADNRUFtfb	0	Bacteria	Bacteroidota	Bacteroidia	Flavobacteriales	Crocinitomicaceae	Fluviicola
ASV573	Non-glacial	LacADNRUFtfc	0	Bacteria	Bacteroidota	Bacteroidia	Flavobacteriales	Crocinitomicaceae	Fluviicola
ASV573	Glacial	LacAmpRUFtf-a	0	Bacteria	Bacteroidota	Bacteroidia	Flavobacteriales	Crocinitomicaceae	Fluviicola
ASV573	Glacial	LacAmpRUFtf-b	0	Bacteria	Bacteroidota	Bacteroidia	Flavobacteriales	Crocinitomicaceae	Fluviicola
ASV573	Glacial	LacAmpRUFtf-c	0	Bacteria	Bacteroidota	Bacteroidia	Flavobacteriales	Crocinitomicaceae	Fluviicola
ASV576	Baie de la Table	BdT0-2	0	Bacteria	Verrucomicrobio	Verrucomicrobiae	Pedosphaerales	Pedosphaeraceae	UBA6082
ASV576	Control	Ctr-tf-IIb	0	Bacteria	Verrucomicrobio	Verrucomicrobiae	Pedosphaerales	Pedosphaeraceae	UBA6082
ASV576	Non-glacial	LacADNRUFtfa	0	Bacteria	Verrucomicrobio	Verrucomicrobiae	Pedosphaerales	Pedosphaeraceae	UBA6082
ASV576	Non-glacial	LacADNRUFtfb	0	Bacteria	Verrucomicrobio	Verrucomicrobiae	Pedosphaerales	Pedosphaeraceae	UBA6082
ASV576	Non-glacial	LacADNRUFtfc	0	Bacteria	Verrucomicrobio	Verrucomicrobiae	Pedosphaerales	Pedosphaeraceae	UBA6082
ASV576	Glacial	LacAmpRUFtf-a	0	Bacteria	Verrucomicrobio	Verrucomicrobiae	Pedosphaerales	Pedosphaeraceae	UBA6082
ASV576	Glacial	LacAmpRUFtf-b	0	Bacteria	Verrucomicrobio	Verrucomicrobiae	Pedosphaerales	Pedosphaeraceae	UBA6082
ASV576	Glacial	LacAmpRUFtf-c	0	Bacteria	Verrucomicrobio	Verrucomicrobiae	Pedosphaerales	Pedosphaeraceae	UBA6082
ASV577	Glacial	LacAmpRUFtf-b	0,002699492	Bacteria	Actinobacteriota	Actinobacteria	Actinomycetales	Brevibacteriaceae	Brevibacterium
ASV577	Glacial	LacAmpRUFtf-c	0,001016703	Bacteria	Actinobacteriota	Actinobacteria	Actinomycetales	Brevibacteriaceae	Brevibacterium
ASV577	Baie de la Table	BdT0-2	0	Bacteria	Actinobacteriota	Actinobacteria	Actinomycetales	Brevibacteriaceae	Brevibacterium
ASV577	Control	Ctr-tf-IIb	0	Bacteria	Actinobacteriota	Actinobacteria	Actinomycetales	Brevibacteriaceae	Brevibacterium
ASV577	Non-glacial	LacADNRUFtfa	0	Bacteria	Actinobacteriota	Actinobacteria	Actinomycetales	Brevibacteriaceae	Brevibacterium
ASV577	Non-glacial	LacADNRUFtfb	0	Bacteria	Actinobacteriota	Actinobacteria	Actinomycetales	Brevibacteriaceae	Brevibacterium
ASV577	Non-glacial	LacADNRUFtfc	0	Bacteria	Actinobacteriota	Actinobacteria	Actinomycetales	Brevibacteriaceae	Brevibacterium
ASV577	Glacial	LacAmpRUFtf-a	0	Bacteria	Actinobacteriota	Actinobacteria	Actinomycetales	Brevibacteriaceae	Brevibacterium

ASV58	Control	Ctr-tf-IIb	0,003874369	Bacteria	Proteobacteria	Alphaproteobacteria	Parvibaculales	Parvibaculaceae	Mf105b01
ASV58	Non-glacial	LacADNRUFtfc	0,001309408	Bacteria	Proteobacteria	Alphaproteobacteria	Parvibaculales	Parvibaculaceae	Mf105b01
ASV58	Glacial	LacAmpRUFtf-b	0,001079797	Bacteria	Proteobacteria	Alphaproteobacteria	Parvibaculales	Parvibaculaceae	Mf105b01
ASV58	Glacial	LacAmpRUFtf-a	0,000321492	Bacteria	Proteobacteria	Alphaproteobacteria	Parvibaculales	Parvibaculaceae	Mf105b01
ASV58	Non-glacial	LacADNRUFtfb	0,000210748	Bacteria	Proteobacteria	Alphaproteobacteria	Parvibaculales	Parvibaculaceae	Mf105b01
ASV58	Baie de la Table	BdT0-2	0	Bacteria	Proteobacteria	Alphaproteobacteria	Parvibaculales	Parvibaculaceae	Mf105b01
ASV58	Non-glacial	LacADNRUFtfa	0	Bacteria	Proteobacteria	Alphaproteobacteria	Parvibaculales	Parvibaculaceae	Mf105b01
ASV58	Glacial	LacAmpRUFtf-c	0	Bacteria	Proteobacteria	Alphaproteobacteria	Parvibaculales	Parvibaculaceae	Mf105b01
ASV580	Baie de la Table	BdT0-2	0	Bacteria	Proteobacteria	Gammaproteobacteria	Betaproteobacteriales	Burkholderiaceae	Rhizobacter
ASV580	Control	Ctr-tf-IIb	0	Bacteria	Proteobacteria	Gammaproteobacteria	Betaproteobacteriales	Burkholderiaceae	Rhizobacter
ASV580	Non-glacial	LacADNRUFtfa	0	Bacteria	Proteobacteria	Gammaproteobacteria	Betaproteobacteriales	Burkholderiaceae	Rhizobacter
ASV580	Non-glacial	LacADNRUFtfb	0	Bacteria	Proteobacteria	Gammaproteobacteria	Betaproteobacteriales	Burkholderiaceae	Rhizobacter
ASV580	Non-glacial	LacADNRUFtfc	0	Bacteria	Proteobacteria	Gammaproteobacteria	Betaproteobacteriales	Burkholderiaceae	Rhizobacter
ASV580	Glacial	LacAmpRUFtf-a	0	Bacteria	Proteobacteria	Gammaproteobacteria	Betaproteobacteriales	Burkholderiaceae	Rhizobacter
ASV580	Glacial	LacAmpRUFtf-b	0	Bacteria	Proteobacteria	Gammaproteobacteria	Betaproteobacteriales	Burkholderiaceae	Rhizobacter
ASV580	Glacial	LacAmpRUFtf-c	0	Bacteria	Proteobacteria	Gammaproteobacteria	Betaproteobacteriales	Burkholderiaceae	Rhizobacter
ASV581	Non-glacial	LacADNRUFtfa	0,000692873	Bacteria	Proteobacteria	Alphaproteobacteria	Rhizobiales	Methyloligellaceae	Methyloceanibacter
ASV581	Non-glacial	LacADNRUFtfb	0,000442571	Bacteria	Proteobacteria	Alphaproteobacteria	Rhizobiales	Methyloligellaceae	Methyloceanibacter
ASV581	Non-glacial	LacADNRUFtfc	9,70E-05	Bacteria	Proteobacteria	Alphaproteobacteria	Rhizobiales	Methyloligellaceae	Methyloceanibacter
ASV581	Baie de la Table	BdT0-2	0	Bacteria	Proteobacteria	Alphaproteobacteria	Rhizobiales	Methyloligellaceae	Methyloceanibacter
ASV581	Control	Ctr-tf-IIb	0	Bacteria	Proteobacteria	Alphaproteobacteria	Rhizobiales	Methyloligellaceae	Methyloceanibacter
ASV581	Glacial	LacAmpRUFtf-a	0	Bacteria	Proteobacteria	Alphaproteobacteria	Rhizobiales	Methyloligellaceae	Methyloceanibacter
ASV581	Glacial	LacAmpRUFtf-b	0	Bacteria	Proteobacteria	Alphaproteobacteria	Rhizobiales	Methyloligellaceae	Methyloceanibacter
ASV581	Glacial	LacAmpRUFtf-c	0	Bacteria	Proteobacteria	Alphaproteobacteria	Rhizobiales	Methyloligellaceae	Methyloceanibacter
ASV584	Control	Ctr-tf-IIb	0,00035258	Bacteria	Bacteroidota	Bacteroidia	Flavobacteriales	Flavobacteriaceae	Dokdonia
ASV584	Baie de la Table	BdT0-2	0	Bacteria	Bacteroidota	Bacteroidia	Flavobacteriales	Flavobacteriaceae	Dokdonia
ASV584	Non-glacial	LacADNRUFtfa	0	Bacteria	Bacteroidota	Bacteroidia	Flavobacteriales	Flavobacteriaceae	Dokdonia
ASV584	Non-glacial	LacADNRUFtfb	0	Bacteria	Bacteroidota	Bacteroidia	Flavobacteriales	Flavobacteriaceae	Dokdonia
ASV584	Non-glacial	LacADNRUFtfc	0	Bacteria	Bacteroidota	Bacteroidia	Flavobacteriales	Flavobacteriaceae	Dokdonia
ASV584	Glacial	LacAmpRUFtf-a	0	Bacteria	Bacteroidota	Bacteroidia	Flavobacteriales	Flavobacteriaceae	Dokdonia
ASV584	Glacial	LacAmpRUFtf-b	0	Bacteria	Bacteroidota	Bacteroidia	Flavobacteriales	Flavobacteriaceae	Dokdonia
ASV584	Glacial	LacAmpRUFtf-c	0	Bacteria	Bacteroidota	Bacteroidia	Flavobacteriales	Flavobacteriaceae	Dokdonia
ASV586	Baie de la Table	BdT0-2	0	Bacteria	Proteobacteria	Gammaproteobacteria	Betaproteobacteriales	Methylophilaceae	Methylopumilus_A
ASV586	Control	Ctr-tf-IIb	0	Bacteria	Proteobacteria	Gammaproteobacteria	Betaproteobacteriales	Methylophilaceae	Methylopumilus_A

ASV586	Non-glacial	LacADNRUFtfa	0	Bacteria	Proteobacteria	Gammaproteobacteria	Betaproteobacteriales	Methylophilaceae	Methylopumilus_A
ASV586	Non-glacial	LacADNRUFtfb	0	Bacteria	Proteobacteria	Gammaproteobacteria	Betaproteobacteriales	Methylophilaceae	Methylopumilus_A
ASV586	Non-glacial	LacADNRUFtfc	0	Bacteria	Proteobacteria	Gammaproteobacteria	Betaproteobacteriales	Methylophilaceae	Methylopumilus_A
ASV586	Glacial	LacAmpRUFtf-a	0	Bacteria	Proteobacteria	Gammaproteobacteria	Betaproteobacteriales	Methylophilaceae	Methylopumilus_A
ASV586	Glacial	LacAmpRUFtf-b	0	Bacteria	Proteobacteria	Gammaproteobacteria	Betaproteobacteriales	Methylophilaceae	Methylopumilus_A
ASV586	Glacial	LacAmpRUFtf-c	0	Bacteria	Proteobacteria	Gammaproteobacteria	Betaproteobacteriales	Methylophilaceae	Methylopumilus_A
ASV591	Baie de la Table	BdT0-2	0	Bacteria	Proteobacteria	Gammaproteobacteria	Legionellales	Legionellaceae	Tatlockia
ASV591	Control	Ctr-tf-IIb	0	Bacteria	Proteobacteria	Gammaproteobacteria	Legionellales	Legionellaceae	Tatlockia
ASV591	Non-glacial	LacADNRUFtfa	0	Bacteria	Proteobacteria	Gammaproteobacteria	Legionellales	Legionellaceae	Tatlockia
ASV591	Non-glacial	LacADNRUFtfb	0	Bacteria	Proteobacteria	Gammaproteobacteria	Legionellales	Legionellaceae	Tatlockia
ASV591	Non-glacial	LacADNRUFtfc	0	Bacteria	Proteobacteria	Gammaproteobacteria	Legionellales	Legionellaceae	Tatlockia
ASV591	Glacial	LacAmpRUFtf-a	0	Bacteria	Proteobacteria	Gammaproteobacteria	Legionellales	Legionellaceae	Tatlockia
ASV591	Glacial	LacAmpRUFtf-b	0	Bacteria	Proteobacteria	Gammaproteobacteria	Legionellales	Legionellaceae	Tatlockia
ASV591	Glacial	LacAmpRUFtf-c	0	Bacteria	Proteobacteria	Gammaproteobacteria	Legionellales	Legionellaceae	Tatlockia
ASV594	Glacial	LacAmpRUFtf-c	0,003485839	Bacteria	Firmicutes	Bacilli	Staphylococcales	Staphylococcaceae	Staphylococcus
ASV594	Non-glacial	LacADNRUFtfb	0,000231823	Bacteria	Firmicutes	Bacilli	Staphylococcales	Staphylococcaceae	Staphylococcus
ASV594	Baie de la Table	BdT0-2	0	Bacteria	Firmicutes	Bacilli	Staphylococcales	Staphylococcaceae	Staphylococcus
ASV594	Control	Ctr-tf-IIb	0	Bacteria	Firmicutes	Bacilli	Staphylococcales	Staphylococcaceae	Staphylococcus
ASV594	Non-glacial	LacADNRUFtfa	0	Bacteria	Firmicutes	Bacilli	Staphylococcales	Staphylococcaceae	Staphylococcus
ASV594	Non-glacial	LacADNRUFtfc	0	Bacteria	Firmicutes	Bacilli	Staphylococcales	Staphylococcaceae	Staphylococcus
ASV594	Glacial	LacAmpRUFtf-a	0	Bacteria	Firmicutes	Bacilli	Staphylococcales	Staphylococcaceae	Staphylococcus
ASV594	Glacial	LacAmpRUFtf-b	0	Bacteria	Firmicutes	Bacilli	Staphylococcales	Staphylococcaceae	Staphylococcus
ASV595	Baie de la Table	BdT0-2	0	Bacteria	Proteobacteria	Gammaproteobacteria	Pseudomonadales	Spongiibacteraceae	Dasania
ASV595	Control	Ctr-tf-IIb	0	Bacteria	Proteobacteria	Gammaproteobacteria	Pseudomonadales	Spongiibacteraceae	Dasania
ASV595	Non-glacial	LacADNRUFtfa	0	Bacteria	Proteobacteria	Gammaproteobacteria	Pseudomonadales	Spongiibacteraceae	Dasania
ASV595	Non-glacial	LacADNRUFtfb	0	Bacteria	Proteobacteria	Gammaproteobacteria	Pseudomonadales	Spongiibacteraceae	Dasania
ASV595	Non-glacial	LacADNRUFtfc	0	Bacteria	Proteobacteria	Gammaproteobacteria	Pseudomonadales	Spongiibacteraceae	Dasania
ASV595	Glacial	LacAmpRUFtf-a	0	Bacteria	Proteobacteria	Gammaproteobacteria	Pseudomonadales	Spongiibacteraceae	Dasania
ASV595	Glacial	LacAmpRUFtf-b	0	Bacteria	Proteobacteria	Gammaproteobacteria	Pseudomonadales	Spongiibacteraceae	Dasania
ASV595	Glacial	LacAmpRUFtf-c	0	Bacteria	Proteobacteria	Gammaproteobacteria	Pseudomonadales	Spongiibacteraceae	Dasania
ASV596	Baie de la Table	BdT0-2	0	Bacteria	Bacteroidota	Bacteroidia	Cytophagales	Spirosomaceae	Emticicia
ASV596	Control	Ctr-tf-IIb	0	Bacteria	Bacteroidota	Bacteroidia	Cytophagales	Spirosomaceae	Emticicia
ASV596	Non-glacial	LacADNRUFtfa	0	Bacteria	Bacteroidota	Bacteroidia	Cytophagales	Spirosomaceae	Emticicia
ASV596	Non-glacial	LacADNRUFtfb	0	Bacteria	Bacteroidota	Bacteroidia	Cytophagales	Spirosomaceae	Emticicia

ASV596	Non-glacial	LacADNRUFtfc	0	Bacteria	Bacteroidota	Bacteroidia	Cytophagales	Spirosomaceae	Emticicia
ASV596	Glacial	LacAmpRUFtf-a	0	Bacteria	Bacteroidota	Bacteroidia	Cytophagales	Spirosomaceae	Emticicia
ASV596	Glacial	LacAmpRUFtf-b	0	Bacteria	Bacteroidota	Bacteroidia	Cytophagales	Spirosomaceae	Emticicia
ASV596	Glacial	LacAmpRUFtf-c	0	Bacteria	Bacteroidota	Bacteroidia	Cytophagales	Spirosomaceae	Emticicia
ASV60	Baie de la Table	BdT0-2	0,01120341	Bacteria	Proteobacteria	Gammaproteobacteria	SAR86	D2472	SAR86A
ASV60	Non-glacial	LacADNRUFtfa	0,002309576	Bacteria	Proteobacteria	Gammaproteobacteria	SAR86	D2472	SAR86A
ASV60	Non-glacial	LacADNRUFtfb	0,001433087	Bacteria	Proteobacteria	Gammaproteobacteria	SAR86	D2472	SAR86A
ASV60	Glacial	LacAmpRUFtf-b	0,001295756	Bacteria	Proteobacteria	Gammaproteobacteria	SAR86	D2472	SAR86A
ASV60	Glacial	LacAmpRUFtf-a	0,001085035	Bacteria	Proteobacteria	Gammaproteobacteria	SAR86	D2472	SAR86A
ASV60	Control	Ctr-tf-IIb	0,000572942	Bacteria	Proteobacteria	Gammaproteobacteria	SAR86	D2472	SAR86A
ASV60	Glacial	LacAmpRUFtf-c	0,000484144	Bacteria	Proteobacteria	Gammaproteobacteria	SAR86	D2472	SAR86A
ASV60	Non-glacial	LacADNRUFtfc	0,000460718	Bacteria	Proteobacteria	Gammaproteobacteria	SAR86	D2472	SAR86A
ASV602	Control	Ctr-tf-IIb	0,000208343	Bacteria	Bacteroidota	Bacteroidia	Flavobacteriales	Flavobacteriaceae	HC6-5
ASV602	Baie de la Table	BdT0-2	0	Bacteria	Bacteroidota	Bacteroidia	Flavobacteriales	Flavobacteriaceae	HC6-5
ASV602	Non-glacial	LacADNRUFtfa	0	Bacteria	Bacteroidota	Bacteroidia	Flavobacteriales	Flavobacteriaceae	HC6-5
ASV602	Non-glacial	LacADNRUFtfb	0	Bacteria	Bacteroidota	Bacteroidia	Flavobacteriales	Flavobacteriaceae	HC6-5
ASV602	Non-glacial	LacADNRUFtfc	0	Bacteria	Bacteroidota	Bacteroidia	Flavobacteriales	Flavobacteriaceae	HC6-5
ASV602	Glacial	LacAmpRUFtf-a	0	Bacteria	Bacteroidota	Bacteroidia	Flavobacteriales	Flavobacteriaceae	HC6-5
ASV602	Glacial	LacAmpRUFtf-b	0	Bacteria	Bacteroidota	Bacteroidia	Flavobacteriales	Flavobacteriaceae	HC6-5
ASV602	Glacial	LacAmpRUFtf-c	0	Bacteria	Bacteroidota	Bacteroidia	Flavobacteriales	Flavobacteriaceae	HC6-5
ASV607	Baie de la Table	BdT0-2	0	Bacteria	Proteobacteria	Alphaproteobacteria	Sphingomonadales	Sphingomonadaceae	Sphingopyxis
ASV607	Control	Ctr-tf-IIb	0	Bacteria	Proteobacteria	Alphaproteobacteria	Sphingomonadales	Sphingomonadaceae	Sphingopyxis
ASV607	Non-glacial	LacADNRUFtfa	0	Bacteria	Proteobacteria	Alphaproteobacteria	Sphingomonadales	Sphingomonadaceae	Sphingopyxis
ASV607	Non-glacial	LacADNRUFtfb	0	Bacteria	Proteobacteria	Alphaproteobacteria	Sphingomonadales	Sphingomonadaceae	Sphingopyxis
ASV607	Non-glacial	LacADNRUFtfc	0	Bacteria	Proteobacteria	Alphaproteobacteria	Sphingomonadales	Sphingomonadaceae	Sphingopyxis
ASV607	Glacial	LacAmpRUFtf-a	0	Bacteria	Proteobacteria	Alphaproteobacteria	Sphingomonadales	Sphingomonadaceae	Sphingopyxis
ASV607	Glacial	LacAmpRUFtf-b	0	Bacteria	Proteobacteria	Alphaproteobacteria	Sphingomonadales	Sphingomonadaceae	Sphingopyxis
ASV607	Glacial	LacAmpRUFtf-c	0	Bacteria	Proteobacteria	Alphaproteobacteria	Sphingomonadales	Sphingomonadaceae	Sphingopyxis
ASV625	Baie de la Table	BdT0-2	0	Bacteria	Planctomycetota	Planctomycetes	Planctomycetales	Planctomycetaceae	Schlesneria
ASV625	Control	Ctr-tf-IIb	0	Bacteria	Planctomycetota	Planctomycetes	Planctomycetales	Planctomycetaceae	Schlesneria
ASV625	Non-glacial	LacADNRUFtfa	0	Bacteria	Planctomycetota	Planctomycetes	Planctomycetales	Planctomycetaceae	Schlesneria
ASV625	Non-glacial	LacADNRUFtfb	0	Bacteria	Planctomycetota	Planctomycetes	Planctomycetales	Planctomycetaceae	Schlesneria
ASV625	Non-glacial	LacADNRUFtfc	0	Bacteria	Planctomycetota	Planctomycetes	Planctomycetales	Planctomycetaceae	Schlesneria
ASV625	Glacial	LacAmpRUFtf-a	0	Bacteria	Planctomycetota	Planctomycetes	Planctomycetales	Planctomycetaceae	Schlesneria

ASV625	Glacial	LacAmpRUFtf-b	0	Bacteria	Planctomycetota	Planctomycetes	Planctomycetales	Planctomycetaceae	Schlesneria
ASV625	Glacial	LacAmpRUFtf-c	0	Bacteria	Planctomycetota	Planctomycetes	Planctomycetales	Planctomycetaceae	Schlesneria
ASV632	Baie de la Table	BdT0-2	0	Bacteria	Dependentiae	Babeliae	Babeliales	Vermiphilaceae	Vermiphilus
ASV632	Control	Ctr-tf-IIb	0	Bacteria	Dependentiae	Babeliae	Babeliales	Vermiphilaceae	Vermiphilus
ASV632	Non-glacial	LacADNRUFtfa	0	Bacteria	Dependentiae	Babeliae	Babeliales	Vermiphilaceae	Vermiphilus
ASV632	Non-glacial	LacADNRUFtfb	0	Bacteria	Dependentiae	Babeliae	Babeliales	Vermiphilaceae	Vermiphilus
ASV632	Non-glacial	LacADNRUFtfc	0	Bacteria	Dependentiae	Babeliae	Babeliales	Vermiphilaceae	Vermiphilus
ASV632	Glacial	LacAmpRUFtf-a	0	Bacteria	Dependentiae	Babeliae	Babeliales	Vermiphilaceae	Vermiphilus
ASV632	Glacial	LacAmpRUFtf-b	0	Bacteria	Dependentiae	Babeliae	Babeliales	Vermiphilaceae	Vermiphilus
ASV632	Glacial	LacAmpRUFtf-c	0	Bacteria	Dependentiae	Babeliae	Babeliales	Vermiphilaceae	Vermiphilus
ASV636	Control	Ctr-tf-IIb	0,000180296	Bacteria	Desulfobacterota	Desulfovibrionia	Desulfovibrionales	Desulfovibrionaceae	Desulfovibrio_H
ASV636	Baie de la Table	BdT0-2	0	Bacteria	Desulfobacterota	Desulfovibrionia	Desulfovibrionales	Desulfovibrionaceae	Desulfovibrio_H
ASV636	Non-glacial	LacADNRUFtfa	0	Bacteria	Desulfobacterota	Desulfovibrionia	Desulfovibrionales	Desulfovibrionaceae	Desulfovibrio_H
ASV636	Non-glacial	LacADNRUFtfb	0	Bacteria	Desulfobacterota	Desulfovibrionia	Desulfovibrionales	Desulfovibrionaceae	Desulfovibrio_H
ASV636	Non-glacial	LacADNRUFtfc	0	Bacteria	Desulfobacterota	Desulfovibrionia	Desulfovibrionales	Desulfovibrionaceae	Desulfovibrio_H
ASV636	Glacial	LacAmpRUFtf-a	0	Bacteria	Desulfobacterota	Desulfovibrionia	Desulfovibrionales	Desulfovibrionaceae	Desulfovibrio_H
ASV636	Glacial	LacAmpRUFtf-b	0	Bacteria	Desulfobacterota	Desulfovibrionia	Desulfovibrionales	Desulfovibrionaceae	Desulfovibrio_H
ASV636	Glacial	LacAmpRUFtf-c	0	Bacteria	Desulfobacterota	Desulfovibrionia	Desulfovibrionales	Desulfovibrionaceae	Desulfovibrio_H
ASV641	Glacial	LacAmpRUFtf-b	0,002591513	Bacteria	Firmicutes	Bacilli	Mycoplasmatales	Ureaplasmataceae	Mycoplasma_AA
ASV641	Glacial	LacAmpRUFtf-c	9,68E-05	Bacteria	Firmicutes	Bacilli	Mycoplasmatales	Ureaplasmataceae	Mycoplasma_AA
ASV641	Baie de la Table	BdT0-2	0	Bacteria	Firmicutes	Bacilli	Mycoplasmatales	Ureaplasmataceae	Mycoplasma_AA
ASV641	Control	Ctr-tf-IIb	0	Bacteria	Firmicutes	Bacilli	Mycoplasmatales	Ureaplasmataceae	Mycoplasma_AA
ASV641	Non-glacial	LacADNRUFtfa	0	Bacteria	Firmicutes	Bacilli	Mycoplasmatales	Ureaplasmataceae	Mycoplasma_AA
ASV641	Non-glacial	LacADNRUFtfb	0	Bacteria	Firmicutes	Bacilli	Mycoplasmatales	Ureaplasmataceae	Mycoplasma_AA
ASV641	Non-glacial	LacADNRUFtfc	0	Bacteria	Firmicutes	Bacilli	Mycoplasmatales	Ureaplasmataceae	Mycoplasma_AA
ASV641	Glacial	LacAmpRUFtf-a	0	Bacteria	Firmicutes	Bacilli	Mycoplasmatales	Ureaplasmataceae	Mycoplasma_AA
ASV65	Glacial	LacAmpRUFtf-a	0,000442051	Bacteria	Proteobacteria	Gammaproteobacteria	Enterobacterales	Alteromonadaceae	Pseudoalteromonas
ASV65	Non-glacial	LacADNRUFtfa	0,000138575	Bacteria	Proteobacteria	Gammaproteobacteria	Enterobacterales	Alteromonadaceae	Pseudoalteromonas
ASV65	Control	Ctr-tf-IIb	8,41E-05	Bacteria	Proteobacteria	Gammaproteobacteria	Enterobacterales	Alteromonadaceae	Pseudoalteromonas
ASV65	Baie de la Table	BdT0-2	0	Bacteria	Proteobacteria	Gammaproteobacteria	Enterobacterales	Alteromonadaceae	Pseudoalteromonas
ASV65	Non-glacial	LacADNRUFtfb	0	Bacteria	Proteobacteria	Gammaproteobacteria	Enterobacterales	Alteromonadaceae	Pseudoalteromonas
ASV65	Non-glacial	LacADNRUFtfc	0	Bacteria	Proteobacteria	Gammaproteobacteria	Enterobacterales	Alteromonadaceae	Pseudoalteromonas
ASV65	Glacial	LacAmpRUFtf-b	0	Bacteria	Proteobacteria	Gammaproteobacteria	Enterobacterales	Alteromonadaceae	Pseudoalteromonas
ASV65	Glacial	LacAmpRUFtf-c	0	Bacteria	Proteobacteria	Gammaproteobacteria	Enterobacterales	Alteromonadaceae	Pseudoalteromonas

ASV651	Baie de la Table	BdT0-2	0	Bacteria	Cyanobacteriota	Cyanobacteriia	Cyanobacteriales	Chamaesiphonaceae	Chamaesiphon
ASV651	Control	Ctr-tf-IIb	0	Bacteria	Cyanobacteriota	Cyanobacteriia	Cyanobacteriales	Chamaesiphonaceae	Chamaesiphon
ASV651	Non-glacial	LacADNRUFtfa	0	Bacteria	Cyanobacteriota	Cyanobacteriia	Cyanobacteriales	Chamaesiphonaceae	Chamaesiphon
ASV651	Non-glacial	LacADNRUFtfb	0	Bacteria	Cyanobacteriota	Cyanobacteriia	Cyanobacteriales	Chamaesiphonaceae	Chamaesiphon
ASV651	Non-glacial	LacADNRUFtfc	0	Bacteria	Cyanobacteriota	Cyanobacteriia	Cyanobacteriales	Chamaesiphonaceae	Chamaesiphon
ASV651	Glacial	LacAmpRUFtf-a	0	Bacteria	Cyanobacteriota	Cyanobacteriia	Cyanobacteriales	Chamaesiphonaceae	Chamaesiphon
ASV651	Glacial	LacAmpRUFtf-b	0	Bacteria	Cyanobacteriota	Cyanobacteriia	Cyanobacteriales	Chamaesiphonaceae	Chamaesiphon
ASV651	Glacial	LacAmpRUFtf-c	0	Bacteria	Cyanobacteriota	Cyanobacteriia	Cyanobacteriales	Chamaesiphonaceae	Chamaesiphon
ASV654	Baie de la Table	BdT0-2	0	Bacteria	Bacteroidota	Bacteroidia	AKYH767	b-17BO	UBA2475
ASV654	Control	Ctr-tf-IIb	0	Bacteria	Bacteroidota	Bacteroidia	AKYH767	b-17BO	UBA2475
ASV654	Non-glacial	LacADNRUFtfa	0	Bacteria	Bacteroidota	Bacteroidia	AKYH767	b-17BO	UBA2475
ASV654	Non-glacial	LacADNRUFtfb	0	Bacteria	Bacteroidota	Bacteroidia	AKYH767	b-17BO	UBA2475
ASV654	Non-glacial	LacADNRUFtfc	0	Bacteria	Bacteroidota	Bacteroidia	AKYH767	b-17BO	UBA2475
ASV654	Glacial	LacAmpRUFtf-a	0	Bacteria	Bacteroidota	Bacteroidia	AKYH767	b-17BO	UBA2475
ASV654	Glacial	LacAmpRUFtf-b	0	Bacteria	Bacteroidota	Bacteroidia	AKYH767	b-17BO	UBA2475
ASV654	Glacial	LacAmpRUFtf-c	0	Bacteria	Bacteroidota	Bacteroidia	AKYH767	b-17BO	UBA2475
ASV655	Glacial	LacAmpRUFtf-a	0,001125221	Bacteria	Bacteroidota	Bacteroidia	Flavobacteriales	Crocinitomicaceae	Brumimicrobium
ASV655	Baie de la Table	BdT0-2	0	Bacteria	Bacteroidota	Bacteroidia	Flavobacteriales	Crocinitomicaceae	Brumimicrobium
ASV655	Control	Ctr-tf-IIb	0	Bacteria	Bacteroidota	Bacteroidia	Flavobacteriales	Crocinitomicaceae	Brumimicrobium
ASV655	Non-glacial	LacADNRUFtfa	0	Bacteria	Bacteroidota	Bacteroidia	Flavobacteriales	Crocinitomicaceae	Brumimicrobium
ASV655	Non-glacial	LacADNRUFtfb	0	Bacteria	Bacteroidota	Bacteroidia	Flavobacteriales	Crocinitomicaceae	Brumimicrobium
ASV655	Non-glacial	LacADNRUFtfc	0	Bacteria	Bacteroidota	Bacteroidia	Flavobacteriales	Crocinitomicaceae	Brumimicrobium
ASV655	Glacial	LacAmpRUFtf-b	0	Bacteria	Bacteroidota	Bacteroidia	Flavobacteriales	Crocinitomicaceae	Brumimicrobium
ASV655	Glacial	LacAmpRUFtf-c	0	Bacteria	Bacteroidota	Bacteroidia	Flavobacteriales	Crocinitomicaceae	Brumimicrobium
ASV656	Baie de la Table	BdT0-2	0	Bacteria	Proteobacteria	Gammaproteobacteria	HTCC5015	HTCC5015	HTCC5015
ASV656	Control	Ctr-tf-IIb	0	Bacteria	Proteobacteria	Gammaproteobacteria	HTCC5015	HTCC5015	HTCC5015
ASV656	Non-glacial	LacADNRUFtfa	0	Bacteria	Proteobacteria	Gammaproteobacteria	HTCC5015	HTCC5015	HTCC5015
ASV656	Non-glacial	LacADNRUFtfb	0	Bacteria	Proteobacteria	Gammaproteobacteria	HTCC5015	HTCC5015	HTCC5015
ASV656	Non-glacial	LacADNRUFtfc	0	Bacteria	Proteobacteria	Gammaproteobacteria	HTCC5015	HTCC5015	HTCC5015
ASV656	Glacial	LacAmpRUFtf-a	0	Bacteria	Proteobacteria	Gammaproteobacteria	HTCC5015	HTCC5015	HTCC5015
ASV656	Glacial	LacAmpRUFtf-b	0	Bacteria	Proteobacteria	Gammaproteobacteria	HTCC5015	HTCC5015	HTCC5015
ASV656	Glacial	LacAmpRUFtf-c	0	Bacteria	Proteobacteria	Gammaproteobacteria	HTCC5015	HTCC5015	HTCC5015
ASV66	Baie de la Table	BdT0-2	0	Bacteria	Verrucomicrobio	Verrucomicrobiae	Methylacidiphilales	UBA3015	UBA3015
ASV66	Control	Ctr-tf-IIb	0	Bacteria	Verrucomicrobio	Verrucomicrobiae	Methylacidiphilales	UBA3015	UBA3015

ASV66	Non-glacial	LacADNRUFtfa	0	Bacteria	Verrucomicrobio	Verrucomicrobiae	Methylacidiphilales	UBA3015	UBA3015
ASV66	Non-glacial	LacADNRUFtfb	0	Bacteria	Verrucomicrobio	Verrucomicrobiae	Methylacidiphilales	UBA3015	UBA3015
ASV66	Non-glacial	LacADNRUFtfc	0	Bacteria	Verrucomicrobio	Verrucomicrobiae	Methylacidiphilales	UBA3015	UBA3015
ASV66	Glacial	LacAmpRUFtf-a	0	Bacteria	Verrucomicrobio	Verrucomicrobiae	Methylacidiphilales	UBA3015	UBA3015
ASV66	Glacial	LacAmpRUFtf-b	0	Bacteria	Verrucomicrobio	Verrucomicrobiae	Methylacidiphilales	UBA3015	UBA3015
ASV66	Glacial	LacAmpRUFtf-c	0	Bacteria	Verrucomicrobio	Verrucomicrobiae	Methylacidiphilales	UBA3015	UBA3015
ASV668	Control	Ctr-tf-IIb	0,00016427	Bacteria	Proteobacteria	Gammaproteobacteria	Pseudomonadales	Hahellaceae	Marinobacter
ASV668	Baie de la Table	BdT0-2	0	Bacteria	Proteobacteria	Gammaproteobacteria	Pseudomonadales	Hahellaceae	Marinobacter
ASV668	Non-glacial	LacADNRUFtfa	0	Bacteria	Proteobacteria	Gammaproteobacteria	Pseudomonadales	Hahellaceae	Marinobacter
ASV668	Non-glacial	LacADNRUFtfb	0	Bacteria	Proteobacteria	Gammaproteobacteria	Pseudomonadales	Hahellaceae	Marinobacter
ASV668	Non-glacial	LacADNRUFtfc	0	Bacteria	Proteobacteria	Gammaproteobacteria	Pseudomonadales	Hahellaceae	Marinobacter
ASV668	Glacial	LacAmpRUFtf-a	0	Bacteria	Proteobacteria	Gammaproteobacteria	Pseudomonadales	Hahellaceae	Marinobacter
ASV668	Glacial	LacAmpRUFtf-b	0	Bacteria	Proteobacteria	Gammaproteobacteria	Pseudomonadales	Hahellaceae	Marinobacter
ASV668	Glacial	LacAmpRUFtf-c	0	Bacteria	Proteobacteria	Gammaproteobacteria	Pseudomonadales	Hahellaceae	Marinobacter
ASV669	Glacial	LacAmpRUFtf-c	0,000726216	Bacteria	Proteobacteria	Gammaproteobacteria	Betaproteobacteriales	Burkholderiaceae	Massilia
ASV669	Non-glacial	LacADNRUFtfb	0,000337197	Bacteria	Proteobacteria	Gammaproteobacteria	Betaproteobacteriales	Burkholderiaceae	Massilia
ASV669	Control	Ctr-tf-IIb	0,000256422	Bacteria	Proteobacteria	Gammaproteobacteria	Betaproteobacteriales	Burkholderiaceae	Massilia
ASV669	Baie de la Table	BdT0-2	0	Bacteria	Proteobacteria	Gammaproteobacteria	Betaproteobacteriales	Burkholderiaceae	Massilia
ASV669	Non-glacial	LacADNRUFtfa	0	Bacteria	Proteobacteria	Gammaproteobacteria	Betaproteobacteriales	Burkholderiaceae	Massilia
ASV669	Non-glacial	LacADNRUFtfc	0	Bacteria	Proteobacteria	Gammaproteobacteria	Betaproteobacteriales	Burkholderiaceae	Massilia
ASV669	Glacial	LacAmpRUFtf-a	0	Bacteria	Proteobacteria	Gammaproteobacteria	Betaproteobacteriales	Burkholderiaceae	Massilia
ASV669	Glacial	LacAmpRUFtf-b	0	Bacteria	Proteobacteria	Gammaproteobacteria	Betaproteobacteriales	Burkholderiaceae	Massilia
ASV670	Glacial	LacAmpRUFtf-a	0,001607459	Bacteria	Proteobacteria	Gammaproteobacteria	Pseudomonadales	Halieaceae	Luminiphilus
ASV670	Glacial	LacAmpRUFtf-b	0,001079797	Bacteria	Proteobacteria	Gammaproteobacteria	Pseudomonadales	Halieaceae	Luminiphilus
ASV670	Non-glacial	LacADNRUFtfc	0,00014549	Bacteria	Proteobacteria	Gammaproteobacteria	Pseudomonadales	Halieaceae	Luminiphilus
ASV670	Control	Ctr-tf-IIb	3,21E-05	Bacteria	Proteobacteria	Gammaproteobacteria	Pseudomonadales	Halieaceae	Luminiphilus
ASV670	Baie de la Table	BdT0-2	0	Bacteria	Proteobacteria	Gammaproteobacteria	Pseudomonadales	Halieaceae	Luminiphilus
ASV670	Non-glacial	LacADNRUFtfa	0	Bacteria	Proteobacteria	Gammaproteobacteria	Pseudomonadales	Halieaceae	Luminiphilus
ASV670	Non-glacial	LacADNRUFtfb	0	Bacteria	Proteobacteria	Gammaproteobacteria	Pseudomonadales	Halieaceae	Luminiphilus
ASV670	Glacial	LacAmpRUFtf-c	0	Bacteria	Proteobacteria	Gammaproteobacteria	Pseudomonadales	Halieaceae	Luminiphilus
ASV673	Non-glacial	LacADNRUFtfa	0,000138575	Bacteria	Proteobacteria	Gammaproteobacteria	Pseudomonadales	Saccharospirillaceae	Bermanella
ASV673	Baie de la Table	BdT0-2	0	Bacteria	Proteobacteria	Gammaproteobacteria	Pseudomonadales	Saccharospirillaceae	Bermanella
ASV673	Control	Ctr-tf-IIb	0	Bacteria	Proteobacteria	Gammaproteobacteria	Pseudomonadales	Saccharospirillaceae	Bermanella
ASV673	Non-glacial	LacADNRUFtfb	0	Bacteria	Proteobacteria	Gammaproteobacteria	Pseudomonadales	Saccharospirillaceae	Bermanella

ASV673	Non-glacial	LacADNRUFtfc	0	Bacteria	Proteobacteria	Gammaproteobacteria	Pseudomonadales	Saccharospirillaceae	Bermanella
ASV673	Glacial	LacAmpRUFtf-a	0	Bacteria	Proteobacteria	Gammaproteobacteria	Pseudomonadales	Saccharospirillaceae	Bermanella
ASV673	Glacial	LacAmpRUFtf-b	0	Bacteria	Proteobacteria	Gammaproteobacteria	Pseudomonadales	Saccharospirillaceae	Bermanella
ASV673	Glacial	LacAmpRUFtf-c	0	Bacteria	Proteobacteria	Gammaproteobacteria	Pseudomonadales	Saccharospirillaceae	Bermanella
ASV68	Non-glacial	LacADNRUFtfc	0,009626576	Bacteria	Bacteroidota	Bacteroidia	Cytophagales	Spirosomaceae	Flectobacillus
ASV68	Non-glacial	LacADNRUFtfb	0,004762908	Bacteria	Bacteroidota	Bacteroidia	Cytophagales	Spirosomaceae	Flectobacillus
ASV68	Non-glacial	LacADNRUFtfa	0,004526768	Bacteria	Bacteroidota	Bacteroidia	Cytophagales	Spirosomaceae	Flectobacillus
ASV68	Baie de la Table	BdT0-2	0	Bacteria	Bacteroidota	Bacteroidia	Cytophagales	Spirosomaceae	Flectobacillus
ASV68	Control	Ctr-tf-IIb	0	Bacteria	Bacteroidota	Bacteroidia	Cytophagales	Spirosomaceae	Flectobacillus
ASV68	Glacial	LacAmpRUFtf-a	0	Bacteria	Bacteroidota	Bacteroidia	Cytophagales	Spirosomaceae	Flectobacillus
ASV68	Glacial	LacAmpRUFtf-b	0	Bacteria	Bacteroidota	Bacteroidia	Cytophagales	Spirosomaceae	Flectobacillus
ASV68	Glacial	LacAmpRUFtf-c	0	Bacteria	Bacteroidota	Bacteroidia	Cytophagales	Spirosomaceae	Flectobacillus
ASV682	Glacial	LacAmpRUFtf-c	0,001452433	Bacteria	Bdellovibrionota	Bacteriovoracia	Bacteriovoracales	Bacteriovoracaceae	Halobacteriovorax
ASV682	Baie de la Table	BdT0-2	0	Bacteria	Bdellovibrionota	Bacteriovoracia	Bacteriovoracales	Bacteriovoracaceae	Halobacteriovorax
ASV682	Control	Ctr-tf-IIb	0	Bacteria	Bdellovibrionota	Bacteriovoracia	Bacteriovoracales	Bacteriovoracaceae	Halobacteriovorax
ASV682	Non-glacial	LacADNRUFtfa	0	Bacteria	Bdellovibrionota	Bacteriovoracia	Bacteriovoracales	Bacteriovoracaceae	Halobacteriovorax
ASV682	Non-glacial	LacADNRUFtfb	0	Bacteria	Bdellovibrionota	Bacteriovoracia	Bacteriovoracales	Bacteriovoracaceae	Halobacteriovorax
ASV682	Non-glacial	LacADNRUFtfc	0	Bacteria	Bdellovibrionota	Bacteriovoracia	Bacteriovoracales	Bacteriovoracaceae	Halobacteriovorax
ASV682	Glacial	LacAmpRUFtf-a	0	Bacteria	Bdellovibrionota	Bacteriovoracia	Bacteriovoracales	Bacteriovoracaceae	Halobacteriovorax
ASV682	Glacial	LacAmpRUFtf-b	0	Bacteria	Bdellovibrionota	Bacteriovoracia	Bacteriovoracales	Bacteriovoracaceae	Halobacteriovorax
ASV689	Baie de la Table	BdT0-2	0	Bacteria	Cyanobacteriota	Cyanobacteriia	Cyanobacteriales	Microcystaceae	Synechocystis
ASV689	Control	Ctr-tf-IIb	0	Bacteria	Cyanobacteriota	Cyanobacteriia	Cyanobacteriales	Microcystaceae	Synechocystis
ASV689	Non-glacial	LacADNRUFtfa	0	Bacteria	Cyanobacteriota	Cyanobacteriia	Cyanobacteriales	Microcystaceae	Synechocystis
ASV689	Non-glacial	LacADNRUFtfb	0	Bacteria	Cyanobacteriota	Cyanobacteriia	Cyanobacteriales	Microcystaceae	Synechocystis
ASV689	Non-glacial	LacADNRUFtfc	0	Bacteria	Cyanobacteriota	Cyanobacteriia	Cyanobacteriales	Microcystaceae	Synechocystis
ASV689	Glacial	LacAmpRUFtf-a	0	Bacteria	Cyanobacteriota	Cyanobacteriia	Cyanobacteriales	Microcystaceae	Synechocystis
ASV689	Glacial	LacAmpRUFtf-b	0	Bacteria	Cyanobacteriota	Cyanobacteriia	Cyanobacteriales	Microcystaceae	Synechocystis
ASV689	Glacial	LacAmpRUFtf-c	0	Bacteria	Cyanobacteriota	Cyanobacteriia	Cyanobacteriales	Microcystaceae	Synechocystis
ASV69	Non-glacial	LacADNRUFtfb	0,000358272	Bacteria	Actinobacteriota	Acidimicrobiia	Microtrichales	Ilumatobacteraceae	UBA3006
ASV69	Baie de la Table	BdT0-2	0	Bacteria	Actinobacteriota	Acidimicrobiia	Microtrichales	Ilumatobacteraceae	UBA3006
ASV69	Control	Ctr-tf-IIb	0	Bacteria	Actinobacteriota	Acidimicrobiia	Microtrichales	Ilumatobacteraceae	UBA3006
ASV69	Non-glacial	LacADNRUFtfa	0	Bacteria	Actinobacteriota	Acidimicrobiia	Microtrichales	Ilumatobacteraceae	UBA3006
ASV69	Non-glacial	LacADNRUFtfc	0	Bacteria	Actinobacteriota	Acidimicrobiia	Microtrichales	Ilumatobacteraceae	UBA3006
ASV69	Glacial	LacAmpRUFtf-a	0	Bacteria	Actinobacteriota	Acidimicrobiia	Microtrichales	Ilumatobacteraceae	UBA3006

ASV69	Glacial	LacAmpRUFtf-b	0	Bacteria	Actinobacteriota	Acidimicrobiia	Microtrichales	Ilumatobacteraceae	UBA3006
ASV69	Glacial	LacAmpRUFtf-c	0	Bacteria	Actinobacteriota	Acidimicrobiia	Microtrichales	Ilumatobacteraceae	UBA3006
ASV691	Baie de la Table	BdT0-2	0	Bacteria	Bacteroidota	Bacteroidia	Chitinophagales	Saprospiraceae	Haliscomenobacter
ASV691	Control	Ctr-tf-IIb	0	Bacteria	Bacteroidota	Bacteroidia	Chitinophagales	Saprospiraceae	Haliscomenobacter
ASV691	Non-glacial	LacADNRUFtfa	0	Bacteria	Bacteroidota	Bacteroidia	Chitinophagales	Saprospiraceae	Haliscomenobacter
ASV691	Non-glacial	LacADNRUFtfb	0	Bacteria	Bacteroidota	Bacteroidia	Chitinophagales	Saprospiraceae	Haliscomenobacter
ASV691	Non-glacial	LacADNRUFtfc	0	Bacteria	Bacteroidota	Bacteroidia	Chitinophagales	Saprospiraceae	Haliscomenobacter
ASV691	Glacial	LacAmpRUFtf-a	0	Bacteria	Bacteroidota	Bacteroidia	Chitinophagales	Saprospiraceae	Haliscomenobacter
ASV691	Glacial	LacAmpRUFtf-b	0	Bacteria	Bacteroidota	Bacteroidia	Chitinophagales	Saprospiraceae	Haliscomenobacter
ASV691	Glacial	LacAmpRUFtf-c	0	Bacteria	Bacteroidota	Bacteroidia	Chitinophagales	Saprospiraceae	Haliscomenobacter
ASV693	Baie de la Table	BdT0-2	0	Bacteria	Proteobacteria	Gammaproteobacteria	Betaproteobacteriales	Neisseriaceae	Neisseria
ASV693	Control	Ctr-tf-IIb	0	Bacteria	Proteobacteria	Gammaproteobacteria	Betaproteobacteriales	Neisseriaceae	Neisseria
ASV693	Non-glacial	LacADNRUFtfa	0	Bacteria	Proteobacteria	Gammaproteobacteria	Betaproteobacteriales	Neisseriaceae	Neisseria
ASV693	Non-glacial	LacADNRUFtfb	0	Bacteria	Proteobacteria	Gammaproteobacteria	Betaproteobacteriales	Neisseriaceae	Neisseria
ASV693	Non-glacial	LacADNRUFtfc	0	Bacteria	Proteobacteria	Gammaproteobacteria	Betaproteobacteriales	Neisseriaceae	Neisseria
ASV693	Glacial	LacAmpRUFtf-a	0	Bacteria	Proteobacteria	Gammaproteobacteria	Betaproteobacteriales	Neisseriaceae	Neisseria
ASV693	Glacial	LacAmpRUFtf-b	0	Bacteria	Proteobacteria	Gammaproteobacteria	Betaproteobacteriales	Neisseriaceae	Neisseria
ASV693	Glacial	LacAmpRUFtf-c	0	Bacteria	Proteobacteria	Gammaproteobacteria	Betaproteobacteriales	Neisseriaceae	Neisseria
ASV694	Glacial	LacAmpRUFtf-c	0,001016703	Bacteria	Proteobacteria	Gammaproteobacteria	Pseudomonadales	Pseudomonadaceae	Pseudomonas_D
ASV694	Glacial	LacAmpRUFtf-b	0,00010798	Bacteria	Proteobacteria	Gammaproteobacteria	Pseudomonadales	Pseudomonadaceae	Pseudomonas_D
ASV694	Baie de la Table	BdT0-2	0	Bacteria	Proteobacteria	Gammaproteobacteria	Pseudomonadales	Pseudomonadaceae	Pseudomonas_D
ASV694	Control	Ctr-tf-IIb	0	Bacteria	Proteobacteria	Gammaproteobacteria	Pseudomonadales	Pseudomonadaceae	Pseudomonas_D
ASV694	Non-glacial	LacADNRUFtfa	0	Bacteria	Proteobacteria	Gammaproteobacteria	Pseudomonadales	Pseudomonadaceae	Pseudomonas_D
ASV694	Non-glacial	LacADNRUFtfb	0	Bacteria	Proteobacteria	Gammaproteobacteria	Pseudomonadales	Pseudomonadaceae	Pseudomonas_D
ASV694	Non-glacial	LacADNRUFtfc	0	Bacteria	Proteobacteria	Gammaproteobacteria	Pseudomonadales	Pseudomonadaceae	Pseudomonas_D
ASV694	Glacial	LacAmpRUFtf-a	0	Bacteria	Proteobacteria	Gammaproteobacteria	Pseudomonadales	Pseudomonadaceae	Pseudomonas_D
ASV698	Baie de la Table	BdT0-2	0	Bacteria	Proteobacteria	Gammaproteobacteria	Pseudomonadales	Cellvibrionaceae	Teredinibacter
ASV698	Control	Ctr-tf-IIb	0	Bacteria	Proteobacteria	Gammaproteobacteria	Pseudomonadales	Cellvibrionaceae	Teredinibacter
ASV698	Non-glacial	LacADNRUFtfa	0	Bacteria	Proteobacteria	Gammaproteobacteria	Pseudomonadales	Cellvibrionaceae	Teredinibacter
ASV698	Non-glacial	LacADNRUFtfb	0	Bacteria	Proteobacteria	Gammaproteobacteria	Pseudomonadales	Cellvibrionaceae	Teredinibacter
ASV698	Non-glacial	LacADNRUFtfc	0	Bacteria	Proteobacteria	Gammaproteobacteria	Pseudomonadales	Cellvibrionaceae	Teredinibacter
ASV698	Glacial	LacAmpRUFtf-a	0	Bacteria	Proteobacteria	Gammaproteobacteria	Pseudomonadales	Cellvibrionaceae	Teredinibacter
ASV698	Glacial	LacAmpRUFtf-b	0	Bacteria	Proteobacteria	Gammaproteobacteria	Pseudomonadales	Cellvibrionaceae	Teredinibacter
ASV698	Glacial	LacAmpRUFtf-c	0	Bacteria	Proteobacteria	Gammaproteobacteria	Pseudomonadales	Cellvibrionaceae	Teredinibacter

ASV7	Non-glacial	LacADNRUFtfc	0,108947624	Bacteria	Proteobacteria	Alphaproteobacteria	Rhodobacterales	Rhodobacteraceae	Planktotalea
ASV7	Control	Ctr-tf-IIb	0,076269387	Bacteria	Proteobacteria	Alphaproteobacteria	Rhodobacterales	Rhodobacteraceae	Planktotalea
ASV7	Glacial	LacAmpRUFtf-b	0,039952489	Bacteria	Proteobacteria	Alphaproteobacteria	Rhodobacterales	Rhodobacteraceae	Planktotalea
ASV7	Glacial	LacAmpRUFtf-a	0,028572577	Bacteria	Proteobacteria	Alphaproteobacteria	Rhodobacterales	Rhodobacteraceae	Planktotalea
ASV7	Non-glacial	LacADNRUFtfb	0,025247629	Bacteria	Proteobacteria	Alphaproteobacteria	Rhodobacterales	Rhodobacteraceae	Planktotalea
ASV7	Non-glacial	LacADNRUFtfa	0,010208324	Bacteria	Proteobacteria	Alphaproteobacteria	Rhodobacterales	Rhodobacteraceae	Planktotalea
ASV7	Glacial	LacAmpRUFtf-c	0,007746308	Bacteria	Proteobacteria	Alphaproteobacteria	Rhodobacterales	Rhodobacteraceae	Planktotalea
ASV7	Baie de la Table	BdT0-2	0,001038365	Bacteria	Proteobacteria	Alphaproteobacteria	Rhodobacterales	Rhodobacteraceae	Planktotalea
ASV71	Baie de la Table	BdT0-2	0	Bacteria	Firmicutes	Bacilli	Lactobacillales	Lactobacillaceae	Lactobacillus_H
ASV71	Control	Ctr-tf-IIb	0	Bacteria	Firmicutes	Bacilli	Lactobacillales	Lactobacillaceae	Lactobacillus_H
ASV71	Non-glacial	LacADNRUFtfa	0	Bacteria	Firmicutes	Bacilli	Lactobacillales	Lactobacillaceae	Lactobacillus_H
ASV71	Non-glacial	LacADNRUFtfb	0	Bacteria	Firmicutes	Bacilli	Lactobacillales	Lactobacillaceae	Lactobacillus_H
ASV71	Non-glacial	LacADNRUFtfc	0	Bacteria	Firmicutes	Bacilli	Lactobacillales	Lactobacillaceae	Lactobacillus_H
ASV71	Glacial	LacAmpRUFtf-a	0	Bacteria	Firmicutes	Bacilli	Lactobacillales	Lactobacillaceae	Lactobacillus_H
ASV71	Glacial	LacAmpRUFtf-b	0	Bacteria	Firmicutes	Bacilli	Lactobacillales	Lactobacillaceae	Lactobacillus_H
ASV71	Glacial	LacAmpRUFtf-c	0	Bacteria	Firmicutes	Bacilli	Lactobacillales	Lactobacillaceae	Lactobacillus_H
ASV715	Control	Ctr-tf-IIb	0,000144237	Bacteria	Proteobacteria	Alphaproteobacteria	Rhizobiales	Rhizobiaceae	Ahrensia
ASV715	Baie de la Table	BdT0-2	0	Bacteria	Proteobacteria	Alphaproteobacteria	Rhizobiales	Rhizobiaceae	Ahrensia
ASV715	Non-glacial	LacADNRUFtfa	0	Bacteria	Proteobacteria	Alphaproteobacteria	Rhizobiales	Rhizobiaceae	Ahrensia
ASV715	Non-glacial	LacADNRUFtfb	0	Bacteria	Proteobacteria	Alphaproteobacteria	Rhizobiales	Rhizobiaceae	Ahrensia
ASV715	Non-glacial	LacADNRUFtfc	0	Bacteria	Proteobacteria	Alphaproteobacteria	Rhizobiales	Rhizobiaceae	Ahrensia
ASV715	Glacial	LacAmpRUFtf-a	0	Bacteria	Proteobacteria	Alphaproteobacteria	Rhizobiales	Rhizobiaceae	Ahrensia
ASV715	Glacial	LacAmpRUFtf-b	0	Bacteria	Proteobacteria	Alphaproteobacteria	Rhizobiales	Rhizobiaceae	Ahrensia
ASV715	Glacial	LacAmpRUFtf-c	0	Bacteria	Proteobacteria	Alphaproteobacteria	Rhizobiales	Rhizobiaceae	Ahrensia
ASV72	Baie de la Table	BdT0-2	0	Bacteria	Firmicutes	Bacilli	Lactobacillales	Listeriaceae	Listeria
ASV72	Control	Ctr-tf-IIb	0	Bacteria	Firmicutes	Bacilli	Lactobacillales	Listeriaceae	Listeria
ASV72	Non-glacial	LacADNRUFtfa	0	Bacteria	Firmicutes	Bacilli	Lactobacillales	Listeriaceae	Listeria
ASV72	Non-glacial	LacADNRUFtfb	0	Bacteria	Firmicutes	Bacilli	Lactobacillales	Listeriaceae	Listeria
ASV72	Non-glacial	LacADNRUFtfc	0	Bacteria	Firmicutes	Bacilli	Lactobacillales	Listeriaceae	Listeria
ASV72	Glacial	LacAmpRUFtf-a	0	Bacteria	Firmicutes	Bacilli	Lactobacillales	Listeriaceae	Listeria
ASV72	Glacial	LacAmpRUFtf-b	0	Bacteria	Firmicutes	Bacilli	Lactobacillales	Listeriaceae	Listeria
ASV72	Glacial	LacAmpRUFtf-c	0	Bacteria	Firmicutes	Bacilli	Lactobacillales	Listeriaceae	Listeria
ASV725	Baie de la Table	BdT0-2	0	Bacteria	Bacteroidota	Bacteroidia	Flavobacteriales	Crocinitomicaceae	Crocinitomix
ASV725	Control	Ctr-tf-IIb	0	Bacteria	Bacteroidota	Bacteroidia	Flavobacteriales	Crocinitomicaceae	Crocinitomix

ASV725	Non-glacial	LacADNRUFtfa	0	Bacteria	Bacteroidota	Bacteroidia	Flavobacteriales	Crocinitomicaceae	Crocinitomix
ASV725	Non-glacial	LacADNRUFtfb	0	Bacteria	Bacteroidota	Bacteroidia	Flavobacteriales	Crocinitomicaceae	Crocinitomix
ASV725	Non-glacial	LacADNRUFtfc	0	Bacteria	Bacteroidota	Bacteroidia	Flavobacteriales	Crocinitomicaceae	Crocinitomix
ASV725	Glacial	LacAmpRUFtf-a	0	Bacteria	Bacteroidota	Bacteroidia	Flavobacteriales	Crocinitomicaceae	Crocinitomix
ASV725	Glacial	LacAmpRUFtf-b	0	Bacteria	Bacteroidota	Bacteroidia	Flavobacteriales	Crocinitomicaceae	Crocinitomix
ASV725	Glacial	LacAmpRUFtf-c	0	Bacteria	Bacteroidota	Bacteroidia	Flavobacteriales	Crocinitomicaceae	Crocinitomix
ASV726	Baie de la Table	BdT0-2	0	Bacteria	Proteobacteria	Alphaproteobacteria	Rhodobacterales	Rhodobacteraceae	Pacificibacter
ASV726	Control	Ctr-tf-IIb	0	Bacteria	Proteobacteria	Alphaproteobacteria	Rhodobacterales	Rhodobacteraceae	Pacificibacter
ASV726	Non-glacial	LacADNRUFtfa	0	Bacteria	Proteobacteria	Alphaproteobacteria	Rhodobacterales	Rhodobacteraceae	Pacificibacter
ASV726	Non-glacial	LacADNRUFtfb	0	Bacteria	Proteobacteria	Alphaproteobacteria	Rhodobacterales	Rhodobacteraceae	Pacificibacter
ASV726	Non-glacial	LacADNRUFtfc	0	Bacteria	Proteobacteria	Alphaproteobacteria	Rhodobacterales	Rhodobacteraceae	Pacificibacter
ASV726	Glacial	LacAmpRUFtf-a	0	Bacteria	Proteobacteria	Alphaproteobacteria	Rhodobacterales	Rhodobacteraceae	Pacificibacter
ASV726	Glacial	LacAmpRUFtf-b	0	Bacteria	Proteobacteria	Alphaproteobacteria	Rhodobacterales	Rhodobacteraceae	Pacificibacter
ASV726	Glacial	LacAmpRUFtf-c	0	Bacteria	Proteobacteria	Alphaproteobacteria	Rhodobacterales	Rhodobacteraceae	Pacificibacter
ASV734	Baie de la Table	BdT0-2	0	Bacteria	Proteobacteria	Alphaproteobacteria	Sphingomonadales	Sphingomonadaceae	Sandarakinorhabdus
ASV734	Control	Ctr-tf-IIb	0	Bacteria	Proteobacteria	Alphaproteobacteria	Sphingomonadales	Sphingomonadaceae	Sandarakinorhabdus
ASV734	Non-glacial	LacADNRUFtfa	0	Bacteria	Proteobacteria	Alphaproteobacteria	Sphingomonadales	Sphingomonadaceae	Sandarakinorhabdus
ASV734	Non-glacial	LacADNRUFtfb	0	Bacteria	Proteobacteria	Alphaproteobacteria	Sphingomonadales	Sphingomonadaceae	Sandarakinorhabdus
ASV734	Non-glacial	LacADNRUFtfc	0	Bacteria	Proteobacteria	Alphaproteobacteria	Sphingomonadales	Sphingomonadaceae	Sandarakinorhabdus
ASV734	Glacial	LacAmpRUFtf-a	0	Bacteria	Proteobacteria	Alphaproteobacteria	Sphingomonadales	Sphingomonadaceae	Sandarakinorhabdus
ASV734	Glacial	LacAmpRUFtf-b	0	Bacteria	Proteobacteria	Alphaproteobacteria	Sphingomonadales	Sphingomonadaceae	Sandarakinorhabdus
ASV734	Glacial	LacAmpRUFtf-c	0	Bacteria	Proteobacteria	Alphaproteobacteria	Sphingomonadales	Sphingomonadaceae	Sandarakinorhabdus
ASV74	Control	Ctr-tf-IIb	0,011258509	Bacteria	Proteobacteria	Alphaproteobacteria	Rhodobacterales	Rhodobacteraceae	Loktanella
ASV74	Non-glacial	LacADNRUFtfb	0,007650158	Bacteria	Proteobacteria	Alphaproteobacteria	Rhodobacterales	Rhodobacteraceae	Loktanella
ASV74	Non-glacial	LacADNRUFtfc	0,006377304	Bacteria	Proteobacteria	Alphaproteobacteria	Rhodobacterales	Rhodobacteraceae	Loktanella
ASV74	Non-glacial	LacADNRUFtfa	0,003325789	Bacteria	Proteobacteria	Alphaproteobacteria	Rhodobacterales	Rhodobacteraceae	Loktanella
ASV74	Glacial	LacAmpRUFtf-a	0,002853239	Bacteria	Proteobacteria	Alphaproteobacteria	Rhodobacterales	Rhodobacteraceae	Loktanella
ASV74	Baie de la Table	BdT0-2	0	Bacteria	Proteobacteria	Alphaproteobacteria	Rhodobacterales	Rhodobacteraceae	Loktanella
ASV74	Glacial	LacAmpRUFtf-b	0	Bacteria	Proteobacteria	Alphaproteobacteria	Rhodobacterales	Rhodobacteraceae	Loktanella
ASV74	Glacial	LacAmpRUFtf-c	0	Bacteria	Proteobacteria	Alphaproteobacteria	Rhodobacterales	Rhodobacteraceae	Loktanella
ASV750	Glacial	LacAmpRUFtf-c	0,002372307	Bacteria	Proteobacteria	Gammaproteobacteria	Pseudomonadales	Moraxellaceae	Acinetobacter
ASV750	Non-glacial	LacADNRUFtfb	0,000252898	Bacteria	Proteobacteria	Gammaproteobacteria	Pseudomonadales	Moraxellaceae	Acinetobacter
ASV750	Control	Ctr-tf-IIb	0,000124204	Bacteria	Proteobacteria	Gammaproteobacteria	Pseudomonadales	Moraxellaceae	Acinetobacter
ASV750	Baie de la Table	BdT0-2	0	Bacteria	Proteobacteria	Gammaproteobacteria	Pseudomonadales	Moraxellaceae	Acinetobacter

ASV750	Non-glacial	LacADNRUFtfa	0	Bacteria	Proteobacteria	Gammaproteobacteria	Pseudomonadales	Moraxellaceae	Acinetobacter
ASV750	Non-glacial	LacADNRUFtfc	0	Bacteria	Proteobacteria	Gammaproteobacteria	Pseudomonadales	Moraxellaceae	Acinetobacter
ASV750	Glacial	LacAmpRUFtf-a	0	Bacteria	Proteobacteria	Gammaproteobacteria	Pseudomonadales	Moraxellaceae	Acinetobacter
ASV750	Glacial	LacAmpRUFtf-b	0	Bacteria	Proteobacteria	Gammaproteobacteria	Pseudomonadales	Moraxellaceae	Acinetobacter
ASV76	Baie de la Table	BdT0-2	0,001639523	Bacteria	Proteobacteria	Gammaproteobacteria	Pseudomonadales	Pseudohongiellaceae	OM182
ASV76	Control	Ctr-tf-IIb	0,001438365	Bacteria	Proteobacteria	Gammaproteobacteria	Pseudomonadales	Pseudohongiellaceae	OM182
ASV76	Glacial	LacAmpRUFtf-a	0,00068317	Bacteria	Proteobacteria	Gammaproteobacteria	Pseudomonadales	Pseudohongiellaceae	OM182
ASV76	Non-glacial	LacADNRUFtfa	0,000508107	Bacteria	Proteobacteria	Gammaproteobacteria	Pseudomonadales	Pseudohongiellaceae	OM182
ASV76	Non-glacial	LacADNRUFtfb	0,000295047	Bacteria	Proteobacteria	Gammaproteobacteria	Pseudomonadales	Pseudohongiellaceae	OM182
ASV76	Non-glacial	LacADNRUFtfc	0,000266731	Bacteria	Proteobacteria	Gammaproteobacteria	Pseudomonadales	Pseudohongiellaceae	OM182
ASV76	Glacial	LacAmpRUFtf-b	0	Bacteria	Proteobacteria	Gammaproteobacteria	Pseudomonadales	Pseudohongiellaceae	OM182
ASV76	Glacial	LacAmpRUFtf-c	0	Bacteria	Proteobacteria	Gammaproteobacteria	Pseudomonadales	Pseudohongiellaceae	OM182
ASV760	Glacial	LacAmpRUFtf-c	0,001113532	Bacteria	Proteobacteria	Gammaproteobacteria	Betaproteobacteriales	Neisseriaceae	Snodgrassella
ASV760	Non-glacial	LacADNRUFtfb	6,32E-05	Bacteria	Proteobacteria	Gammaproteobacteria	Betaproteobacteriales	Neisseriaceae	Snodgrassella
ASV760	Baie de la Table	BdT0-2	0	Bacteria	Proteobacteria	Gammaproteobacteria	Betaproteobacteriales	Neisseriaceae	Snodgrassella
ASV760	Control	Ctr-tf-IIb	0	Bacteria	Proteobacteria	Gammaproteobacteria	Betaproteobacteriales	Neisseriaceae	Snodgrassella
ASV760	Non-glacial	LacADNRUFtfa	0	Bacteria	Proteobacteria	Gammaproteobacteria	Betaproteobacteriales	Neisseriaceae	Snodgrassella
ASV760	Non-glacial	LacADNRUFtfc	0	Bacteria	Proteobacteria	Gammaproteobacteria	Betaproteobacteriales	Neisseriaceae	Snodgrassella
ASV760	Glacial	LacAmpRUFtf-a	0	Bacteria	Proteobacteria	Gammaproteobacteria	Betaproteobacteriales	Neisseriaceae	Snodgrassella
ASV760	Glacial	LacAmpRUFtf-b	0	Bacteria	Proteobacteria	Gammaproteobacteria	Betaproteobacteriales	Neisseriaceae	Snodgrassella
ASV766	Glacial	LacAmpRUFtf-a	8,04E-05	Bacteria	Proteobacteria	Alphaproteobacteria	Rhizobiales	Devosiaceae	Pelagibacterium
ASV766	Baie de la Table	BdT0-2	0	Bacteria	Proteobacteria	Alphaproteobacteria	Rhizobiales	Devosiaceae	Pelagibacterium
ASV766	Control	Ctr-tf-IIb	0	Bacteria	Proteobacteria	Alphaproteobacteria	Rhizobiales	Devosiaceae	Pelagibacterium
ASV766	Non-glacial	LacADNRUFtfa	0	Bacteria	Proteobacteria	Alphaproteobacteria	Rhizobiales	Devosiaceae	Pelagibacterium
ASV766	Non-glacial	LacADNRUFtfb	0	Bacteria	Proteobacteria	Alphaproteobacteria	Rhizobiales	Devosiaceae	Pelagibacterium
ASV766	Non-glacial	LacADNRUFtfc	0	Bacteria	Proteobacteria	Alphaproteobacteria	Rhizobiales	Devosiaceae	Pelagibacterium
ASV766	Glacial	LacAmpRUFtf-b	0	Bacteria	Proteobacteria	Alphaproteobacteria	Rhizobiales	Devosiaceae	Pelagibacterium
ASV766	Glacial	LacAmpRUFtf-c	0	Bacteria	Proteobacteria	Alphaproteobacteria	Rhizobiales	Devosiaceae	Pelagibacterium
ASV767	Non-glacial	LacADNRUFtfa	0,000323341	Bacteria	Firmicutes	Bacilli	Bacillales	Bacillaceae_A	Bacillus_AK
ASV767	Non-glacial	LacADNRUFtfc	0,000218235	Bacteria	Firmicutes	Bacilli	Bacillales	Bacillaceae_A	Bacillus_AK
ASV767	Baie de la Table	BdT0-2	0	Bacteria	Firmicutes	Bacilli	Bacillales	Bacillaceae_A	Bacillus_AK
ASV767	Control	Ctr-tf-IIb	0	Bacteria	Firmicutes	Bacilli	Bacillales	Bacillaceae_A	Bacillus_AK
ASV767	Non-glacial	LacADNRUFtfb	0	Bacteria	Firmicutes	Bacilli	Bacillales	Bacillaceae_A	Bacillus_AK
ASV767	Glacial	LacAmpRUFtf-a	0	Bacteria	Firmicutes	Bacilli	Bacillales	Bacillaceae_A	Bacillus_AK

ASV767	Glacial	LacAmpRUFtf-b	0	Bacteria	Firmicutes	Bacilli	Bacillales	Bacillaceae_A	Bacillus_AK
ASV767	Glacial	LacAmpRUFtf-c	0	Bacteria	Firmicutes	Bacilli	Bacillales	Bacillaceae_A	Bacillus_AK
ASV780	Baie de la Table	BdT0-2	0	Bacteria	Proteobacteria	Alphaproteobacteria	Rhizobiales	Devosiaceae	Devosia
ASV780	Control	Ctr-tf-IIb	0	Bacteria	Proteobacteria	Alphaproteobacteria	Rhizobiales	Devosiaceae	Devosia
ASV780	Non-glacial	LacADNRUFtfa	0	Bacteria	Proteobacteria	Alphaproteobacteria	Rhizobiales	Devosiaceae	Devosia
ASV780	Non-glacial	LacADNRUFtfb	0	Bacteria	Proteobacteria	Alphaproteobacteria	Rhizobiales	Devosiaceae	Devosia
ASV780	Non-glacial	LacADNRUFtfc	0	Bacteria	Proteobacteria	Alphaproteobacteria	Rhizobiales	Devosiaceae	Devosia
ASV780	Glacial	LacAmpRUFtf-a	0	Bacteria	Proteobacteria	Alphaproteobacteria	Rhizobiales	Devosiaceae	Devosia
ASV780	Glacial	LacAmpRUFtf-b	0	Bacteria	Proteobacteria	Alphaproteobacteria	Rhizobiales	Devosiaceae	Devosia
ASV780	Glacial	LacAmpRUFtf-c	0	Bacteria	Proteobacteria	Alphaproteobacteria	Rhizobiales	Devosiaceae	Devosia
ASV789	Glacial	LacAmpRUFtf-c	0,001404018	Bacteria	Actinobacteriota	Actinobacteria	Actinomycetales	Micrococcaceae	Glutamicibacter
ASV789	Baie de la Table	BdT0-2	0	Bacteria	Actinobacteriota	Actinobacteria	Actinomycetales	Micrococcaceae	Glutamicibacter
ASV789	Control	Ctr-tf-IIb	0	Bacteria	Actinobacteriota	Actinobacteria	Actinomycetales	Micrococcaceae	Glutamicibacter
ASV789	Non-glacial	LacADNRUFtfa	0	Bacteria	Actinobacteriota	Actinobacteria	Actinomycetales	Micrococcaceae	Glutamicibacter
ASV789	Non-glacial	LacADNRUFtfb	0	Bacteria	Actinobacteriota	Actinobacteria	Actinomycetales	Micrococcaceae	Glutamicibacter
ASV789	Non-glacial	LacADNRUFtfc	0	Bacteria	Actinobacteriota	Actinobacteria	Actinomycetales	Micrococcaceae	Glutamicibacter
ASV789	Glacial	LacAmpRUFtf-a	0	Bacteria	Actinobacteriota	Actinobacteria	Actinomycetales	Micrococcaceae	Glutamicibacter
ASV789	Glacial	LacAmpRUFtf-b	0	Bacteria	Actinobacteriota	Actinobacteria	Actinomycetales	Micrococcaceae	Glutamicibacter
ASV797	Control	Ctr-tf-IIb	2,80E-05	Bacteria	Proteobacteria	Alphaproteobacteria	Rhodobacterales	Rhodobacteraceae	Pseudopelagicola
ASV797	Baie de la Table	BdT0-2	0	Bacteria	Proteobacteria	Alphaproteobacteria	Rhodobacterales	Rhodobacteraceae	Pseudopelagicola
ASV797	Non-glacial	LacADNRUFtfa	0	Bacteria	Proteobacteria	Alphaproteobacteria	Rhodobacterales	Rhodobacteraceae	Pseudopelagicola
ASV797	Non-glacial	LacADNRUFtfb	0	Bacteria	Proteobacteria	Alphaproteobacteria	Rhodobacterales	Rhodobacteraceae	Pseudopelagicola
ASV797	Non-glacial	LacADNRUFtfc	0	Bacteria	Proteobacteria	Alphaproteobacteria	Rhodobacterales	Rhodobacteraceae	Pseudopelagicola
ASV797	Glacial	LacAmpRUFtf-a	0	Bacteria	Proteobacteria	Alphaproteobacteria	Rhodobacterales	Rhodobacteraceae	Pseudopelagicola
ASV797	Glacial	LacAmpRUFtf-b	0	Bacteria	Proteobacteria	Alphaproteobacteria	Rhodobacterales	Rhodobacteraceae	Pseudopelagicola
ASV797	Glacial	LacAmpRUFtf-c	0	Bacteria	Proteobacteria	Alphaproteobacteria	Rhodobacterales	Rhodobacteraceae	Pseudopelagicola
ASV80	Baie de la Table	BdT0-2	0	Bacteria	Verrucomicrobio	Verrucomicrobiae	Chthoniobacterales	UBA6821	UBA6821
ASV80	Control	Ctr-tf-IIb	0	Bacteria	Verrucomicrobio	Verrucomicrobiae	Chthoniobacterales	UBA6821	UBA6821
ASV80	Non-glacial	LacADNRUFtfa	0	Bacteria	Verrucomicrobio	Verrucomicrobiae	Chthoniobacterales	UBA6821	UBA6821
ASV80	Non-glacial	LacADNRUFtfb	0	Bacteria	Verrucomicrobio	Verrucomicrobiae	Chthoniobacterales	UBA6821	UBA6821
ASV80	Non-glacial	LacADNRUFtfc	0	Bacteria	Verrucomicrobio	Verrucomicrobiae	Chthoniobacterales	UBA6821	UBA6821
ASV80	Glacial	LacAmpRUFtf-a	0	Bacteria	Verrucomicrobio	Verrucomicrobiae	Chthoniobacterales	UBA6821	UBA6821
ASV80	Glacial	LacAmpRUFtf-b	0	Bacteria	Verrucomicrobio	Verrucomicrobiae	Chthoniobacterales	UBA6821	UBA6821
ASV80	Glacial	LacAmpRUFtf-c	0	Bacteria	Verrucomicrobio	Verrucomicrobiae	Chthoniobacterales	UBA6821	UBA6821

ASV803	Glacial	LacAmpRUFtf-a	0,000160746	Bacteria	Proteobacteria	Gammaproteobacteria	Pseudomonadales	Alcanivoracaceae	Alcanivorax
ASV803	Baie de la Table	BdT0-2	0	Bacteria	Proteobacteria	Gammaproteobacteria	Pseudomonadales	Alcanivoracaceae	Alcanivorax
ASV803	Control	Ctr-tf-IIb	0	Bacteria	Proteobacteria	Gammaproteobacteria	Pseudomonadales	Alcanivoracaceae	Alcanivorax
ASV803	Non-glacial	LacADNRUFtfa	0	Bacteria	Proteobacteria	Gammaproteobacteria	Pseudomonadales	Alcanivoracaceae	Alcanivorax
ASV803	Non-glacial	LacADNRUFtfb	0	Bacteria	Proteobacteria	Gammaproteobacteria	Pseudomonadales	Alcanivoracaceae	Alcanivorax
ASV803	Non-glacial	LacADNRUFtfc	0	Bacteria	Proteobacteria	Gammaproteobacteria	Pseudomonadales	Alcanivoracaceae	Alcanivorax
ASV803	Glacial	LacAmpRUFtf-b	0	Bacteria	Proteobacteria	Gammaproteobacteria	Pseudomonadales	Alcanivoracaceae	Alcanivorax
ASV803	Glacial	LacAmpRUFtf-c	0	Bacteria	Proteobacteria	Gammaproteobacteria	Pseudomonadales	Alcanivoracaceae	Alcanivorax
ASV809	Glacial	LacAmpRUFtf-a	0,000482238	Bacteria	Verrucomicrobio	Lentisphaeria	Lentisphaerales	Lentisphaeraceae	Lentisphaera
ASV809	Baie de la Table	BdT0-2	0,000109302	Bacteria	Verrucomicrobio	Lentisphaeria	Lentisphaerales	Lentisphaeraceae	Lentisphaera
ASV809	Control	Ctr-tf-IIb	0	Bacteria	Verrucomicrobio	Lentisphaeria	Lentisphaerales	Lentisphaeraceae	Lentisphaera
ASV809	Non-glacial	LacADNRUFtfa	0	Bacteria	Verrucomicrobio	Lentisphaeria	Lentisphaerales	Lentisphaeraceae	Lentisphaera
ASV809	Non-glacial	LacADNRUFtfb	0	Bacteria	Verrucomicrobio	Lentisphaeria	Lentisphaerales	Lentisphaeraceae	Lentisphaera
ASV809	Non-glacial	LacADNRUFtfc	0	Bacteria	Verrucomicrobio	Lentisphaeria	Lentisphaerales	Lentisphaeraceae	Lentisphaera
ASV809	Glacial	LacAmpRUFtf-b	0	Bacteria	Verrucomicrobio	Lentisphaeria	Lentisphaerales	Lentisphaeraceae	Lentisphaera
ASV809	Glacial	LacAmpRUFtf-c	0	Bacteria	Verrucomicrobio	Lentisphaeria	Lentisphaerales	Lentisphaeraceae	Lentisphaera
ASV811	Glacial	LacAmpRUFtf-c	0,000387315	Bacteria	Actinobacteriota	Actinobacteria	Actinomycetales	Micrococcaceae	Micrococcus
ASV811	Non-glacial	LacADNRUFtfb	0,000273973	Bacteria	Actinobacteriota	Actinobacteria	Actinomycetales	Micrococcaceae	Micrococcus
ASV811	Baie de la Table	BdT0-2	0	Bacteria	Actinobacteriota	Actinobacteria	Actinomycetales	Micrococcaceae	Micrococcus
ASV811	Control	Ctr-tf-IIb	0	Bacteria	Actinobacteriota	Actinobacteria	Actinomycetales	Micrococcaceae	Micrococcus
ASV811	Non-glacial	LacADNRUFtfa	0	Bacteria	Actinobacteriota	Actinobacteria	Actinomycetales	Micrococcaceae	Micrococcus
ASV811	Non-glacial	LacADNRUFtfc	0	Bacteria	Actinobacteriota	Actinobacteria	Actinomycetales	Micrococcaceae	Micrococcus
ASV811	Glacial	LacAmpRUFtf-a	0	Bacteria	Actinobacteriota	Actinobacteria	Actinomycetales	Micrococcaceae	Micrococcus
ASV811	Glacial	LacAmpRUFtf-b	0	Bacteria	Actinobacteriota	Actinobacteria	Actinomycetales	Micrococcaceae	Micrococcus
ASV819	Glacial	LacAmpRUFtf-b	0,000971817	Bacteria	Proteobacteria	Gammaproteobacteria	Pseudomonadales	Nitrincolaceae	Rs1
ASV819	Control	Ctr-tf-IIb	0,000104171	Bacteria	Proteobacteria	Gammaproteobacteria	Pseudomonadales	Nitrincolaceae	Rs1
ASV819	Baie de la Table	BdT0-2	0	Bacteria	Proteobacteria	Gammaproteobacteria	Pseudomonadales	Nitrincolaceae	Rs1
ASV819	Non-glacial	LacADNRUFtfa	0	Bacteria	Proteobacteria	Gammaproteobacteria	Pseudomonadales	Nitrincolaceae	Rs1
ASV819	Non-glacial	LacADNRUFtfb	0	Bacteria	Proteobacteria	Gammaproteobacteria	Pseudomonadales	Nitrincolaceae	Rs1
ASV819	Non-glacial	LacADNRUFtfc	0	Bacteria	Proteobacteria	Gammaproteobacteria	Pseudomonadales	Nitrincolaceae	Rs1
ASV819	Glacial	LacAmpRUFtf-a	0	Bacteria	Proteobacteria	Gammaproteobacteria	Pseudomonadales	Nitrincolaceae	Rs1
ASV819	Glacial	LacAmpRUFtf-c	0	Bacteria	Proteobacteria	Gammaproteobacteria	Pseudomonadales	Nitrincolaceae	Rs1
ASV822	Baie de la Table	BdT0-2	0	Bacteria	Verrucomicrobio	Verrucomicrobiae	Chthoniobacterales	Chthoniobacteraceae	Chthoniobacter
ASV822	Control	Ctr-tf-IIb	0	Bacteria	Verrucomicrobio	Verrucomicrobiae	Chthoniobacterales	Chthoniobacteraceae	Chthoniobacter

ASV822	Non-glacial	LacADNRUFtfa	0	Bacteria	Verrucomicrobio	Verrucomicrobiae	Chthoniobacterales	Chthoniobacteraceae	Chthoniobacter
ASV822	Non-glacial	LacADNRUFtfb	0	Bacteria	Verrucomicrobio	Verrucomicrobiae	Chthoniobacterales	Chthoniobacteraceae	Chthoniobacter
ASV822	Non-glacial	LacADNRUFtfc	0	Bacteria	Verrucomicrobio	Verrucomicrobiae	Chthoniobacterales	Chthoniobacteraceae	Chthoniobacter
ASV822	Glacial	LacAmpRUFtf-a	0	Bacteria	Verrucomicrobio	Verrucomicrobiae	Chthoniobacterales	Chthoniobacteraceae	Chthoniobacter
ASV822	Glacial	LacAmpRUFtf-b	0	Bacteria	Verrucomicrobio	Verrucomicrobiae	Chthoniobacterales	Chthoniobacteraceae	Chthoniobacter
ASV822	Glacial	LacAmpRUFtf-c	0	Bacteria	Verrucomicrobio	Verrucomicrobiae	Chthoniobacterales	Chthoniobacteraceae	Chthoniobacter
ASV829	Baie de la Table	BdT0-2	0	Bacteria	Bacteroidota	Bacteroidia	Flavobacteriales	Flavobacteriaceae	LPB0136
ASV829	Control	Ctr-tf-IIb	0	Bacteria	Bacteroidota	Bacteroidia	Flavobacteriales	Flavobacteriaceae	LPB0136
ASV829	Non-glacial	LacADNRUFtfa	0	Bacteria	Bacteroidota	Bacteroidia	Flavobacteriales	Flavobacteriaceae	LPB0136
ASV829	Non-glacial	LacADNRUFtfb	0	Bacteria	Bacteroidota	Bacteroidia	Flavobacteriales	Flavobacteriaceae	LPB0136
ASV829	Non-glacial	LacADNRUFtfc	0	Bacteria	Bacteroidota	Bacteroidia	Flavobacteriales	Flavobacteriaceae	LPB0136
ASV829	Glacial	LacAmpRUFtf-a	0	Bacteria	Bacteroidota	Bacteroidia	Flavobacteriales	Flavobacteriaceae	LPB0136
ASV829	Glacial	LacAmpRUFtf-b	0	Bacteria	Bacteroidota	Bacteroidia	Flavobacteriales	Flavobacteriaceae	LPB0136
ASV829	Glacial	LacAmpRUFtf-c	0	Bacteria	Bacteroidota	Bacteroidia	Flavobacteriales	Flavobacteriaceae	LPB0136
ASV831	Control	Ctr-tf-IIb	0,000100165	Bacteria	Bacteroidota	Bacteroidia	Flavobacteriales	Flavobacteriaceae	LPB0005
ASV831	Baie de la Table	BdT0-2	0	Bacteria	Bacteroidota	Bacteroidia	Flavobacteriales	Flavobacteriaceae	LPB0005
ASV831	Non-glacial	LacADNRUFtfa	0	Bacteria	Bacteroidota	Bacteroidia	Flavobacteriales	Flavobacteriaceae	LPB0005
ASV831	Non-glacial	LacADNRUFtfb	0	Bacteria	Bacteroidota	Bacteroidia	Flavobacteriales	Flavobacteriaceae	LPB0005
ASV831	Non-glacial	LacADNRUFtfc	0	Bacteria	Bacteroidota	Bacteroidia	Flavobacteriales	Flavobacteriaceae	LPB0005
ASV831	Glacial	LacAmpRUFtf-a	0	Bacteria	Bacteroidota	Bacteroidia	Flavobacteriales	Flavobacteriaceae	LPB0005
ASV831	Glacial	LacAmpRUFtf-b	0	Bacteria	Bacteroidota	Bacteroidia	Flavobacteriales	Flavobacteriaceae	LPB0005
ASV831	Glacial	LacAmpRUFtf-c	0	Bacteria	Bacteroidota	Bacteroidia	Flavobacteriales	Flavobacteriaceae	LPB0005
ASV839	Baie de la Table	BdT0-2	0	Bacteria	Proteobacteria	Alphaproteobacteria	Rhizobiales	Rhizobiaceae	Pseudorhizobium
ASV839	Control	Ctr-tf-IIb	0	Bacteria	Proteobacteria	Alphaproteobacteria	Rhizobiales	Rhizobiaceae	Pseudorhizobium
ASV839	Non-glacial	LacADNRUFtfa	0	Bacteria	Proteobacteria	Alphaproteobacteria	Rhizobiales	Rhizobiaceae	Pseudorhizobium
ASV839	Non-glacial	LacADNRUFtfb	0	Bacteria	Proteobacteria	Alphaproteobacteria	Rhizobiales	Rhizobiaceae	Pseudorhizobium
ASV839	Non-glacial	LacADNRUFtfc	0	Bacteria	Proteobacteria	Alphaproteobacteria	Rhizobiales	Rhizobiaceae	Pseudorhizobium
ASV839	Glacial	LacAmpRUFtf-a	0	Bacteria	Proteobacteria	Alphaproteobacteria	Rhizobiales	Rhizobiaceae	Pseudorhizobium
ASV839	Glacial	LacAmpRUFtf-b	0	Bacteria	Proteobacteria	Alphaproteobacteria	Rhizobiales	Rhizobiaceae	Pseudorhizobium
ASV839	Glacial	LacAmpRUFtf-c	0	Bacteria	Proteobacteria	Alphaproteobacteria	Rhizobiales	Rhizobiaceae	Pseudorhizobium
ASV844	Baie de la Table	BdT0-2	0	Bacteria	Proteobacteria	Gammaproteobacteria	UBA11654	UBA11654	UBA11654
ASV844	Control	Ctr-tf-IIb	0	Bacteria	Proteobacteria	Gammaproteobacteria	UBA11654	UBA11654	UBA11654
ASV844	Non-glacial	LacADNRUFtfa	0	Bacteria	Proteobacteria	Gammaproteobacteria	UBA11654	UBA11654	UBA11654
ASV844	Non-glacial	LacADNRUFtfb	0	Bacteria	Proteobacteria	Gammaproteobacteria	UBA11654	UBA11654	UBA11654

ASV844	Non-glacial	LacADNRUFtfc	0	Bacteria	Proteobacteria	Gammaproteobacteria	UBA11654	UBA11654	UBA11654
ASV844	Glacial	LacAmpRUFtf-a	0	Bacteria	Proteobacteria	Gammaproteobacteria	UBA11654	UBA11654	UBA11654
ASV844	Glacial	LacAmpRUFtf-b	0	Bacteria	Proteobacteria	Gammaproteobacteria	UBA11654	UBA11654	UBA11654
ASV844	Glacial	LacAmpRUFtf-c	0	Bacteria	Proteobacteria	Gammaproteobacteria	UBA11654	UBA11654	UBA11654
ASV851	Glacial	LacAmpRUFtf-c	0,000580973	Bacteria	Proteobacteria	Gammaproteobacteria	Betaproteobacteriales	Burkholderiaceae	Ralstonia
ASV851	Baie de la Table	BdT0-2	0	Bacteria	Proteobacteria	Gammaproteobacteria	Betaproteobacteriales	Burkholderiaceae	Ralstonia
ASV851	Control	Ctr-tf-IIb	0	Bacteria	Proteobacteria	Gammaproteobacteria	Betaproteobacteriales	Burkholderiaceae	Ralstonia
ASV851	Non-glacial	LacADNRUFtfa	0	Bacteria	Proteobacteria	Gammaproteobacteria	Betaproteobacteriales	Burkholderiaceae	Ralstonia
ASV851	Non-glacial	LacADNRUFtfb	0	Bacteria	Proteobacteria	Gammaproteobacteria	Betaproteobacteriales	Burkholderiaceae	Ralstonia
ASV851	Non-glacial	LacADNRUFtfc	0	Bacteria	Proteobacteria	Gammaproteobacteria	Betaproteobacteriales	Burkholderiaceae	Ralstonia
ASV851	Glacial	LacAmpRUFtf-a	0	Bacteria	Proteobacteria	Gammaproteobacteria	Betaproteobacteriales	Burkholderiaceae	Ralstonia
ASV851	Glacial	LacAmpRUFtf-b	0	Bacteria	Proteobacteria	Gammaproteobacteria	Betaproteobacteriales	Burkholderiaceae	Ralstonia
ASV86	Non-glacial	LacADNRUFtfb	0,000674394	Bacteria	Planctomycetota	Planctomycetes	Pirellulales	Thermoguttaceae	RBG-16-64-12
ASV86	Glacial	LacAmpRUFtf-a	0,000401865	Bacteria	Planctomycetota	Planctomycetes	Pirellulales	Thermoguttaceae	RBG-16-64-12
ASV86	Non-glacial	LacADNRUFtfc	0,000387973	Bacteria	Planctomycetota	Planctomycetes	Pirellulales	Thermoguttaceae	RBG-16-64-12
ASV86	Non-glacial	LacADNRUFtfa	0,000138575	Bacteria	Planctomycetota	Planctomycetes	Pirellulales	Thermoguttaceae	RBG-16-64-12
ASV86	Control	Ctr-tf-IIb	2,40E-05	Bacteria	Planctomycetota	Planctomycetes	Pirellulales	Thermoguttaceae	RBG-16-64-12
ASV86	Baie de la Table	BdT0-2	0	Bacteria	Planctomycetota	Planctomycetes	Pirellulales	Thermoguttaceae	RBG-16-64-12
ASV86	Glacial	LacAmpRUFtf-b	0	Bacteria	Planctomycetota	Planctomycetes	Pirellulales	Thermoguttaceae	RBG-16-64-12
ASV86	Glacial	LacAmpRUFtf-c	0	Bacteria	Planctomycetota	Planctomycetes	Pirellulales	Thermoguttaceae	RBG-16-64-12
ASV865	Baie de la Table	BdT0-2	0	Bacteria	Bacteroidota	Bacteroidia	Sphingobacteriales	Sphingobacteriaceae	Pedobacter_A
ASV865	Control	Ctr-tf-IIb	0	Bacteria	Bacteroidota	Bacteroidia	Sphingobacteriales	Sphingobacteriaceae	Pedobacter_A
ASV865	Non-glacial	LacADNRUFtfa	0	Bacteria	Bacteroidota	Bacteroidia	Sphingobacteriales	Sphingobacteriaceae	Pedobacter_A
ASV865	Non-glacial	LacADNRUFtfb	0	Bacteria	Bacteroidota	Bacteroidia	Sphingobacteriales	Sphingobacteriaceae	Pedobacter_A
ASV865	Non-glacial	LacADNRUFtfc	0	Bacteria	Bacteroidota	Bacteroidia	Sphingobacteriales	Sphingobacteriaceae	Pedobacter_A
ASV865	Glacial	LacAmpRUFtf-a	0	Bacteria	Bacteroidota	Bacteroidia	Sphingobacteriales	Sphingobacteriaceae	Pedobacter_A
ASV865	Glacial	LacAmpRUFtf-b	0	Bacteria	Bacteroidota	Bacteroidia	Sphingobacteriales	Sphingobacteriaceae	Pedobacter_A
ASV865	Glacial	LacAmpRUFtf-c	0	Bacteria	Bacteroidota	Bacteroidia	Sphingobacteriales	Sphingobacteriaceae	Pedobacter_A
ASV868	Glacial	LacAmpRUFtf-b	0,002807472	Bacteria	Proteobacteria	Gammaproteobacteria	Pseudomonadales	Halomonadaceae	Halomonas
ASV868	Non-glacial	LacADNRUFtfb	8,43E-05	Bacteria	Proteobacteria	Gammaproteobacteria	Pseudomonadales	Halomonadaceae	Halomonas
ASV868	Baie de la Table	BdT0-2	0	Bacteria	Proteobacteria	Gammaproteobacteria	Pseudomonadales	Halomonadaceae	Halomonas
ASV868	Control	Ctr-tf-IIb	0	Bacteria	Proteobacteria	Gammaproteobacteria	Pseudomonadales	Halomonadaceae	Halomonas
ASV868	Non-glacial	LacADNRUFtfa	0	Bacteria	Proteobacteria	Gammaproteobacteria	Pseudomonadales	Halomonadaceae	Halomonas
ASV868	Non-glacial	LacADNRUFtfc	0	Bacteria	Proteobacteria	Gammaproteobacteria	Pseudomonadales	Halomonadaceae	Halomonas

ASV868	Glacial	LacAmpRUFtf-a	0	Bacteria	Proteobacteria	Gammaproteobacteria	Pseudomonadales	Halomonadaceae	Halomonas
ASV868	Glacial	LacAmpRUFtf-c	0	Bacteria	Proteobacteria	Gammaproteobacteria	Pseudomonadales	Halomonadaceae	Halomonas
ASV874	Baie de la Table	BdT0-2	0	Bacteria	Elusimicrobiota	Elusimicrobia	UBA1565	UBA9628	GWA2-66-18
ASV874	Control	Ctr-tf-IIb	0	Bacteria	Elusimicrobiota	Elusimicrobia	UBA1565	UBA9628	GWA2-66-18
ASV874	Non-glacial	LacADNRUFtfa	0	Bacteria	Elusimicrobiota	Elusimicrobia	UBA1565	UBA9628	GWA2-66-18
ASV874	Non-glacial	LacADNRUFtfb	0	Bacteria	Elusimicrobiota	Elusimicrobia	UBA1565	UBA9628	GWA2-66-18
ASV874	Non-glacial	LacADNRUFtfc	0	Bacteria	Elusimicrobiota	Elusimicrobia	UBA1565	UBA9628	GWA2-66-18
ASV874	Glacial	LacAmpRUFtf-a	0	Bacteria	Elusimicrobiota	Elusimicrobia	UBA1565	UBA9628	GWA2-66-18
ASV874	Glacial	LacAmpRUFtf-b	0	Bacteria	Elusimicrobiota	Elusimicrobia	UBA1565	UBA9628	GWA2-66-18
ASV874	Glacial	LacAmpRUFtf-c	0	Bacteria	Elusimicrobiota	Elusimicrobia	UBA1565	UBA9628	GWA2-66-18
ASV876	Glacial	LacAmpRUFtf-c	0,001597676	Bacteria	Firmicutes_A	Clostridia	Tissierellales	Helcococcaceae	Anaerococcus
ASV876	Non-glacial	LacADNRUFtfb	0,000400421	Bacteria	Firmicutes_A	Clostridia	Tissierellales	Helcococcaceae	Anaerococcus
ASV876	Baie de la Table	BdT0-2	0	Bacteria	Firmicutes_A	Clostridia	Tissierellales	Helcococcaceae	Anaerococcus
ASV876	Control	Ctr-tf-IIb	0	Bacteria	Firmicutes_A	Clostridia	Tissierellales	Helcococcaceae	Anaerococcus
ASV876	Non-glacial	LacADNRUFtfa	0	Bacteria	Firmicutes_A	Clostridia	Tissierellales	Helcococcaceae	Anaerococcus
ASV876	Non-glacial	LacADNRUFtfc	0	Bacteria	Firmicutes_A	Clostridia	Tissierellales	Helcococcaceae	Anaerococcus
ASV876	Glacial	LacAmpRUFtf-a	0	Bacteria	Firmicutes_A	Clostridia	Tissierellales	Helcococcaceae	Anaerococcus
ASV876	Glacial	LacAmpRUFtf-b	0	Bacteria	Firmicutes_A	Clostridia	Tissierellales	Helcococcaceae	Anaerococcus
ASV885	Baie de la Table	BdT0-2	0	Bacteria	Proteobacteria	Alphaproteobacteria	Rickettsiales	Rickettsiaceae	Rickettsia
ASV885	Control	Ctr-tf-IIb	0	Bacteria	Proteobacteria	Alphaproteobacteria	Rickettsiales	Rickettsiaceae	Rickettsia
ASV885	Non-glacial	LacADNRUFtfa	0	Bacteria	Proteobacteria	Alphaproteobacteria	Rickettsiales	Rickettsiaceae	Rickettsia
ASV885	Non-glacial	LacADNRUFtfb	0	Bacteria	Proteobacteria	Alphaproteobacteria	Rickettsiales	Rickettsiaceae	Rickettsia
ASV885	Non-glacial	LacADNRUFtfc	0	Bacteria	Proteobacteria	Alphaproteobacteria	Rickettsiales	Rickettsiaceae	Rickettsia
ASV885	Glacial	LacAmpRUFtf-a	0	Bacteria	Proteobacteria	Alphaproteobacteria	Rickettsiales	Rickettsiaceae	Rickettsia
ASV885	Glacial	LacAmpRUFtf-b	0	Bacteria	Proteobacteria	Alphaproteobacteria	Rickettsiales	Rickettsiaceae	Rickettsia
ASV885	Glacial	LacAmpRUFtf-c	0	Bacteria	Proteobacteria	Alphaproteobacteria	Rickettsiales	Rickettsiaceae	Rickettsia
ASV886	Glacial	LacAmpRUFtf-a	0,000160746	Bacteria	Marinisomatota	Marinisomatia	Marinisomatales	Marinisomataceae	Marinisoma
ASV886	Baie de la Table	BdT0-2	0	Bacteria	Marinisomatota	Marinisomatia	Marinisomatales	Marinisomataceae	Marinisoma
ASV886	Control	Ctr-tf-IIb	0	Bacteria	Marinisomatota	Marinisomatia	Marinisomatales	Marinisomataceae	Marinisoma
ASV886	Non-glacial	LacADNRUFtfa	0	Bacteria	Marinisomatota	Marinisomatia	Marinisomatales	Marinisomataceae	Marinisoma
ASV886	Non-glacial	LacADNRUFtfb	0	Bacteria	Marinisomatota	Marinisomatia	Marinisomatales	Marinisomataceae	Marinisoma
ASV886	Non-glacial	LacADNRUFtfc	0	Bacteria	Marinisomatota	Marinisomatia	Marinisomatales	Marinisomataceae	Marinisoma
ASV886	Glacial	LacAmpRUFtf-b	0	Bacteria	Marinisomatota	Marinisomatia	Marinisomatales	Marinisomataceae	Marinisoma
ASV886	Glacial	LacAmpRUFtf-c	0	Bacteria	Marinisomatota	Marinisomatia	Marinisomatales	Marinisomataceae	Marinisoma

ASV889	Glacial	LacAmpRUFtf-b	0,002375553	Bacteria	Fusobacteriota	Fusobacteriia	Fusobacteriales	Fusobacteriaceae	Fusobacterium
ASV889	Non-glacial	LacADNRUFtfc	4,85E-05	Bacteria	Fusobacteriota	Fusobacteriia	Fusobacteriales	Fusobacteriaceae	Fusobacterium
ASV889	Baie de la Table	BdT0-2	0	Bacteria	Fusobacteriota	Fusobacteriia	Fusobacteriales	Fusobacteriaceae	Fusobacterium
ASV889	Control	Ctr-tf-IIb	0	Bacteria	Fusobacteriota	Fusobacteriia	Fusobacteriales	Fusobacteriaceae	Fusobacterium
ASV889	Non-glacial	LacADNRUFtfa	0	Bacteria	Fusobacteriota	Fusobacteriia	Fusobacteriales	Fusobacteriaceae	Fusobacterium
ASV889	Non-glacial	LacADNRUFtfb	0	Bacteria	Fusobacteriota	Fusobacteriia	Fusobacteriales	Fusobacteriaceae	Fusobacterium
ASV889	Glacial	LacAmpRUFtf-a	0	Bacteria	Fusobacteriota	Fusobacteriia	Fusobacteriales	Fusobacteriaceae	Fusobacterium
ASV889	Glacial	LacAmpRUFtf-c	0	Bacteria	Fusobacteriota	Fusobacteriia	Fusobacteriales	Fusobacteriaceae	Fusobacterium
ASV890	Glacial	LacAmpRUFtf-c	0,001065117	Bacteria	Firmicutes_A	Clostridia	Oscillospirales	Ruminococcaceae	Faecalibacterium
ASV890	Baie de la Table	BdT0-2	0	Bacteria	Firmicutes_A	Clostridia	Oscillospirales	Ruminococcaceae	Faecalibacterium
ASV890	Control	Ctr-tf-IIb	0	Bacteria	Firmicutes_A	Clostridia	Oscillospirales	Ruminococcaceae	Faecalibacterium
ASV890	Non-glacial	LacADNRUFtfa	0	Bacteria	Firmicutes_A	Clostridia	Oscillospirales	Ruminococcaceae	Faecalibacterium
ASV890	Non-glacial	LacADNRUFtfb	0	Bacteria	Firmicutes_A	Clostridia	Oscillospirales	Ruminococcaceae	Faecalibacterium
ASV890	Non-glacial	LacADNRUFtfc	0	Bacteria	Firmicutes_A	Clostridia	Oscillospirales	Ruminococcaceae	Faecalibacterium
ASV890	Glacial	LacAmpRUFtf-a	0	Bacteria	Firmicutes_A	Clostridia	Oscillospirales	Ruminococcaceae	Faecalibacterium
ASV890	Glacial	LacAmpRUFtf-b	0	Bacteria	Firmicutes_A	Clostridia	Oscillospirales	Ruminococcaceae	Faecalibacterium
ASV892	Glacial	LacAmpRUFtf-c	0,001065117	Bacteria	Deinococcota	Deinococci	Deinococcales	Deinococcaceae	Deinococcus
ASV892	Baie de la Table	BdT0-2	0	Bacteria	Deinococcota	Deinococci	Deinococcales	Deinococcaceae	Deinococcus
ASV892	Control	Ctr-tf-IIb	0	Bacteria	Deinococcota	Deinococci	Deinococcales	Deinococcaceae	Deinococcus
ASV892	Non-glacial	LacADNRUFtfa	0	Bacteria	Deinococcota	Deinococci	Deinococcales	Deinococcaceae	Deinococcus
ASV892	Non-glacial	LacADNRUFtfb	0	Bacteria	Deinococcota	Deinococci	Deinococcales	Deinococcaceae	Deinococcus
ASV892	Non-glacial	LacADNRUFtfc	0	Bacteria	Deinococcota	Deinococci	Deinococcales	Deinococcaceae	Deinococcus
ASV892	Glacial	LacAmpRUFtf-a	0	Bacteria	Deinococcota	Deinococci	Deinococcales	Deinococcaceae	Deinococcus
ASV892	Glacial	LacAmpRUFtf-b	0	Bacteria	Deinococcota	Deinococci	Deinococcales	Deinococcaceae	Deinococcus
ASV9	Glacial	LacAmpRUFtf-c	0,336916001	Bacteria	Proteobacteria	Gammaproteobacteria	Pseudomonadales	Pseudomonadaceae	Pseudomonas_E
ASV9	Glacial	LacAmpRUFtf-b	0,182485693	Bacteria	Proteobacteria	Gammaproteobacteria	Pseudomonadales	Pseudomonadaceae	Pseudomonas_E
ASV9	Non-glacial	LacADNRUFtfb	0,121580611	Bacteria	Proteobacteria	Gammaproteobacteria	Pseudomonadales	Pseudomonadaceae	Pseudomonas_E
ASV9	Non-glacial	LacADNRUFtfa	0,039308975	Bacteria	Proteobacteria	Gammaproteobacteria	Pseudomonadales	Pseudomonadaceae	Pseudomonas_E
ASV9	Non-glacial	LacADNRUFtfc	0,022550921	Bacteria	Proteobacteria	Gammaproteobacteria	Pseudomonadales	Pseudomonadaceae	Pseudomonas_E
ASV9	Glacial	LacAmpRUFtf-a	0,01659701	Bacteria	Proteobacteria	Gammaproteobacteria	Pseudomonadales	Pseudomonadaceae	Pseudomonas_E
ASV9	Control	Ctr-tf-IIb	0,007596489	Bacteria	Proteobacteria	Gammaproteobacteria	Pseudomonadales	Pseudomonadaceae	Pseudomonas_E
ASV9	Baie de la Table	BdT0-2	0	Bacteria	Proteobacteria	Gammaproteobacteria	Pseudomonadales	Pseudomonadaceae	Pseudomonas_E
ASV900	Baie de la Table	BdT0-2	0	Bacteria	Proteobacteria	Alphaproteobacteria	Azospirillales	Azospirillaceae	Azospirillum
ASV900	Control	Ctr-tf-IIb	0	Bacteria	Proteobacteria	Alphaproteobacteria	Azospirillales	Azospirillaceae	Azospirillum

ASV900	Non-glacial	LacADNRUFtfa	0	Bacteria	Proteobacteria	Alphaproteobacteria	Azospirillales	Azospirillaceae	Azospirillum
ASV900	Non-glacial	LacADNRUFtfb	0	Bacteria	Proteobacteria	Alphaproteobacteria	Azospirillales	Azospirillaceae	Azospirillum
ASV900	Non-glacial	LacADNRUFtfc	0	Bacteria	Proteobacteria	Alphaproteobacteria	Azospirillales	Azospirillaceae	Azospirillum
ASV900	Glacial	LacAmpRUFtf-a	0	Bacteria	Proteobacteria	Alphaproteobacteria	Azospirillales	Azospirillaceae	Azospirillum
ASV900	Glacial	LacAmpRUFtf-b	0	Bacteria	Proteobacteria	Alphaproteobacteria	Azospirillales	Azospirillaceae	Azospirillum
ASV900	Glacial	LacAmpRUFtf-c	0	Bacteria	Proteobacteria	Alphaproteobacteria	Azospirillales	Azospirillaceae	Azospirillum
ASV906	Glacial	LacAmpRUFtf-c	0,000823045	Bacteria	Firmicutes	Bacilli	Lactobacillales	Lactobacillaceae	Lactobacillus
ASV906	Baie de la Table	BdT0-2	0	Bacteria	Firmicutes	Bacilli	Lactobacillales	Lactobacillaceae	Lactobacillus
ASV906	Control	Ctr-tf-IIb	0	Bacteria	Firmicutes	Bacilli	Lactobacillales	Lactobacillaceae	Lactobacillus
ASV906	Non-glacial	LacADNRUFtfa	0	Bacteria	Firmicutes	Bacilli	Lactobacillales	Lactobacillaceae	Lactobacillus
ASV906	Non-glacial	LacADNRUFtfb	0	Bacteria	Firmicutes	Bacilli	Lactobacillales	Lactobacillaceae	Lactobacillus
ASV906	Non-glacial	LacADNRUFtfc	0	Bacteria	Firmicutes	Bacilli	Lactobacillales	Lactobacillaceae	Lactobacillus
ASV906	Glacial	LacAmpRUFtf-a	0	Bacteria	Firmicutes	Bacilli	Lactobacillales	Lactobacillaceae	Lactobacillus
ASV906	Glacial	LacAmpRUFtf-b	0	Bacteria	Firmicutes	Bacilli	Lactobacillales	Lactobacillaceae	Lactobacillus
ASV91	Non-glacial	LacADNRUFtfc	0,010766246	Bacteria	Proteobacteria	Gammaproteobacteria	Pseudomonadales	Pseudomonadaceae	Pseudomonas_A
ASV91	Control	Ctr-tf-IIb	0,010328981	Bacteria	Proteobacteria	Gammaproteobacteria	Pseudomonadales	Pseudomonadaceae	Pseudomonas_A
ASV91	Glacial	LacAmpRUFtf-a	0,00124578	Bacteria	Proteobacteria	Gammaproteobacteria	Pseudomonadales	Pseudomonadaceae	Pseudomonas_A
ASV91	Glacial	LacAmpRUFtf-b	0,000863838	Bacteria	Proteobacteria	Gammaproteobacteria	Pseudomonadales	Pseudomonadaceae	Pseudomonas_A
ASV91	Baie de la Table	BdT0-2	0	Bacteria	Proteobacteria	Gammaproteobacteria	Pseudomonadales	Pseudomonadaceae	Pseudomonas_A
ASV91	Non-glacial	LacADNRUFtfa	0	Bacteria	Proteobacteria	Gammaproteobacteria	Pseudomonadales	Pseudomonadaceae	Pseudomonas_A
ASV91	Non-glacial	LacADNRUFtfb	0	Bacteria	Proteobacteria	Gammaproteobacteria	Pseudomonadales	Pseudomonadaceae	Pseudomonas_A
ASV91	Glacial	LacAmpRUFtf-c	0	Bacteria	Proteobacteria	Gammaproteobacteria	Pseudomonadales	Pseudomonadaceae	Pseudomonas_A
ASV915	Control	Ctr-tf-IIb	8,01E-05	Bacteria	Bacteroidota	Bacteroidia	Flavobacteriales	Flavobacteriaceae	Algibacter
ASV915	Baie de la Table	BdT0-2	0	Bacteria	Bacteroidota	Bacteroidia	Flavobacteriales	Flavobacteriaceae	Algibacter
ASV915	Non-glacial	LacADNRUFtfa	0	Bacteria	Bacteroidota	Bacteroidia	Flavobacteriales	Flavobacteriaceae	Algibacter
ASV915	Non-glacial	LacADNRUFtfb	0	Bacteria	Bacteroidota	Bacteroidia	Flavobacteriales	Flavobacteriaceae	Algibacter
ASV915	Non-glacial	LacADNRUFtfc	0	Bacteria	Bacteroidota	Bacteroidia	Flavobacteriales	Flavobacteriaceae	Algibacter
ASV915	Glacial	LacAmpRUFtf-a	0	Bacteria	Bacteroidota	Bacteroidia	Flavobacteriales	Flavobacteriaceae	Algibacter
ASV915	Glacial	LacAmpRUFtf-b	0	Bacteria	Bacteroidota	Bacteroidia	Flavobacteriales	Flavobacteriaceae	Algibacter
ASV915	Glacial	LacAmpRUFtf-c	0	Bacteria	Bacteroidota	Bacteroidia	Flavobacteriales	Flavobacteriaceae	Algibacter
ASV92	Glacial	LacAmpRUFtf-b	0,003131411	Bacteria	Proteobacteria	Gammaproteobacteria	Pseudomonadales	HTCC2089	GCA-002727775
ASV92	Baie de la Table	BdT0-2	0,002295333	Bacteria	Proteobacteria	Gammaproteobacteria	Pseudomonadales	HTCC2089	GCA-002727775
ASV92	Non-glacial	LacADNRUFtfa	0,002217192	Bacteria	Proteobacteria	Gammaproteobacteria	Pseudomonadales	HTCC2089	GCA-002727775
ASV92	Non-glacial	LacADNRUFtfb	0,001812434	Bacteria	Proteobacteria	Gammaproteobacteria	Pseudomonadales	HTCC2089	GCA-002727775

ASV92	Control	Ctr-tf-IIb	0,001037706	Bacteria	Proteobacteria	Gammaproteobacteria	Pseudomonadales	HTCC2089	GCA-002727775
ASV92	Glacial	LacAmpRUFtf-a	0,00068317	Bacteria	Proteobacteria	Gammaproteobacteria	Pseudomonadales	HTCC2089	GCA-002727775
ASV92	Non-glacial	LacADNRUFtfc	0,000315228	Bacteria	Proteobacteria	Gammaproteobacteria	Pseudomonadales	HTCC2089	GCA-002727775
ASV92	Glacial	LacAmpRUFtf-c	0	Bacteria	Proteobacteria	Gammaproteobacteria	Pseudomonadales	HTCC2089	GCA-002727775
ASV922	Glacial	LacAmpRUFtf-a	0,000361678	Bacteria	Bacteroidota	Bacteroidia	Flavobacteriales	Flavobacteriaceae	Winogradskyella
ASV922	Baie de la Table	BdT0-2	0	Bacteria	Bacteroidota	Bacteroidia	Flavobacteriales	Flavobacteriaceae	Winogradskyella
ASV922	Control	Ctr-tf-IIb	0	Bacteria	Bacteroidota	Bacteroidia	Flavobacteriales	Flavobacteriaceae	Winogradskyella
ASV922	Non-glacial	LacADNRUFtfa	0	Bacteria	Bacteroidota	Bacteroidia	Flavobacteriales	Flavobacteriaceae	Winogradskyella
ASV922	Non-glacial	LacADNRUFtfb	0	Bacteria	Bacteroidota	Bacteroidia	Flavobacteriales	Flavobacteriaceae	Winogradskyella
ASV922	Non-glacial	LacADNRUFtfc	0	Bacteria	Bacteroidota	Bacteroidia	Flavobacteriales	Flavobacteriaceae	Winogradskyella
ASV922	Glacial	LacAmpRUFtf-b	0	Bacteria	Bacteroidota	Bacteroidia	Flavobacteriales	Flavobacteriaceae	Winogradskyella
ASV922	Glacial	LacAmpRUFtf-c	0	Bacteria	Bacteroidota	Bacteroidia	Flavobacteriales	Flavobacteriaceae	Winogradskyella
ASV927	Non-glacial	LacADNRUFtfb	0,000126449	Bacteria	Bacteroidota	Bacteroidia	Flavobacteriales	koll-22	UBA1494
ASV927	Baie de la Table	BdT0-2	0	Bacteria	Bacteroidota	Bacteroidia	Flavobacteriales	koll-22	UBA1494
ASV927	Control	Ctr-tf-IIb	0	Bacteria	Bacteroidota	Bacteroidia	Flavobacteriales	koll-22	UBA1494
ASV927	Non-glacial	LacADNRUFtfa	0	Bacteria	Bacteroidota	Bacteroidia	Flavobacteriales	koll-22	UBA1494
ASV927	Non-glacial	LacADNRUFtfc	0	Bacteria	Bacteroidota	Bacteroidia	Flavobacteriales	koll-22	UBA1494
ASV927	Glacial	LacAmpRUFtf-a	0	Bacteria	Bacteroidota	Bacteroidia	Flavobacteriales	koll-22	UBA1494
ASV927	Glacial	LacAmpRUFtf-b	0	Bacteria	Bacteroidota	Bacteroidia	Flavobacteriales	koll-22	UBA1494
ASV927	Glacial	LacAmpRUFtf-c	0	Bacteria	Bacteroidota	Bacteroidia	Flavobacteriales	koll-22	UBA1494
ASV93	Control	Ctr-tf-IIb	0,007516357	Bacteria	Verrucomicrobio	Verrucomicrobiae	Verrucomicrobiales	Akkermansiaceae	Rubritalea
ASV93	Non-glacial	LacADNRUFtfc	0,005649855	Bacteria	Verrucomicrobio	Verrucomicrobiae	Verrucomicrobiales	Akkermansiaceae	Rubritalea
ASV93	Non-glacial	LacADNRUFtfb	0,004488936	Bacteria	Verrucomicrobio	Verrucomicrobiae	Verrucomicrobiales	Akkermansiaceae	Rubritalea
ASV93	Glacial	LacAmpRUFtf-a	0,002933612	Bacteria	Verrucomicrobio	Verrucomicrobiae	Verrucomicrobiales	Akkermansiaceae	Rubritalea
ASV93	Non-glacial	LacADNRUFtfa	0,002771491	Bacteria	Verrucomicrobio	Verrucomicrobiae	Verrucomicrobiales	Akkermansiaceae	Rubritalea
ASV93	Baie de la Table	BdT0-2	0,001858127	Bacteria	Verrucomicrobio	Verrucomicrobiae	Verrucomicrobiales	Akkermansiaceae	Rubritalea
ASV93	Glacial	LacAmpRUFtf-b	0,001511716	Bacteria	Verrucomicrobio	Verrucomicrobiae	Verrucomicrobiales	Akkermansiaceae	Rubritalea
ASV93	Glacial	LacAmpRUFtf-c	0	Bacteria	Verrucomicrobio	Verrucomicrobiae	Verrucomicrobiales	Akkermansiaceae	Rubritalea
ASV934	Baie de la Table	BdT0-2	0	Bacteria	Proteobacteria	Alphaproteobacteria	Rhodobacterales	Rhodobacteraceae	Celeribacter_A
ASV934	Control	Ctr-tf-IIb	0	Bacteria	Proteobacteria	Alphaproteobacteria	Rhodobacterales	Rhodobacteraceae	Celeribacter_A
ASV934	Non-glacial	LacADNRUFtfa	0	Bacteria	Proteobacteria	Alphaproteobacteria	Rhodobacterales	Rhodobacteraceae	Celeribacter_A
ASV934	Non-glacial	LacADNRUFtfb	0	Bacteria	Proteobacteria	Alphaproteobacteria	Rhodobacterales	Rhodobacteraceae	Celeribacter_A
ASV934	Non-glacial	LacADNRUFtfc	0	Bacteria	Proteobacteria	Alphaproteobacteria	Rhodobacterales	Rhodobacteraceae	Celeribacter_A
ASV934	Glacial	LacAmpRUFtf-a	0	Bacteria	Proteobacteria	Alphaproteobacteria	Rhodobacterales	Rhodobacteraceae	Celeribacter_A

ASV934	Glacial	LacAmpRUFtf-b	0	Bacteria	Proteobacteria	Alphaproteobacteria	Rhodobacterales	Rhodobacteraceae	Celeribacter_A
ASV934	Glacial	LacAmpRUFtf-c	0	Bacteria	Proteobacteria	Alphaproteobacteria	Rhodobacterales	Rhodobacteraceae	Celeribacter_A
ASV94	Baie de la Table	BdT0-2	0,006011586	Bacteria	Bacteroidota	Bacteroidia	Flavobacteriales	Flavobacteriaceae	Leeuwenhoekiella
ASV94	Non-glacial	LacADNRUFtfa	0,000461915	Bacteria	Bacteroidota	Bacteroidia	Flavobacteriales	Flavobacteriaceae	Leeuwenhoekiella
ASV94	Non-glacial	LacADNRUFtfb	0,000189673	Bacteria	Bacteroidota	Bacteroidia	Flavobacteriales	Flavobacteriaceae	Leeuwenhoekiella
ASV94	Non-glacial	LacADNRUFtfc	9,70E-05	Bacteria	Bacteroidota	Bacteroidia	Flavobacteriales	Flavobacteriaceae	Leeuwenhoekiella
ASV94	Control	Ctr-tf-IIb	8,81E-05	Bacteria	Bacteroidota	Bacteroidia	Flavobacteriales	Flavobacteriaceae	Leeuwenhoekiella
ASV94	Glacial	LacAmpRUFtf-a	0	Bacteria	Bacteroidota	Bacteroidia	Flavobacteriales	Flavobacteriaceae	Leeuwenhoekiella
ASV94	Glacial	LacAmpRUFtf-b	0	Bacteria	Bacteroidota	Bacteroidia	Flavobacteriales	Flavobacteriaceae	Leeuwenhoekiella
ASV94	Glacial	LacAmpRUFtf-c	0	Bacteria	Bacteroidota	Bacteroidia	Flavobacteriales	Flavobacteriaceae	Leeuwenhoekiella
ASV943	Baie de la Table	BdT0-2	0	Bacteria	Planctomycetota	Planctomycetes	Isosphaerales	Isosphaeraceae	Paludisphaera
ASV943	Control	Ctr-tf-IIb	0	Bacteria	Planctomycetota	Planctomycetes	Isosphaerales	Isosphaeraceae	Paludisphaera
ASV943	Non-glacial	LacADNRUFtfa	0	Bacteria	Planctomycetota	Planctomycetes	Isosphaerales	Isosphaeraceae	Paludisphaera
ASV943	Non-glacial	LacADNRUFtfb	0	Bacteria	Planctomycetota	Planctomycetes	Isosphaerales	Isosphaeraceae	Paludisphaera
ASV943	Non-glacial	LacADNRUFtfc	0	Bacteria	Planctomycetota	Planctomycetes	Isosphaerales	Isosphaeraceae	Paludisphaera
ASV943	Glacial	LacAmpRUFtf-a	0	Bacteria	Planctomycetota	Planctomycetes	Isosphaerales	Isosphaeraceae	Paludisphaera
ASV943	Glacial	LacAmpRUFtf-b	0	Bacteria	Planctomycetota	Planctomycetes	Isosphaerales	Isosphaeraceae	Paludisphaera
ASV943	Glacial	LacAmpRUFtf-c	0	Bacteria	Planctomycetota	Planctomycetes	Isosphaerales	Isosphaeraceae	Paludisphaera
ASV953	Baie de la Table	BdT0-2	0	Bacteria	Acidobacteriota	Luteitaleia	Luteitaleales	SCN-69-37	SCN-69-37
ASV953	Control	Ctr-tf-IIb	0	Bacteria	Acidobacteriota	Luteitaleia	Luteitaleales	SCN-69-37	SCN-69-37
ASV953	Non-glacial	LacADNRUFtfa	0	Bacteria	Acidobacteriota	Luteitaleia	Luteitaleales	SCN-69-37	SCN-69-37
ASV953	Non-glacial	LacADNRUFtfb	0	Bacteria	Acidobacteriota	Luteitaleia	Luteitaleales	SCN-69-37	SCN-69-37
ASV953	Non-glacial	LacADNRUFtfc	0	Bacteria	Acidobacteriota	Luteitaleia	Luteitaleales	SCN-69-37	SCN-69-37
ASV953	Glacial	LacAmpRUFtf-a	0	Bacteria	Acidobacteriota	Luteitaleia	Luteitaleales	SCN-69-37	SCN-69-37
ASV953	Glacial	LacAmpRUFtf-b	0	Bacteria	Acidobacteriota	Luteitaleia	Luteitaleales	SCN-69-37	SCN-69-37
ASV953	Glacial	LacAmpRUFtf-c	0	Bacteria	Acidobacteriota	Luteitaleia	Luteitaleales	SCN-69-37	SCN-69-37
ASV966	Baie de la Table	BdT0-2	0	Bacteria	Bacteroidota	Bacteroidia	Cytophagales	Spirosomaceae	Spirosoma
ASV966	Control	Ctr-tf-IIb	0	Bacteria	Bacteroidota	Bacteroidia	Cytophagales	Spirosomaceae	Spirosoma
ASV966	Non-glacial	LacADNRUFtfa	0	Bacteria	Bacteroidota	Bacteroidia	Cytophagales	Spirosomaceae	Spirosoma
ASV966	Non-glacial	LacADNRUFtfb	0	Bacteria	Bacteroidota	Bacteroidia	Cytophagales	Spirosomaceae	Spirosoma
ASV966	Non-glacial	LacADNRUFtfc	0	Bacteria	Bacteroidota	Bacteroidia	Cytophagales	Spirosomaceae	Spirosoma
ASV966	Glacial	LacAmpRUFtf-a	0	Bacteria	Bacteroidota	Bacteroidia	Cytophagales	Spirosomaceae	Spirosoma
ASV966	Glacial	LacAmpRUFtf-b	0	Bacteria	Bacteroidota	Bacteroidia	Cytophagales	Spirosomaceae	Spirosoma
ASV966	Glacial	LacAmpRUFtf-c	0	Bacteria	Bacteroidota	Bacteroidia	Cytophagales	Spirosomaceae	Spirosoma

ASV969	Glacial	LacAmpRUFtf-b	0,000971817	Bacteria	Verrucomicrobio	Verrucomicrobiae	Opitutales	Opitutaceae	UBA5691
ASV969	Baie de la Table	BdT0-2	0	Bacteria	Verrucomicrobio	Verrucomicrobiae	Opitutales	Opitutaceae	UBA5691
ASV969	Control	Ctr-tf-IIb	0	Bacteria	Verrucomicrobio	Verrucomicrobiae	Opitutales	Opitutaceae	UBA5691
ASV969	Non-glacial	LacADNRUFtfa	0	Bacteria	Verrucomicrobio	Verrucomicrobiae	Opitutales	Opitutaceae	UBA5691
ASV969	Non-glacial	LacADNRUFtfb	0	Bacteria	Verrucomicrobio	Verrucomicrobiae	Opitutales	Opitutaceae	UBA5691
ASV969	Non-glacial	LacADNRUFtfc	0	Bacteria	Verrucomicrobio	Verrucomicrobiae	Opitutales	Opitutaceae	UBA5691
ASV969	Glacial	LacAmpRUFtf-a	0	Bacteria	Verrucomicrobio	Verrucomicrobiae	Opitutales	Opitutaceae	UBA5691
ASV969	Glacial	LacAmpRUFtf-c	0	Bacteria	Verrucomicrobio	Verrucomicrobiae	Opitutales	Opitutaceae	UBA5691
ASV971	Baie de la Table	BdT0-2	0	Bacteria	Bacteroidota	Bacteroidia	Flavobacteriales	koll-22	BRH-c54
ASV971	Control	Ctr-tf-IIb	0	Bacteria	Bacteroidota	Bacteroidia	Flavobacteriales	koll-22	BRH-c54
ASV971	Non-glacial	LacADNRUFtfa	0	Bacteria	Bacteroidota	Bacteroidia	Flavobacteriales	koll-22	BRH-c54
ASV971	Non-glacial	LacADNRUFtfb	0	Bacteria	Bacteroidota	Bacteroidia	Flavobacteriales	koll-22	BRH-c54
ASV971	Non-glacial	LacADNRUFtfc	0	Bacteria	Bacteroidota	Bacteroidia	Flavobacteriales	koll-22	BRH-c54
ASV971	Glacial	LacAmpRUFtf-a	0	Bacteria	Bacteroidota	Bacteroidia	Flavobacteriales	koll-22	BRH-c54
ASV971	Glacial	LacAmpRUFtf-b	0	Bacteria	Bacteroidota	Bacteroidia	Flavobacteriales	koll-22	BRH-c54
ASV971	Glacial	LacAmpRUFtf-c	0	Bacteria	Bacteroidota	Bacteroidia	Flavobacteriales	koll-22	BRH-c54
ASV972	Baie de la Table	BdT0-2	0	Bacteria	Proteobacteria	Alphaproteobacteria	Rhizobiales	Xanthobacteraceae	Afipia
ASV972	Control	Ctr-tf-IIb	0	Bacteria	Proteobacteria	Alphaproteobacteria	Rhizobiales	Xanthobacteraceae	Afipia
ASV972	Non-glacial	LacADNRUFtfa	0	Bacteria	Proteobacteria	Alphaproteobacteria	Rhizobiales	Xanthobacteraceae	Afipia
ASV972	Non-glacial	LacADNRUFtfb	0	Bacteria	Proteobacteria	Alphaproteobacteria	Rhizobiales	Xanthobacteraceae	Afipia
ASV972	Non-glacial	LacADNRUFtfc	0	Bacteria	Proteobacteria	Alphaproteobacteria	Rhizobiales	Xanthobacteraceae	Afipia
ASV972	Glacial	LacAmpRUFtf-a	0	Bacteria	Proteobacteria	Alphaproteobacteria	Rhizobiales	Xanthobacteraceae	Afipia
ASV972	Glacial	LacAmpRUFtf-b	0	Bacteria	Proteobacteria	Alphaproteobacteria	Rhizobiales	Xanthobacteraceae	Afipia
ASV972	Glacial	LacAmpRUFtf-c	0	Bacteria	Proteobacteria	Alphaproteobacteria	Rhizobiales	Xanthobacteraceae	Afipia
ASV974	Baie de la Table	BdT0-2	0	Bacteria	Bacteroidota	Bacteroidia	Sphingobacteriales	Sphingobacteriaceae	Pedobacter
ASV974	Control	Ctr-tf-IIb	0	Bacteria	Bacteroidota	Bacteroidia	Sphingobacteriales	Sphingobacteriaceae	Pedobacter
ASV974	Non-glacial	LacADNRUFtfa	0	Bacteria	Bacteroidota	Bacteroidia	Sphingobacteriales	Sphingobacteriaceae	Pedobacter
ASV974	Non-glacial	LacADNRUFtfb	0	Bacteria	Bacteroidota	Bacteroidia	Sphingobacteriales	Sphingobacteriaceae	Pedobacter
ASV974	Non-glacial	LacADNRUFtfc	0	Bacteria	Bacteroidota	Bacteroidia	Sphingobacteriales	Sphingobacteriaceae	Pedobacter
ASV974	Glacial	LacAmpRUFtf-a	0	Bacteria	Bacteroidota	Bacteroidia	Sphingobacteriales	Sphingobacteriaceae	Pedobacter
ASV974	Glacial	LacAmpRUFtf-b	0	Bacteria	Bacteroidota	Bacteroidia	Sphingobacteriales	Sphingobacteriaceae	Pedobacter
ASV974	Glacial	LacAmpRUFtf-c	0	Bacteria	Bacteroidota	Bacteroidia	Sphingobacteriales	Sphingobacteriaceae	Pedobacter
ASV98	Baie de la Table	BdT0-2	0,020876599	Archaea	Thermoplasmato	MGII	MGII	MGIIA	UBA562
ASV98	Non-glacial	LacADNRUFtfa	0,000646681	Archaea	Thermoplasmato	MGII	MGII	MGIIA	UBA562

ASV98	Non-glacial	LacADNRUFtfb	0,000316122	Archaea	Thermoplasmato	MGII	MGII	MGIIA	UBA562
ASV98	Control	Ctr-tf-IIb	0	Archaea	Thermoplasmato	MGII	MGII	MGIIA	UBA562
ASV98	Non-glacial	LacADNRUFtfc	0	Archaea	Thermoplasmato	MGII	MGII	MGIIA	UBA562
ASV98	Glacial	LacAmpRUFtf-a	0	Archaea	Thermoplasmato	MGII	MGII	MGIIA	UBA562
ASV98	Glacial	LacAmpRUFtf-b	0	Archaea	Thermoplasmato	MGII	MGII	MGIIA	UBA562
ASV98	Glacial	LacAmpRUFtf-c	0	Archaea	Thermoplasmato	MGII	MGII	MGIIA	UBA562
ASV999	Baie de la Table	BdT0-2	0	Bacteria	Myxococcota	Polyangia	Polyangiales	Polyangiaceae	Chondromyces
ASV999	Control	Ctr-tf-IIb	0	Bacteria	Myxococcota	Polyangia	Polyangiales	Polyangiaceae	Chondromyces
ASV999	Non-glacial	LacADNRUFtfa	0	Bacteria	Myxococcota	Polyangia	Polyangiales	Polyangiaceae	Chondromyces
ASV999	Non-glacial	LacADNRUFtfb	0	Bacteria	Myxococcota	Polyangia	Polyangiales	Polyangiaceae	Chondromyces
ASV999	Non-glacial	LacADNRUFtfc	0	Bacteria	Myxococcota	Polyangia	Polyangiales	Polyangiaceae	Chondromyces
ASV999	Glacial	LacAmpRUFtf-a	0	Bacteria	Myxococcota	Polyangia	Polyangiales	Polyangiaceae	Chondromyces
ASV999	Glacial	LacAmpRUFtf-b	0	Bacteria	Myxococcota	Polyangia	Polyangiales	Polyangiaceae	Chondromyces
ASV999	Glacial	LacAmpRUFtf-c	0	Bacteria	Myxococcota	Polyangia	Polyangiales	Polyangiaceae	Chondromyces
