# Supplementary material for: Response of marine microbes to iron contained in colloids of glacial origin: a Kerguelen Island case study
Source: ISME Commun. 2025 Jun 3;5(1):ycaf093. doi: 10.1093/ismeco/ycaf093 (PMC12445661; doi:10.1093/ismeco/ycaf093)
Supplement: UPDATED_SOURCEThoppil_ISMECom_Suppl_Revised_v1_ycaf093 [file updated_sourcethoppil_ismecom_suppl_revised_v1_ycaf093.zip › Table_S5.pdf]

Table S5. Summary statistics and taxonomic assignment of the metagenome-assembled genomes (MAGs)

Bins	Total_length	num_contigs	N50	GC_content	Completion	Redundancy	Domain	Phylum	Class	Order	Family	Genus	Species
Bin_123	3699636	14	519148	55,42702496	100	0	Bacteria	Proteobacteria	Alphaproteobacteria	Parvibaculales	Parvibaculaceae	Mf105b01	
Bin_134	2384009	28	108624	52,16745447	100	1,408450704	Bacteria	Proteobacteria	Alphaproteobacteria	Caulobacterales			
Bin_20_sub	7573391	242	54705	64,47053067	100	1,408450704	Bacteria	Proteobacteria	Alphaproteobacteria	Rhizobiales	Xanthobacteraceae	Bradyrhizobium	
Bin_30	3423924	74	75833	55,36310789	100	0	Bacteria	Proteobacteria	Alphaproteobacteria	Rhodobacterales	Rhodobacteraceae	Sulfitobacter	
Bin_32	5607410	288	30848	69,37479564	100	7,042253521	Bacteria	Proteobacteria	Alphaproteobacteria	Rhizobiales	Beijerinckiaceae	Methylobacterium	
maxbin_051_	4398813	288	75213	49,10866903	100	23,94366197	Bacteria	Proteobacteria					
maxbin_117	4094116	587	8915	43,12350169	100	9,85915493	Bacteria	Proteobacteria	Gammaproteobacteria	Pseudomonadales	Marinomonadaceae	Marinomonas	
maxbin_138	2933189	368	11396	57,98950614	100	4,225352113	Bacteria	Proteobacteria	Alphaproteobacteria	Rhodobacterales	Rhodobacteraceae		
Bin_115	3140301	121	50675	37,37133337	98,5915493	18,30985915	Bacteria	Proteobacteria	Gammaproteobacteria	Enterobacterales	Alteromonadaceae	Colwellia	
Bin_136	3100081	41	138461	48,58980581	98,5915493	0	Bacteria	Proteobacteria	Gammaproteobacteria	Pseudomonadales	HTCC2089	UBA4421	UBA4421 sp009886205
Bin_152	3260242	144	39943	33,12083668	98,5915493	2,816901408	Bacteria	Bacteroidota	Bacteroidia	Flavobacteriales	Flavobacteriaceae	Flavobacterium	
Bin_154	3751445	122	54366	47,72303689	98,5915493	0	Bacteria	Proteobacteria	Gammaproteobacteria	Pseudomonadales	HTCC2089	UBA4582	UBA4582 sp012960755
Bin_55	4830341	138	56955	35,06851247	98,5915493	1,408450704	Bacteria	Bacteroidota	Bacteroidia	Cytophagales	Spirosomaceae	Arcicella	Arcicella sp014376355
Bin_79	2405432	92	47493	38,15116058	98,5915493	2,816901408	Bacteria	Bacteroidota	Bacteroidia	NS11-12g	UBA9320	UBA9320	UBA9320 sp003484585
maxbin_024	6639489	224	51404	59,55073514	98,5915493	8,450704225	Bacteria	Proteobacteria	Gammaproteobacteria	Pseudomonadales	Pseudomonadaceae	Pseudomonas	
maxbin_031	2176607	195	19500	36,42594731	98,5915493	22,53521127	Bacteria	Bacteroidota	Bacteroidia	Flavobacteriales	Flavobacteriaceae	UBA7428	UBA7428 sp003023645
maxbin_042	6843397	354	32648	65,95646276	98,5915493	5,633802817	Bacteria	Proteobacteria	Gammaproteobacteria	Burkholderiales	Burkholderiaceae	Comamonas	
maxbin_043	3268357	117	65677	58,15464394	98,5915493	7,042253521	Bacteria	Actinobacteriota	Actinomycetia	Propionibacteriales	Propionibacteriaceae	Cutibacterium	Cutibacterium acnes
maxbin_053	3194216	171	43600	48,03879352	98,5915493	15,49295775	Bacteria	Proteobacteria	Gammaproteobacteria	Pseudomonadales	Porticoccaceae	HTCC2207	
maxbin_061	1485656	124	18465	46,99946827	98,5915493	2,816901408	Bacteria	Actinobacteriota	Actinomycetia	Actinomycetales	Microbacteriaceae	Rhodoluna	Rhodoluna sp903832875
maxbin_086	1842206	170	20888	52,94613475	98,5915493	5,633802817	Bacteria	Actinobacteriota	Actinomycetia	Mycobacteriales	Mycobacteriaceae	Lawsonella	Lawsonella clevelandensis
maxbin_134	2989733	44	139429	58,6517253	98,5915493	4,225352113	Bacteria	Proteobacteria	Alphaproteobacteria	Rhodobacterales	Rhodobacteraceae	Thalassobacter	Thalassobacter stenotrophicus
Bin_130	3051140	78	66072	61,99842921	97,18309859	0	Bacteria	Actinobacteriota	Acidimicrobiia	Acidimicrobiales	Ilumatobacteraceae	Ilumatobacter	Ilumatobacter sp002711735
Bin_21	1998408	35	139388	32,62272014	97,18309859	0	Bacteria	Bacteroidota	Bacteroidia	Flavobacteriales	Flavobacteriaceae	GCA-002733185	GCA-002733185 sp004213605
Bin_56	2449575	37	121599	32,67011596	97,18309859	2,816901408	Bacteria						
Bin_8	2133026	185	16081	38,64623499	97,18309859	1,408450704	Bacteria	Proteobacteria	Alphaproteobacteria	Sphingomonadales	Emcibacteraceae	UBA4441	UBA4441 sp002390425
maxbin_012	2385458	15	277737	39,94527692	97,18309859	0	Bacteria	Proteobacteria	Gammaproteobacteria	Nitrosococcales	Methylophagaceae	GCA-002733105	GCA-002733105 sp011053005
maxbin_018_	2569960	353	11611	42,89267619	97,18309859	23,94366197	Bacteria	Proteobacteria	Gammaproteobacteria	Pseudomonadales	Porticoccaceae	HTCC2207	HTCC2207 sp002313335
maxbin_021	3808381	76	97446	47,24332931	97,18309859	0	Bacteria	Proteobacteria	Gammaproteobacteria	Pseudomonadales	Spongiibacteraceae	Oceanicoccus	Oceanicoccus sp000169075
maxbin_026	3383095	168	33994	52,96961611	97,18309859	7,042253521	Bacteria	Proteobacteria	Alphaproteobacteria	Rhodobacterales	Rhodobacteraceae		
maxbin_079	2406991	55	86361	55,86256602	97,18309859	0	Bacteria	Proteobacteria	Gammaproteobacteria	Pseudomonadales	Pseudohongiellaceae	OM182	OM182 sp001438145
maxbin_152	4956636	360	23273	50,40101408	97,18309859	8,450704225	Bacteria	Proteobacteria	Gammaproteobacteria	Pseudomonadales	Nitrincolaceae		
Bin_86	1933509	60	52806	42,02223843	95,77464789	12,67605634	Bacteria	Bacteroidota	Bacteroidia	Flavobacteriales	Schleiferiaceae	UBA10364	UBA10364 sp003487785
maxbin_025	2253162	112	39535	48,95960442	95,77464789	1,408450704	Bacteria	Proteobacteria	Gammaproteobacteria	Pseudomonadales	Porticoccaceae	HTCC2207	HTCC2207 sp002335945
maxbin_028_	2743491	242	23788	44,67045474	95,77464789	2,816901408	Bacteria	Proteobacteria	Gammaproteobacteria	Pseudomonadales	Porticoccaceae	HTCC2207	HTCC2207 sp002313335
Bin_125	4594781	146	52032	54,30551335	94,36619718	4,225352113	Bacteria	Verrucomicrobi	Verrucomicrobiae	Verrucomicrobiales	Akkermansiaceae	SW10	SW10 sp002172625
Bin_168_sub	3975123	198	29738	58,28165728	94,36619718	1,408450704	Bacteria	Proteobacteria	Alphaproteobacteria	Rhodobacterales	Rhodobacteraceae	Roseovarius	Roseovarius sp900313005
Bin_18	3850545	131	95649	49,80779739	94,36619718	5,633802817	Bacteria	Proteobacteria	Gammaproteobacteria	Burkholderiales	Burkholderiaceae	Polaromonas	Polaromonas vacuolata
Bin_33	2255024	228	14109	52,94699126	94,36619718	4,225352113	Bacteria	Proteobacteria	Gammaproteobacteria	Pseudomonadales	Halieaceae	Luminiphilus	Luminiphilus sp003331335
Bin_7_sub	3730189	426	11844	50,22045122	94,36619718	0	Bacteria	Proteobacteria	Alphaproteobacteria	Rhodobacterales	Rhodobacteraceae	Halocynthiibacter	Halocynthiibacter arcticus
Bin_13	1622866	104	26572	29,32609884	92,95774648	1,408450704	Bacteria	Bacteroidota	Bacteroidia	Flavobacteriales	Flavobacteriaceae	MAG-121220-bin8	MAG-121220-bin8 sp902510655
Bin_146	2462627	85	51465	37,57674981	92,95774648	1,408450704	Bacteria	Bacteroidota	Bacteroidia	Flavobacteriales	Crocinitomicaceae	UBA952	
Bin_155	6259713	265	37427	65,45149471	92,95774648	7,042253521	Bacteria	Proteobacteria	Gammaproteobacteria	Burkholderiales	Burkholderiaceae		
Bin_169	3709557	164	44047	52,05582486	92,95774648	4,225352113	Bacteria	Verrucomicrobi	Verrucomicrobiae	Verrucomicrobiales	Akkermansiaceae	SW10	
maxbin_014_	3633655	55	112889	33,87749068	92,95774648	1,408450704	Bacteria	Bacteroidota	Bacteroidia	Flavobacteriales	Flavobacteriaceae	Flavobacterium	
maxbin_035_	2485699	210	18863	49,22506487	92,95774648	2,816901408	Bacteria	Bacteroidota	Bacteroidia	Flavobacteriales	UA16	UA16	UA16 sp002390615
maxbin_046_	2098423	118	49291	33,33646415	91,54929577	16,90140845	Bacteria	Bacteroidota	Bacteroidia	Flavobacteriales	Flavobacteriaceae	Polaribacter	
maxbin_126	4947367	647	18334	43,41776583	91,54929577	16,90140845	Bacteria	Proteobacteria	Gammaproteobacteria	Enterobacterales	Alteromonadaceae	Glaciecola	Glaciecola sp000155775
maxbin_143	4287199	44	222618	62,19031383	91,54929577	2,816901408	Bacteria	Proteobacteria	Gammaproteobacteria	Pseudomonadales	Pseudomonadaceae	Pseudomonas	
Bin_161	3317245	164	42112	53,27677303	90,14084507	1,408450704	Bacteria	Proteobacteria	Alphaproteobacteria	Rhodobacterales	Rhodobacteraceae	Yoonia	Yoonia ponticola
Bin_96	1998193	292	9862	34,24794372	90,14084507	46,47887324	Bacteria	Proteobacteria	Gammaproteobacteria	SAR86	D2472	MED-G78	MED-G78 sp902509965
maxbin_090	3227616	39	130124	62,89388222	90,14084507	2,816901408	Bacteria	Proteobacteria	Alphaproteobacteria	Rhizobiales	Hyphomicrobiaceae	Hyphomicrobium	Hyphomicrobium sp009026145
maxbin_095	4816603	275	31168	40,91765266	90,14084507	2,816901408	Bacteria	Proteobacteria	Gammaproteobacteria	Pseudomonadales	Marinomonadaceae	Marinomonas	
maxbin_140	2320521	239	14463	52,49180405	90,14084507	2,816901408	Bacteria						
Bin_58	2697840	289	11451	41,66006041	88,73239437	1,408450704	Bacteria	Bacteroidota	Bacteroidia	Flavobacteriales	Luteibaculaceae	Luteibaculum	Luteibaculum oceani
Bin_93_sub	2215289	376	6528	31,83762382	88,73239437	5,633802817	Bacteria	Bacteroidota	Bacteroidia	Flavobacteriales	Flavobacteriaceae	SCGC-AAA160-P02	SCGC-AAA160-P02 sp000383355

maxbin_039_	1980740	109	38758	33,47607957	88,73239437	7,042253521	Bacteria	Bacteroidota	Bacteroidia	Flavobacteriales	Flavobacteriaceae	MS024-2A	MS024-2A sp009886625
maxbin_099	2768781	601	5494	57,52972128	88,73239437	1,408450704	Bacteria	Proteobacteria	Alphaproteobacteria	Sphingomonadales	Sphingomonadaceae	Altererythrobacter	Altererythrobacter sp003149575
maxbin_110_	2552055	623	4526	44,69228086	88,73239437	15,49295775	Bacteria	Proteobacteria	Gammaproteobacteria	Enterobacterales	Vibrionaceae	Vibrio	
maxbin_120_	2200372	504	4982	29,02063845	88,73239437	1,408450704	Bacteria	Campylobacterc	Campylobacteria	Campylobacterales	Arcobacteraceae		
Bin_156	2662085	86	55509	33,49424259	87,32394366	2,816901408	Bacteria	Bacteroidota	Bacteroidia	Flavobacteriales	Flavobacteriaceae	Flavobacterium	
Bin_44_sub	5944035	740	10027	62,47029382	87,32394366	1,408450704	Bacteria	Proteobacteria	Gammaproteobacteria	Burkholderiales	Burkholderiaceae	Janthinobacterium	
Bin_84	2137834	142	30551	36,77790286	87,32394366	0	Bacteria	Proteobacteria	Alphaproteobacteria	Rhodobacterales	Rhodobacteraceae	Amylibacter	Amylibacter sp000153745
maxbin_142	3338143	225	23877	62,00267433	87,32394366	2,816901408	Bacteria	Proteobacteria	Alphaproteobacteria	Rhodobacterales	Rhodobacteraceae		
maxbin_148_	1700421	497	3480	41,27972492	87,32394366	25,35211268	Bacteria	Bacteroidota	Bacteroidia	Flavobacteriales	Flavobacteriaceae	UBA7446	UBA7446 sp002470745
Bin_160_sub	1930393	409	4856	38,04707236	85,91549296	19,71830986	Bacteria	Proteobacteria	Gammaproteobacteria	SAR86	D2472	D2472	D2472 sp002469975
Bin_91	2645982	444	6364	50,90388197	85,91549296	25,35211268	Bacteria	Proteobacteria	Alphaproteobacteria	Rhodobacterales	Rhodobacteraceae	Ascidiaceihabitans	Ascidiaceihabitans sp002478745
maxbin_044_	4285797	135	213091	33,11336825	85,91549296	7,042253521	Bacteria	Bacteroidota	Bacteroidia	Flavobacteriales	Flavobacteriaceae	Flavobacterium	
maxbin_129_	3195379	321	20129	42,02827367	85,91549296	23,94366197	Bacteria	Proteobacteria	Gammaproteobacteria				
maxbin_133_	2968379	185	42179	54,84746687	85,91549296	8,450704225	Bacteria	Proteobacteria	Alphaproteobacteria	Rhodobacterales	Rhodobacteraceae	Nereida	Nereida ignava
Bin_100	1984424	125	21350	35,86230031	84,50704225	1,408450704	Bacteria	Bacteroidota	Bacteroidia	Flavobacteriales	Flavobacteriaceae	Hel1-33-131	Hel1-33-131 sp001735745
Bin_19	2090849	363	6692	50,53454316	84,50704225	5,633802817	Bacteria	Proteobacteria	Alphaproteobacteria	Rhodobacterales	Rhodobacteraceae		
Bin_80_sub	2810766	189	26890	55,70328694	84,50704225	2,816901408	Bacteria	Proteobacteria	Alphaproteobacteria	Rhodobacterales	Rhodobacteraceae		
Bin_95_sub	7924933	1099	8312	44,47822071	84,50704225	16,90140845	Bacteria	Proteobacteria	Gammaproteobacteria	Pseudomonadales	Nitrincolaceae	Neptuniibacter	Neptuniibacter caesariensis
Bin_47	2214231	340	7484	42,60396424	83,09859155	2,816901408	Bacteria	Verrucomicrobi	Verrucomicrobiae	Opitutales	MB11C04	MB11C04	MB11C04 sp902580225
maxbin_155	3944048	514	10219	45,70728731	83,09859155	1,408450704	Bacteria	Bacteroidota	Bacteroidia	Flavobacteriales	Flavobacteriaceae	Muricauda	Muricauda taeanensis
Bin_135	4119586	383	14476	40,94133829	81,69014085	0	Bacteria	Bacteroidota	Bacteroidia	Cytophagales	Cyclobacteriaceae	Ekhidna	Ekhidna sp006969745
Bin_153_sub	3448005	84	109330	53,29157061	81,69014085	1,408450704	Bacteria	Proteobacteria	Gammaproteobacteria	Pseudomonadales	HTCC2089	UBA9926	UBA9926 sp003451575
maxbin_154_	2259681	403	7018	40,93646317	81,69014085	7,042253521	Bacteria	Bacteroidota	Rhodothermia	Balneolales	Balneolaceae	UBA1275	UBA1275 sp002457365
Bin_109_sub	2561419	124	44264	48,3043552	80,28169014	4,225352113	Bacteria	Proteobacteria	Gammaproteobacteria	Pseudomonadales	Porticoccaceae	HTCC2207	HTCC2207 sp002335945
Bin_63_sub	2121901	155	21228	34,8127175	74,64788732	2,816901408	Bacteria	Bacteroidota	Bacteroidia	Flavobacteriales	Flavobacteriaceae	Hel1-33-131	Hel1-33-131 sp001735745
maxbin_157	2073715	393	6514	57,98934128	71,83098592	0	Bacteria	Proteobacteria	Alphaproteobacteria	Puniceispirillales	Puniceispirillaceae	UBA8309	
Bin_106	2086015	265	12720	30,41808907	70,42253521	7,042253521	Bacteria	Bacteroidota	Bacteroidia	Flavobacteriales	Flavobacteriaceae	SGZJ01	SGZJ01 sp004213995
