# Supplementary material for: Response of marine microbes to iron contained in colloids of glacial origin: a Kerguelen Island case study
Source: ISME Commun. 2025 Jun 3;5(1):ycaf093. doi: 10.1093/ismeco/ycaf093 (PMC12445661; doi:10.1093/ismeco/ycaf093)
Supplement: UPDATED_SOURCEThoppil_ISMECom_Suppl_Revised_v1_ycaf093 [file updated_sourcethoppil_ismecom_suppl_revised_v1_ycaf093.zip › Thoppil_ISMECom_Suppl_Revised.pdf]

Supplementary Information for

"Response of marine microbes to iron contained in colloids of glacial origin: A Kerguelen Island case study"

Rhea Thoppil[1], Stéphane Blain[1], Rui Zhang[1], Audrey Guéneuguès[1], Olivier Crispi[1], Philippe Catala[1], Barbara Marie[1], Ingrid Obernosterer[1]

1 CNRS, Sorbonne Université, Laboratoire d'Océanographie Microbienne, LOMIC, F-66650 Banyuls/mer, France.

This file includes:

Supplementary Methods
Supplementary Figures
Supplementary Table 1, 2, 4 and 6

Other supplementary material for this manuscript includes the following:

Supplementary Table 3 and 5 in pdf format
Supplementary References

Supplementary Methods

Inorganic nutrient analyses

For nitrate (NO_3^-) and phosphate (PO_4^{3-}) lake water was collected by a 50 mL syringe that was directly connected to the spigot of the Niskin bottle. The samples were drawn through a 0.45 µm Uptidisc (Whatman) adapted for the syringe. The filtered samples were poisoned with mercuric chloride ($HgCl_2$, 20 mg L^{-1} final concentration) and stored in the dark until analysed in the home lab. Concentrations of NO_3^- and PO_4^{3-} were determined with a segmented flow analyser (Skalar) equipped with a colorimetric detection using methods described previously (1)

Dissolved organic carbon

Samples (10 mL, in triplicate) for dissolved organic carbon (DOC) analyses of lake water and aged seawater were filtered through two combusted (450 ◦C, 4 h) GF/F filters directly into pre-combusted glass ampoules and acidified with H_3PO_4 (final pH = 2). The sealed glass ampoules were stored in the dark at room temperature until analysis. DOC measurements were performed on a Shimadzu TOC- V-CSH (2). Prior to injection, DOC samples were sparged with CO2 -free air for 6 min to remove inorganic carbon. 100 µL of each of the sample replicates were injected in triplicate and the analytical precision was 2%. Standards were prepared with acetanilide. Consensus reference materials provided in sealed glass ampoules were injected every 12 to 17 samples to ensure stable operating conditions.

Dissolved Fe determination

Samples (30 ml) were collected in trace metal clean low HDPE bottles and acidified with 30µL of HNO_3 (Merck Suprapur grade). The sample were stored at room temperature until analysis in the laboratory a few months later. The DFe concentrations were determined by Q-ICPMS (Agilent). The accuracy (5%) was determined using the Standard Reference Material 1643 (NIST) containing trace elements in fresh water.

Enumeration of non-phototrophic prokaryotes

For the enumeration of non-phototrophic prokaryotes of the 2 freshwater systems, unfiltered lake water (1.44 mL) was fixed with glutaraldehyde grade I 25% (1% final concentration), incubated for 30 min at 4°C, and then stored at -20°C (roughly 1 week) and at -80°C until analysis. For microbial incubation experiments, glutaraldehyde fixed samples were incubated for 30 min at 4°C and then at -80°C until analysis. Prior to flow cytometric analyses, samples were thawed at room temperature. Counts were performed on a FACSCanto II flow cytometer (Becton Dickinson) equipped with 3 air-cooled lasers: blue (argon 488 nm), red (633 nm) and violet (407 nm). For the enumeration of non- autofluorescent cells, mainly heterotrophic prokaryotes, cells were stained with SYBR Green I (Invitrogen – Molecular Probes) at 0.025% (vol/vol) final concentration for 15 min at room temperature in the dark. Stained prokaryotic cells were discriminated and enumerated ac-cording to their right-angle light scatter (SSC) and green fluorescence at 530/30 nm. Fluorescent beads (1.002 µm; Polysciences Europe) were systematically added to each

analysed sample as an internal standard. The cell abundance was determined from the flow rate, which was calculated with TruCount beads (BD biosciences).

Microbial community composition

The filter units were thawed and closed with a sterile pipette tip end at the outflow, 425 µL lysis buffer were added per sample (40 mM EDTA, 50 mM Tris and 0.75 M sucrose) and three freeze-thaw cycles were performed with liquid nitrogen and a water bath at 65 ◦C. Subsequently, 25 µL of freshly prepared lysozyme solution were added (2 mg mL-1 final concentration), the filter units were placed in a rotary mixer and incubated at 37 ◦C during 45 min, and then 8 µL of proteinase K solution (0.2 mg mL- 1final concentration) and sodium dodecyl sulphate (SDS) (1%) were added and maintained at 55 ◦C with gentle agitation every 10 min for 2 h. PCR amplification was performed under the following conditions: an initial denaturation step of 95 ◦C for 3 min, followed by 30 cycles of denaturation at 95°C for 45 s, annealing at 50°C for 45 s, and extension at 68°C for 90 s, and a final elongation step at 68°C for 5 min.

Amplicon sequencing variants (ASVs) were obtained with *DADA2* v1.24 (3) based on the following parameters: *truncLen=c(200,200), maxN=0, maxEE=c(2,2), truncQ=2, rm.phix=TRUE*. The pipeline consisted of certain steps: filter and trim, dereplication, sample inference, merge paired reads, sequence table construction and chimera removal. A mock community with known reference sequences of bacterial strains were evaluated to check the accuracy of *DADA2* and the residual error rate of the analysis was 0%, ensuring that the ASVs identified by *DADA2* present in the mock community exactly matched the reference sequences of expected bacterial strains. A total of 2238 ASVs were acquired from the 8 samples collected (including the only control and initial community acting as inoculum). The number of reads per sample varied between 10,062 and 282,039. Singletons were accordingly removed and after normalization using rarefaction, 1586 ASVs were removed with remaining 652 ASVs in total for 8 samples. The non-metric dimensional scaling (nMDS) ordinations was performed using the Bray-Curtis dissimilarity index which was calculated from the relative abundances of microbial taxa. The analysis was conducted using maximum iterations of 100 and a stress reduction tolerance of 0.0001 using *phyloseq* v1.40 (4). An Analysis of Similarity (ANOSIM) was implemented using the same Bray-Curtis dissimilarity index using *vegan* v2.6 (5). The analysis was performed with 999 permutations and significance was determined using the test statistic (R) and significant p-value at 0.001. Similarity percentage analysis (SIMPER) was performed to identify ASVs that contributed most to the dissimilarities between the glacial and non-glacial amended colloidal treatments using the Bray-Curtis dissimilarity index with *vegan* v2.6. Contributions were ranked by average dissimilarity and only ASVs contributing more than 70% dissimilarity were considered relevant to the analysis.

Metagenomic gene sequencing

The quantified gene occurrences in gene samples obtained from *salmon* v.1.10.2 were normalized as genes per kilobase million (GPM) based on the formula retrieved from (6) where:

GPM =

$$\frac{\frac{Number\ of\ reads\ mapped\ to\ genes}{genes\ length\ in\ base\ pairs}}{Sum(\frac{Number\ of\ reads\ mapped\ to\ genes}{genes\ length\ in\ base\ pairs})}\ x\ 10\text{^}6$$

This metric can be used for metagenomes to remove gene length effect and ensure comparability between samples and taking sequencing depth into account.

Supplementary References

1. Aminot A, Kéroul R. Dosage automatique des nutriments dans les eaux marines - Alain... - Librairie Eyrolles. Vol. Collection Méthodes d'analyse en milieu marin. 2007. 188 p.
2. Benner R, Strom M. A critical evaluation of the analytical blank associated with DOC measurements by high-temperature catalytic oxidation. Marine Chemistry. 1993 Jan 1;41(1):153–60.
3. Callahan BJ, McMurdie PJ, Rosen MJ, Han AW, Johnson AJA, Holmes SP. DADA2: High-resolution sample inference from Illumina amplicon data. Nat Methods. 2016 Jul;13(7):581–3.
4. McMurdie PJ, Holmes S. phyloseq: An R Package for Reproducible Interactive Analysis and Graphics of Microbiome Census Data. PLOS ONE. 2013 Apr 22;8(4):e61217.
5. Dixon P. VEGAN, a package of R functions for community ecology. Journal of Vegetation Science. 2003;14(6):927–30.
6. Zhang R, Debeljak P, Blain S, Obernosterer I. Seasonal shifts in Fe-acquisition strategies in Southern Ocean microbial communities revealed by metagenomics and autonomous sampling. Environ Microbiol. 2023 Oct;25(10):1816–29.
7. Styczynski M, Biegniewski G, Decewicz P, Rewerski B, Debiec-Andrzejewska K, Dziewit L. Application of Psychrotolerant Antarctic Bacteria and Their Metabolites as Efficient Plant Growth Promoting Agents. Front Bioeng Biotechnol. 2022 Feb 24;10:772891.
8. Johnstone TC, Nolan EM. Beyond iron: non-classical biological functions of bacterial siderophores. Dalton Trans. 2015 Mar 25;44(14):6320–39.

Supplementary Figures

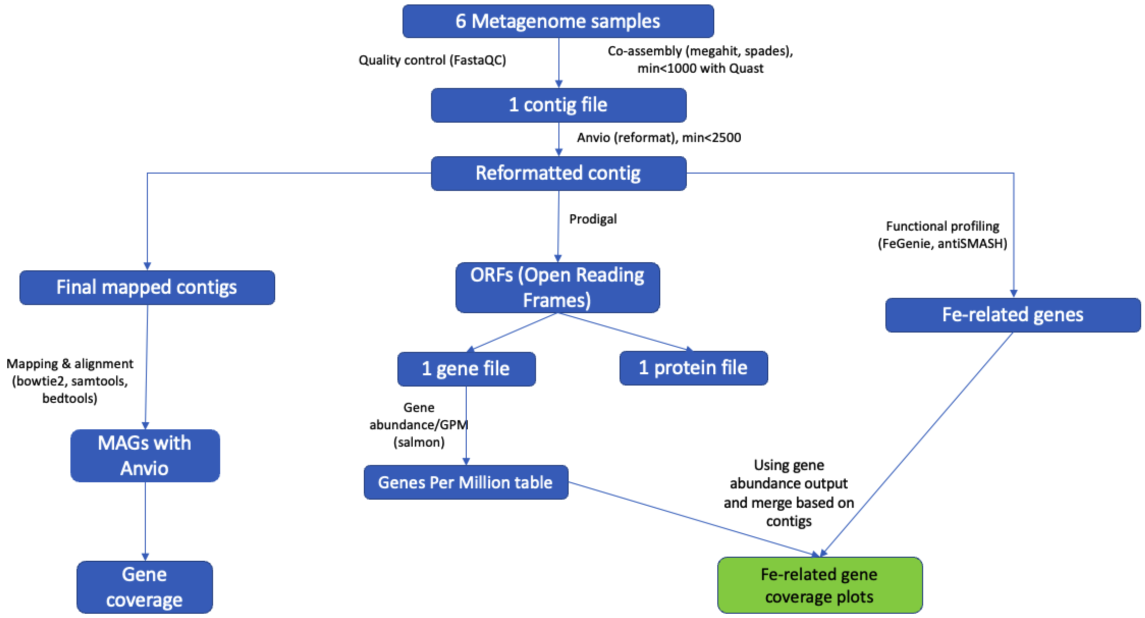

Supplementary Figure. 1 Schematic representation of the bioinformatic pipeline designed for the co-assembly metagenomic approach for the samples used in the study with details on the type of packages used and respective parameters.

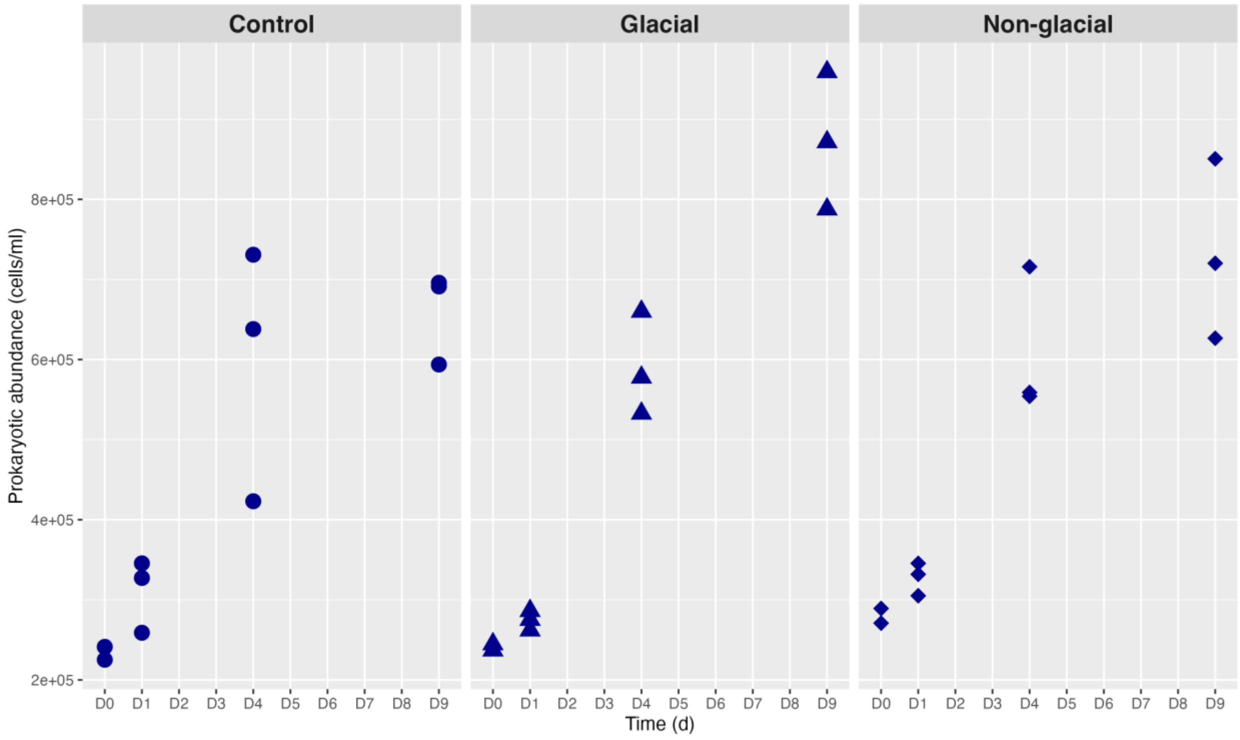

Supplementary Figure. 2 Dot plot displaying prokaryotic abundances in the triplicate batch cultures during the incubation period (10 days) in the control (Ctrl) and the two treatments, amended with glacial and non-glacial colloids.

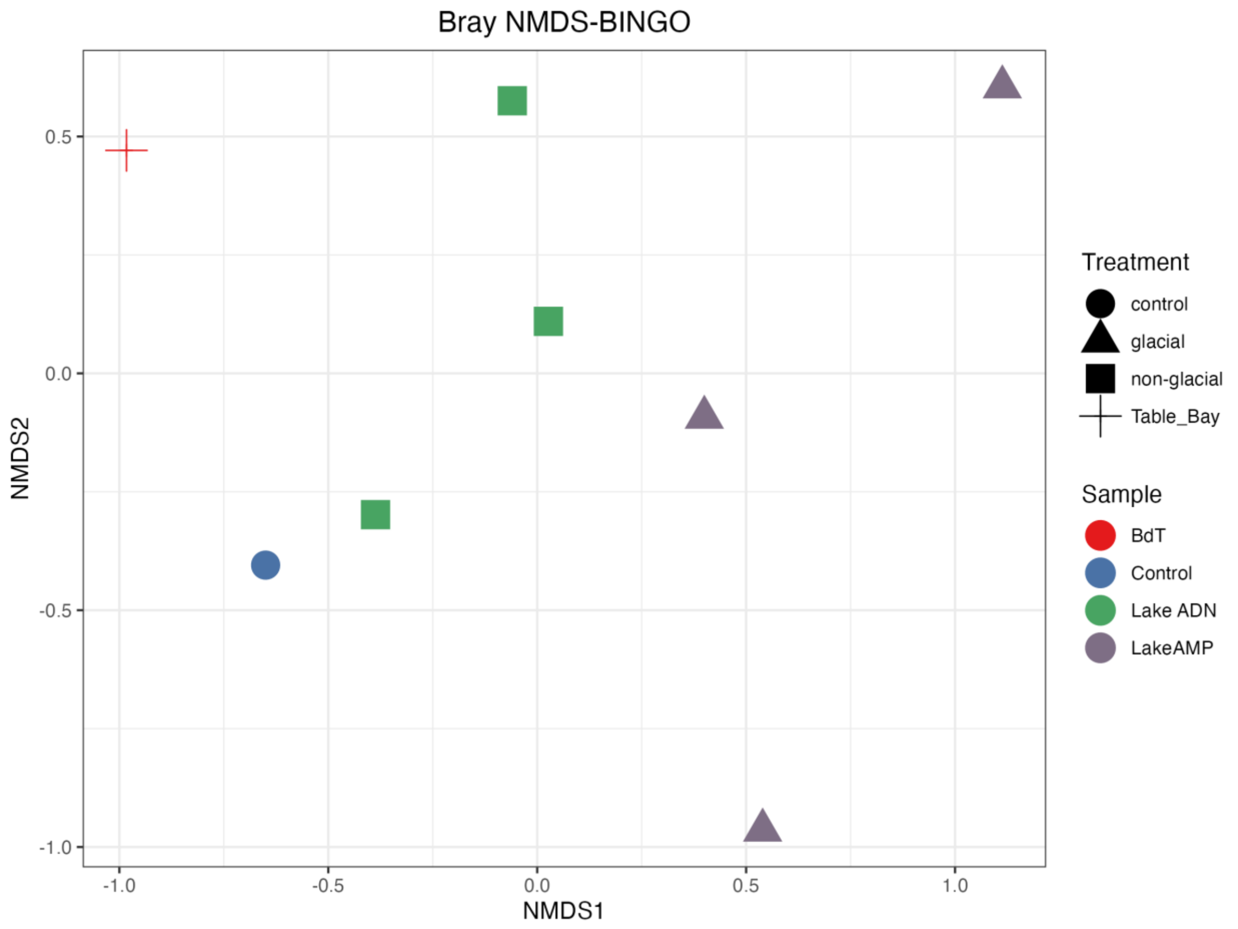

Supplementary Figure. 3 Non-Metric Multidimensional Scaling (NMDS) ordination of microbial community composition across 8 environmental samples based on Bray-Curtis dissimilarity. Colours indicate sampling sites such as red: BdT (Table Bay), blue: Control, green: Lake AND (Non-glacial), purple: Lake AMP (Glacial), and shapes represent type of samples (circle: Control, triangle: Glacial, Square: Non-glacial, Cross: Table_Bay) with stress at 0.1

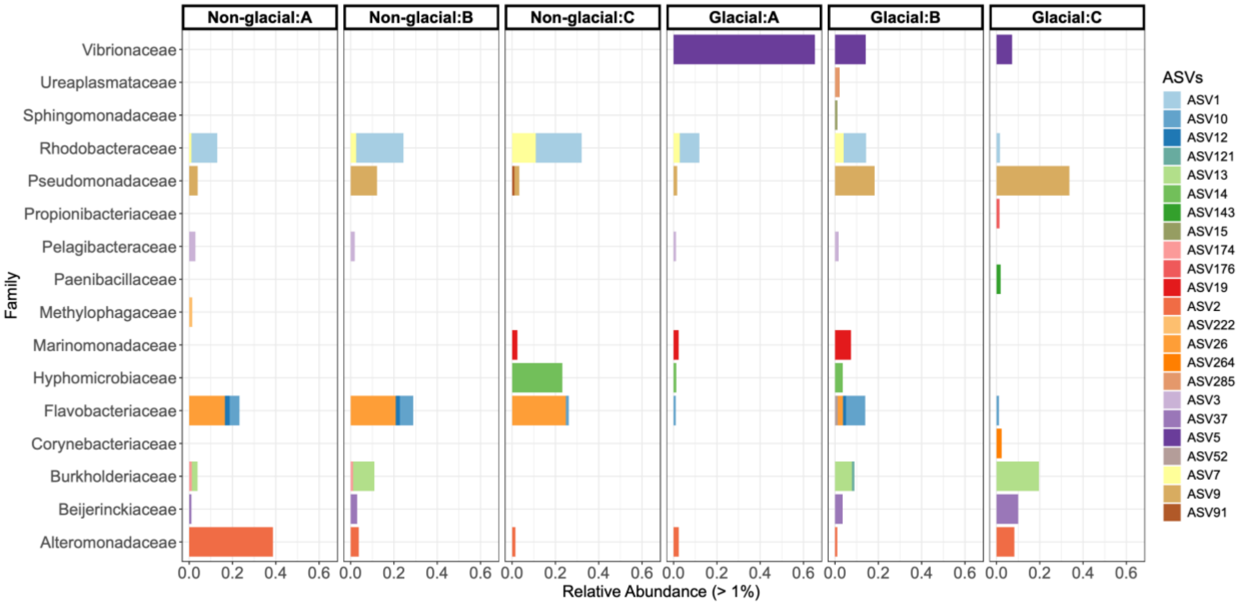

Supplementary Figure. 4 Relative abundance of ASVs (>1%) grouped at the family level showing the composition of microbial communities in the incubations amended with non-glacial and glacial colloids shown as replicates for each treatment.

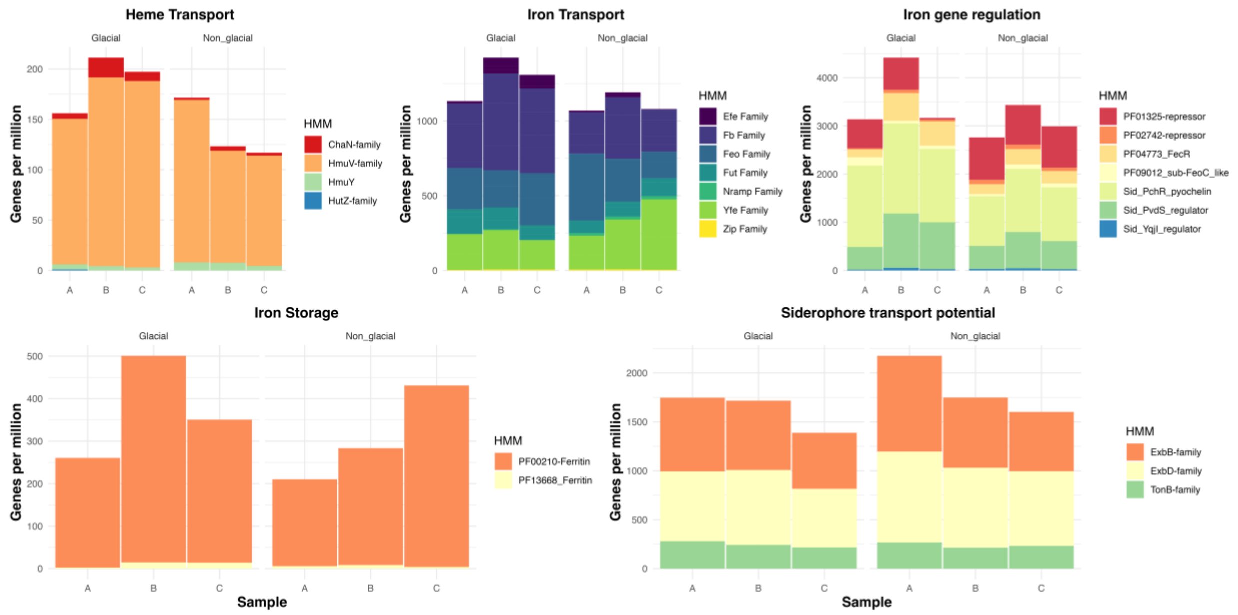

Supplementary Figure. 5 Stacked bar plots of normalized gene abundances (GPM) in incubations with glacial and non-glacial colloids in each replicate (A, B, C) obtained using FeGenie. Top-left: Heme transport genes. Top-centre: Iron transport genes. Top-right: Iron gene regulation genes. Bottom-left: Iron storage genes. Bottom-right: Siderophore transport potential genes

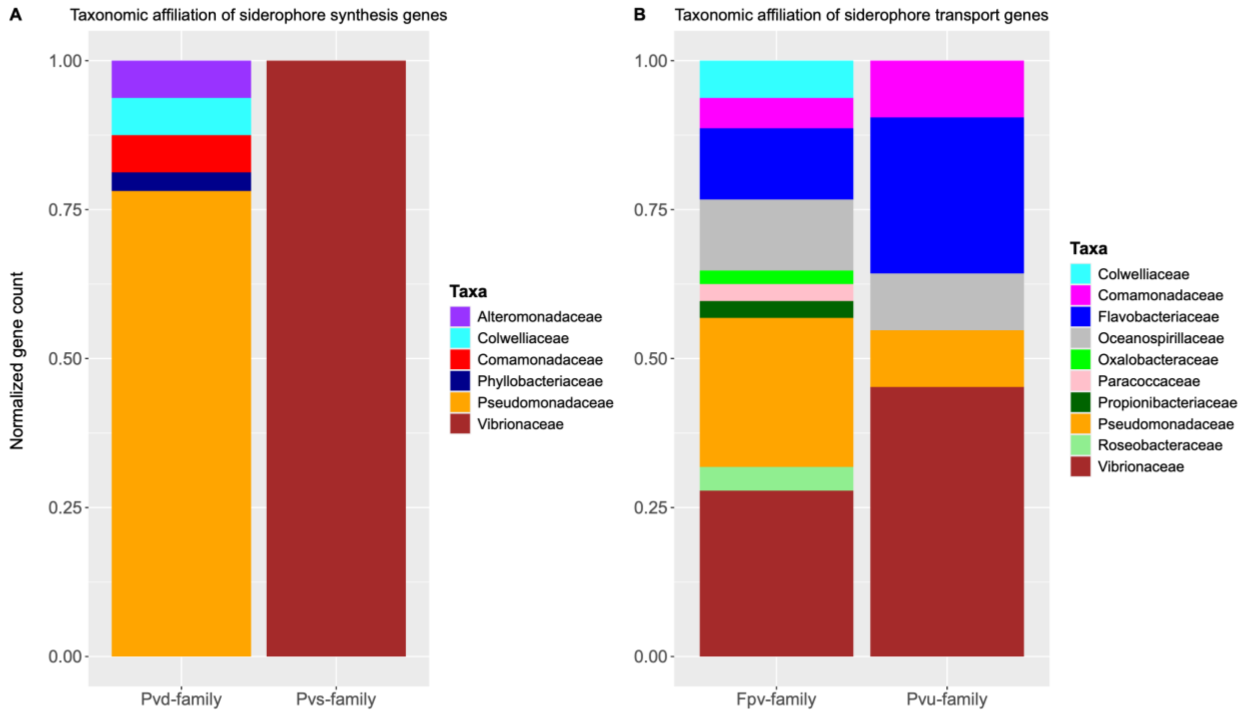

Supplementary Figure. 6 Relative contribution of prokaryotic taxa to the taxonomic affiliation of (A) significant siderophore synthesis genes (*pvd* and *pvs*) and (B) siderophore transport genes (*fpv* and *pvu*) (GPM) assigned using BlastP.

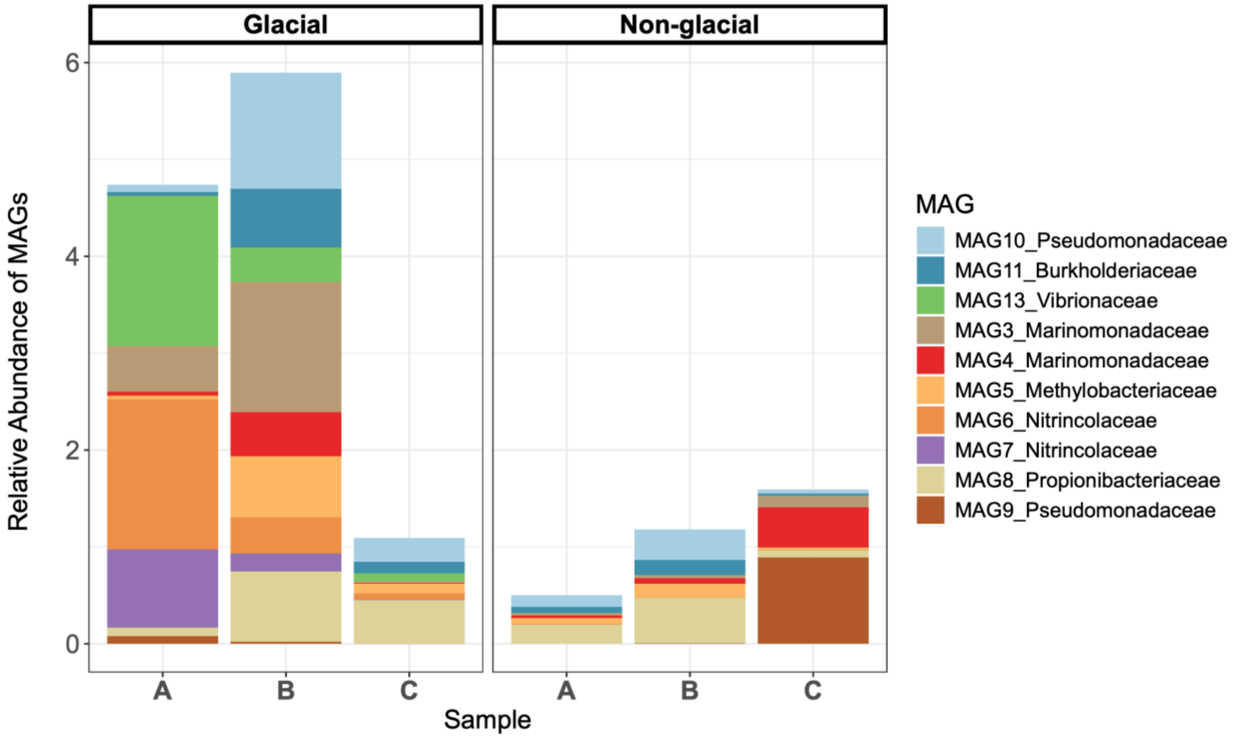

Supplementary Figure. 7 Relative abundance of MAGs of interest grouped in each biological replicate (A, B, C) of the incubations amended with glacial (left) and non-glacial colloids (right) are shown.

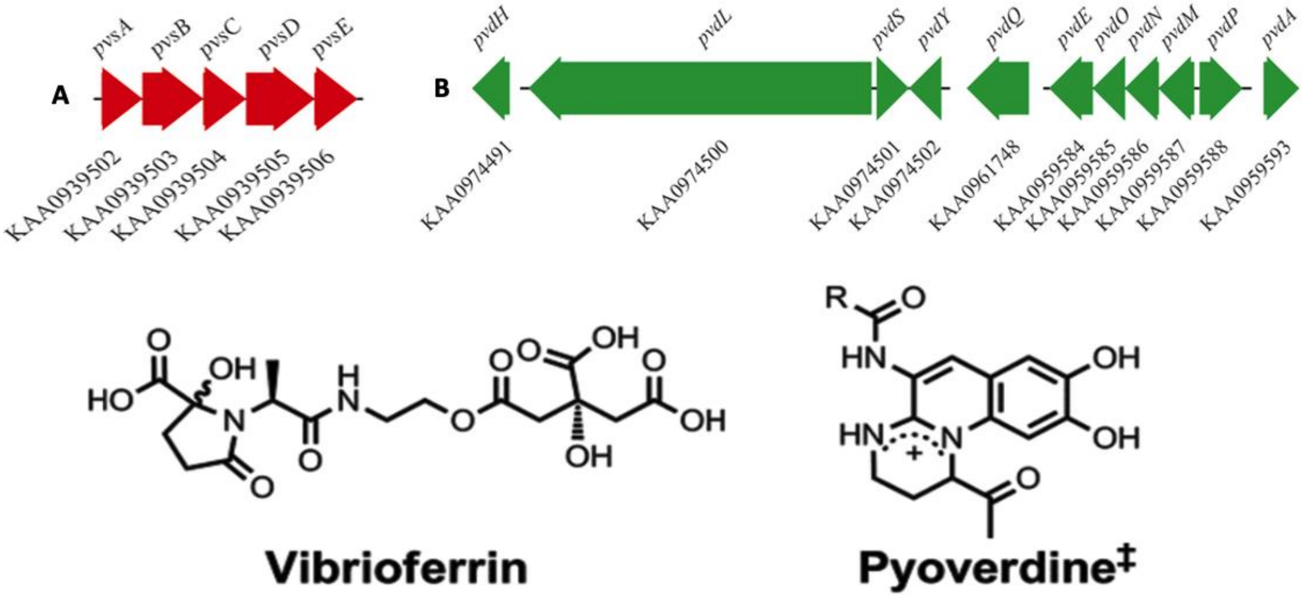

Supplementary Figure. 8 Biosynthetic gene clusters involved in siderophore synthesis adapted from (7) with gene names and associated GenBank accession numbers for (A) Vibrioferrin and (B) Pyoverdine along with their respective chemical structures adapted from (8)

Table S1. Statistics of amplicon sequence variants (ASVs) of samples from the incubation experiments and the *Baie de la Table*. Non-glacial: Incubations amended with non-glacial colloids; Glacial: Incubations amended with glacial colloids; *Baie de la Table*: microbial community used as inoculum; Control 1: the only control incubation for which DNA was available.

**Samples**	**ASV**				
	**# Raw read pairs**	**# Adapter clipped read pairs**	**# Primer Clipped read pairs**	**# Combined reads**	**#No. of reads per sample**
**Non-glacial-a**	26,72	26,717	26,493	26,439	23,958
**Non-glacial-b**	58,8	58,78	58,228	58,104	52,686
**Non-glacial-c**	50,641	50,634	50,29	50,184	43,668
**Glacial-a**	31,664	31,661	31,378	31,281	26,039
**Glacial-b**	11,469	11,41	11,208	11,188	10,062
**Glacial-c**	24,986	24,858	24,368	24,308	21,502

**Baie de la Table (inoculum)**	33,694	33,694	33,427	33,356	24,183
**Control_1**	315,859	315,855	313,898	313,075	282,039

Table S2. Sequencing statistics of the metagenomes from samples at the final time point of the incubations amended with non-glacial and glacial colloids.

<table>
<tr><th></th><th colspan="5">Metagenome</th></tr>
<tr><th></th><th># Read Pairs</th><th>Avg. base pairs (bp)*</th><th># Contigs (≥ 1,000 bp)</th><th>Overall alignment rate (%)</th><th>No. of genes</th></tr>
<tr><td>Non-glacial_a</td><td>212 405 116</td><td rowspan="6">3 642,00</td><td rowspan="6">175 478</td><td>81,10</td><td rowspan="6">499,592</td></tr>
<tr><td>Non-glacial_b</td><td>204 554 126</td><td>72,56</td></tr>
<tr><td>Non-glacial_c</td><td>271 149 520</td><td>88,14</td></tr>
<tr><td>Glacial_a</td><td>186 715 192</td><td>78,42</td></tr>
<tr><td>Glacial_b</td><td>246 872 186</td><td>79,83</td></tr>
<tr><td>Glacial_c</td><td>194 399 174</td><td>18,04</td></tr>
</table>

* Co-assembly approach using megahit

Table S3. List of all detected amplicon sequence variants (ASVs) with taxonomic assignments and respective relative abundances based on the type of treatments: glacial and non-glacial amended incubations (Provided as a separate pdf file at end of document: Table_S3.pdf)

Table S4. Summary of SIMPER statistics conducted on amplicon sequence variants (ASVs) using a pair-wise comparison between glacial and non-glacial amended incubations describing the cumulative contribution of the ASV with a p-value < 0.05 along with their taxonomic assignments

OTU	Comparison	SIMPER contribution	p	Kingdom	Phylum	Class	Order	Family	Genus
ASV5	non-glacial_glacial	0,175540901	0,00138889	Bacteria	Proteobacteria	Gammaproteobacteria	Enterobacterales	Vibrionaceae	Vibrio
ASV1	non-glacial_glacial	0,070657802	0,00138889	Bacteria	Proteobacteria	Alphaproteobacteria	Rhodobacterales	Rhodobacteraceae	Sulfitobacter
ASV26	non-glacial_glacial	0,067496866	0,00138889	Bacteria	Bacteroidota	Bacteroidia	Flavobacteriales	Flavobacteriaceae	Flavobacterium
ASV6	non-glacial_glacial	0,011608262	0,00138889	Bacteria	Proteobacteria	Alphaproteobacteria	Rhodobacterales	Rhodobacteraceae	Amylibacter
ASV174	non-glacial_glacial	0,006185623	0,00138889	Bacteria	Proteobacteria	Gammaproteobacteria	Burkholderiales	Oxalobacteraceae	Rugamonas
ASV188	non-glacial_glacial	0,004060167	0,00138889	Bacteria	Bacteroidota	Bacteroidia	Cytophagales	Spirosomaceae	Arcicella
ASV264	non-glacial_glacial	0,003842171	0,00138889	Bacteria	Actinobacteriota	Actinobacteria	Corynebacteriales	Corynebacteriaceae	Corynebacterium
ASV120	non-glacial_glacial	0,003760423	0,00138889	Bacteria	Proteobacteria	Gammaproteobacteria	Enterobacterales	Vibrionaceae	Aliivibrio
ASV187	non-glacial_glacial	0,0024252	0,00138889	Bacteria	Proteobacteria	Alphaproteobacteria	Rhodobacterales	Rhodobacteraceae	Jannaschia
ASV100	non-glacial_glacial	0,001008229	0,00138889	Bacteria	Proteobacteria	Gammaproteobacteria	Enterobacterales	Alteromonadaceae	Glaciecola

Table S5. Summary statistics and taxonomic assignment of the metagenome-assembled genomes (MAGs) (Provided as a separate pdf file at end of document: Table_S5.pdf)

Table S6. Description of each HMM identified using antiSMASH for each MAG detected based on significant MAGs from FeGenie results for (A) siderophore synthesis and (B) siderophore transport genes where some MAGs have more than one HMM assigned to it.

(A)

MAG	ID	HMM	Description	Closest match
maxbin 024	MAG10_Pseudomonadacea	IucA/IucC family protein	Involved in the production of multiple siderophores (Mydy et al., 2021)	[Pseudomonas]
maxbin 024	MAG10_Pseudomonadacea	non-ribosomal peptide synthetase	Needed to produce siderophores in Gram -ve bacteria (Li et al., 2018)	[Pseudomonas]
maxbin 110	MAG13_Vibrionaceae	IucA/IucC family siderophore biosynthesis protein	Involved in the production of multiple siderophores (Mydy et al., 2021)	[Vibrio splendidus]
maxbin 117	MAG3_Marinomonadaceae	non-ribosomal peptide synthetase	Needed to produce siderophores in Gram -ve bacteria (Li et al., 2018)	[Marinomonas]
Bin_32	MAG5_Methylobacteriacea	IucA/IucC family protein	Involved in the production of multiple siderophores (Mydy et al., 2021)	[Methylorubrum populi]
Bin_95_sub	MAG6_Nitrincolaceae	IucA/IucC family siderophore biosynthesis protein	Involved in the production of multiple siderophores (Mydy et al., 2021)	[Vibrio]
Bin_95_sub	MAG6_Nitrincolaceae	siderophore biosynthesis protein PvsD	Synthesis of siderophore vibrioferrin (Tanabe et al., 2003)	[Vibrio splendidus]
maxbin 043	MAG8_Propionibacteriacea	IucA/IucC family protein	Involved in the production of multiple siderophores (Mydy et al., 2021)	[Pseudomonas]
maxbin 143	MAG9_Pseudomonadaceae	IucA/IucC family siderophore biosynthesis protein	Involved in the production of multiple siderophores (Mydy et al., 2021)	[Pseudomonas]

(B)

MAG	ID	HMM	Description	Closest match
Bin_95_sub	MAG6_Nitrincolaceae	TonB-dependent siderophore receptor	Mediates transport of siderophores in Gram -ve bacteria (Ferguson & Diesenhofer,2002)	(Vibrio)
maxbin_042	MAG11_Burkholderiaceae	TonB-dependent siderophore receptor	Mediates transport of siderophores in Gram -ve bacteria (Ferguson & Diesenhofer,2002)	[Delftia]
maxbin_024	MAG10_Pseudomonadaceae	TonB-dependent receptor	Mediates transport of siderophores in Gram -ve bacteria (Ferguson & Diesenhofer,2002)	[Pseudomonas]
maxbin 110	MAG13_Vibrionaceae	TonB-dependent receptor	Mediates transport of siderophores in Gram -ve bacteria (Ferguson & Diesenhofer,2002)	[Vibrio]
maxbin 043	MAG8_Propionibacteriaceae	MFS transporter	Major facilitator superfamily (membrane transport proteins) that transport sugars, metabolites, neurotransmitters, siderophores and organic anions (Pao et al 1998)	[Pseudomonas]
maxbin 117	MAG3_Marinomonadaceae	MFS transporter	Major facilitator superfamily (membrane transport proteins) that transport sugars, metabolites, neurotransmitters, siderophores and organic anions (Pao et al 1998)	Marinomonas
maxbin_024	MAG10_Pseudomonadaceae	MFS transporter	Major facilitator superfamily (membrane transport proteins) that transport sugars, metabolites, neurotransmitters, siderophores and organic anions (Pao et al 1998)	[Pseudomonas]
maxbin 043	MAG8_Propionibacteriaceae	MFS transporter	Major facilitator superfamily (membrane transport proteins) that transport sugars, metabolites, neurotransmitters, siderophores and organic anions (Pao et al 1998)	[Pseudomonas]
maxbin 117	MAG3_Marinomonadaceae	iron chelate uptake ABC transporter family permease subunit	Transporter specific for siderophore (Dassa 2006)	[Marinomonas]
maxbin 117	MAG3_Marinomonadaceae	Fe(3+)-siderophore ABC transporter permease	Transporter specific for siderophore (Koster 2005)	[Marinomonas]
maxbin 117	MAG3_Marinomonadaceae	enterobactin transporter EntS	Siderophore transporter for enterobactin (Earhart 2004)	[Marinomonas]
maxbin_024	MAG10_Pseudomonadaceae	cyclic peptide export ABC transporter	Cyclic peptide transporter for PvDE and SyrD (lipodepsipeptide) from Pseudomonas (ATP-Binding cassette) superfamily (Girard et al., 2020)	[Pseudomonas]
maxbin_042	MAG11_Burkholderiaceae	cyclic peptide export ABC transporter	Cyclic peptide transporter for PvDE and SyrD (lipodepsipeptide) from Pseudomonas (ATP-Binding cassette) superfamily (Girard et al., 2020)	[Pseudomonas]
maxbin_043	MAG8_Propionibacteriaceae	ABC transporter ATP-binding protein	Transport or import of mutliple siderophores (Davidson et al., 2008)	[Massilia atriviolacea]
maxbin_152	MAG7_Nitrincolaceae	ABC transporter ATP-binding protein	Transport or import of mutliple siderophores (Davidson et al., 2008)	[Pontibacterium]
maxbin_024	MAG10_Pseudomonadaceae	ABC transporter ATP-binding protein	Transport or import of mutliple siderophores (Davidson et al., 2008)	[Pseudomonas]
maxbin 117	MAG3_Marinomonadaceae	ABC transporter ATP-binding protein	Transport or import of mutliple siderophores (Davidson et al., 2008)	[Marinomonas]

221
